# Exploring functions of long noncoding RNAs across multiple cancers through co-expression network

**DOI:** 10.1038/s41598-017-00856-8

**Published:** 2017-04-07

**Authors:** Suqing Li, Bin Li, Yuanting Zheng, Menglong Li, Leming Shi, Xuemei Pu

**Affiliations:** 1 College of Chemistry, Sichuan University, Chengdu, 610064 China; 2 Center for Pharmacogenomics, School of Life Sciences, and State Key Laboratory of Genetic Engineering and Shanghai Cancer Center/Cancer Institute, Fudan University, Shanghai, 201203 China; 3 Collaborative Innovation Center for Genetics and Development, Fudan University, Shanghai, 200438 China

## Abstract

In contrast to protein-coding genes, long-noncoding RNAs (lncRNAs) are much less well understood, despite increasing evidence indicating a wide range of their biological functions, and possible roles in various cancers. Based on public RNA-seq datasets of four solid cancer types, we here utilize Weighted Correlation Network Analysis (WGCNA) to propose a strategy for exploring the functions of lncRNAs altered in more than two cancer types, which we call onco-lncRNAs. Results indicate that cancer-expressed lncRNAs show high tissue specificity and are weakly expressed, more so than protein-coding genes. Most of the 236 onco-lncRNAs we identified have not been reported to have associations with cancers before. Our analysis exploits co-expression network to reveal that onco-lncRNAs likely play key roles in the multistep development of human cancers, covering a wide range of functions in genome stability maintenance, signaling, cell adhesion and motility, morphogenesis, cell cycle, immune and inflammatory response. These observations contribute to a more comprehensive understanding of cancer-associated lncRNAs, while demonstrating a novel and efficient strategy for subsequent functional studies of lncRNAs.

## Introduction

Long noncoding RNA (lncRNA) belongs to a class of noncoding RNAs longer than 200 nucleotides^1, 2^. With the development of RNA sequencing, epigenomic technologies and computational techniques, an increasing number of lncRNAs have been discovered^3^. Although lncRNAs were previously regarded as “noise” in the genome owing to lack of protein-encoding capacity, more and more emerging evidences have indicated that the lncRNAs play a wide range of roles, covering biological functions like cell proliferation, survival, differentiation, and chromatin remodeling^4–7^. Consequently, it is not surprising that the dysregulation of lncRNA genes was implicated in tumor biology^8^.

However, compared to well-studied protein-coding genes, the functions of most lncRNAs have not been elucidated despite of their large proportions in genomes. In the lncRNAdb v2.0^9^, less than 1% of lncRNAs have been individually characterized among nearly 16,000 annotated lncRNA genes in GENCODE. Thus, it remains a great challenge in understanding the functional characteristics of lncRNAs. In general, loss- and gain-of function biological experiments through gene knockdown, overexpression or editing are considered to be golden standards to define the functions of lncRNAs^10^. However, the characterization through the experimental approaches is still limited due to their low throughput and demand for prior knowledge about potential mechanisms of the candidates^11^.

Alternatively, computational analysis provides another way to explore the functions of the lncRNAs. Some computational work predicted lncRNA structures based on their sequences^12, 13^. However, the structures predicted by the computational methods still remain a high false-positive rate, and the distinct structure–function relationships for many lncRNAs are still unknown^14^. In addition, some computational studies explored the potential functions of lncRNAs through identifying molecules interacting with them^15, 16^. But, the lack of molecular interaction data for many lncRNAs also hampers their functional annotation.

It is well accepted that co-expressed genes are more likely to be co-regulated and functionally related^17^. Therefore, identifying co-expressed protein-coding genes can help assign the functions of lncRNAs^11^. Weighted correlation network analysis (WGCNA), a powerful guilt-by-association (GBA) method for constructing co-expression network based on expression data, can reconstruct gene co-expression modules and summarize such modules using module eigengenes and intramodular hub genes^18^. It has been successfully applied to study protein-coding genes, like distinguishing dysfunctional regulatory subnetworks and finding candidate biomarkers^19^. However, few studies used it to investigate cancer-associated lncRNAs^20^. Cogill *et al*.^21^ used known cancer-associated coding genes from COSMIC to find co-expressed lncRNAs only from microarray expression data of normal tissues rather than cancer tissues, and constructed a co-expression network using WGCNA to explore their potential functions.

In recent years, the Cancer Genome Atlas (TCGA, https://cancergenome.nih.gov/) project has generated comprehensive, multi-dimensional maps of the key genomic changes in 33 types of cancers, which help us understanding how such changes interact to drive diseases. From this project, researchers found that cancers from different tissues could share some common features like mutations, methylation, and transcriptomic changes, and the cross-cancer aberrations are more likely to act as oncogenic contributors and can provide an opportunity to find new therapeutic biomarkers in clinics^22, 23^. In fact, some previous work showed that a few lncRNAs are altered in multiple cancers. For example, MALAT1 was first identified as a prognostic biomaker for lung cancer survival^24^. Later, its expression dysregulation was also observed in other types of tumors, including malignancy in liver^25^, breast^26^ and colon^27^. In addition, other lncRNAs like HOTAIR, PTENP1, MEG3 and CONCR were reported to be dysregulated in several cancer types^28, 29^. Yan *et al*.^30^ also observed that some lncRNAs are abnormally expressed in several cancers. However, most works focused on cancer-associated lncRNAs only in independent cancer type^31^ while studies on the lncRNAs across multiple cancers have been absent. In fact, this kind of lncRNAs may be proved as potential oncogenes or tumor suppressors across multiple cancers and extend our understanding of the common events across tumor types. Thus, it is highly desired to study these poorly understood but crucial regulators across multiple cancers.

In this work, we utilized a computational strategy to perform a systematic study on lncRNAs significantly altered in more than 2 cancer types, based on public RNA-seq datasets of four common solid cancer types (prostate cancer, bladder cancer, lung adenocarcinoma and breast cancer). RNA-seq is a revolutionary technology based on next-generation sequencing, and is considered as the most comprehensive way for studying complete transcriptome in more details and with more accurate measurements than other techniques of lncRNAs expression profiling like microarray and serial analysis of gene expression (SAGE)^4^. Finally, 236 onco-lncRNAs were identified in our work, and most of them have not been reported to be related with cancers. WGCNA combined with DAVID (the database for annotation, visualization and integrated discovery)^32^ were used to explore their functions. We revealed that the onco-lncRNAs likely take key roles in the multistep development of human cancers, covering a wide range of functions in genome stability maintenance, signaling, cell adhesion and motility, morphogenesis, cell cycle, immune and inflammatory response. Our study contributes to a comprehensive understanding of the onco-lncRNAs with the aid of the co-expression network, which may guide subsequently experimental studies on the altered lncRNAs in cancers.

## Results

### Expression profiles of lncRNAs across cancers

We downloaded public RNA-seq datasets containing four cancer types for our analysis: bladder cancer (BLC)^33^, prostate cancer (PRC)^34^, lung adenocarcinoma (ADC)^35^ and estrogen receptor positive (ER+) breast cancer (EBC)^36^ (Supplementary Table S1). GENCODE v23 gtf file, containing 19,797 protein-coding genes and 15,931 lncRNA genes, was used for annotation.

**Figure 1:**
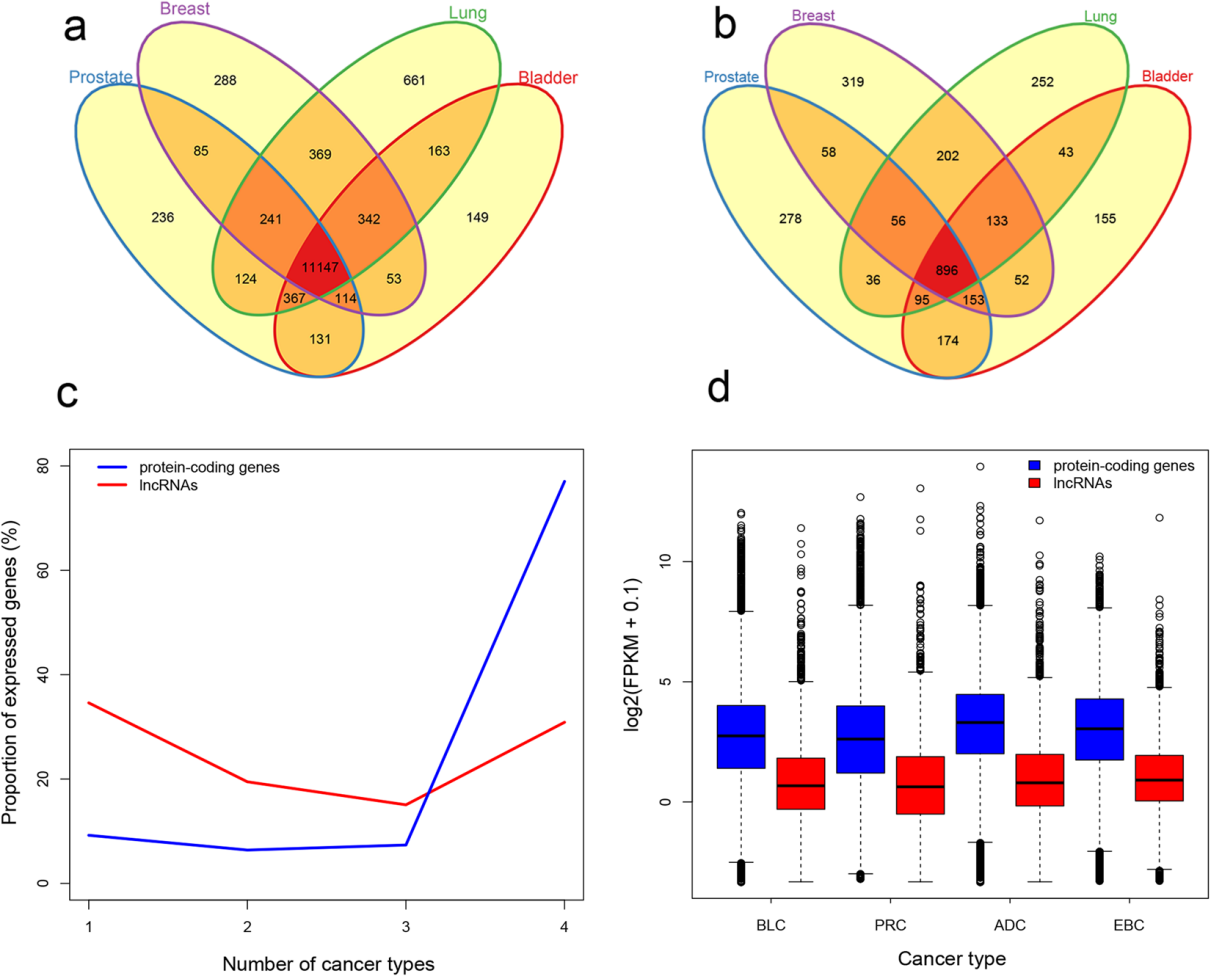
Distributions and expression levels of expressed genes in the four cancer types. The venn plot of 14,470 expressed protein-coding genes (**a**) and 2,902 expressed lncRNA genes (**b**). (**c**) The proportion of expressed protein-coding genes (blue) and lncRNA genes (red) appearing in different number of cancers. The x axis depicts the number of cancer types. The y-axis depicts the proportion of expressed genes, which is the ratio between the counts of expressed genes appearing in different number of cancers and the total counts of all expressed genes. (**d**) The expression levels (log2(FPKM + 0.1)) of expressed protein-coding genes (blue) and lncRNAs (red) in each cancer type.

After mapping and quantification, we defined expressed genes based on a threshold of FPKM ≥ 1 in more than 80% of normal samples or 80% of tumor samples for each cancer type. Consequently, there are total 14,470 expressed protein-coding genes (PCGs) (73.1% of all annotated protein-coding genes in the GENCODE) and 2,902 expressed lncRNA genes (18.2% of all annotated lncRNA genes in the GENCODE) in the four cancer types. For all the expressed lncRNAs and PCGs, we calculated the number and proportion of expressed genes appearing in different number of cancers (Fig. 1a–c). We found that the majority (77.0%) of the expressed PCGs are detected in all the four cancers compared with 30.1% of the lncRNAs. Meanwhile, a minority (9.2%) of the PCGs show expression in only one cancer in contrast with a bigger proportion (34.6%) of the lncRNAs. We also computed distributions of FPKM values for the expressed lncRNAs and PCGs in each cancer type. As shown in Fig. 1d, the lncRNAs have a lower expression level than the PCGs in all the cancer types. The observation provides further support for previous observations that the expression of lncRNA genes displays much more tissue-specific and lower expression than PCGs^37^.

### Differentially expressed lncRNA genes

**Figure 2:**
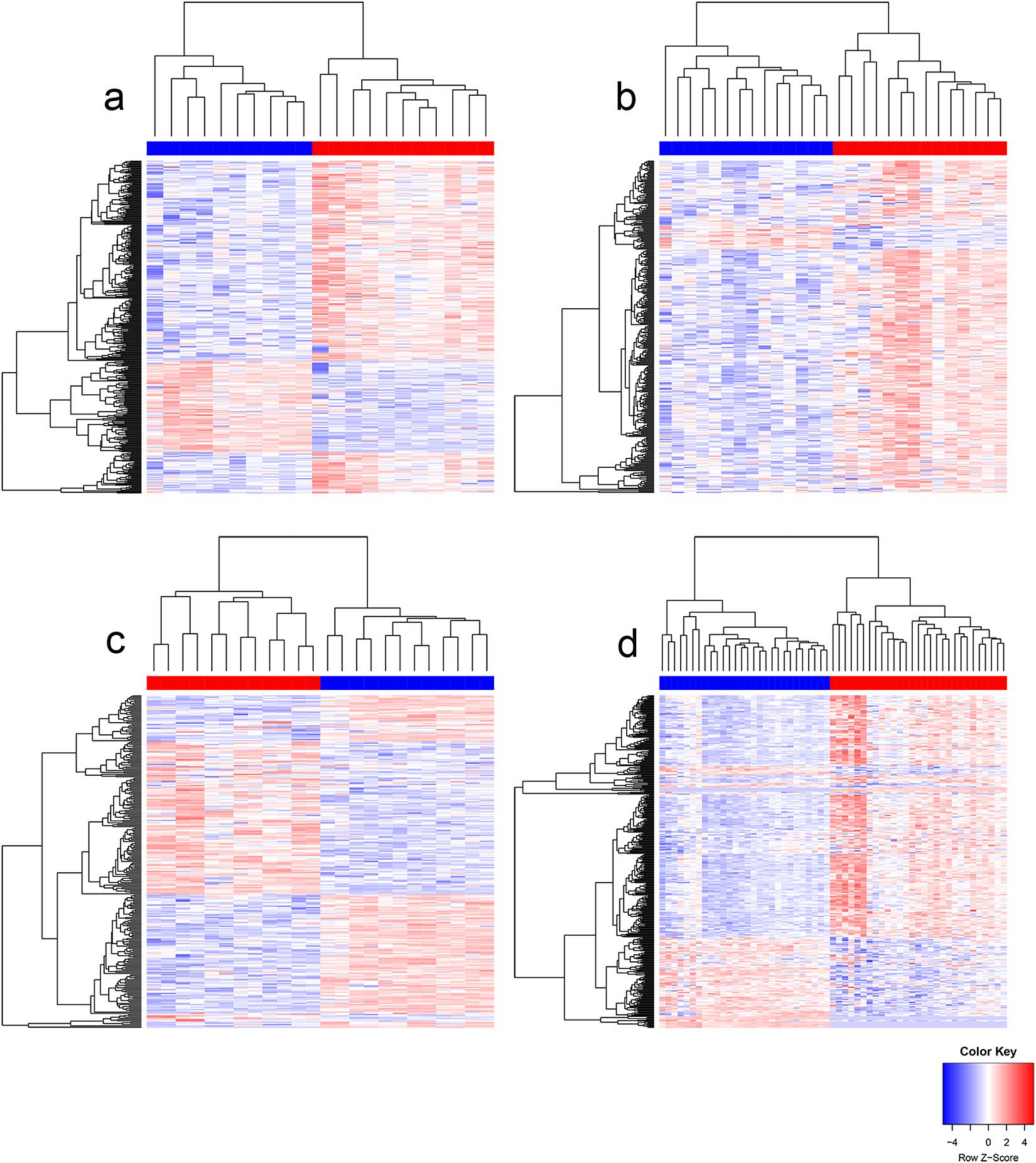
Hierarchical clustering based on expression profiles of significantly differentially expressed lncRNA genes (DELs) from each cancer type. (**a**) The heatmap of 357 DELs in bladder cancer. (**b**) The heatmap of 321 DELs in prostate cancer. (**c**) The heatmap of 267 DELs lung adenocarcinoma. (**d**) The heatmap of 375 DELs in breast cancer. The intensity of the color scheme is scaled to expression values (log2(FPKM + 0.1)) which are Z-score standardized per gene. The color bar above the heatmap represents the sample groups, and red indicates tumor sample, and blue represents normal sample.

We defined differentially expressed genes between the tumor samples and matched normal samples based on the following criteria: fold change ≥2 or ≤0.5 and FDR ≤ 0.01. And we got 357, 321, 267 and 375 differentially expressed lncRNAs (DELs) in BLC, PRC, ADC and EBC, respectively. The total number of DELs for the four cancer types is 1,010. To obtain an overview of the expression profile for DELs in each cancer, we performed hierarchical cluster analysis (Fig. 2). It can be seen that all heatmaps show a distinct regulating direction and a clear separation between the normal samples and the tumor ones for the DELs. In addition, we also identified 5,595 differential expressed protein-coding genes (DEPs) in all.

Among all the DELs, there are 774 (76.6% of the 1,010 DELs) lncRNAs which are differentially expressed only in one cancer type (Supplementary Table S2). Only few DELs here were indicated by earlier works to be associated with cancers (Supplementary Table S3). For instance, UCA1 was reported to play a regulatory role in promoting human bladder cancer proliferation^38^. In our analysis, it is up-regulated (log2FC = 3.3, FDR = 4.5 × 10^−4^) in BLC. AATBC is also differentially expressed (log2FC = 3.6, FDR = 2.5 × 10^−9^) in BLC, which was reported to facilitate proliferation and inhibit cell apoptosis in bladder cancer^39^. PCAT29, as a new biomarker in prostate cancer^40^, is the most significant DELs (log2FC = 2.68, FDR = 4.43 × 10^−15^) in PRC from our analysis. CTBP1-AS is observed to be significantly altered (log2FC = 2.7, FDR = 8.2 × 10^−10^) in PRC, which was reported to be an androgen-responsive lncRNA in prostate cancer^41^. The consistency between our results and the findings from earlier works confirms the reliability of our analysis method. Intriguingly, most of DELs altered in only one cancer type have not been reported to be related to cancer yet, for example, MIR99AHG, the most down-regulated DEL (log2FC = −6.44, FDR = 1.23 × 10^−27^) in BLC, and LINC00968, a significantly down-regulated DEL (log2FC = −3.77, FDR = 1.58 × 10^−49^) in ADC. These unreported lncRNAs could provide helpful information for possible biomarkers in further experiments owing to their significant dysregulation in the specific tumor type.

**Figure 3:**
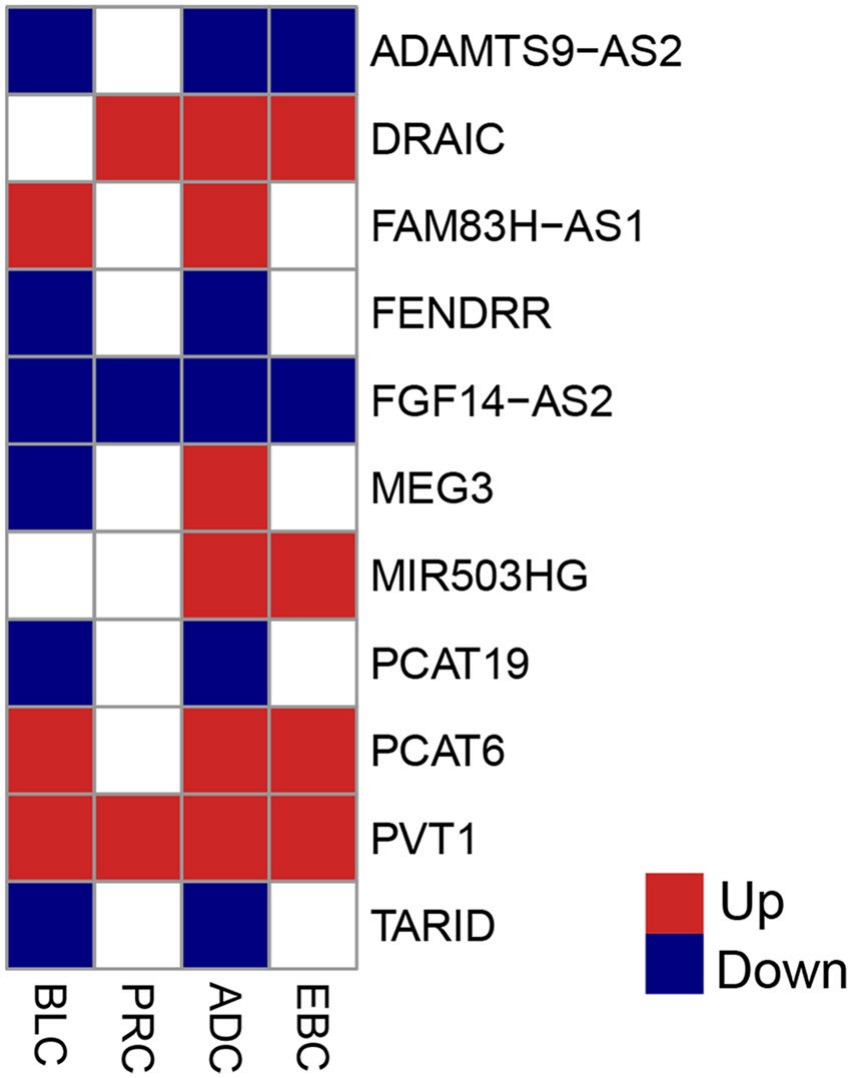
Heatmap of the eleven well-characterized onco-lncRNAs across all the four cancers. Red denotes up-regulation, and blue indicates down-regulation. Blank represents the gene which is not significantly dysexpressed in cancer.

The remaining 236 (23.4%) DELs are altered in more than two cancer types (Supplementary Table S4). Previous studies indicated that lncRNAs differentially expressed in multiple cancer types may have conserved oncogenic or tumor suppressor roles^42^. Thus, we defined the 236 DELs as onco-lncRNAs in our study. Among all onco-lncRNAs, there were only 9 DELs dysregulated in all the four cancer types: CTD-2047H16.2, CTD-2517M22.14, CTD-2574D22.3, FGF14-AS2, PVT1, RP11-196G18.22, RP11-346D14.1, RP11-498C9.4 and RP11-510N19.5. Majority of the 236 DELs were missed in earlier studies (Supplementary Table S5). Only 11 onco-lncRNAs were reported to have a bearing on tumorigenesis previously^42–52^ (Fig. 3), two of which (PVT1 and MEG3) were confirmed by conclusive evidences as cancer-associated lncRNAs in multiple cancers^43, 44^. The other nine known cancer-associated lncRNAs were only studied in one cancer type and there have been no experimental evidences and clinic data to support their associations with multiple cancers. In contrast, we also found 2,017 PCGs significantly altered in more than two cancer types (Supplementary Table S6), in which 92 genes were reported as oncogenes in COSMIC database (https://cancer.sanger.ac.uk/census) (Supplementary Table S7), for example, MYC, NOTCH1 and MET.

To gain insight into the associations between the onco-lncRNAs and multiple cancers, we did a survival analysis through an online tool Kaplan-Meier Plotter, which contains a large number of microarray datasets of breast cancer, lung cancer, gastric cancer and ovarian cancer^53–56^. We chose three known cancer-associated lncRNAs (ADAMTS9-AS2, FGF14-AS2 and PCAT19) whose Affymetrix id can be found in this tool to perform the survival analysis over the four cancer types included in the Kaplan-Meier Plotter (Supplementary Fig. S1). For all the three lncRNAs, the survival time is significantly separated between high-expression groups and low-expression ones in all the four tumor types (p ≤ 0.05). And overexpression of the three lncRNAs exhibits a good prognostic effect in all the cancer types, except for FGF14-AS2 whose low expression is good for the survival in the gastric and ovarian cancer. Although the three lncRNAs were identified as cancer-related by previous studies on single cancer types^45, 46, 51^, our analysis has revealed for the first time that their expression levels are significantly associated with clinical prognosis across multiple cancers. The results imply that the three lncRNAs may play important roles in multiple cancers, which could also provide support for the other onco-lncRNAs identified here being important in multiple cancers.

### Module-based functional characterization of onco-lncRNAs with co-expression network analysis

In order to explore potential functions of the 236 onco-lncRNAs, we used WGCNA to construct a co-expression network based on their normalized expression data of all the 236 onco-lncRNAs and 6,316 PCGs whose expression profile are highly correlated with at least five onco-lncRNAs (see Materials and Methods). Finally, we got 18 modules with sizes ranging from 34 to 1,463 genes, in which the number of onco-lncRNAs varies between 0 and 67 (Supplementary Fig. S2 and Supplementary Table S4). We took the first principal component as a module eigengene and used it to represent the overall expression profile of a module^18^, as shown in Supplementary Fig. S3. We obtained the variation of the eigengene between the normal tissues and the tumor ones by one-way analysis of variance (ANOVA) with FDR corrected p-value. The p-value cutoff was set to be 0.0001. Consequently, 12 modules containing the onco-lncRNAs were selected for downstream analysis. The details of the 12 modules are listed in Table 1.

**Table 1:** Overview of 12 cancer-associated modules.

Module	PCGs’ counts	Onco-lncRNAs’ counts	Known-function lncRNAs	Module Cancer Pvalue	Functional category
greenyellow	178	4	—	5.6 × 10^−14^	cell adhesion
tan	118	3	—	1.5 × 10^−10^	cell cycle
black	242	4	PKI55	6.5 × 10^−9^	signal transduction
cyan	99	8	FENDRR, MIR22HG, DIO3OS, PCAT19	8 × 10^−9^	response to immune activity and stimulus
yellow	441	12	FGF14-AS2	1.2 × 10^−8^	response to immune activity and stimulus
brown	844	67	—	4.0 × 10^−7^	genomic stability
lightgreen	30	4	—	1.4 × 10^−6^	cell cycle
blue	1402	41	PVT1, PCAT6, TINCR, TARID, MIR210HG, MIR503HG	1.91 × 10^−5^	signal transduction
red	288	16	ADAMTS9-AS2, MIR143HG	3.4 × 10^−5^	cell adhesion
green	308	7	—	3.7 × 10^−5^	response to immune activity and stimulus
salmon	109	5	—	3.7 × 10^−5^	cell cycle
magenta	182	29	—	9.0 × 10^−5^	morphogenesis

**Figure 4:**
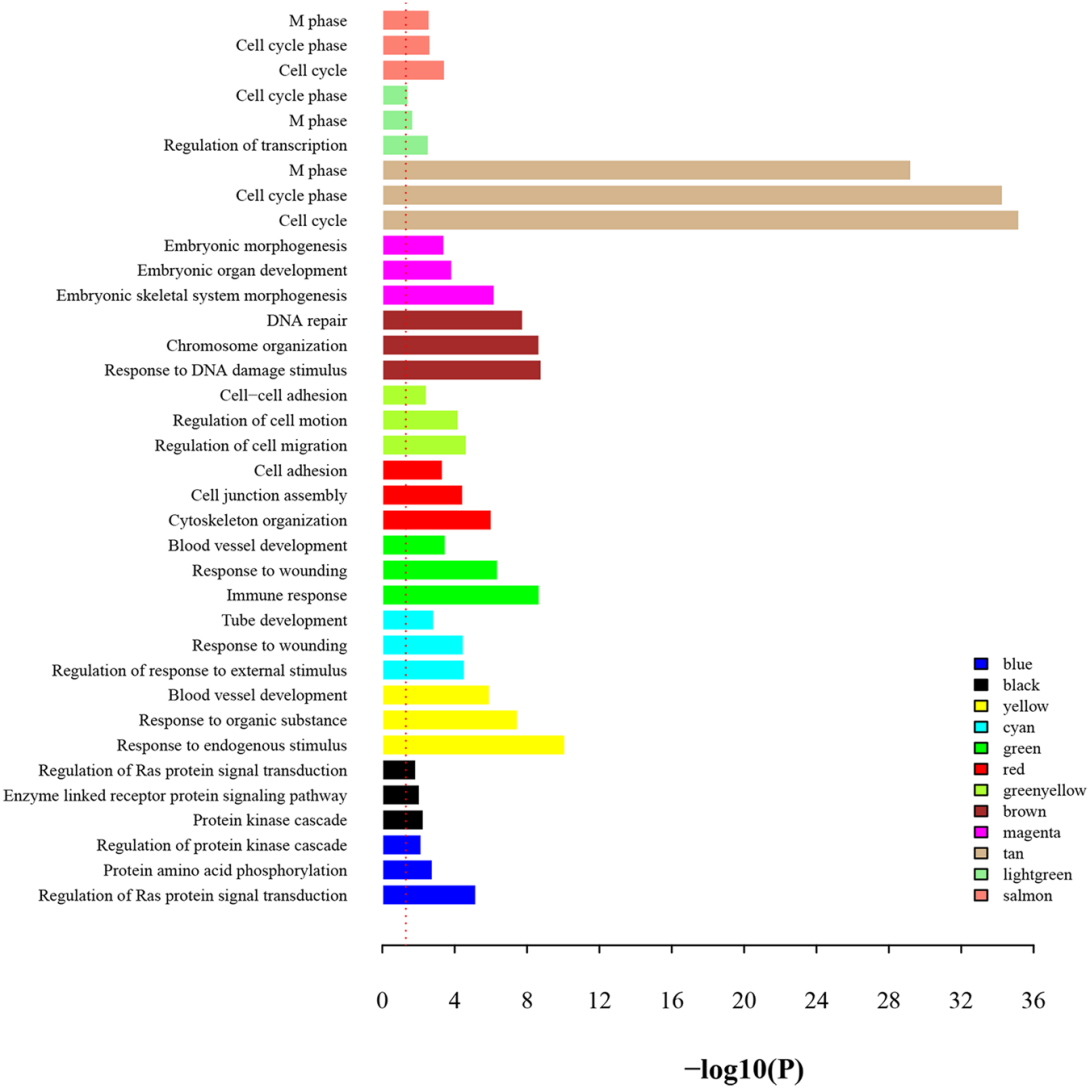
Barplot of representative GO biological process (BP) terms of 12 modules. Three representative GO BP terms were chosen from top 10 significant terms for each module. The y-axis depicts names of BP terms, and the x-axis depicts −log10 (P-value). Bar color denotes the module color. The red dotted line denotes p-value of 0.05.

To further determine the biological functions of the onco-lncRNAs in the 12 modules, DAVID^32^ was used to mine the modules’ biological significance including GO biological process (BP) terms and KEGG pathways. Supplementary Table S8 lists all significantly enriched GO BP terms (p ≤ 0.05) for each module. Figure 4 displays three representative terms for each module. Table 2 lists the significant KEGG pathways with p ≤ 0.05 and gene counts ≥5. According to major biological processes, the 12 modules were parceled out in the following six sections.

**Table 2:** Significant KEGG pathways (P ≤ 0.05, counts ≥ 5) associated with 12 cancer-associated modules.

Module	Entry	Name	Count	P Value
brown	hsa03040	Spliceosome	11	2.8 × 10^−4^
	hsa03430	Mismatch repair	5	1.2 × 10^−3^
blue	hsa04370	VEGF signaling pathway	14	2.3 × 10^−4^
	hsa04666	Fc gamma R-mediated phagocytosis	15	7.6 × 10^−4^
	hsa04330	Notch signaling pathway	10	1.0 × 10^−3^
	hsa04070	Phosphatidylinositol signaling system	12	2.6 × 10^−3^
	hsa04664	Fc epsilon RI signaling pathway	12	3.9 × 10^−3^
	hsa04660	T cell receptor signaling pathway	14	7.1 × 10^−3^
	hsa04010	MAPK signaling pathway	25	1.4 × 10^−2^
	hsa04662	B cell receptor signaling pathway	10	2.4 × 10^−2^
	hsa05222	Small cell lung cancer	10	4.5 × 10^−2^
black	hsa05220	Chronic myeloid leukemia	5	7.4 × 10^−3^
	hsa04722	Neurotrophin signaling pathway	6	8.9 × 10^−3^
	hsa05200	Pathways in cancer	9	1.9 × 10^−2^
	hsa04062	Chemokine signaling pathway	6	4.4 × 10^−2^
	hsa04010	MAPK signaling pathway	7	5.0 × 10^−2^
yellow	hsa04910	Insulin signaling pathway	12	1.4 × 10^−3^
	hsa05218	Melanoma	7	1.5 × 10^−2^
	hsa05200	Pathways in cancer	17	2.2 × 10^−2^
	hsa04062	Chemokine signaling pathway	11	3.8 × 10^−2^
	hsa03320	PPAR signaling pathway	6	4.5 × 10^−2^
cyan	hsa04610	Complement and coagulation cascades	5	1.5 × 10^−3^
	hsa04010	MAPK signaling pathway	6	4.2 × 10^−2^
green	hsa04610	Complement and coagulation cascades	9	6.1 × 10^−5^
	hsa05332	Graft-versus-host disease	6	9.9 × 10^−4^
	hsa04650	Natural killer cell mediated cytotoxicity	10	1.3 × 10^−3^
	hsa05416	Viral myocarditis	6	1.3 × 10^−2^
	hsa04621	NOD-like receptor signaling pathway	5	3.5 × 10^−2^
red	hsa04510	Focal adhesion	17	1.9 × 10^−7^
	hsa04270	Vascular smooth muscle contraction	13	3.2 × 10^−7^
	hsa04810	Regulation of actin cytoskeleton	15	1.3 × 10^−5^
	hsa04020	Calcium signaling pathway	12	1.8 × 10^−4^
	hsa04260	Cardiac muscle contraction	8	3.3 × 10^−4^
	hsa04530	Tight junction	10	4.2 × 10^−4^
	hsa04512	ECM-receptor interaction	6	1.4 × 10^−2^
	hsa04670	Leukocyte transendothelial migration	7	1.5 × 10^−2^
	hsa04010	MAPK signaling pathway	10	3.8 × 10^−2^
tan	hsa03030	DNA replication	6	1.2 × 10^−6^
	hsa04110	Cell cycle	7	4.7 × 10^−5^

#### Modules associated with signal transduction

The blue module contains 41 onco-lncRNAs and 1,402 PCGs. As observed from Fig. 4 and Supplementary Table S8, PCGs in this module are significantly enriched in processes like regulation of Ras protein signal transductions, protein amino acid phosphorylation and protein kinase cascade. The significant KEGG pathways included several signaling pathways relevant to cancer, like VEGF signaling pathway, Notch signaling pathway and MAPK signaling pathway (Table 2). VEGF signaling pathway can mediate proliferation and migration of endothelial cells and promote their survival and vascular permeability. Inappropriate regulation of VEGF was observed to have effects on cell migration and survival in cancers^57^. The black module contains four onco-lncRNAs and their enriched functions are similar to the blue module, as reflected by Fig. 4, Table 2 and Supplementary Table S8.

In the two modules, only two onco-lncRNAs (PVT1 and PCAT6 in the blue module), which exhibit connections to the genes with topological overlaps (*w*) greater than or equal to 0.05 in the network, were reported to have associations with cancers^43, 47^. Supplementary Fig. S4a,b show distributions of their expression profiles in the four cancers. It can be seen that PVT1 displays significant overexpression in all the four tumor tissues while PCAT6 is significantly overexpressed in three types of tumor tissue except for PRC. In addition, the two lncRNAs are highly connected (*w* ≥ 0.05) to genes showing functions in the signaling cascade (Supplementary Fig. S5). For example, HGS is a positive regulator of VEGF and insulin signaling^58^, and the absence of KCTD13 is likely to lead to hyperactivation of the RhoA signaling pathway^59^.

PVT1, as a candidate oncogene, was revealed to be related with cell proliferation and tumor progression in many neoplastic diseases^43^. Tseng^60^ indicated that high MYC protein levels in 8q24-amplified human cancer cells require gain of PVT1 expression to suppress phosphorylation of T58, in turn protecting MYC protein from degradation. Indeed, our functional analysis of the blue module shows that phosphorylation is a significant functional term (Supplementary Table S8) and the nodes connected to PVT1 contain a MYC-related gene, EHMT1 (PCC = 0.72) (Supplementary Fig. S5), which is part of the E2F6 complex involved in silencing of MYC-responsive genes and G0/G1 cell cycle transition^61^. The consistency between our computational analysis and the earlier observations confirms reliability of our predicted results. Thus, it is reasonable to speculate that the onco-lncRNAs clustered in the blue and black modules very likely play important roles in many signaling circuits, in turn influencing the cancer progress.

#### Modules associated with response to immune activity and stimulus

The yellow module, which contains 12 onco-lncRNAs and 441 PCGs, shows functional enrichment in response to endogenous stimulus, organic substances, and blood vessel development (Fig. 4, Supplementary Table S8). Pathway analysis further reveals that the genes in the module are enriched in some signal transduction pathways like insulin signaling pathway and chemokine signaling pathway, and also in some cancer-associated pathways like melanoma (Table 2). The cyan and green modules, which contain 8 and 7 onco-lncRNAs, respectively, share the BP terms about vascular system with the yellow module. In addition, the cyan and green modules also show enrichment in immune response, and inflammatory regulation (Fig. 4, Supplementary Table S8).

The lncRNA FGF14-AS2 in the yellow module, was reported to be a breast-cancer-associated lncRNA and may act as a tumor suppressor^45^. This gene is observed to be down-regulated in the tumor tissues of all the four cancer types, as evidenced by Supplementary Fig. S4c. Some protein-coding genes connected to it (*w* ≥ 0.05) take roles in the function of response to stimulus (Supplementary Fig. S6). In addition, FGF14-AS2 shows a high correlation with a famous cancer-associated gene VEGFB (PCC = 0.70), which is a member of vascular endothelial growth factor family and dysregulated in many cancers^62^.

The cyan module contains four lncRNAs (MIR22HG, PCAT19, FENDRR and DIO3OS) whose functions were characterized^51, 63–65^. Although there have been no studies to provide evidence for their associations with cancers, in our study, they exhibit an accordant down-expression pattern in the cancer tissues when they were significantly dys-expressed (Supplementary Fig. S4d–g). In addition, some protein-coding genes connected to the four lncRNAs (*w* ≥ 0.05) are also associated with the response to stimulus (Supplementary Fig. S7). For MIR22HG, two studies revealed its roles in chemical stress responses^63, 66^. In our study, MIR22HG shows a positive correlation with IL6 (PCC = 0.68), which was implicated in inflammation, hematopoiesis and carcinogenesis^67^. In addition, MIR22HG also exhibits a positive correlation with CCL2 (PCC = 0.80), which was reported to be involved in immunoregulatory and inflammatory processes of multiple cancers^68^. FENDRR presents a strong positive correlation with FOXF1 (PCC = 0.85), consistent with a previous study^42^. FOXF1 was indicated to play roles in response to wounding and chemical stimulus, and be important in human development and tissue repair^69^.

The observations above indicate that the onco-lncRNAs in these modules may contribute to functions associated with the response to stimulus and immunity. Previous researches proposed that the immune response is an attempt by the immune system to eradicate tumor, and could enhance tumorigenesis and progression^70^.

#### Modules associated with cell adhesion

There are 16 onco-lncRNAs and 288 PCGs in the red module. Our analysis shows that the genes in this module are significantly enriched in BP terms like cytoskeleton organization, cell junction assembly and cell adhesion (Fig. 4 and Supplementary Table S8). Further analysis indicates that they show enrichments in pathways associated with focal adhesion, regulation of actin cytoskeleton, tight junction and ECM-receptor interaction (Table 2), consistent with the observations from BP terms. Similarly, genes in the greenyellow module containing four onco-lncRNAs also show significant enrichment in the functions involved in cell migration and cell adhesion (Fig. 4 and Supplementary Table S8).

There are two reported lncRNAs (ADAMTS9-AS2 and MIR143HG) in the red module. MIR143HG, as a cardiac mesoderm enhancer-associated non-coding RNA^71^, is observed to be significantly down-regulated in BLC and EBC (Supplementary Fig. S4i). ADAMTS9-AS2 was reported to be significantly down-regulated in glioma tumor tissues and its overexpression would result in significant inhibition of glioma cell migration^46^. In our study, ADAMTS9-AS2 is significantly down-expressed in BLC, ADC and EBC (Supplementary Fig. S4h). Furthermore, it can be seen from Supplementary Fig. S8 that most genes directly connected to ADAMTS9-AS2 (*w* ≥ 0.05) also participate in the functions like cell adhesion and migration, for example, NCAM1^72^ (PCC = 0.70) and PALLD^73^ (PCC = 0.70). Similar to ADAMTS9-AS2, MIR143HG also exhibits connections with genes involved in cell adhesion like TGFB1I1^74^ (PCC = 0.80).

Thus, it can be conjectured that the onco-lncRNAs in the red and greenyellow modules most possibly participate in maintaining cell shape and changing attachment to other cells or extracellular matrix. The dysregulation in these functions can promote migration of cancer cells, leading to local invasion and distant metastasis^70^.

#### Module associated with genomic stability

The brown module contains the most onco-lncRNAs (67) among all the modules and 844 PCGs. However, none of the 67 onco-lncRNAs have been reported to have associations with cancers. The genes in this module significantly contribute to response to DNA damage stimulus, DNA repair and chromosome organization processes (Fig. 4 and Supplementary Table S8). The BP terms are associated with functions of maintaining genomic stability and their disorders were revealed to be connected with predisposition to cancer^75, 76^. In addition, the genes are mainly enriched in spliceosome pathway and base excision repair pathway (Table 2), which are also associated with regulation of genomic stability. Therefore, it is reasonable to infer that the 67 onco-lncRNAs may play roles in maintaining genomic stability under normal circumstances and their imbalances could promote the cancerization progress.

#### Module associated with morphogenesis

The magenta module contains 29 onco-lncRNAs and 182 PCGs. None of the 29 onco-lncRNAs have been reported to be correlated with cancers. The enrichment analysis for BP terms reveals that most significant terms are involved in embryogenesis progresses, like embryonic skeletal system morphogenesis, embryonic organ morphogenesis and embryonic morphogenesis (Fig. 4 and Supplementary Table S8). Some evidences indicated that genes with functions involved in the embryo development stage would play a role in carcinogenesis^77^. Thus, it can be inferred that the onco-lncRNAs in the module magenta may be associated with steps of the embryonic morphogenesis and would advance carcinoma to progress to higher pathological grades^78–80^.

#### Modules associated with cell cycle

The tan, lightgreen and salmon modules contain three, four and five onco-lncRNAs, and the number of PCGs are 118, 30 and 109, respectively. The enrichment function terms in the three modules are all involved in cell cycles (Fig. 4 and Supplementary Table S8). As accepted, the most fundamental trait of cancer cells is to sustain proliferation and the abnormality of the cell cycle has been considered to be a common feature of cancers^81^. Thus, it can be speculated that the 12 onco-lncRNAs in the three modules could play an important role in cancers through dysregulation of the cell cycle.

In summary, the module analysis above indicates that the functions of onco-lncRNAs in the 12 modules are involved in the biological roles relevant to malignancies. Compared to the previous observations^21, 31^, some new functions of the lncRNAs like the morphogenesis and immune regulation are revealed by our work.

### Hub-based analysis

**Figure 5:**
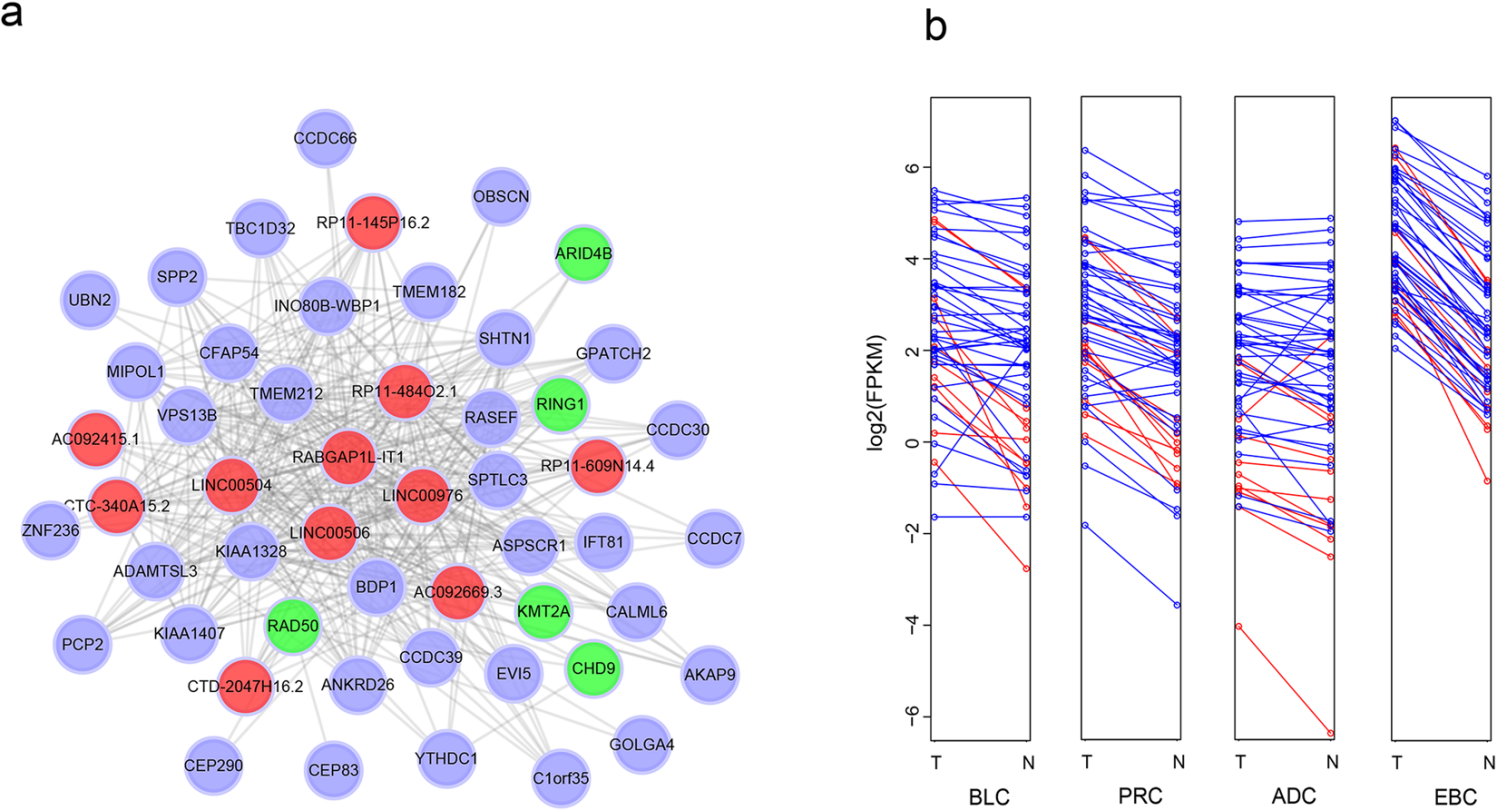
Network and expression levels of 51 top hub genes in brown module. (**a**) Cytoscape network visualization of 51 genes, in which only edges with weight (*w*) above a threshold of 0.2 are displayed. The red nodes denote lncRNA genes. The green and blue nodes both denote protein-coding genes, but the green color stands for genes with function in maintaining genomic stability. (**b**) The expression levels of 51 genes in each cancer type. The y axis represents the average FPKM value of samples in each group. And the blue and red colors denote protein-coding genes and onco-lncRNAs, respectively.

Highly connected hub nodes are central to the network’s architecture^18^ and some studies suggested that genes more centralized in the network are more likely to be key drivers to proper cellular function than peripheral genes^82^. As observed above, the brown module has the most number of onco-lncRNAs and its eigengene shows a significant difference between the tumor and normal samples. Taking the brown module as an example, we further identify its intramodular hub genes. Although the filter for genes used in building the network may lead the onco-lncRNA connectivity towards higher values, we could obtain more important nodes from the onco-lncRNAs through identifying hub nodes. We selected the 5% of nodes (51/911) with the highest connectivity as hub genes from the brown module, which contain 11 onco-lncRNAs and 40 PCGs. Figure 5a is a network of these hub genes, which only displays connections with *w* above a threshold of 0.2. It can be seen that the 11 onco-lncRNAs exhibit high connectivity with neighboring genes (RAD50, CHD9, KMT2A, ARID4B and RING1) whose functions are involved in maintaining genome stability. Especially, RAD50, KMT2A and ARID4B were revealed as biomarkers in a broad range of human malignancies^83, 84^. In addition, the 51 hub genes are overexpressed in the tumor tissues of each cancer type (Fig. 5b). The observation further indicates that the 11 onco-lncRNAs may play important roles in the regulation of genome stability in tumor biology. Meanwhile, their higher connectivity than other onco-lncRNAs in brown module suggests that they may play more crucial roles in the biological functions and the development of cancers than the other onco-lncRNAs in this module.

## Conclusion

Although accumulating studies have indicated that the lncRNAs play important roles in tumor progress, the functions for most lncRNAs have not been unraveled, in particular for potential oncogenic lncRNAs across multiple cancers. In this study, we mainly utilized the gene co-expression network to study the functions of the onco-lncRNAs for the four solid cancer types.

The 236 onco-lncRNAs altered in multiple cancers were identified, majority of which were unreported previously to have associations with cancers. Our co-expression network and function enrichment analysis indicate that the onco-lncRNAs should play carcinogenic roles in the most fundamental functions involved in regulating proliferation and genome stability, providing further supports for the previous observations^31^. More importantly, our results reveal that the onco-lncRNAs are also associated with some biological capabilities implicated in the processes related to major hallmarks of cancers, like cell adhesion and motility, morphogenesis, immune and inflammatory response.

Overall, our study is the first time to use WGCNA approach to investigate the functions of the lncRNAs across multiple cancers based on RNA-seq data. Although the biological importance of the unreported onco-lncRNAs need further evaluation by experiments, our study proposed a facile yet efficient strategy to identify important lncRNAs associated with cancers and predict their potential functional roles, which may guide subsequently experimental studies.

## Materials and Methods

### RNA-seq datasets

Raw fastaq files of RNA-seq datasets for the four cancer types were downloaded from the European Nucleotide Archive (http://www.ebi.ac.uk/ena), including bladder cancer (SRP018008)^33^, prostate cancer (ERP000550)^34^, lung adenocarcinoma (SRP012656)^35^ and estrogen receptor positive (ER+) breast cancer (SRP042620)^36^ (Supplementary Table S1). In each dataset, we only choose those tumor samples with matched adjacent normal samples. Finally, we obtained 132 samples, of which at least 11 sample pairs for each cancer type, to analyze downstream.

### Raw reads alignment and expression quantification

After the sequence quality control on the raw sequence data by fastqc v0.11.5 (http://www.bioinformatics.babraham.ac.uk/projects/fastqc/), raw reads were mapped back to the reference genome GRCh38.p3 by TopHat v2.0.13^85, 86^, and we used the GENCODE v23 gtf file (ftp://ftp.sanger.ac.uk/pub/gencode/Gencode_human/release_23/gencode.v23.annotation.gtf.gz) as annotation file, which contains 15,931 lncRNA genes. Then we used Cufflinks v2.2.1^86, 87^ for the gene assembly and quantification. We obtained the gene expression levels by summarizing the FPKM value (Fragment Per Kilobase per Million mapped reads). In order to minimize the false positive and maintain a high number of differential expressed genes in downstream analysis, we only kept the expressed genes in terms of the criterion of FPKM ≥ 1 in more than 80% of the normal samples or 80% of the tumor samples for each cancer type according to Supplementary Fig. S9.

### Differential expression analysis

We performed differential expression analysis on each cancer, based on BAM files derived from TopHat. DESeq2 v1.12.4^88^ was used to test differential expression between the tumor and normal samples. A gene is defined as a differentially expressed gene between the normal sample and tumor one when the FDR adjusted p value is less than 0.01 (FDR ≤ 0.01) and the fold change (FC) is at least 2 times higher or lower (|log2FC| ≥ 1).

### Co-expression network construction of onco-lncRNAs

We defined the lncRNAs significantly altered in more than two cancer types as onco-lncRNAs. In order to predict their functions, WGCNA v1.51^18^ was used to construct a co-expression network between the onco-lncRNAs and their “closely correlated” PCGs, based on the signed Pearson Correlation Coefficient (PCC) between their normalized expression levels as provided by Cuffnorm^86^. A PCG is defined to be “closely correlated” with the onco-lncRNAs when its absolute values of Pearson correlation coefficients with more than 5 onco-lncRNAs are equal or greater than 0.5. Consequently, 6,316 correlated-PCGs were obtained. We then calculated a correlation matrix containing the absolute values of pairwise Pearson correlations among all the onco-lncRNAs and the correlated PCGs for the samples under study. In order to achieve a scale-free topology, we set β = 9 in terms of Supplementary Fig. S10 and converted the pairwise correlation into an adjacency matrix of connection strengths through soft-thresholding approach (connection strength = |correlation|^β^). A dissimilarity matrix based on topological overlap measure (TOM) was used to identify gene modules through a dynamic tree-cutting algorithm^18^. All modules were assigned to the corresponding color. The module eigengene was used to represent each module, which was calculated by the first principal component. Using the module eigengenes, Module-Cancer relationships were estimated by one-way ANOVA with FDR corrected p-value between the module eigengene and the tissue type (normal and tumor). Then we selected 12 significantly cancer-associated modules (p-value ≤ 0.0001) for the downstream analysis. We also analyzed the hub genes of the brown module, which were derived from top 5% genes with the highest connectivity.

### Functional enrichment analysis of onco-lncRNA-containing modules

We used DAVID v6.7^32^ (https://david-d.ncifcrf.gov/) to perform the functional enrichment analysis for each module. The tool computes a modified Fisher exact test p-value. In the main text, we only show the three representative terms from top 10 most significantly GO BP terms of each module. But, all significant terms (p ≤ 0.05) are listed in Supplementary Table S8. In addition, we only concerned significant KEGG pathways with p-value ≤ 0.05 and the number of enriched genes ≥ 5 (Table 2).

### Statistical analysis and visualization

Statistical analysis was performed using R-3.3.1. Most of the visualizations were also presented by R, except for the survival analysis and the network visualization, where the Kaplan-Meier Plotter (http://kmplot.com/) and Cytoscape v3.3.0 (http://www.cytoscape.org/) tools were used. For the survival analysis, we chose recommended parameters from the web server to analyze the association between a queried gene and the survival time. Samples were grouped according to the median expression of the selected gene. All the survival curves denote overall survival (OS).

## Electronic supplementary material

Supplementary information
