# Supplementary material for: Exploring functions of long noncoding RNAs across multiple cancers through co-expression network
Source: Sci Rep. 2017 Apr 7;7:754. doi: 10.1038/s41598-017-00856-8 (PMC5429718; doi:10.1038/s41598-017-00856-8)
Supplement: Supplementary file 1 — Supplementary information [file 41598_2017_856_MOESM1_ESM.pdf]

# Exploring functions of long noncoding RNAs across multiple cancers through co-expression network

Suqing Li[1], Bin Li[2], Yuanting Zheng[2, 3], Menglong Li[1], Leming Shi[2, 3]* and Xuemei Pu[1]*

[1]College of Chemistry, Sichuan University, Chengdu 610064, China.

[2]Center for Pharmacogenomics, School of Life Sciences, and State Key Laboratory of Genetic Engineering and MOE Key Laboratory of Contemporary Anthropology, Fudan University, Shanghai 201203, China.

[3]Collaborative Innovation Center for Genetics and Development, Fudan University, Shanghai 200438, China.

*To whom correspondence should be addressed (lemingshi@fudan.edu.cn and xmpuscu@scu.edu.cn).

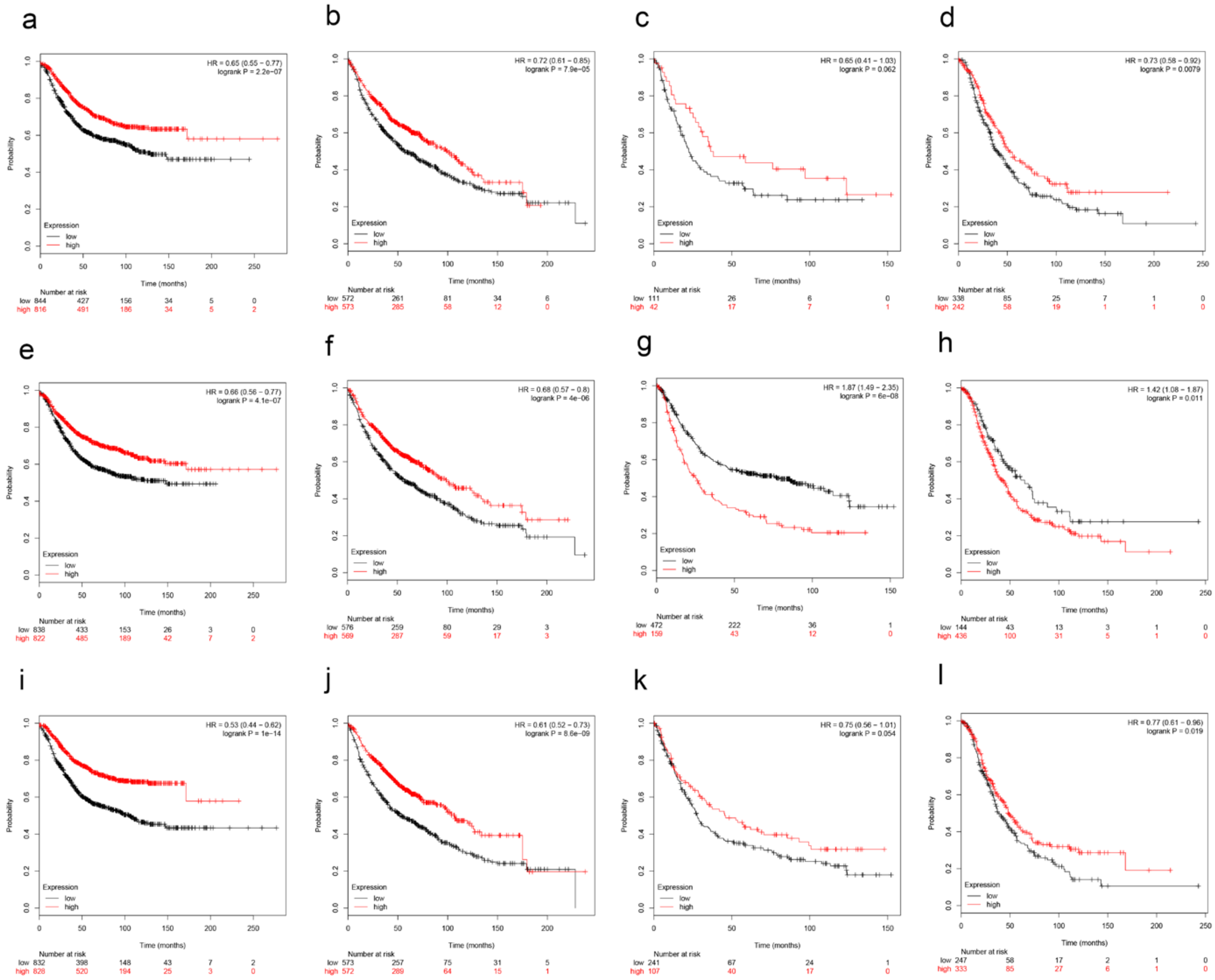
a
HR = 0.65 (0.55 − 0.77)
logrank P = 2.2e−07
Probability
Time (months)
Expression
low
high
Number at risk
low 844 427 156 34 5 0
high 816 491 186 34 5 2
b
HR = 0.72 (0.61 − 0.85)
logrank P = 7.9e−05
Probability
Time (months)
Expression
low
high
Number at risk
low 572 261 81 34 6
high 573 285 58 12 0
c
HR = 0.65 (0.41 − 1.03)
logrank P = 0.062
Probability
Time (months)
Expression
low
high
Number at risk
low 111 26 6 0
high 42 17 7 1
d
HR = 0.73 (0.58 − 0.92)
logrank P = 0.0079
Probability
Time (months)
Expression
low
high
Number at risk
low 338 85 25 7 1 0
high 242 58 19 1 1 0
e
HR = 0.66 (0.56 − 0.77)
logrank P = 4.1e−07
Probability
Time (months)
Expression
low
high
Number at risk
low 838 433 153 26 3 0
high 822 485 189 42 7 2
f
HR = 0.68 (0.57 − 0.8)
logrank P = 4e−06
Probability
Time (months)
Expression
low
high
Number at risk
low 576 259 80 29 3
high 569 287 59 17 3
g
HR = 1.87 (1.49 − 2.35)
logrank P = 6e−08
Probability
Time (months)
Expression
low
high
Number at risk
low 472 222 36 1
high 159 43 12 0
h
HR = 1.42 (1.08 − 1.87)
logrank P = 0.011
Probability
Time (months)
Expression
low
high
Number at risk
low 144 43 13 3 1 0
high 436 100 31 5 1 0
i
HR = 0.53 (0.44 − 0.62)
logrank P = 1e−14
Probability
Time (months)
Expression
low
high
Number at risk
low 832 398 148 43 7 2
high 828 520 194 25 3 0
j
HR = 0.61 (0.52 − 0.73)
logrank P = 8.6e−09
Probability
Time (months)
Expression
low
high
Number at risk
low 573 257 75 31 5
high 572 289 64 15 1
k
HR = 0.75 (0.56 − 1.01)
logrank P = 0.054
Probability
Time (months)
Expression
low
high
Number at risk
low 241 67 24 1
high 107 40 17 0
l
HR = 0.77 (0.61 − 0.96)
logrank P = 0.019
Probability
Time (months)
Expression
low
high
Number at risk
low 247 58 17 2 1 0
high 333 85 27 6 1 0

**Supplementary Figure S1 | Survival analysis of low-expression and high-expression for each lncRNA using online tool Kaplan-Meier Plotter. (a**, **b**, **c, d**) The Kaplan-Meier plots of ADAMTS9-AS2 in the breast cancer, lung cancer, gastric cancer and ovarian cancer. (**e**, **f, g, h)** The Kaplan-Meier plots of FGF14-AS2 in the breast cancer, lung cancer, gastric cancer and ovarian cancer. **(I, j, k, l**) The Kaplan-Meier plots of FGF14-AS2 in the breast cancer, lung cancer, gastric cancer and ovarian cancer. The red line denotes high expression, and the black denotes low expression. The x-axis is time, and the y axis is the proportion of subjects surviving. The digits under each plot represent the number of survival patients at each time point. The overexpression of the three lncRNAs significantly reduces risk of death when compared to their low expressions in 4 cancer types, except for FGF14-AS2 in gastric cancer and ovarian cancer, the low expression of which reduces the death rate.

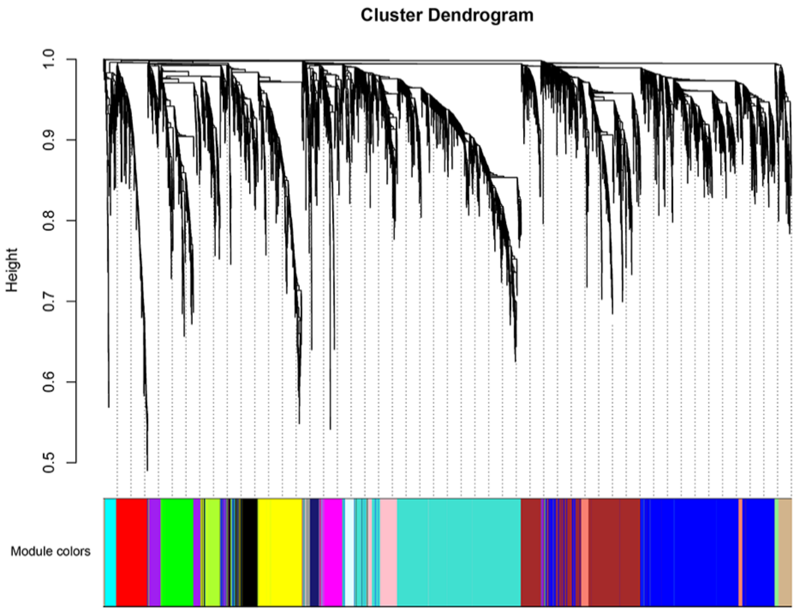


**Supplementary Figure S2 | Modules defined by the weighted correlation network analysis (WGCNA)**. Tree on the top is the clustering dendrogram of genes, and the colorful bands represent modules in this network.

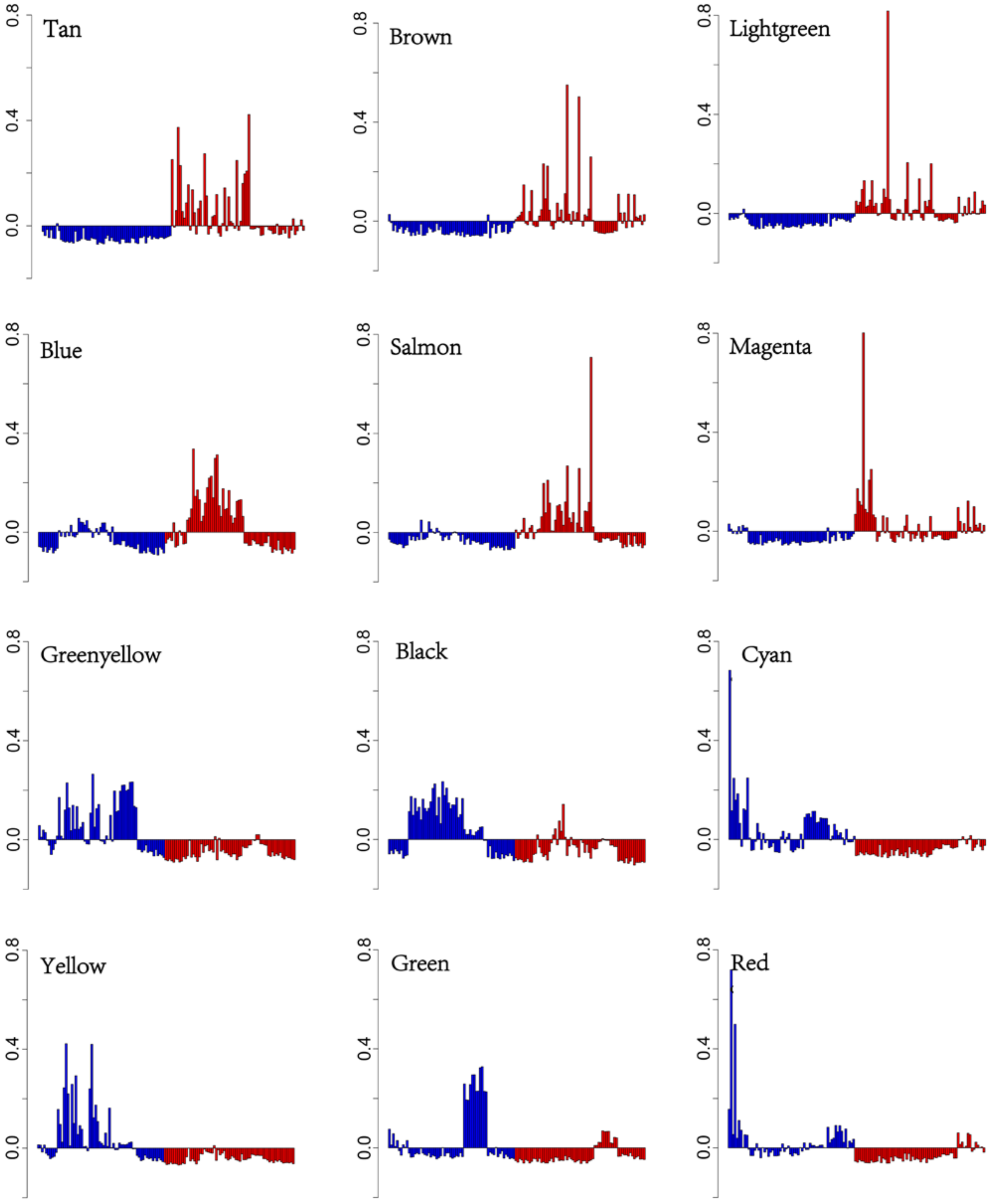


**Supplementary Figure S3 | Module eigengene's profile across all samples.** The x-axis depicts all samples, and the y-axis depicts the expression level of the eigengene. The red bar represents the tumor sample, and the blue denotes the normal sample.

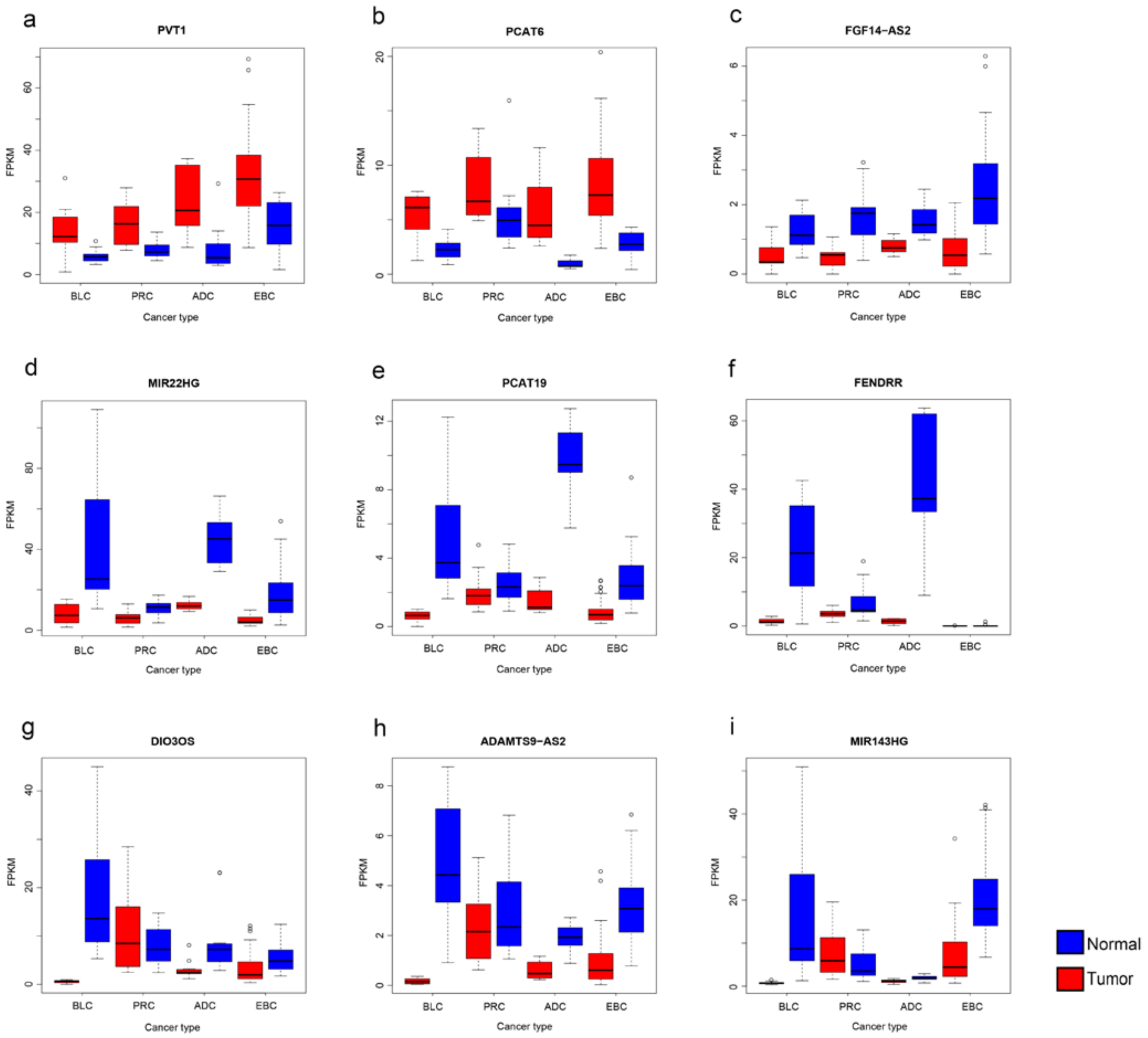


**Supplementary Figure S4 | Expression levels (FPKM) of eight reported lncRNAs,** (**a**) PVT1, (**b**) PCAT6, (**c**) FGF14-AS2. (**d**) MIR22HG, (**e**) PCAT19, (**f**) FENDRR, (**g**) DIO3OS, (**h**) ADAMTS9-AS2, (**i**) MIR143HG. The red box denotes the tumor tissue and the blue is the normal tissue. The x-axis depicts the cancer type.

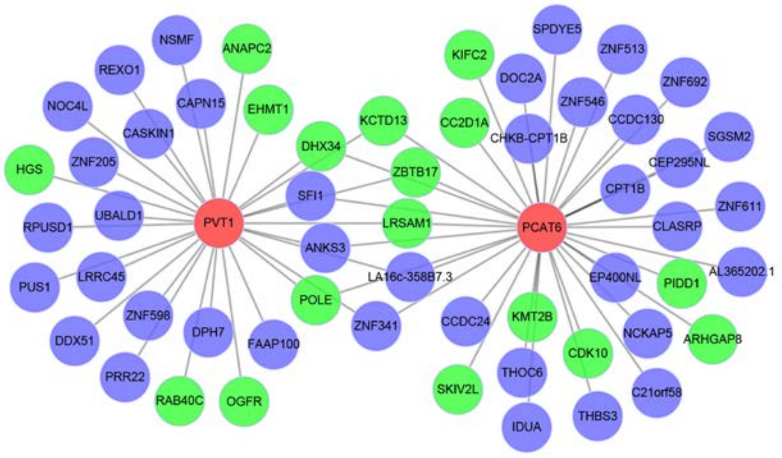


**Supplementary Figure S5 | Functional investigation of genes correlated with PVT1 and PCAT6.** The red nodes denote the two lncRNAs. The green nodes denote genes involved in protein catabolic process and regulation of protein kinase cascade. The blue nodes denote other connected genes.

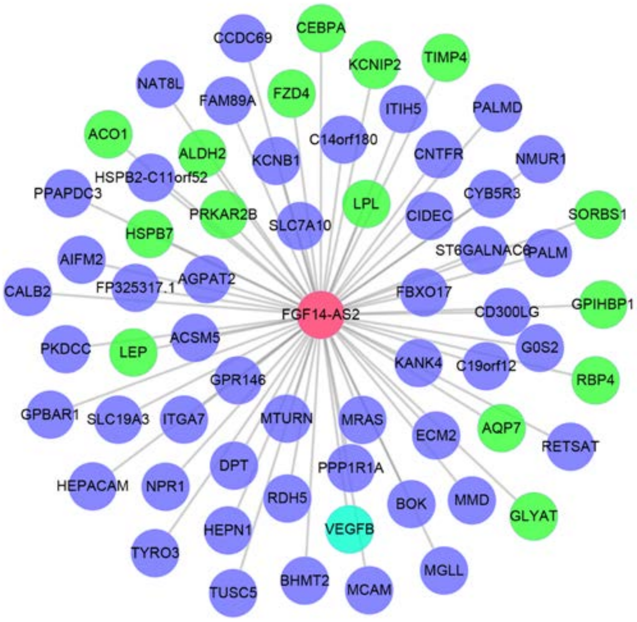


**Supplementary Figure S6 | Functional investigation of genes correlated with FGF14-AS2.** The red nodes denote the FGF14-AS2. The green nodes represent genes involved in response to stimulus. The cyan one is a famous cancer-associated gene VEGFB. The blue nodes denote other connected

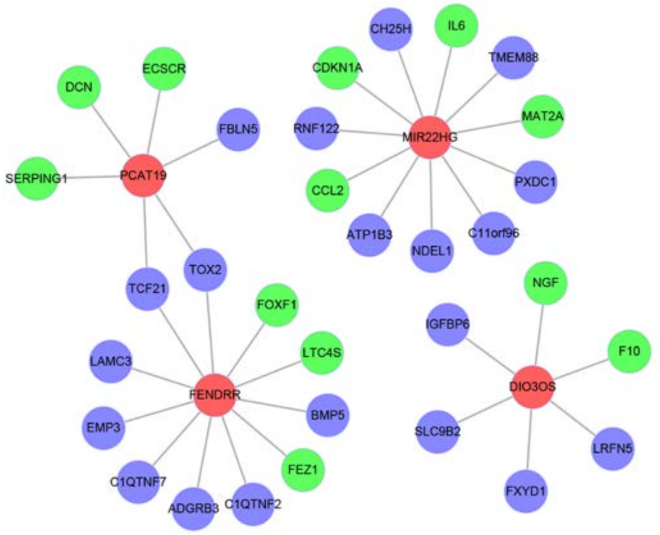


Supplementary Figure S7 | Functional investigation of genes correlated with PCAT19, FENDRR, MIR22HG and DIO3OS. The red nodes denote the four lncRNAs. The green nodes denote genes involved in response to stimulus and inflammation. The blue nodes denote other connected genes.

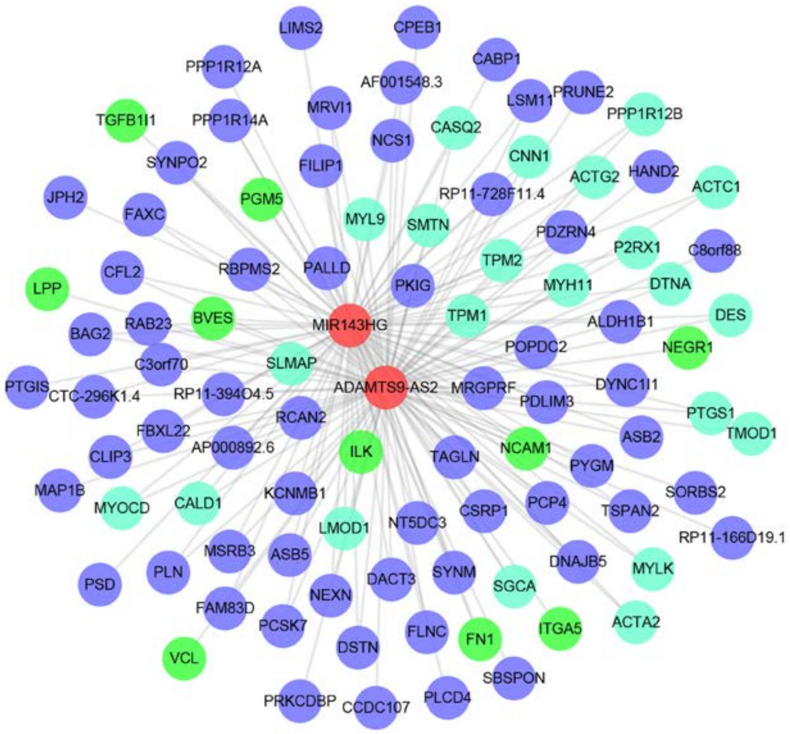


**Supplementary Figure S8 | Functional investigation of genes correlated with MIR143HG and ADAMTS9-AS2.** The red nodes denote the two lncRNAs. The green nodes denote genes involed in cell adhesion. The cyan nodes are genes showing function terms about muscle. The blue nodes denote other connected genes.

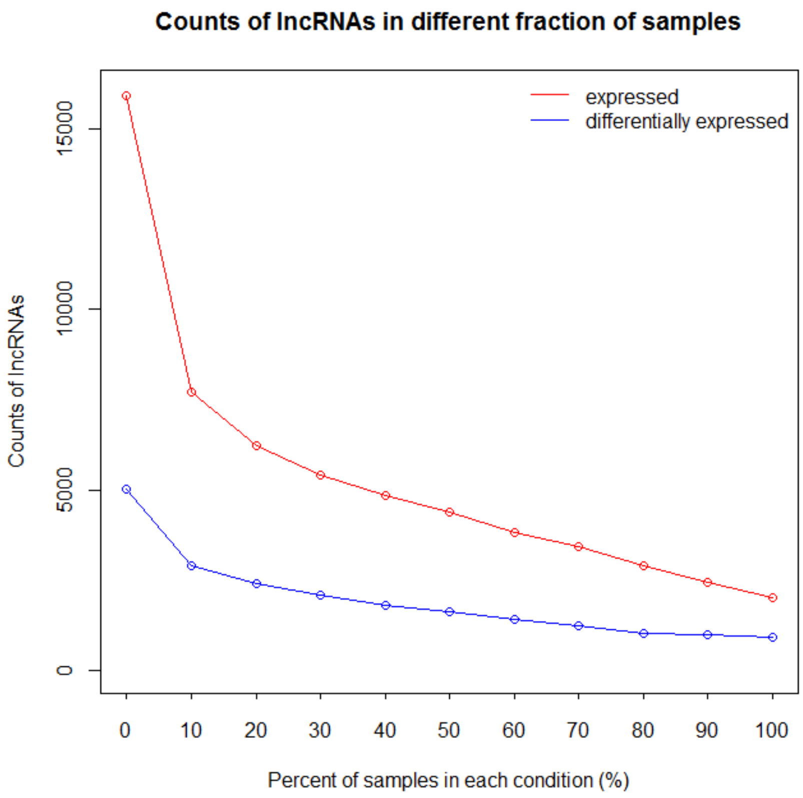


**Supplementary Figure S9 | The number of expressed lncRNAs (red) and differentially expressed lncRNAs (blue) with different fraction of samples.** The x-axis is the different fraction of samples, and the y-axis is the total number of expressed genes. In the work, we used the criterion FPKM $\geq$ 1 to define the expressed levels. The number of expressed lncRNAs (red) decreased continuously with increase of the fraction of samples, which is similar with the number of differentially expressed genes (blue). But the number of differentially expressed genes (blue) becomes stable at 80%. Thus, to minimize the false positive and maintain a high number of differential expressed lncRNAs, we did our filter which requires the expression level of a gene exceed a threshold (FPKM $\geq$ 1) in more than a fraction of samples (80%) in at least one condition in each cancer type.

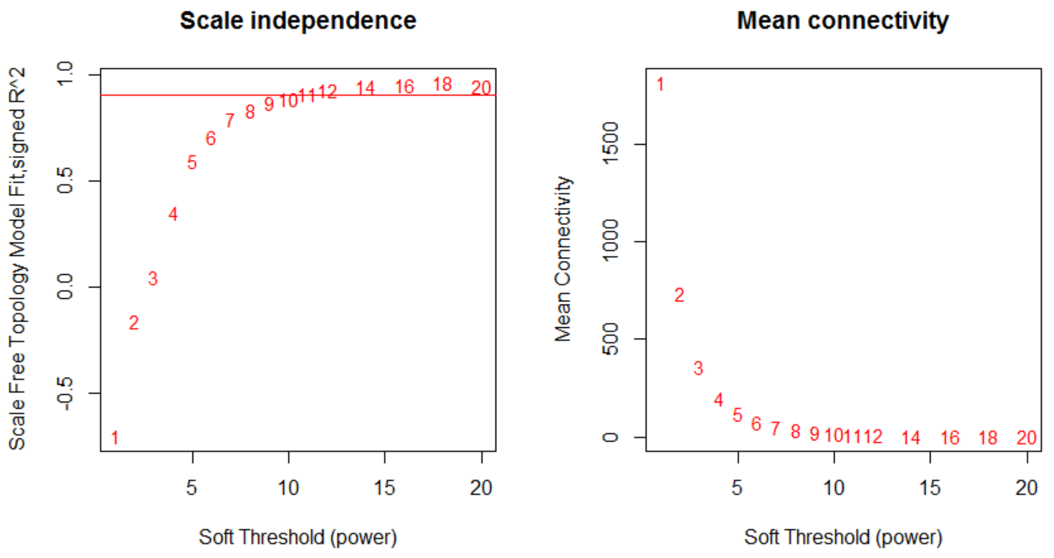


**Supplementary Figure S10 | Analysis of network topology for different soft thresholds**. The left panel shows the scale-free topology fitting index ($R^2$, y-axis) as a function of the soft-thresholding power (x-axis). The right panel displays the mean connectivity (degree, y-axis) as a function of the soft-thresholding power (x-axis). Red Arabic numerals in the panels denote different soft-thresholds. The red line in left panel means $R^2 = 0.9$. There is a trade-off between maximizing scale-free topology model fit ($R^2$) and maintaining a high mean number of connections. Thus, we set $\beta = 9$.

## Supplementary Table S1: RNA-Seq Datasets

| Accession | Study | Cancer type | Samples (pairs) | Library layout | Reads length (bp) |
|---|---|---|---|---|---|
| SRP012656 | A high dimensional deep sequencing study of non-small cell lung adenocarcinoma in never-smoker Korean females | lung adenocarcinoma (ADC) | 12 | paired | 78 |
| SRP042620 | Breast Cancer RNA-seq | ER+ breast cancer (EBC) | 29 | paired | 50 |
| ERP000550 | Complete transcriptomic landscape of prostate cancer in the Chinese population using RNA-seq | Prostate cancer (PRC) | 14 | paired | 90 |
| SRP018008 | Homo sapiens Transcriptome or Gene expression | Bladder cancer (BLC) | 11 | paired | 100 |

**Supplementary Table S2: DELs (differentially expressed lncRNAs) only altered in one cancer type**

**BLA**

| gene_id | gene_name | log2FC | FDR |
|---|---|---|---|
| ENSG00000151303.11 | RP11-96C23.13 | 1.4 | 6.7E-04 |
| ENSG00000153363.12 | LINC00467 | 1.3 | 9.3E-03 |
| ENSG00000166770.10 | ZNF667-AS1 | -3.3 | 3.6E-14 |
| ENSG00000174365.19 | SNHG11 | 1.4 | 2.3E-04 |
| ENSG00000177410.12 | ZFAS1 | 1.8 | 5.6E-03 |
| ENSG00000180139.11 | ACTA2-AS1 | -4.6 | 1.2E-17 |
| ENSG00000197182.12 | MIRLET7BHG | 1.6 | 7.8E-04 |
| ENSG00000203497.2 | PDCD4-AS1 | -1.2 | 7.4E-04 |
| ENSG00000203706.8 | SERTAD4-AS1 | -1.8 | 1.3E-04 |
| ENSG00000204282.4 | TNRC6C-AS1 | 1.1 | 1.8E-03 |
| ENSG00000204528.3 | PSORS1C3 | 1.9 | 7.5E-03 |
| ENSG00000204685.6 | STARD7-AS1 | 1.0 | 5.3E-04 |
| ENSG00000205664.10 | RP11-706O15.1 | 1.1 | 6.7E-03 |
| ENSG00000214049.6 | UCA1 | 3.3 | 4.5E-04 |
| ENSG00000214773.1 | RP11-717D12.1 | 1.8 | 1.7E-03 |
| ENSG00000215386.10 | MIR99AHG | -6.4 | 1.2E-27 |
| ENSG00000215458.8 | AATBC | 3.6 | 2.5E-09 |
| ENSG00000223813.2 | AC007255.8 | 1.6 | 9.6E-03 |
| ENSG00000223947.1 | AC016738.4 | 1.5 | 8.2E-03 |
| ENSG00000224020.1 | MIR181A2HG | 1.3 | 6.4E-03 |
| ENSG00000224652.1 | LINC00885 | 1.9 | 3.2E-03 |
| ENSG00000224699.8 | LAMTOR5-AS1 | 1.6 | 2.0E-07 |
| ENSG00000224822.1 | THRB-IT1 | 2.4 | 3.0E-05 |
| ENSG00000225506.2 | CYP4A22-AS1 | 1.4 | 1.5E-03 |
| ENSG00000225655.5 | PGM5-AS1 | -4.6 | 2.6E-06 |
| ENSG00000225746.8 | SNHG23 | -2.2 | 4.7E-03 |
| ENSG00000226332.2 | RP11-157P1.4 | 1.5 | 3.1E-03 |
| ENSG00000226363.3 | HAGLROS | 2.0 | 1.7E-03 |
| ENSG00000226904.1 | RP11-561O23.8 | 2.5 | 5.7E-03 |
| ENSG00000227121.1 | RP11-319F12.2 | 2.9 | 8.9E-04 |
| ENSG00000227372.10 | TP73-AS1 | -2.3 | 4.2E-06 |
| ENSG00000227782.2 | CTC-529I10.2 | 1.1 | 6.3E-03 |
| ENSG00000228113.7 | AC003991.3 | 1.7 | 1.6E-03 |
| ENSG00000228274.3 | RP3-508I15.9 | 1.1 | 5.4E-03 |
| ENSG00000228302.2 | RP11-186N15.3 | 1.2 | 9.5E-06 |
| ENSG00000229539.1 | RP11-119B16.2 | 1.6 | 7.8E-06 |
| ENSG00000229694.6 | RP11-305L7.6 | -2.0 | 2.8E-04 |
| ENSG00000230074.1 | RP11-195F19.9 | -1.7 | 1.1E-03 |
| ENSG00000230177.1 | RP5-1112D6.4 | 1.2 | 4.6E-04 |
| ENSG00000230185.4 | C9orf147 | 1.6 | 1.5E-03 |
| ENSG00000230337.1 | RP4-635E18.6 | 2.2 | 6.8E-03 |
| ENSG00000230630.2 | DNM3OS | -2.4 | 2.4E-09 |
| ENSG00000231439.4 | WASIR2 | 3.3 | 2.3E-06 |
| ENSG00000231607.8 | DLEU2 | 1.2 | 3.2E-04 |
| ENSG00000231721.6 | LINC-PINT | -2.0 | 2.2E-07 |
| ENSG00000231764.8 | DLX6-AS1 | 1.8 | 7.8E-03 |
| ENSG00000232229.5 | LINC00865 | -1.8 | 3.7E-04 |
| ENSG00000232855.6 | AF131217.1 | -4.1 | 4.1E-16 |
| ENSG00000233098.8 | CCDC144NL-AS1 | -2.2 | 6.1E-05 |
| ENSG00000233117.2 | LINC00702 | -4.9 | 6.0E-14 |
| ENSG00000233175.2 | CTD-2020K17.3 | 1.6 | 4.6E-03 |
| ENSG00000234456.7 | MAGI2-AS3 | -3.3 | 8.7E-13 |
| ENSG00000234546.2 | RP3-510D11.2 | 2.3 | 1.6E-06 |
| ENSG00000234638.1 | AC053503.6 | -7.9 | 2.3E-17 |
| ENSG00000234771.3 | SLC25A25-AS1 | 1.1 | 9.1E-04 |
| ENSG00000234912.9 | SNHG20 | 1.2 | 2.5E-03 |
| ENSG00000234945.7 | GTF3C2-AS1 | 1.6 | 3.2E-03 |
| ENSG00000235280.2 | MCF2L-AS1 | 1.7 | 5.5E-04 |
| ENSG00000235531.9 | MSC-AS1 | -3.6 | 1.2E-17 |
| ENSG00000235872.2 | RP11-335O4.3 | 2.0 | 2.5E-03 |
| ENSG00000236200.5 | KDM4A-AS1 | 1.3 | 1.2E-04 |
| ENSG00000237125.8 | HAND2-AS1 | -4.7 | 2.5E-20 |
| ENSG00000237187.8 | NR2F1-AS1 | -2.3 | 1.1E-08 |
| ENSG00000237718.2 | AC009095.4 | 1.4 | 1.6E-03 |
| ENSG00000237978.5 | KCNMB2-AS1 | 2.3 | 7.3E-03 |
| ENSG00000239415.1 | AP001469.9 | 1.5 | 3.1E-03 |
| ENSG00000242282.6 | AC108488.4 | -1.6 | 7.8E-04 |
| ENSG00000242622.1 | RP11-18H7.1 | 1.7 | 6.9E-04 |
| ENSG00000242795.2 | RP11-3K16.2 | 3.1 | 8.6E-10 |
| ENSG00000243004.5 | AC005062.2 | -2.3 | 5.7E-05 |
| ENSG00000243368.2 | MCCC1-AS1 | 1.5 | 5.1E-05 |
| ENSG00000243701.5 | LINC00883 | -1.6 | 7.1E-03 |
| ENSG00000246334.2 | PRR7-AS1 | 2.0 | 3.0E-04 |
| ENSG00000246366.6 | LACTB2-AS1 | 1.5 | 2.0E-04 |
| ENSG00000246526.2 | RP11-539L10.2 | 2.6 | 5.5E-07 |
| ENSG00000246695.7 | RASSF8-AS1 | -1.7 | 1.2E-03 |
| ENSG00000247516.4 | MIR4458HG | -1.9 | 6.4E-03 |
| ENSG00000248309.5 | MEF2C-AS1 | -4.1 | 2.0E-13 |
| ENSG00000249082.2 | C5orf66-AS1 | -3.3 | 5.3E-05 |
| ENSG00000250132.6 | RP11-359B12.2 | -1.1 | 8.4E-03 |
| ENSG00000250303.3 | RP11-356J5.12 | -2.6 | 2.2E-06 |
| ENSG00000251661.3 | RP11-326C3.11 | -1.8 | 2.1E-03 |
| ENSG00000253106.1 | RP11-158K1.3 | 1.1 | 1.9E-03 |
| ENSG00000253854.1 | RP11-10N23.2 | 1.5 | 4.5E-03 |
| ENSG00000254510.1 | RP11-867G23.10 | -5.5 | 2.4E-16 |
| ENSG00000254531.1 | FLJ20021 | -1.1 | 5.4E-03 |
| ENSG00000254951.7 | RP11-494M8.4 | 1.6 | 6.2E-03 |
| ENSG00000255090.5 | MIR100HG | -3.3 | 9.6E-10 |
| ENSG00000255495.1 | AC145124.2 | -1.4 | 4.1E-03 |
| ENSG00000255717.6 | SNHG1 | 1.1 | 3.8E-03 |
| ENSG00000256546.1 | AC156455.1 | -2.4 | 4.4E-04 |
| ENSG00000257475.2 | RP11-983P16.2 | 2.0 | 4.8E-04 |
| ENSG00000257596.1 | RP11-968A15.2 | 2.4 | 2.1E-05 |
| ENSG00000257671.1 | RP3-416H24.1 | 1.5 | 7.5E-03 |
| ENSG00000257698.1 | RP11-620J15.3 | -2.2 | 3.1E-04 |
| ENSG00000258199.1 | RP11-977G19.5 | 3.6 | 1.6E-09 |
| ENSG00000258559.2 | AC005519.4 | 1.8 | 1.6E-04 |
| ENSG00000259250.1 | RP11-50C13.1 | 1.8 | 1.5E-03 |
| ENSG00000259291.2 | RP11-617F23.1 | -1.7 | 4.1E-03 |
| ENSG00000259321.1 | RP11-468E2.5 | 2.5 | 7.6E-04 |
| ENSG00000259654.1 | RP11-245C17.2 | 2.2 | 2.6E-04 |
| ENSG00000259865.1 | RP11-488L18.10 | 1.2 | 1.4E-03 |
| ENSG00000259976.1 | RP11-553L6.5 | -2.0 | 1.3E-10 |
| ENSG00000260005.6 | AC027601.1 | 1.4 | 5.3E-03 |
| ENSG00000260077.1 | RP11-254F7.2 | -1.6 | 1.5E-04 |
| ENSG00000260337.3 | RP11-386M24.6 | 1.8 | 5.0E-04 |
| ENSG00000260361.1 | CTD-2313J17.5 | 1.1 | 4.9E-03 |
| ENSG00000260708.1 | CTA-29F11.1 | -1.7 | 2.0E-03 |
| ENSG00000260774.1 | CTD-2083E4.4 | 1.0 | 5.3E-03 |
| ENSG00000261068.2 | RP11-7K24.3 | 1.8 | 8.2E-03 |
| ENSG00000261762.1 | RP11-650L12.2 | 2.2 | 2.7E-04 |
| ENSG00000261766.1 | RP11-22P6.2 | 1.7 | 7.4E-05 |
| ENSG00000262001.1 | DLGAP1-AS2 | 1.0 | 6.8E-03 |

**PRO**

| gene_id | gene_name | log2FC | FDR |
|---|---|---|---|
| ENSG00000154537.4 | FAM27C | -1.2 | 1.8E-03 |
| ENSG00000157306.14 | RP11-66N24.4 | 1.3 | 2.4E-04 |
| ENSG00000172250.14 | SERHL | 1.3 | 9.2E-04 |
| ENSG00000179406.6 | LINC00174 | 1.2 | 1.4E-04 |
| ENSG00000182109.7 | RP11-69E11.4 | 1.1 | 1.0E-04 |
| ENSG00000184224.3 | C11orf72 | 1.0 | 1.1E-04 |
| ENSG00000196295.11 | AC005154.6 | 1.1 | 2.7E-05 |
| ENSG00000197815.4 | RP1-253P7.4 | 1.5 | 1.0E-06 |
| ENSG00000198590.11 | C3orf35 | 1.5 | 1.1E-07 |
| ENSG00000203620.2 | RP11-84A19.2 | 1.3 | 5.5E-04 |
| ENSG00000203739.3 | RP11-296O14.3 | 1.0 | 3.3E-04 |
| ENSG00000205861.11 | C1QTNF9B-AS1 | 1.4 | 6.7E-03 |
| ENSG00000205913.6 | SRRM2-AS1 | 1.2 | 4.7E-09 |
| ENSG00000213121.2 | RP11-306O13.1 | 1.7 | 2.6E-10 |
| ENSG00000216921.7 | AC131097.4 | -1.9 | 1.5E-05 |
| ENSG00000223400.1 | AP006748.1 | 1.9 | 5.8E-03 |
| ENSG00000223745.7 | RP4-717I23.3 | 1.5 | 4.0E-09 |
| ENSG00000224259.5 | LINC01133 | -1.7 | 2.3E-04 |
| ENSG00000224660.1 | SH3BP5-AS1 | 1.2 | 2.9E-04 |
| ENSG00000224843.6 | LINC00240 | 1.3 | 8.7E-05 |
| ENSG00000224961.1 | RP1-278O22.1 | -1.1 | 7.6E-05 |
| ENSG00000225177.5 | RP11-390P2.4 | 1.4 | 3.3E-08 |
| ENSG00000225313.5 | RP11-415J8.3 | 1.0 | 7.1E-03 |
| ENSG00000225880.5 | LINC00115 | 1.0 | 1.2E-03 |
| ENSG00000225914.1 | XXbac-BPG154L12.4 | 1.5 | 5.5E-11 |
| ENSG00000225937.1 | PCA3 | 3.2 | 2.4E-08 |
| ENSG00000226051.6 | ZNF503-AS1 | -1.9 | 1.8E-10 |
| ENSG00000226803.7 | RP11-203B9.4 | 1.3 | 3.6E-05 |
| ENSG00000227719.1 | AC006042.6 | 1.5 | 3.2E-05 |
| ENSG00000228109.1 | MFI2-AS1 | 1.1 | 5.3E-04 |
| ENSG00000228393.3 | LINC01004 | 1.2 | 1.1E-05 |
| ENSG00000228486.9 | LINC01125 | 1.2 | 9.2E-06 |
| ENSG00000228798.1 | AP000473.5 | 1.7 | 4.0E-05 |
| ENSG00000228862.3 | RP11-91I20.3 | -1.5 | 3.4E-05 |
| ENSG00000230082.1 | PRRT3-AS1 | 1.6 | 9.1E-07 |
| ENSG00000230454.1 | U73166.2 | 1.6 | 1.7E-05 |
| ENSG00000230590.7 | FTX | 1.2 | 3.7E-04 |
| ENSG00000230732.4 | AC127904.2 | 1.1 | 1.5E-03 |
| ENSG00000231609.5 | AC009501.4 | 1.2 | 8.4E-06 |
| ENSG00000231711.2 | LINC00899 | 1.2 | 4.5E-06 |
| ENSG00000231789.2 | PIK3CD-AS2 | -1.5 | 2.7E-07 |
| ENSG00000231806.2 | PCAT7 | 1.7 | 1.0E-08 |
| ENSG00000232104.3 | RFX3-AS1 | 1.3 | 2.1E-05 |
| ENSG00000232442.1 | CTD-3184A7.4 | 1.3 | 1.5E-06 |
| ENSG00000232611.1 | RP11-1114A5.4 | 1.1 | 2.3E-04 |
| ENSG00000232774.7 | FLJ22447 | 1.5 | 5.2E-13 |
| ENSG00000232931.5 | LINC00342 | 1.5 | 4.9E-05 |
| ENSG00000232973.11 | CYP1B1-AS1 | 1.0 | 5.3E-04 |
| ENSG00000233008.5 | RP11-475O6.1 | 1.3 | 3.4E-06 |
| ENSG00000233461.5 | RP11-295G20.2 | 1.2 | 2.1E-04 |
| ENSG00000233901.5 | LINC01503 | -1.3 | 1.8E-04 |
| ENSG00000233967.6 | RP11-250B2.3 | 1.2 | 1.4E-03 |
| ENSG00000234753.5 | FOXP4-AS1 | 1.9 | 1.8E-08 |
| ENSG00000234949.2 | AC104667.3 | 1.9 | 1.3E-05 |
| ENSG00000235475.1 | LINC01372 | 2.1 | 2.0E-10 |
| ENSG00000235703.5 | LINC00894 | 1.3 | 3.4E-04 |
| ENSG00000236991.6 | EDRF1-AS1 | 1.6 | 1.0E-04 |
| ENSG00000237188.3 | RP11-337C18.8 | 1.1 | 5.1E-07 |
| ENSG00000237343.1 | RP11-763B22.4 | 1.5 | 1.3E-03 |
| ENSG00000237457.6 | LINC01351 | 1.1 | 7.2E-03 |
| ENSG00000237491.8 | RP11-206L10.9 | 1.0 | 7.0E-05 |
| ENSG00000237883.1 | DGUOK-AS1 | 1.1 | 4.5E-04 |
| ENSG00000237903.1 | RP3-404F18.5 | 1.5 | 6.3E-04 |
| ENSG00000238009.6 | RP11-34P13.7 | 1.2 | 4.8E-04 |
| ENSG00000241769.7 | LINC00893 | 1.3 | 2.1E-06 |
| ENSG00000242349.5 | NPPA-AS1 | 1.8 | 6.1E-06 |
| ENSG00000243069.7 | ARHGEF26-AS1 | 1.2 | 9.2E-06 |
| ENSG00000244879.4 | GABPB1-AS1 | 1.5 | 3.6E-08 |
| ENSG00000245532.5 | NEAT1 | 1.6 | 5.1E-10 |
| ENSG00000245552.6 | RP11-712B9.2 | 1.1 | 4.0E-03 |
| ENSG00000245888.6 | FLJ21408 | 1.6 | 4.4E-08 |
| ENSG00000246763.6 | RGMB-AS1 | 1.1 | 4.8E-05 |
| ENSG00000247287.2 | RP11-902B17.1 | 1.4 | 4.0E-08 |
| ENSG00000247679.2 | RP11-1277A3.1 | 1.2 | 2.2E-04 |
| ENSG00000247828.7 | TMEM161B-AS1 | 1.1 | 3.9E-07 |
| ENSG00000248019.2 | FAM13A-AS1 | 1.1 | 1.3E-04 |
| ENSG00000248092.7 | NNT-AS1 | 1.1 | 1.8E-03 |
| ENSG00000248663.6 | LINC00992 | 1.2 | 7.1E-03 |
| ENSG00000249307.5 | LINC01088 | 1.8 | 3.1E-05 |
| ENSG00000249352.4 | RP11-141O11.2 | 1.1 | 7.2E-03 |
| ENSG00000249885.1 | ARHGEF38-IT1 | 2.0 | 4.1E-07 |
| ENSG00000250237.1 | CTC-498J12.1 | 1.3 | 2.7E-03 |
| ENSG00000250786.1 | SNHG18 | -1.6 | 3.1E-05 |
| ENSG00000253438.2 | PCAT1 | 2.2 | 5.0E-13 |
| ENSG00000254489.1 | RP5-1024C24.1 | -1.6 | 5.7E-04 |
| ENSG00000255240.5 | RP11-142C4.6 | 1.6 | 4.7E-07 |
| ENSG00000255348.1 | RP11-700F16.3 | 1.5 | 1.1E-03 |
| ENSG00000255435.6 | RP11-770J1.3 | 1.1 | 4.0E-04 |
| ENSG00000255545.7 | RP11-627G23.1 | 3.3 | 5.8E-13 |
| ENSG00000256433.2 | RP1-102E24.8 | 1.6 | 1.1E-08 |
| ENSG00000257002.1 | AP000438.2 | 1.2 | 7.7E-03 |
| ENSG00000257605.2 | RP11-680A11.5 | 1.0 | 1.7E-03 |
| ENSG00000257681.1 | RP11-341G23.4 | 1.4 | 7.8E-04 |
| ENSG00000257989.1 | RP11-288H2.2 | 1.3 | 7.2E-04 |
| ENSG00000258458.5 | CTD-2555K7.2 | 1.4 | 1.6E-07 |
| ENSG00000258727.1 | RP11-66N24.3 | 1.9 | 5.3E-08 |
| ENSG00000258867.5 | LINC01146 | 1.1 | 6.0E-03 |
| ENSG00000259287.2 | RP11-3D4.2 | 1.2 | 6.7E-05 |
| ENSG00000259457.1 | RP11-279F6.2 | 1.7 | 2.5E-03 |
| ENSG00000259583.2 | RP11-66B24.4 | 1.6 | 3.7E-06 |
| ENSG00000259641.5 | PCAT29 | 2.7 | 4.4E-15 |
| ENSG00000259659.1 | RP11-151N17.1 | 1.4 | 6.4E-05 |
| ENSG00000259704.1 | CTD-3094K11.1 | -2.4 | 2.1E-13 |
| ENSG00000259972.2 | AC009120.6 | 1.0 | 5.1E-04 |
| ENSG00000260095.1 | RP11-715J22.3 | 1.2 | 7.1E-04 |
| ENSG00000260228.5 | RP11-483P21.2 | 1.9 | 4.8E-04 |
| ENSG00000260296.1 | RP11-395I6.3 | 1.5 | 1.2E-05 |
| ENSG00000260317.1 | RP11-48B3.4 | 1.3 | 6.7E-05 |
| ENSG00000260565.6 | CTD-2270P14.1 | 1.0 | 6.2E-05 |
| ENSG00000260884.1 | AC009120.5 | 1.1 | 1.0E-03 |
| ENSG00000260896.5 | PRCAT47 | 2.6 | 1.7E-05 |
| ENSG00000260923.5 | AC137934.1 | 1.4 | 4.9E-09 |
| ENSG00000261159.1 | RP11-723O4.9 | 1.7 | 4.2E-06 |

**ADC**

| gene_id | gene_name | log2FC | FDR |
|---|---|---|---|
| ENSG00000177788.5 | RP5-1061H20.4 | 1.7 | 2.8E-08 |
| ENSG00000183250.11 | LINC01547 | 1.8 | 6.7E-11 |
| ENSG00000188511.12 | C22orf34 | -1.4 | 5.8E-11 |
| ENSG00000196167.9 | COLCA1 | 1.3 | 1.3E-04 |
| ENSG00000197291.8 | RAMP2-AS1 | -1.3 | 2.6E-16 |
| ENSG00000203804.4 | ADAMTSL4-AS1 | -1.4 | 9.2E-07 |
| ENSG00000204055.4 | RP11-247A12.2 | 1.4 | 3.3E-03 |
| ENSG00000204623.8 | ZNRD1-AS1 | 1.0 | 2.3E-33 |
| ENSG00000205037.2 | RP11-863P13.4 | -2.1 | 8.7E-10 |
| ENSG00000214708.4 | AC090616.2 | -1.6 | 6.4E-07 |
| ENSG00000219665.8 | CTD-2006C1.2 | 1.0 | 8.9E-12 |
| ENSG00000223473.2 | GS1-124K5.3 | 1.1 | 2.3E-18 |
| ENSG00000223561.6 | AC003090.1 | -1.1 | 4.3E-03 |
| ENSG00000224023.10 | FLJ37035 | 1.3 | 2.5E-03 |
| ENSG00000224239.1 | AC090044.2 | -1.1 | 1.9E-05 |
| ENSG00000224397.5 | LINC01272 | -1.3 | 3.2E-05 |
| ENSG00000224888.4 | RP5-1142A6.2 | -1.2 | 4.6E-06 |
| ENSG00000225342.2 | AC079630.4 | -1.1 | 7.0E-04 |
| ENSG00000225473.1 | ATP13A4-AS1 | 2.1 | 2.8E-03 |
| ENSG00000225792.1 | AC004540.4 | -2.1 | 9.7E-09 |
| ENSG00000225873.1 | LINC00694 | -1.7 | 3.0E-05 |
| ENSG00000225938.1 | RP4-575N6.4 | -2.5 | 1.6E-22 |
| ENSG00000226239.1 | RP1-310O13.7 | -2.1 | 2.0E-08 |
| ENSG00000226598.1 | AC017060.1 | 2.0 | 7.1E-07 |
| ENSG00000226871.1 | AC135178.7 | -2.3 | 6.9E-05 |
| ENSG00000227036.6 | LINC00511 | 2.9 | 8.6E-18 |
| ENSG00000227066.1 | RP3-340N1.2 | 4.6 | 6.9E-22 |
| ENSG00000228221.5 | LINC00578 | 1.9 | 9.7E-11 |
| ENSG00000228265.5 | RALY-AS1 | 1.0 | 4.1E-06 |
| ENSG00000228401.4 | RP11-251M1.1 | -3.6 | 1.4E-18 |
| ENSG00000229512.1 | AC068580.5 | 1.3 | 4.1E-04 |
| ENSG00000229867.1 | STEAP3-AS1 | 1.4 | 4.4E-06 |
| ENSG00000230148.8 | HOXB-AS1 | 1.1 | 2.2E-06 |
| ENSG00000230551.4 | CTB-89H12.4 | 1.1 | 1.6E-05 |
| ENSG00000230615.6 | RP5-1198O20.4 | -1.8 | 6.1E-10 |
| ENSG00000231106.2 | LINC01436 | 3.0 | 3.3E-15 |
| ENSG00000231187.2 | RP11-38L15.3 | -1.4 | 8.7E-09 |
| ENSG00000232415.1 | CTB-51J22.1 | -1.1 | 4.3E-04 |
| ENSG00000232803.1 | SLCO4A1-AS1 | -2.3 | 2.5E-06 |
| ENSG00000232814.2 | COL4A2-AS1 | -1.4 | 5.4E-06 |
| ENSG00000232878.3 | DPYD-AS1 | 1.9 | 1.7E-03 |
| ENSG00000233038.5 | AC011899.9 | -1.3 | 5.4E-06 |
| ENSG00000233237.6 | LINC00472 | -2.4 | 1.9E-15 |
| ENSG00000233834.6 | AC005083.1 | 1.5 | 4.7E-16 |
| ENSG00000234211.2 | RP11-25K21.1 | 3.0 | 2.3E-08 |
| ENSG00000234281.5 | LANCL1-AS1 | -2.5 | 4.8E-19 |
| ENSG00000234380.1 | LINC01426 | 3.2 | 5.1E-27 |
| ENSG00000234617.1 | SNRK-AS1 | -1.3 | 9.1E-04 |
| ENSG00000235027.1 | AC068580.6 | 1.6 | 1.4E-06 |
| ENSG00000235385.1 | GS1-600G8.5 | -3.0 | 7.5E-13 |
| ENSG00000235387.1 | LINC00961 | -3.1 | 5.0E-21 |
| ENSG00000235770.5 | LINC00607 | -1.7 | 4.7E-18 |
| ENSG00000235997.2 | AC109642.1 | -2.1 | 2.1E-09 |
| ENSG00000236242.1 | MYO16-AS1 | -4.2 | 6.1E-36 |
| ENSG00000236304.1 | AP001189.4 | -3.2 | 3.8E-27 |
| ENSG00000236810.5 | TCEB3-AS1 | 1.1 | 3.3E-06 |
| ENSG00000236830.6 | CBR3-AS1 | 1.1 | 1.3E-05 |
| ENSG00000236833.1 | AC024560.2 | 1.2 | 7.6E-05 |
| ENSG00000237523.1 | LINC00857 | 1.1 | 1.9E-05 |
| ENSG00000237781.3 | RP11-54A4.2 | -1.4 | 2.3E-05 |
| ENSG00000238018.2 | AC093110.3 | -2.4 | 1.2E-21 |
| ENSG00000238057.8 | ZEB2-AS1 | -1.3 | 2.4E-04 |
| ENSG00000238258.1 | RP11-342D11.2 | 1.5 | 5.8E-03 |
| ENSG00000239268.2 | RP11-384F7.2 | -3.1 | 1.8E-20 |
| ENSG00000243961.2 | RP5-839B4.8 | -1.5 | 1.3E-03 |
| ENSG00000244124.1 | ATP1B3-AS1 | -2.4 | 2.2E-12 |
| ENSG00000244300.2 | GATA2-AS1 | -1.7 | 1.7E-08 |
| ENSG00000244513.6 | CTD-2013N24.2 | -1.4 | 1.1E-11 |
| ENSG00000244953.1 | RP11-613D13.8 | -3.5 | 1.0E-24 |
| ENSG00000245694.8 | CRNDE | 1.3 | 1.3E-05 |
| ENSG00000246273.6 | SBF2-AS1 | 1.3 | 5.2E-09 |
| ENSG00000246430.6 | LINC00968 | -3.8 | 1.6E-49 |
| ENSG00000246582.2 | RP11-1149O23.3 | 1.0 | 2.3E-04 |
| ENSG00000247137.8 | RP11-727A23.5 | 1.1 | 1.5E-09 |
| ENSG00000247317.3 | RP11-273G15.2 | 2.2 | 1.1E-09 |
| ENSG00000249042.5 | CTD-2015H6.3 | 2.0 | 3.3E-11 |
| ENSG00000249096.6 | RP11-290F5.1 | 1.8 | 1.2E-07 |
| ENSG00000249364.5 | RP11-434D9.1 | -2.0 | 8.2E-06 |
| ENSG00000249430.1 | CTD-2231H16.1 | 1.2 | 2.0E-04 |
| ENSG00000249592.5 | RP11-440L14.1 | 1.3 | 5.5E-27 |
| ENSG00000249835.2 | VCAN-AS1 | 1.8 | 6.6E-03 |
| ENSG00000250073.2 | RP11-677M14.3 | -1.8 | 8.5E-15 |
| ENSG00000250899.3 | RP11-253E3.3 | -2.4 | 4.4E-66 |
| ENSG00000250978.5 | RP11-357D18.1 | -1.8 | 3.8E-09 |
| ENSG00000251003.7 | ZFPM2-AS1 | 4.7 | 9.8E-34 |
| ENSG00000251230.5 | RP11-701P16.5 | -3.1 | 2.0E-16 |
| ENSG00000251364.6 | CTD-2516F10.2 | 1.8 | 1.2E-13 |
| ENSG00000253320.5 | AZIN1-AS1 | 1.5 | 1.3E-05 |
| ENSG00000253364.1 | RP11-731F5.2 | 1.6 | 3.2E-03 |
| ENSG00000253522.3 | CTC-231O11.1 | 1.8 | 9.8E-05 |
| ENSG00000253671.1 | RP11-806O11.1 | 1.2 | 5.0E-05 |
| ENSG00000253716.5 | LINC01604 | 1.1 | 1.4E-04 |
| ENSG00000253944.1 | RP11-156K13.1 | 1.1 | 1.4E-03 |
| ENSG00000254703.2 | SENCR | -1.5 | 1.0E-09 |
| ENSG00000254837.2 | AP001372.2 | 1.1 | 8.2E-08 |
| ENSG00000255026.1 | RP11-326C3.2 | 1.6 | 8.9E-05 |
| ENSG00000255197.5 | RP11-750H9.5 | -1.2 | 2.4E-06 |
| ENSG00000255399.3 | TBX5-AS1 | -1.8 | 1.1E-13 |
| ENSG00000255847.5 | RP11-167N4.2 | 2.2 | 5.3E-06 |
| ENSG00000256948.1 | RP11-598F7.3 | -1.5 | 3.6E-08 |
| ENSG00000257261.5 | RP11-96H19.1 | 1.2 | 4.4E-06 |
| ENSG00000257474.5 | RP11-359M6.1 | -1.5 | 6.0E-04 |
| ENSG00000258959.1 | RP11-1017G21.4 | -1.1 | 2.9E-06 |
| ENSG00000259153.1 | RP6-65G23.3 | 2.1 | 4.8E-23 |
| ENSG00000259772.6 | RP11-16E12.2 | 2.2 | 2.1E-13 |
| ENSG00000259925.1 | CTA-363E6.2 | 1.3 | 5.9E-03 |
| ENSG00000260083.1 | MIR762HG | 1.2 | 1.0E-03 |
| ENSG00000260114.2 | CTD-2574D22.4 | 1.5 | 1.4E-13 |
| ENSG00000260121.1 | RP5-1142A6.9 | -1.0 | 1.0E-11 |
| ENSG00000260219.2 | RP11-347C12.10 | 1.3 | 1.4E-07 |
| ENSG00000260231.1 | JHDM1D-AS1 | 1.0 | 1.8E-03 |
| ENSG00000260468.1 | LINC01290 | -1.8 | 1.2E-08 |
| ENSG00000260592.1 | CTA-363E6.6 | 1.2 | 3.5E-06 |

**EBC**

| gene_id | gene_name | log2FC | FDR |
|---|---|---|---|
| ENSG00000116883.8 | RP11-268J15.5 | 1.3 | 3.3E-08 |
| ENSG00000130600.15 | H19 | -1.1 | 4.3E-03 |
| ENSG00000175061.17 | LRRC75A-AS1 | -1.0 | 8.2E-05 |
| ENSG00000196421.7 | LINC00176 | 1.9 | 2.8E-14 |
| ENSG00000196668.3 | LINC00173 | 1.5 | 4.0E-06 |
| ENSG00000197308.8 | GATA3-AS1 | 2.4 | 1.0E-09 |
| ENSG00000205790.1 | DPP9-AS1 | 1.1 | 1.2E-08 |
| ENSG00000213904.8 | LIPE-AS1 | -1.8 | 3.0E-11 |
| ENSG00000214733.8 | RP11-429J17.8 | 2.1 | 2.4E-12 |
| ENSG00000218416.4 | PP14571 | 2.4 | 3.6E-09 |
| ENSG00000218537.1 | MIF-AS1 | -1.1 | 1.7E-03 |
| ENSG00000222041.10 | LINC00152 | 1.2 | 1.2E-09 |
| ENSG00000223356.1 | RP11-66D17.5 | 1.1 | 4.8E-08 |
| ENSG00000223764.2 | RP11-54O7.3 | 2.5 | 1.7E-09 |
| ENSG00000224195.1 | RP11-574K11.5 | 1.2 | 2.9E-07 |
| ENSG00000224376.1 | AC017104.6 | 1.3 | 1.6E-07 |
| ENSG00000224559.2 | LINC01087 | 2.2 | 1.1E-18 |
| ENSG00000224842.2 | RP11-123K19.1 | 1.5 | 4.7E-06 |
| ENSG00000225075.1 | RP11-426L16.3 | 1.1 | 2.7E-05 |
| ENSG00000225243.5 | RP1-127D3.4 | -1.0 | 3.8E-03 |
| ENSG00000225361.3 | PPP1R26-AS1 | 1.1 | 1.1E-22 |
| ENSG00000225756.1 | DBH-AS1 | 1.1 | 2.0E-06 |
| ENSG00000225951.1 | ODF2-AS1 | 1.5 | 1.7E-16 |
| ENSG00000226026.5 | RP11-57H12.3 | 1.4 | 5.7E-12 |
| ENSG00000226647.2 | RP11-336A10.2 | 1.4 | 1.1E-11 |
| ENSG00000226706.1 | RP11-426A6.5 | 1.3 | 2.6E-11 |
| ENSG00000226758.1 | DISC1-IT1 | 1.9 | 2.9E-12 |
| ENSG00000227195.8 | MIR663AHG | 1.1 | 3.7E-04 |
| ENSG00000227220.1 | RP11-69I8.3 | -1.2 | 2.2E-03 |
| ENSG00000227484.1 | XX-C283C717.1 | 1.2 | 3.3E-05 |
| ENSG00000227591.5 | RP1-28O10.1 | -3.9 | 4.5E-16 |
| ENSG00000228343.1 | RP11-1148L6.5 | -1.0 | 1.4E-05 |
| ENSG00000228613.1 | AC144450.1 | 1.7 | 1.2E-07 |
| ENSG00000228748.2 | RP13-39P12.3 | 1.2 | 6.7E-07 |
| ENSG00000228971.2 | RP11-286B14.1 | -2.0 | 9.3E-12 |
| ENSG00000229502.5 | RP11-52J3.2 | 1.2 | 5.6E-09 |
| ENSG00000230061.2 | TRPM2-AS | 2.1 | 8.6E-12 |
| ENSG00000230113.1 | AC091177.1 | -1.1 | 1.5E-03 |
| ENSG00000230487.7 | PSMG3-AS1 | -1.1 | 1.9E-10 |
| ENSG00000230530.1 | LIMD1-AS1 | -1.1 | 1.7E-04 |
| ENSG00000230612.3 | AC004237.1 | 1.2 | 1.1E-07 |
| ENSG00000231064.5 | RP11-263K19.4 | 1.0 | 8.3E-09 |
| ENSG00000231194.1 | FARP1-AS1 | 1.7 | 5.5E-07 |
| ENSG00000231367.5 | AC016995.3 | -1.0 | 1.8E-05 |
| ENSG00000231616.8 | RP11-575L7.4 | 1.5 | 6.5E-10 |
| ENSG00000231856.2 | RP11-327P2.5 | -1.0 | 1.6E-13 |
| ENSG00000231868.1 | RP1-202O8.2 | 2.0 | 4.4E-10 |
| ENSG00000232079.6 | AL035610.1 | 1.5 | 1.1E-07 |
| ENSG00000232600.3 | TONSL-AS1 | 1.1 | 2.3E-15 |
| ENSG00000232682.2 | RP11-388P9.2 | 1.3 | 4.9E-07 |
| ENSG00000233613.5 | DCUN1D2-AS | 1.2 | 2.6E-13 |
| ENSG00000234160.1 | RP11-613M10.6 | -1.6 | 3.7E-12 |
| ENSG00000234614.1 | AL450992.2 | -1.2 | 1.8E-10 |
| ENSG00000234630.1 | LL22NC03-2H8.4 | -1.4 | 1.2E-05 |
| ENSG00000234828.7 | RP11-526A4.1 | 1.6 | 6.5E-10 |
| ENSG00000234961.1 | RP11-124N14.3 | -2.1 | 5.6E-04 |
| ENSG00000235033.7 | RP11-61I13.3 | -1.8 | 2.5E-09 |
| ENSG00000235191.1 | NUCB1-AS1 | 1.3 | 6.0E-07 |
| ENSG00000235426.2 | RP11-342M3.5 | -1.4 | 2.5E-05 |
| ENSG00000235499.1 | AC073046.25 | -1.1 | 7.0E-07 |
| ENSG00000235535.7 | RP11-532N4.2 | 1.3 | 3.3E-07 |
| ENSG00000235725.1 | AC007389.3 | 1.1 | 3.4E-07 |
| ENSG00000235978.6 | AC018816.3 | 2.2 | 2.1E-07 |
| ENSG00000236333.3 | TRHDE-AS1 | -4.0 | 3.1E-18 |
| ENSG00000237017.1 | AC012314.8 | 1.3 | 1.7E-16 |
| ENSG00000237080.2 | EHMT2-AS1 | 1.7 | 1.3E-06 |
| ENSG00000237149.5 | ZNF503-AS2 | -1.2 | 3.9E-06 |
| ENSG00000237361.2 | TUSC8 | -1.5 | 1.3E-04 |
| ENSG00000237414.2 | RP11-321L2.2 | 1.1 | 4.7E-07 |
| ENSG00000237886.1 | LINC01573 | 1.1 | 2.9E-06 |
| ENSG00000238186.1 | RP11-656D10.6 | 1.0 | 1.2E-07 |
| ENSG00000241772.2 | AC092620.2 | 1.6 | 7.1E-11 |
| ENSG00000242396.1 | RP11-67L3.5 | 1.2 | 7.4E-03 |
| ENSG00000243836.5 | WDR86-AS1 | -1.8 | 1.3E-08 |
| ENSG00000244151.1 | RP11-148K1.12 | 1.4 | 2.1E-13 |
| ENSG00000244265.1 | SIAH2-AS1 | 2.5 | 1.2E-17 |
| ENSG00000245149.3 | RNF139-AS1 | 1.1 | 6.8E-13 |
| ENSG00000245904.3 | RP11-796E2.4 | -1.2 | 6.1E-06 |
| ENSG00000248367.1 | CTB-129O4.1 | 1.6 | 3.5E-10 |
| ENSG00000248458.2 | RP4-598P13.1 | 1.3 | 2.8E-08 |
| ENSG00000248774.1 | RP11-798M19.3 | -1.3 | 2.0E-06 |
| ENSG00000249409.1 | RP11-501E14.1 | 1.9 | 2.4E-12 |
| ENSG00000249456.1 | RP11-298J20.4 | -1.8 | 1.5E-05 |
| ENSG00000249846.6 | RP11-77P16.4 | 1.1 | 4.4E-11 |
| ENSG00000250127.1 | CTD-2117L12.1 | 1.3 | 6.3E-10 |
| ENSG00000250802.6 | ZBED3-AS1 | -1.1 | 2.2E-09 |
| ENSG00000251141.5 | RP11-53O19.1 | 2.5 | 2.7E-08 |
| ENSG00000253103.1 | LINC01609 | 1.5 | 5.5E-09 |
| ENSG00000253125.1 | RP11-459E5.1 | 3.2 | 8.0E-18 |
| ENSG00000253174.2 | RP11-360L9.7 | 2.2 | 1.3E-05 |
| ENSG00000253552.7 | HOXA-AS2 | -1.4 | 1.1E-09 |
| ENSG00000254057.1 | RP3-388N13.3 | 1.2 | 7.9E-07 |
| ENSG00000254343.2 | RP11-760H22.2 | -1.0 | 6.1E-05 |
| ENSG00000254459.1 | RP11-91P24.7 | 2.6 | 2.5E-12 |
| ENSG00000254538.1 | RP11-463D19.1 | 1.5 | 2.6E-12 |
| ENSG00000254548.1 | RP11-429J17.5 | 2.8 | 4.2E-12 |
| ENSG00000254814.1 | RP11-535A19.1 | -3.4 | 6.5E-16 |
| ENSG00000254973.1 | RP11-429J17.7 | 1.4 | 6.3E-22 |
| ENSG00000255031.5 | RP11-802E16.3 | 1.4 | 1.3E-08 |
| ENSG00000255142.1 | AP006621.6 | 1.1 | 1.7E-08 |
| ENSG00000255198.4 | SNHG9 | 1.7 | 8.4E-07 |
| ENSG00000255310.2 | AF131215.2 | -1.0 | 9.2E-05 |
| ENSG00000255449.1 | RP11-91P24.6 | 1.2 | 2.5E-06 |
| ENSG00000255455.2 | RP11-890B15.3 | -1.2 | 2.2E-11 |
| ENSG00000255568.3 | BRWD1-AS2 | 1.2 | 2.2E-08 |
| ENSG00000255928.1 | RP11-456I15.2 | 1.5 | 6.6E-11 |
| ENSG00000256116.1 | RP11-783K16.14 | 1.2 | 1.9E-04 |
| ENSG00000256984.1 | RP11-551L14.6 | 1.3 | 1.0E-04 |
| ENSG00000257084.1 | U47924.27 | 2.0 | 7.8E-14 |
| ENSG00000257086.1 | RP11-783K16.13 | 1.6 | 5.3E-15 |
| ENSG00000257509.1 | RP11-762I7.4 | 1.1 | 2.5E-12 |
| ENSG00000257808.1 | RP11-1136G11.8 | 2.0 | 7.4E-10 |
| ENSG00000257894.2 | RP1-78O14.1 | 1.0 | 1.5E-07 |

| | | | |
|---|---|---|---|
| ENSG00000262061.5 | RP11-1260E13.4 | -2.5 | 6.5E-04 |
| ENSG00000262251.1 | RP11-199F11.2 | 1.6 | 7.2E-04 |
| ENSG00000262848.1 | RP11-517A5.5 | 1.3 | 6.6E-03 |
| ENSG00000263335.1 | AF001548.5 | -2.7 | 2.4E-04 |
| ENSG00000263494.1 | AC004702.2 | 3.9 | 4.5E-05 |
| ENSG00000263657.1 | RP11-82O19.1 | 2.6 | 2.5E-07 |
| ENSG00000264304.1 | RP11-20B24.7 | 1.8 | 2.2E-03 |
| ENSG00000265100.1 | RP11-147L13.2 | 2.0 | 1.2E-03 |
| ENSG00000266261.1 | RP11-640I15.1 | 2.3 | 8.8E-03 |
| ENSG00000266651.1 | RP11-138I1.3 | 1.9 | 3.5E-03 |
| ENSG00000267080.5 | ASB16-AS1 | 1.1 | 1.2E-03 |
| ENSG00000267278.5 | MAP3K14-AS1 | 1.2 | 2.0E-04 |
| ENSG00000267288.2 | RP13-890H12.2 | 1.5 | 6.5E-03 |
| ENSG00000267505.1 | CTC-296K1.3 | -6.6 | 2.4E-18 |
| ENSG00000267565.1 | CTC-559E9.8 | 1.4 | 1.6E-03 |
| ENSG00000267612.1 | CTD-3116E22.7 | 2.0 | 6.1E-04 |
| ENSG00000267892.1 | CTD-2540F13.2 | 1.8 | 9.9E-04 |
| ENSG00000267896.1 | AC018766.4 | 2.3 | 5.2E-04 |
| ENSG00000267934.1 | CTB-176F20.3 | 1.4 | 3.4E-03 |
| ENSG00000268119.5 | CTD-2561J22.5 | 1.4 | 8.3E-03 |
| ENSG00000268362.5 | CTD-2017D11.1 | 1.0 | 1.1E-03 |
| ENSG00000269186.1 | LINC01082 | -4.1 | 3.4E-08 |
| ENSG00000269293.2 | ZSCAN16-AS1 | -1.6 | 5.2E-05 |
| ENSG00000269481.1 | CTD-2521M24.6 | 1.8 | 1.7E-08 |
| ENSG00000269609.5 | RPARP-AS1 | 1.5 | 2.2E-04 |
| ENSG00000269646.1 | CTD-2331H12.7 | 2.3 | 1.7E-04 |
| ENSG00000269815.1 | CTD-2278I10.4 | 1.4 | 9.1E-03 |
| ENSG00000269949.1 | RP11-738E22.3 | 2.9 | 7.5E-06 |
| ENSG00000269952.1 | RP11-324I22.3 | -2.9 | 1.0E-05 |
| ENSG00000269967.1 | RP11-84A19.4 | 2.3 | 4.6E-05 |
| ENSG00000269973.1 | RP11-95D17.1 | 1.4 | 1.7E-03 |
| ENSG00000270010.1 | CTD-2132N18.4 | 2.0 | 3.6E-04 |
| ENSG00000270049.1 | RP11-297D21.4 | 1.4 | 1.3E-03 |
| ENSG00000270069.1 | MIR222HG | -2.5 | 5.1E-06 |
| ENSG00000270195.1 | RP11-572O17.1 | 1.5 | 4.0E-03 |
| ENSG00000271579.1 | RP11-116D17.3 | 2.5 | 5.3E-04 |
| ENSG00000271584.1 | RP11-89C3.4 | -3.4 | 2.2E-09 |
| ENSG00000272129.1 | RP11-250B2.6 | 1.7 | 2.9E-03 |
| ENSG00000272140.2 | RP11-574K11.29 | 1.1 | 8.3E-03 |
| ENSG00000272155.1 | RP11-707M3.3 | 1.9 | 5.0E-07 |
| ENSG00000272159.1 | RP11-350N15.6 | -3.7 | 5.0E-04 |
| ENSG00000272256.1 | RP11-489E7.4 | -2.7 | 3.1E-05 |
| ENSG00000272405.1 | RP11-284F21.10 | 2.6 | 3.9E-05 |
| ENSG00000272440.1 | RP11-379F4.6 | 1.8 | 6.4E-03 |
| ENSG00000272667.1 | RP11-395A13.2 | -1.1 | 5.6E-03 |
| ENSG00000272870.1 | RP11-798M19.6 | -2.4 | 4.0E-08 |
| ENSG00000273013.1 | CTD-2002J20.1 | 2.2 | 5.5E-04 |
| ENSG00000273449.1 | RP11-218F10.3 | 1.3 | 1.4E-03 |
| ENSG00000273723.1 | RP11-93H24.3 | 1.8 | 2.1E-04 |
| ENSG00000273747.1 | RP11-90B9.3 | 1.7 | 4.0E-03 |
| ENSG00000274281.1 | RP11-326N17.2 | 1.4 | 7.0E-05 |
| ENSG00000274565.1 | CTD-3035K23.7 | -2.9 | 1.0E-07 |
| ENSG00000274627.1 | RP11-104N10.2 | 1.1 | 4.4E-03 |
| ENSG00000275056.1 | RP11-127I20.8 | 1.3 | 6.1E-03 |
| ENSG00000275202.1 | RP11-156K23.3 | -1.1 | 5.0E-03 |
| ENSG00000275393.1 | RP11-568J23.8 | 2.4 | 6.2E-05 |
| ENSG00000275512.1 | RP11-322E11.2 | 1.2 | 8.8E-04 |
| ENSG00000275601.1 | AC011330.13 | 1.2 | 4.6E-03 |
| ENSG00000276698.1 | RP11-468E2.10 | 1.5 | 1.6E-03 |
| ENSG00000276718.1 | RP1-102E24.10 | 1.8 | 4.0E-04 |
| ENSG00000277631.4 | PGM5P3-AS1 | -4.9 | 1.7E-14 |
| ENSG00000278126.1 | RP11-454E5.4 | -1.4 | 3.2E-03 |
| ENSG00000278185.1 | RP11-153F5.7 | 1.8 | 5.6E-03 |
| ENSG00000278743.1 | RP11-707G18.1 | -2.5 | 6.0E-06 |
| ENSG00000278891.1 | RP11-246E12.2 | 2.7 | 2.0E-05 |
| ENSG00000279029.1 | RP11-144I2.1 | 1.9 | 8.3E-05 |
| ENSG00000279352.1 | RP11-411B10.7 | 1.5 | 2.4E-03 |
| ENSG00000279382.1 | RP11-449J21.3 | 1.9 | 4.9E-05 |
| ENSG00000279495.1 | RP11-521B24.4 | 1.6 | 1.8E-07 |
| ENSG00000279519.1 | RP11-288C18.1 | 1.1 | 4.1E-03 |
| ENSG00000279696.1 | RP11-178H8.7 | 1.5 | 1.9E-03 |
| ENSG00000279742.1 | RP11-700A24.1 | 1.5 | 2.4E-03 |
| ENSG00000279786.1 | CTD-2014D20.1 | 2.4 | 5.7E-07 |
| ENSG00000279880.1 | RP11-855A2.2 | 1.9 | 2.1E-04 |
| ENSG00000280063.1 | RP11-295D4.3 | 1.8 | 2.1E-06 |
| ENSG00000280067.1 | CTD-2600H12.2 | 1.3 | 8.9E-03 |
| ENSG00000280069.1 | CTD-2349P21.3 | 1.9 | 8.6E-06 |
| ENSG00000280164.1 | CH507-254M2.3 | 1.4 | 9.0E-04 |
| ENSG00000280183.1 | RP11-400F19.12 | 2.0 | 1.1E-05 |
| ENSG00000280195.1 | XX-FW83563B9.5 | 1.2 | 4.6E-03 |
| ENSG00000280239.1 | CTB-50L17.8 | 2.3 | 9.2E-06 |
| ENSG00000280334.1 | RP11-361L15.5 | 1.9 | 6.3E-04 |
| ENSG00000280378.1 | RP4-758J24.6 | 1.6 | 6.0E-05 |
| ENSG00000280665.1 | RP11-35J6.1 | 2.2 | 6.3E-05 |
| ENSG00000261584.1 | RP11-457M11.5 | 2.1 | 2.9E-12 |
| ENSG00000261770.1 | CTC-459F4.1 | 1.6 | 9.5E-08 |
| ENSG00000261829.1 | RP11-223I10.1 | 1.7 | 2.4E-03 |
| ENSG00000262089.1 | RP11-589P10.5 | 1.1 | 6.8E-04 |
| ENSG00000262410.1 | RP11-388C12.8 | 1.3 | 9.4E-07 |
| ENSG00000263272.1 | CTC-524C5.2 | 1.5 | 2.9E-07 |
| ENSG00000264112.1 | RP11-159D12.2 | 1.6 | 4.8E-07 |
| ENSG00000265246.1 | RP11-663N22.1 | -1.8 | 8.6E-05 |
| ENSG00000265369.3 | PCAT18 | 1.4 | 4.8E-03 |
| ENSG00000266402.3 | SNHG25 | 1.3 | 8.2E-04 |
| ENSG00000266490.1 | CTD-2349P21.9 | 1.1 | 1.0E-02 |
| ENSG00000266644.1 | RP11-927P21.2 | 1.5 | 6.3E-05 |
| ENSG00000267163.1 | AC084219.3 | 1.7 | 2.7E-05 |
| ENSG00000267470.5 | ZNF571-AS1 | 1.0 | 3.0E-03 |
| ENSG00000267593.1 | RP11-108P20.4 | -1.6 | 2.8E-04 |
| ENSG00000267655.1 | CTD-2286N8.2 | 1.1 | 2.3E-03 |
| ENSG00000267838.2 | AC008746.12 | 1.2 | 3.7E-04 |
| ENSG00000270061.1 | RP11-214K3.19 | 1.7 | 4.8E-06 |
| ENSG00000270164.1 | LINC01480 | 1.1 | 9.7E-05 |
| ENSG00000270189.1 | RP11-258C19.7 | 1.1 | 4.6E-05 |
| ENSG00000270580.5 | PKD1P6 | 1.0 | 1.3E-03 |
| ENSG00000270720.1 | RP11-84C13.2 | 1.2 | 9.4E-04 |
| ENSG00000270871.1 | AC015849.19 | 1.0 | 9.6E-04 |
| ENSG00000271151.1 | RP11-394I13.2 | 2.7 | 4.6E-11 |
| ENSG00000271384.1 | RP11-435O5.7 | 1.3 | 3.1E-03 |
| ENSG00000271553.1 | RP11-274B21.10 | 1.2 | 1.4E-03 |
| ENSG00000271659.1 | RP11-435O5.4 | 1.3 | 8.4E-04 |
| ENSG00000271717.1 | CTD-3020H12.4 | 1.9 | 2.1E-08 |
| ENSG00000271870.1 | RP11-97C16.1 | 1.0 | 3.0E-03 |
| ENSG00000271941.1 | RP11-188P20.3 | 1.5 | 2.9E-04 |
| ENSG00000272301.1 | RP11-111M22.4 | 2.0 | 8.0E-08 |
| ENSG00000272316.1 | XXbac-BPGBPG55C20.[illegible] | 1.1 | 4.2E-06 |
| ENSG00000272330.1 | AC002044.4 | 1.2 | 7.4E-03 |
| ENSG00000272411.1 | RP11-44B19.1 | 1.1 | 8.0E-03 |
| ENSG00000272574.1 | RP11-359K18.4 | 1.5 | 1.7E-03 |
| ENSG00000272732.1 | RP5-1159O4.2 | 1.2 | 2.8E-04 |
| ENSG00000272807.1 | RP11-260M2.1 | 1.0 | 6.9E-03 |
| ENSG00000272894.5 | RP5-1159O4.1 | 1.3 | 4.4E-06 |
| ENSG00000273036.2 | FAM95C | 1.3 | 2.9E-03 |
| ENSG00000273179.1 | RP11-20I20.4 | 3.4 | 7.6E-13 |
| ENSG00000273212.1 | AC000068.10 | 1.6 | 8.1E-03 |
| ENSG00000273218.1 | LLNLR-246C6.1 | 1.1 | 7.6E-04 |
| ENSG00000273306.1 | RP11-527J8.1 | 1.2 | 6.2E-04 |
| ENSG00000274020.1 | LINC01138 | 1.2 | 2.6E-11 |
| ENSG00000274372.4 | RP11-94I2.4 | 3.2 | 2.6E-12 |
| ENSG00000274964.1 | RP11-817I4.1 | 1.1 | 8.0E-03 |
| ENSG00000275005.1 | RP11-234G16.5 | 1.6 | 1.5E-04 |
| ENSG00000275413.1 | CTC-529I10.1 | 1.8 | 1.1E-05 |
| ENSG00000276571.1 | AC002550.6 | 1.2 | 9.7E-04 |
| ENSG00000277978.1 | RP11-403P17.6 | 1.4 | 5.9E-03 |
| ENSG00000278002.1 | RP11-596C23.2 | 1.4 | 1.4E-05 |
| ENSG00000278932.3 | bP-21264C1.2 | 1.7 | 3.5E-04 |
| ENSG00000279103.1 | CTC-204F22.1 | 1.5 | 6.1E-08 |
| ENSG00000279289.1 | RP3-331H24.6 | 1.3 | 1.5E-04 |
| ENSG00000279500.1 | RP11-21K12.2 | 1.1 | 2.0E-03 |
| ENSG00000279549.1 | AP000437.3 | 1.4 | 1.3E-04 |
| ENSG00000279591.1 | AC002044.1 | 1.3 | 3.3E-04 |
| ENSG00000279891.1 | FLJ42393 | 1.3 | 3.9E-03 |
| ENSG00000280032.1 | RP11-832A4.7 | -1.3 | 5.7E-03 |
| ENSG00000280077.1 | RP11-87H9.4 | 1.1 | 4.0E-04 |
| ENSG00000280128.1 | XXbac-BPG283O16.9 | 1.1 | 1.1E-08 |
| ENSG00000280623.1 | PCAT14 | 2.2 | 5.7E-04 |
| ENSG00000280927.1 | CTBP1-AS | 2.7 | 8.2E-10 |
| ENSG00000281103.1 | TRG-AS1 | -1.6 | 1.7E-03 |
| ENSG00000281468.1 | CTC-459F4.9 | 1.3 | 1.6E-05 |
| ENSG00000282542.1 | AC008993.2 | 1.4 | 6.3E-03 |
| ENSG00000260841.1 | CTC-205M6.5 | -1.4 | 2.7E-22 |
| ENSG00000261338.2 | RP11-378A13.1 | -1.1 | 6.1E-05 |
| ENSG00000261634.3 | RP11-352D13.6 | -1.8 | 4.7E-05 |
| ENSG00000261662.1 | RP5-1042I8.7 | -1.0 | 2.4E-06 |
| ENSG00000261801.5 | LOXL1-AS1 | 1.3 | 1.1E-07 |
| ENSG00000261804.1 | RP11-44F14.2 | 1.4 | 1.7E-08 |
| ENSG00000261863.1 | RP11-141J13.5 | -3.3 | 2.5E-17 |
| ENSG00000263050.1 | RP11-667K14.3 | -2.7 | 4.2E-19 |
| ENSG00000263586.1 | HID1-AS1 | -2.7 | 8.8E-12 |
| ENSG00000264044.1 | RP11-192H23.7 | 1.6 | 1.1E-03 |
| ENSG00000264443.1 | RP4-594I10.3 | 1.8 | 3.9E-03 |
| ENSG00000265474.1 | AC010761.9 | 1.4 | 4.4E-03 |
| ENSG00000266010.1 | GATA6-AS1 | -1.5 | 9.5E-07 |
| ENSG00000266283.1 | RP11-627G18.1 | -2.0 | 1.0E-14 |
| ENSG00000267207.1 | RP11-264B14.1 | -2.1 | 1.1E-03 |
| ENSG00000267280.5 | TBX2-AS1 | -2.5 | 7.5E-26 |
| ENSG00000267519.3 | CTD-3252C9.4 | -1.5 | 5.2E-06 |
| ENSG00000267530.2 | AC006273.5 | -1.3 | 2.3E-03 |
| ENSG00000267607.1 | CTD-2369P2.8 | -1.8 | 1.3E-03 |
| ENSG00000267667.1 | RP11-136H19.1 | -1.5 | 9.6E-05 |
| ENSG00000267751.5 | AC009005.2 | 1.4 | 3.0E-03 |
| ENSG00000267774.2 | RP11-2N1.2 | -3.3 | 4.4E-16 |
| ENSG00000268836.1 | LA16c-OS12.2 | -1.2 | 6.2E-04 |
| ENSG00000270012.1 | LL0XNC01-7P3.1 | 1.1 | 3.7E-05 |
| ENSG00000270091.1 | RP11-78O7.2 | -1.5 | 1.5E-10 |
| ENSG00000270761.1 | RP11-385F7.1 | 1.7 | 1.6E-04 |
| ENSG00000271133.5 | CTA-293F17.1 | 1.5 | 1.4E-07 |
| ENSG00000271971.1 | CTD-2006H14.2 | 1.1 | 2.0E-05 |
| ENSG00000272081.1 | CTD-2376I4.2 | 1.2 | 1.9E-04 |
| ENSG00000272145.1 | NFYC-AS1 | 1.6 | 1.7E-11 |
| ENSG00000272512.1 | RP11-54O7.17 | -1.4 | 1.4E-05 |
| ENSG00000272588.1 | RP11-440L14.4 | 1.4 | 5.2E-09 |
| ENSG00000272620.1 | AFAP1-AS1 | 5.9 | 8.1E-42 |
| ENSG00000272734.1 | ADIRF-AS1 | -1.4 | 8.2E-11 |
| ENSG00000272783.1 | RP13-1016M1.2 | -3.0 | 1.3E-14 |
| ENSG00000272913.1 | RP11-440D17.3 | 1.7 | 4.1E-13 |
| ENSG00000273100.1 | RP11-302L19.3 | -2.6 | 7.8E-23 |
| ENSG00000273151.1 | RP11-449P15.2 | 1.0 | 1.1E-12 |
| ENSG00000273760.1 | CH17-360D5.3 | -3.2 | 5.3E-14 |
| ENSG00000274213.1 | RP11-670E13.6 | -1.6 | 1.6E-05 |
| ENSG00000274422.1 | LL22NC03-2H8.5 | 1.0 | 8.0E-03 |
| ENSG00000274653.1 | RP11-347C12.11 | 1.6 | 9.8E-08 |
| ENSG00000275665.1 | CTB-58E17.9 | 1.7 | 3.2E-05 |
| ENSG00000276170.4 | AC124789.1 | -2.7 | 6.2E-14 |
| ENSG00000276850.4 | CH17-360D5.2 | -3.2 | 8.4E-57 |
| ENSG00000277351.1 | RP11-325L12.6 | -1.6 | 4.3E-05 |
| ENSG00000277782.1 | RP11-775C24.5 | -1.6 | 7.8E-16 |
| ENSG00000278918.1 | CTD-2267D19.1 | -1.1 | 4.6E-06 |
| ENSG00000278934.1 | CTD-2006M22.2 | -1.2 | 1.3E-03 |
| ENSG00000279331.1 | RBM12B-AS1 | 2.0 | 1.6E-11 |
| ENSG00000279794.1 | CTD-2621I17.3 | 1.2 | 6.9E-06 |
| ENSG00000279805.1 | CTA-212A2.1 | -2.6 | 3.6E-23 |
| ENSG00000279882.1 | CTB-151G24.2 | 2.2 | 4.3E-08 |
| ENSG00000279940.1 | RP11-243M5.5 | -4.1 | 9.8E-27 |
| ENSG00000280018.3 | CH507-154B10.2 | 1.8 | 2.1E-14 |
| ENSG00000280099.1 | RP11-33E12.2 | -1.3 | 9.6E-05 |
| ENSG00000280145.3 | CH507-154B10.1 | 1.0 | 2.7E-06 |
| ENSG00000281406.1 | BLACAT1 | 3.3 | 6.8E-38 |
| ENSG00000258441.1 | LINC00641 | -1.2 | 1.8E-11 |
| ENSG00000258725.1 | PRC1-AS1 | 1.0 | 3.0E-06 |
| ENSG00000259330.1 | INAFM2 | -1.4 | 1.4E-19 |
| ENSG00000259366.1 | CTD-2647L4.4 | -1.6 | 1.7E-15 |
| ENSG00000259407.1 | RP11-158M2.3 | -1.3 | 7.5E-09 |
| ENSG00000259459.5 | RP11-321G12.1 | 1.6 | 1.2E-04 |
| ENSG00000259725.1 | CTD-3032H12.1 | 1.1 | 3.6E-03 |
| ENSG00000259986.1 | RP11-382A20.4 | 1.0 | 3.8E-09 |
| ENSG00000260025.1 | RP11-490M8.1 | -1.3 | 2.4E-07 |
| ENSG00000260032.1 | LINC00657 | -1.4 | 6.4E-11 |
| ENSG00000260260.1 | SNHG19 | 1.6 | 3.3E-09 |
| ENSG00000260822.1 | GS1-358P8.4 | -1.4 | 8.3E-08 |
| ENSG00000260912.1 | RP11-363E7.4 | -1.1 | 2.1E-06 |
| ENSG00000260975.1 | RP11-142G1.3 | 1.8 | 1.7E-10 |
| ENSG00000261054.1 | RP11-6O2.4 | -2.0 | 2.5E-05 |
| ENSG00000261207.1 | LA16c-361A3.3 | 1.6 | 7.7E-14 |
| ENSG00000261286.1 | RP11-517C16.2 | 1.0 | 1.3E-03 |
| ENSG00000261312.1 | AC002550.5 | 1.9 | 5.1E-06 |
| ENSG00000261353.1 | CTA-14H9.5 | -1.1 | 1.8E-06 |
| ENSG00000261359.2 | PYCARD-AS1 | 1.6 | 1.2E-04 |
| ENSG00000261369.1 | RP11-474B12.1 | 1.5 | 3.5E-15 |
| ENSG00000261396.1 | CTD-2012K14.2 | 1.1 | 8.9E-04 |
| ENSG00000261534.1 | RP11-244O19.1 | -1.4 | 6.4E-14 |
| ENSG00000261567.1 | RP11-680G10.1 | -2.1 | 1.2E-16 |
| ENSG00000261664.5 | TTC39A-AS1 | 1.6 | 2.7E-14 |
| ENSG00000261687.1 | RP11-473C18.3 | 1.2 | 8.3E-03 |
| ENSG00000262445.3 | CTD-2545H1.2 | 1.0 | 4.7E-13 |
| ENSG00000262772.1 | RP11-353N14.2 | 1.7 | 2.9E-08 |
| ENSG00000262903.1 | RP11-235E17.6 | 1.0 | 4.3E-06 |
| ENSG00000263400.6 | TMEM220-AS1 | -1.6 | 8.9E-14 |
| ENSG00000263603.1 | CTD-2349P21.5 | 1.3 | 1.7E-11 |
| ENSG00000263753.6 | LINC00667 | -1.1 | 1.5E-10 |
| ENSG00000264207.1 | RP11-196G18.23 | 1.4 | 5.1E-10 |
| ENSG00000264982.1 | RP11-344B2.2 | -2.2 | 6.6E-03 |
| ENSG00000265393.1 | CTD-2517M22.17 | 1.9 | 2.1E-10 |
| ENSG00000265688.1 | MAFG-AS1 | 1.5 | 1.6E-08 |
| ENSG00000266495.1 | RP11-17J14.2 | 1.5 | 1.7E-03 |
| ENSG00000266601.1 | RP11-6N17.3 | -1.6 | 1.7E-14 |
| ENSG00000266954.1 | RP11-701H16.4 | 2.1 | 1.0E-15 |
| ENSG00000267077.1 | RP11-127I20.5 | 1.7 | 5.6E-08 |
| ENSG00000267117.1 | AC010525.4 | 1.4 | 5.5E-18 |
| ENSG00000267194.1 | RP1-193H18.2 | -2.0 | 2.0E-15 |
| ENSG00000267270.5 | PARD6G-AS1 | 1.1 | 1.1E-05 |
| ENSG00000267282.1 | CTB-129P6.4 | 1.2 | 1.2E-05 |
| ENSG00000267296.2 | CEBPA-AS1 | -1.9 | 1.3E-12 |
| ENSG00000267348.2 | CTB-179K24.3 | 1.3 | 2.5E-11 |
| ENSG00000267577.1 | CTD-2587H24.5 | 1.1 | 2.5E-06 |
| ENSG00000268049.1 | CTD-2619J13.9 | 1.1 | 1.6E-09 |
| ENSG00000268189.2 | AC005785.2 | 1.5 | 2.0E-20 |
| ENSG00000268204.1 | CTD-3214H19.6 | 1.2 | 3.1E-05 |
| ENSG00000268230.5 | CTD-2619J13.8 | 1.7 | 2.0E-16 |
| ENSG00000268592.3 | RAET1E-AS1 | -1.3 | 9.6E-06 |
| ENSG00000268764.1 | CTC-450M9.1 | 1.7 | 3.5E-21 |
| ENSG00000268947.1 | AD000684.2 | 1.2 | 9.0E-07 |
| ENSG00000269194.1 | AC006942.4 | 1.1 | 3.0E-07 |
| ENSG00000269275.1 | CTD-2105E13.15 | 2.2 | 6.9E-08 |
| ENSG00000269653.1 | CTB-102L5.7 | 1.2 | 6.8E-13 |
| ENSG00000269915.1 | AP006621.9 | 1.3 | 5.3E-06 |
| ENSG00000269940.1 | RP11-73M18.7 | -1.1 | 3.0E-08 |
| ENSG00000270190.1 | RP11-803D5.4 | 2.2 | 5.2E-17 |
| ENSG00000270412.1 | RP11-92C4.6 | -2.9 | 2.3E-19 |
| ENSG00000270504.1 | RP11-420L9.5 | -1.3 | 6.1E-11 |
| ENSG00000270547.5 | LINC01235 | -1.1 | 6.2E-08 |
| ENSG00000270704.3 | RP11-701H24.5 | -1.1 | 5.8E-06 |
| ENSG00000270959.1 | LPP-AS2 | -1.4 | 2.6E-24 |
| ENSG00000271119.1 | CTD-2012J19.3 | 2.1 | 8.7E-20 |
| ENSG00000271452.1 | RP11-342K6.2 | -1.4 | 7.7E-12 |
| ENSG00000271730.1 | RP11-787I22.3 | -1.2 | 2.2E-05 |
| ENSG00000271781.1 | CTD-2589H19.6 | 1.0 | 1.2E-05 |
| ENSG00000271811.1 | RP1-79C4.4 | 1.5 | 1.8E-06 |
| ENSG00000271917.1 | RP11-506O24.2 | 2.4 | 6.5E-37 |
| ENSG00000272054.1 | RP11-423P10.2 | 1.5 | 2.4E-11 |
| ENSG00000272335.1 | RP11-53O19.3 | 1.4 | 2.2E-12 |
| ENSG00000272449.1 | RP3-395M20.12 | 1.2 | 1.4E-07 |
| ENSG00000272540.1 | XXbac-BPG252P9.9 | 1.5 | 9.2E-07 |
| ENSG00000272668.1 | RP11-190A12.8 | 2.1 | 2.7E-09 |
| ENSG00000272669.1 | RP3-508I15.21 | 1.1 | 1.6E-06 |
| ENSG00000272711.1 | RP11-259N19.1 | -1.2 | 7.9E-10 |
| ENSG00000272768.1 | RP4-673M15.1 | 1.0 | 3.3E-09 |
| ENSG00000272821.1 | CTA-384D8.36 | 1.2 | 1.5E-08 |
| ENSG00000272941.1 | RP11-134L10.1 | -1.1 | 6.7E-08 |
| ENSG00000272953.1 | RP11-1275H24.2 | 1.6 | 8.2E-17 |
| ENSG00000273261.1 | RP11-531F16.4 | -1.3 | 2.4E-09 |
| ENSG00000273319.1 | RP11-138A9.2 | 1.0 | 7.2E-06 |
| ENSG00000273466.1 | RP11-548H3.1 | 1.0 | 8.0E-05 |
| ENSG00000273619.1 | RP5-908M14.9 | 1.3 | 1.2E-11 |
| ENSG00000274021.1 | RP11-823E8.3 | -2.8 | 9.3E-16 |
| ENSG00000274364.1 | RP3-324O17.7 | 2.1 | 6.5E-17 |
| ENSG00000275223.1 | RP1-63M2.7 | 1.1 | 5.8E-12 |
| ENSG00000275484.1 | CTC-1337H24.4 | 1.7 | 1.3E-16 |
| ENSG00000275516.1 | RP11-353N14.7 | 1.7 | 2.3E-10 |
| ENSG00000276724.1 | RP11-1000B6.7 | -1.3 | 6.3E-07 |
| ENSG00000277382.1 | RP11-318A15.8 | -1.3 | 5.0E-06 |
| ENSG00000277496.1 | RP11-93B14.9 | -1.5 | 1.8E-08 |
| ENSG00000277744.1 | CTC-435M10.12 | 1.0 | 6.5E-08 |
| ENSG00000278900.1 | RP11-1250I15.2 | 1.9 | 8.3E-16 |
| ENSG00000278921.2 | EPB41L4A-AS2 | -1.0 | 5.3E-05 |
| ENSG00000279182.1 | XX-C00717C00720L.1 | 1.8 | 2.6E-03 |
| ENSG00000279191.1 | RP11-803D5.1 | 1.3 | 5.8E-15 |
| ENSG00000279192.1 | PWAR5 | -1.7 | 4.9E-13 |
| ENSG00000279198.1 | CTD-2231E14.2 | 1.1 | 5.2E-07 |
| ENSG00000279296.1 | RP11-609D21.3 | 1.3 | 7.9E-04 |
| ENSG00000279536.1 | RP11-445N20.2 | 1.7 | 2.6E-08 |
| ENSG00000279568.1 | RP11-20I23.5 | 1.3 | 2.5E-11 |
| ENSG00000279611.1 | CTD-2619J13.5 | 1.3 | 8.3E-10 |
| ENSG00000279700.1 | RP13-554M15.2 | 1.3 | 1.7E-11 |
| ENSG00000279930.1 | LA16c-312E8.4 | 1.4 | 8.2E-08 |
| ENSG00000280020.1 | CTD-2095E4.4 | 1.4 | 6.8E-09 |
| ENSG00000280119.1 | AC093642.1 | 2.7 | 8.9E-11 |
| ENSG00000280206.1 | CTB-193M12.5 | 1.1 | 3.4E-07 |
| ENSG00000280335.1 | RP11-15F12.6 | 2.6 | 2.2E-11 |
| ENSG00000280388.1 | RP11-229D13.3 | 1.1 | 1.7E-11 |
| ENSG00000280832.1 | ST3GAL4-AS1 | 1.2 | 6.5E-15 |
| ENSG00000281501.1 | SEPSECS-AS1 | 1.7 | 1.7E-11 |

**Supplementary Table S3: Functional DELs only altered in one cancer type derived from PubMed**

| blader | | pro | | ADC | | EBC | |
|---|---|---|---|---|---|---|---|
| **gene id** | **gene name** | **gene id** | **gene name** | **gene id** | **gene name** | **gene id** | **gene name** |
| ENSG00000153363.12 | LINC00467 | ENSG00000198590.11 | C3orf35 | ENSG00000196167.9 | COLCA1 | ENSG00000130600.15 | H19 |
| ENSG00000166770.10 | ZNF667-AS1 | ENSG00000205861.11 | C1QTNF9B-AS1 | ENSG00000204623.8 | ZNRD1-AS1 | ENSG00000222041.10 | LINC00152 |
| ENSG00000177410.12 | ZFAS1 | ENSG00000224259.5 | LINC01133 | ENSG00000233237.6 | LINC00472 | ENSG00000225756.1 | DBH-AS1 |
| ENSG00000180139.11 | ACTA2-AS1 | ENSG00000225937.1 | PCA3 | ENSG00000234380.1 | LINC01426 | ENSG00000230061.2 | TRPM2-AS |
| ENSG00000214049.6 | UCA1 | ENSG00000231806.2 | PCAT7 | ENSG00000236830.6 | CBR3-AS1 | ENSG00000237361.2 | TUSC8 |
| ENSG00000215386.10 | MIR99AHG | ENSG00000245532.5 | NEAT1 | ENSG00000238057.8 | ZEB2-AS1 | ENSG00000253552.7 | HOXA-AS2 |
| ENSG00000215458.8 | AATBC | ENSG00000246763.6 | RGMB-AS1 | ENSG00000245694.8 | CRNDE | ENSG00000270959.1 | LPP-AS2 |
| ENSG00000227372.10 | TP73-AS1 | ENSG00000253438.2 | PCAT1 | ENSG00000272620.1 | AFAP1-AS1 | | |
| ENSG00000231607.8 | DLEU2 | ENSG00000259641.5 | PCAT29 | ENSG00000281406.1 | BLACAT1 | | |
| ENSG00000231721.6 | LINC-PINT | ENSG00000260896.5 | PRCAT47 | | | | |
| ENSG00000231764.8 | DLX6-AS1 | ENSG00000265369.3 | PCAT18 | | | | |
| ENSG00000234771.3 | SLC25A25-AS1 | ENSG00000280623.1 | PCAT14 | | | | |
| ENSG00000237125.8 | HAND2-AS1 | ENSG00000280927.1 | CTBP1-AS | | | | |
| ENSG00000249082.2 | C5orf66-AS1 | | | | | | |
| ENSG00000255090.5 | MIR100HG | | | | | | |
| ENSG00000255717.6 | SNHG1 | | | | | | |

**Supplementary Table S4: log2FC (fold change) and FDR of onco-lncRNAs in each cancer type and their corresponding modules in the co-expression network**

| Onco-lncRNA and correponding module | | | BLC | | PRC | | ADC | | EBC | |
|---|---|---|---|---|---|---|---|---|---|---|
| gene_id | gene_name | Module | log2FC | FDR | log2FC | FDR | log2FC | FDR | log2FC | FDR |
| ENSG00000228434.1 | AC004951.6 | magenta | - | - | 1.2 | 6.5E-04 | - | - | 1.4 | 8.1E-19 |
| ENSG00000214719.11 | AC005562.1 | grey | - | - | 1.4 | 5.4E-03 | 1.4 | 9.0E-03 | - | - |
| ENSG00000279203.1 | AC005785.5 | brown | 1.3 | 8.9E-03 | 1.6 | 2.2E-07 | - | - | 1.5 | 5.5E-20 |
| ENSG00000279716.1 | AC006128.2 | blue | 1.5 | 2.2E-04 | - | - | - | - | 1.6 | 1.7E-08 |
| ENSG00000233251.7 | AC007743.1 | brown | 1.1 | 1.1E-03 | 1.7 | 6.1E-10 | - | - | 1.1 | 7.5E-07 |
| ENSG00000236255.1 | AC009404.2 | blue | 1.5 | 6.6E-05 | 1.5 | 2.1E-09 | - | - | - | - |
| ENSG00000226266.6 | AC009961.3 | blue | - | - | 1.2 | 6.3E-07 | - | - | 1.3 | 1.7E-19 |
| ENSG00000236039.1 | AC019117.2 | midnightblue | 2.2 | 5.5E-03 | - | - | 1.9 | 3.2E-06 | - | - |
| ENSG00000259605.3 | AC074212.5 | blue | - | - | 1.4 | 5.9E-07 | - | - | 1.3 | 6.2E-07 |
| ENSG00000235493.1 | AC092415.1 | brown | 1.7 | 2.6E-03 | 1.9 | 2.3E-09 | - | - | 1.2 | 1.3E-07 |
| ENSG00000232164.1 | AC092669.3 | brown | - | - | 1.4 | 3.4E-05 | 1.3 | 2.1E-17 | 1.8 | 6.1E-12 |
| ENSG00000237940.3 | AC093642.3 | blue | - | - | 1.4 | 1.3E-03 | - | - | 1.5 | 1.0E-11 |
| ENSG00000244567.1 | AC096772.6 | blue | - | - | - | - | -1.1 | 7.8E-13 | -1.7 | 2.1E-19 |
| ENSG00000233766.7 | AC098617.1 | greenyellow | -3.6 | 8.2E-05 | 2.5 | 2.4E-05 | - | - | - | - |
| ENSG00000278000.1 | AC139100.4 | brown | - | - | 1.6 | 2.4E-05 | - | - | 1.5 | 3.7E-10 |
| ENSG00000203635.2 | AC144450.2 | blue | - | - | 2.3 | 3.1E-10 | - | - | 1.4 | 6.5E-22 |
| ENSG00000261888.1 | AC144831.1 | yellow | - | - | - | - | -1.9 | 3.5E-19 | -1.6 | 1.1E-11 |
| ENSG00000274370.1 | AC144831.3 | yellow | - | - | - | - | -1.8 | 5.9E-17 | -2.0 | 8.4E-14 |
| ENSG00000230606.10 | AC159540.1 | grey | - | - | 2.0 | 1.2E-11 | 1.2 | 3.1E-03 | 2.0 | 2.3E-15 |
| ENSG00000278766.2 | AC159540.2 | grey | - | - | - | - | 1.2 | 9.5E-04 | 2.4 | 2.0E-20 |
| ENSG00000241158.5 | ADAMTS9-AS1 | red | -6.6 | 8.9E-33 | - | - | -2.6 | 4.0E-22 | -2.6 | 1.3E-18 |
| ENSG00000241684.5 | ADAMTS9-AS2 | red | -5.0 | 3.1E-35 | - | - | -1.5 | 6.3E-09 | -1.5 | 1.1E-08 |
| ENSG00000280429.1 | AF001548.3 | red | -6.3 | 2.0E-19 | 1.3 | 5.1E-03 | - | - | - | - |
| ENSG00000271880.1 | AGAP11 | yellow | - | - | - | - | -2.7 | 6.3E-20 | -1.2 | 2.1E-04 |
| ENSG00000246889.2 | AP000487.5 | blue | 1.6 | 9.3E-04 | - | - | - | - | 1.0 | 1.6E-09 |
| ENSG00000280143.1 | AP000892.6 | red | -5.5 | 5.1E-23 | - | - | - | - | -1.9 | 4.4E-11 |
| ENSG00000271816.1 | BMS1P4 | brown | 1.4 | 5.2E-04 | 1.4 | 1.2E-07 | - | - | - | - |
| ENSG00000227051.5 | C14orf132 | green | -2.2 | 1.9E-04 | - | - | -2.2 | 1.1E-31 | - | - |
| ENSG00000225670.4 | CADM3-AS1 | cyan | -5.0 | 8.2E-18 | - | - | -1.5 | 1.2E-08 | - | - |
| ENSG00000272720.1 | CTA-228A9.3 | brown | 1.6 | 3.7E-05 | 1.7 | 2.0E-08 | - | - | - | - |
| ENSG00000275719.1 | CTB-147N14.6 | yellow | - | - | -1.2 | 4.8E-03 | - | - | -1.7 | 5.3E-09 |
| ENSG00000267383.6 | CTC-260E6.6 | lightgreen | 2.0 | 2.1E-04 | 1.1 | 4.6E-04 | - | - | 1.3 | 9.5E-10 |
| ENSG00000267405.1 | CTC-296K1.4 | red | -6.3 | 1.5E-21 | -1.3 | 1.0E-04 | - | - | - | - |
| ENSG00000233937.6 | CTC-338M12.4 | blue | 1.6 | 3.0E-04 | - | - | 1.0 | 6.0E-04 | - | - |
| ENSG00000241956.9 | CTC-340A15.2 | brown | - | - | 1.6 | 4.6E-06 | - | - | 1.5 | 9.5E-09 |
| ENSG00000279759.1 | CTC-425O23.5 | brown | 1.9 | 1.6E-04 | 1.4 | 1.8E-06 | - | - | - | - |
| ENSG00000272367.1 | CTC-428H11.2 | cyan | -1.8 | 5.7E-03 | 1.5 | 5.5E-04 | - | - | - | - |
| ENSG00000262601.1 | CTC-786C10.1 | yellow | - | - | - | - | -1.5 | 4.4E-06 | -3.2 | 8.4E-40 |
| ENSG00000280332.1 | CTD-2013N17.6 | blue | 1.2 | 3.8E-03 | 1.0 | 1.3E-03 | - | - | - | - |
| ENSG00000279425.1 | CTD-2017D11.2 | magenta | 1.2 | 4.1E-03 | 1.1 | 8.7E-05 | - | - | - | - |
| ENSG00000274383.1 | CTD-2017F17.2 | brown | - | - | 2.1 | 5.9E-04 | - | - | 2.1 | 3.1E-10 |
| ENSG00000262979.1 | CTD-2047H16.2 | brown | 2.0 | 5.1E-05 | 1.1 | 9.3E-03 | 1.2 | 4.9E-06 | 3.6 | 7.5E-23 |
| ENSG00000260751.2 | CTD-2196E14.6 | brown | 1.4 | 1.4E-03 | - | - | 1.1 | 2.3E-07 | - | - |
| ENSG00000270933.1 | CTD-2227E11.1 | grey | - | - | 1.5 | 4.1E-04 | 3.6 | 4.5E-14 | - | - |
| ENSG00000230698.1 | CTD-2330K9.2 | grey | 3.6 | 8.0E-05 | - | - | - | - | 3.0 | 8.3E-13 |
| ENSG00000276250.1 | CTD-2349P21.12 | magenta | 1.5 | 4.9E-03 | 1.2 | 8.0E-03 | - | - | - | - |
| ENSG00000255182.2 | CTD-2517M22.14 | blue | 1.4 | 4.3E-04 | 1.5 | 9.9E-05 | 1.1 | 1.3E-06 | 1.5 | 2.1E-13 |
| ENSG00000267523.1 | CTD-2537I9.12 | brown | 1.2 | 6.4E-03 | 1.4 | 2.1E-03 | - | - | 1.1 | 1.2E-09 |
| ENSG00000274925.1 | CTD-2547G23.4 | brown | 1.1 | 6.6E-03 | 1.3 | 3.0E-05 | 1.5 | 1.0E-10 | - | - |
| ENSG00000279117.1 | CTD-2562J17.6 | greenyellow | -2.1 | 2.5E-04 | - | - | -2.5 | 3.5E-28 | -1.3 | 1.6E-05 |
| ENSG00000279789.1 | CTD-2574D22.3 | brown | 2.0 | 7.9E-07 | 1.4 | 5.4E-06 | 1.4 | 1.7E-08 | 1.1 | 3.9E-15 |
| ENSG00000269867.1 | CTD-2583A14.8 | turquoise | 1.8 | 1.8E-08 | - | - | - | - | 1.6 | 2.0E-18 |
| ENSG00000269873.1 | CTD-2587H19.3 | magenta | 1.7 | 4.4E-03 | 1.5 | 6.2E-03 | - | - | - | - |
| ENSG00000268201.1 | CTD-3138B18.6 | brown | - | - | 1.2 | 7.4E-04 | - | - | 1.1 | 4.9E-16 |
| ENSG00000265787.1 | CYP4F35P | magenta | 3.5 | 6.3E-08 | 2.5 | 2.4E-09 | - | - | - | - |
| ENSG00000231890.7 | DARS-AS1 | brown | 1.3 | 1.1E-04 | 1.0 | 8.7E-06 | - | - | - | - |
| ENSG00000235706.7 | DICER1-AS1 | brown | 1.0 | 5.2E-03 | 1.4 | 8.8E-06 | - | - | - | - |
| ENSG00000258498.6 | DIO3OS | cyan | -4.8 | 1.1E-28 | - | - | -1.6 | 1.1E-04 | - | - |
| ENSG00000245750.7 | DRAIC | pink | - | - | 1.9 | 1.2E-08 | 2.4 | 1.2E-08 | 1.1 | 6.7E-03 |
| ENSG00000206195.10 | DUXAP8 | grey | - | - | 1.3 | 1.5E-04 | 1.9 | 2.0E-04 | - | - |
| ENSG00000203499.10 | FAM83H-AS1 | blue | 1.9 | 1.2E-04 | - | - | 2.6 | 4.2E-16 | - | - |
| ENSG00000260852.1 | FBXL19-AS1 | blue | 2.0 | 1.4E-08 | 1.6 | 9.2E-10 | - | - | 1.1 | 5.6E-14 |
| ENSG00000268388.5 | FENDRR | cyan | -4.2 | 5.2E-33 | - | - | -4.7 | 3.6E-31 | - | - |
| ENSG00000272143.1 | FGF14-AS2 | yellow | -1.6 | 1.6E-03 | -1.5 | 3.5E-04 | -1.0 | 7.2E-07 | -1.9 | 4.1E-10 |
| ENSG00000250208.6 | FZD10-AS1 | turquoise | -2.9 | 4.5E-09 | - | - | -1.3 | 7.5E-08 | - | - |
| ENSG00000248890.1 | HHIP-AS1 | cyan | -3.4 | 1.7E-09 | - | - | -1.1 | 1.6E-03 | - | - |
| ENSG00000235313.1 | HM13-IT1 | salmon | - | - | 1.4 | 1.4E-05 | - | - | 1.2 | 1.9E-14 |
| ENSG00000234705.1 | HMGA1P4 | blue | 2.6 | 2.4E-06 | 1.1 | 3.0E-04 | - | - | 2.0 | 1.3E-12 |
| ENSG00000233101.10 | HOXB-AS3 | magenta | 2.6 | 2.7E-04 | - | - | 1.8 | 2.0E-03 | - | - |
| ENSG00000227392.1 | HPN-AS1 | brown | - | - | 2.4 | 5.7E-07 | - | - | 1.8 | 8.5E-17 |
| ENSG00000236778.7 | INTS6-AS1 | brown | - | - | 1.4 | 6.2E-07 | - | - | 1.2 | 3.4E-08 |

| | | | | | | | | | | |
|---|---|---|---|---|---|---|---|---|---|---|
| ENSG00000261505.1 | LA16c-358B7.3 | blue | - | - | 1.3 | 1.5E-03 | - | - | 1.2 | 6.4E-13 |
| ENSG00000226696.5 | LENG8-AS1 | blue | - | - | 1.1 | 8.2E-04 | 1.0 | 5.5E-06 | - | - |
| ENSG00000225194.2 | LINC00092 | turquoise | - | - | -2.0 | 1.2E-06 | -2.1 | 1.4E-12 | - | - |
| ENSG00000248360.7 | LINC00504 | brown | 1.1 | 9.8E-03 | 1.7 | 2.5E-10 | - | - | 1.3 | 2.0E-08 |
| ENSG00000281392.1 | LINC00506 | brown | 1.2 | 7.9E-03 | 1.8 | 1.0E-09 | - | - | 1.8 | 4.2E-11 |
| ENSG00000261824.6 | LINC00662 | brown | - | - | 1.4 | 6.8E-10 | - | - | 1.3 | 4.2E-10 |
| ENSG00000232677.6 | LINC00665 | turquoise | 1.2 | 8.6E-04 | - | - | 1.1 | 4.5E-03 | - | - |
| ENSG00000258647.5 | LINC00930 | magenta | - | - | 2.1 | 7.9E-08 | -1.5 | 8.6E-06 | - | - |
| ENSG00000281657.2 | LINC00976 | brown | - | - | 1.7 | 1.0E-09 | - | - | 1.2 | 6.0E-07 |
| ENSG00000224081.7 | LINC01057 | magenta | 1.4 | 8.1E-04 | 1.5 | 1.6E-06 | - | - | - | - |
| ENSG00000212694.8 | LINC01089 | blue | 1.3 | 6.0E-03 | 1.6 | 2.2E-09 | - | - | - | - |
| ENSG00000267272.5 | LINC01140 | yellow | -2.0 | 7.9E-03 | - | - | - | - | -2.5 | 9.5E-18 |
| ENSG00000281404.1 | LINC01176 | brown | 1.6 | 2.6E-07 | 1.1 | 1.8E-04 | - | - | 1.7 | 1.3E-17 |
| ENSG00000280734.2 | LINC01232 | brown | 2.0 | 1.6E-08 | 1.2 | 1.6E-08 | 1.5 | 1.7E-16 | - | - |
| ENSG00000227953.6 | LINC01341 | magenta | 1.8 | 2.1E-04 | 1.7 | 1.2E-05 | - | - | 1.8 | 1.9E-13 |
| ENSG00000261326.2 | LINC01355 | magenta | 1.2 | 1.3E-03 | 1.5 | 1.0E-09 | 1.5 | 6.8E-27 | - | - |
| ENSG00000238113.6 | LINC01410 | brown | 1.5 | 2.4E-03 | - | - | - | - | 1.7 | 1.4E-11 |
| ENSG00000262468.5 | LINC01569 | blue | - | - | - | - | 1.8 | 5.5E-18 | 1.3 | 1.3E-17 |
| ENSG00000229619.3 | MBNL1-AS1 | red | -4.6 | 1.8E-13 | - | - | -1.3 | 1.8E-09 | -1.9 | 1.5E-22 |
| ENSG00000214548.14 | MEG3 | grey | -4.4 | 7.5E-17 | - | - | 3.0 | 2.9E-07 | - | - |
| ENSG00000249669.7 | MIR143HG | red | -4.3 | 3.5E-15 | - | - | - | - | -1.3 | 2.5E-08 |
| ENSG00000230937.9 | MIR205HG | midnightblue | - | - | -1.4 | 4.5E-03 | 3.7 | 7.3E-13 | - | - |
| ENSG00000247095.2 | MIR210HG | blue | 1.6 | 2.1E-03 | - | - | 1.2 | 1.7E-03 | 1.1 | 4.5E-11 |
| ENSG00000186594.12 | MIR22HG | cyan | -2.3 | 8.5E-07 | - | - | -1.9 | 9.9E-30 | -2.0 | 7.6E-15 |
| ENSG00000267532.3 | MIR497HG | red | -3.3 | 4.4E-04 | - | - | - | - | -1.7 | 5.4E-13 |
| ENSG00000223749.7 | MIR503HG | blue | - | - | - | - | 1.8 | 3.2E-07 | 1.9 | 2.0E-06 |
| ENSG00000261971.6 | MMP25-AS1 | blue | - | - | 1.1 | 1.0E-03 | - | - | 1.0 | 1.2E-03 |
| ENSG00000247809.7 | NR2F2-AS1 | red | -1.6 | 2.8E-04 | - | - | -1.2 | 4.6E-10 | - | - |
| ENSG00000238045.9 | PAGR1 | turquoise | - | - | - | - | 1.3 | 5.2E-06 | 1.1 | 2.8E-17 |
| ENSG00000267107.6 | PCAT19 | cyan | -2.9 | 4.3E-15 | - | - | -2.5 | 2.8E-42 | - | - |
| ENSG00000228288.6 | PCAT6 | blue | 1.3 | 3.7E-04 | - | - | 2.3 | 2.4E-20 | 1.9 | 1.7E-15 |
| ENSG00000196696.12 | PDXDC2P | blue | - | - | 1.6 | 3.9E-06 | - | - | 1.2 | 3.5E-14 |
| ENSG00000224958.5 | PGM5-AS1 | red | -8.9 | 4.4E-35 | -1.1 | 6.8E-04 | - | - | - | - |
| ENSG00000260804.3 | PKI55 | black | - | - | - | - | -1.2 | 2.4E-13 | -1.7 | 4.7E-14 |
| ENSG00000249859.7 | PVT1 | blue | 1.8 | 7.8E-04 | 1.6 | 1.5E-09 | 2.0 | 1.7E-09 | 1.5 | 1.3E-16 |
| ENSG00000223525.1 | RABGAP1L-IT1 | brown | 1.2 | 5.4E-04 | - | - | - | - | 1.4 | 3.8E-09 |
| ENSG00000262877.4 | RP11-1055B8.4 | blue | - | - | 1.7 | 6.3E-06 | - | - | 1.0 | 3.8E-04 |
| ENSG00000280046.1 | RP11-1099M24.6 | brown | 1.5 | 1.3E-04 | 1.2 | 5.6E-06 | - | - | - | - |
| ENSG00000259884.1 | RP11-1100L3.8 | cyan | - | - | 1.5 | 1.4E-03 | -1.6 | 4.4E-05 | 1.5 | 1.5E-03 |
| ENSG00000227388.2 | RP11-112J3.16 | turquoise | 1.5 | 1.3E-03 | 1.1 | 1.9E-03 | - | - | - | - |
| ENSG00000271771.1 | RP11-1250I15.3 | turquoise | 1.8 | 8.9E-03 | 1.8 | 3.9E-05 | - | - | 1.1 | 1.7E-11 |
| ENSG00000274605.1 | RP11-12G12.7 | pink | 1.3 | 9.9E-03 | - | - | 1.9 | 6.2E-17 | - | - |
| ENSG00000223396.2 | RP11-134G8.5 | brown | 1.6 | 3.6E-03 | - | - | 1.3 | 7.5E-07 | - | - |
| ENSG00000280234.1 | RP11-145P16.2 | brown | - | - | 1.5 | 3.4E-04 | - | - | 1.7 | 2.3E-13 |
| ENSG00000272100.1 | RP11-155O18.6 | salmon | 2.3 | 8.4E-04 | - | - | - | - | 1.1 | 4.5E-06 |
| ENSG00000260807.6 | RP11-161M6.2 | pink | - | - | 1.1 | 2.7E-03 | - | - | -1.2 | 2.2E-06 |
| ENSG00000261560.1 | RP11-166B2.3 | magenta | - | - | 1.0 | 7.7E-03 | - | - | 1.0 | 2.4E-12 |
| ENSG00000255248.6 | RP11-166D19.1 | red | -5.5 | 9.1E-51 | - | - | - | - | -1.1 | 3.1E-07 |
| ENSG00000261324.2 | RP11-174G6.5 | pink | 1.3 | 1.6E-04 | 1.5 | 1.6E-05 | - | - | - | - |
| ENSG00000264608.1 | RP11-192H23.8 | lightgreen | 2.5 | 2.8E-07 | 2.0 | 2.8E-09 | - | - | 1.7 | 3.1E-14 |
| ENSG00000261716.1 | RP11-196G18.22 | brown | 1.1 | 6.7E-03 | 1.4 | 7.6E-09 | 1.4 | 1.2E-10 | 1.3 | 1.4E-15 |
| ENSG00000260436.1 | RP11-20I23.7 | brown | - | - | 1.4 | 1.1E-04 | - | - | 1.3 | 4.3E-15 |
| ENSG00000279281.1 | RP11-226M10.3 | magenta | 1.5 | 4.5E-03 | 1.5 | 1.4E-05 | - | - | 1.3 | 5.2E-23 |
| ENSG00000249731.1 | RP11-259O2.3 | brown | - | - | 1.3 | 4.0E-05 | 1.6 | 7.5E-22 | 1.6 | 2.1E-09 |
| ENSG00000260853.1 | RP11-264B17.2 | magenta | 1.4 | 2.4E-03 | 1.1 | 2.1E-03 | - | - | 1.0 | 5.8E-13 |
| ENSG00000272789.1 | RP11-286H15.1 | greenyellow | -4.0 | 1.6E-09 | - | - | -4.3 | 1.2E-30 | - | - |
| ENSG00000277639.1 | RP11-295M3.4 | grey | - | - | -2.6 | 2.9E-09 | - | - | -2.3 | 8.1E-11 |
| ENSG00000273474.1 | RP11-295P9.12 | magenta | 2.2 | 1.5E-04 | 1.6 | 1.6E-05 | - | - | - | - |
| ENSG00000270953.1 | RP11-2E11.9 | salmon | 1.0 | 6.7E-03 | 1.2 | 6.6E-03 | - | - | -1.1 | 6.3E-05 |
| ENSG00000261663.1 | RP11-304L19.11 | brown | 1.2 | 7.3E-03 | - | - | - | - | 1.1 | 2.3E-14 |
| ENSG00000232093.1 | RP11-307C12.11 | blue | 2.1 | 1.3E-03 | - | - | - | - | 1.4 | 6.6E-10 |
| ENSG00000262580.5 | RP11-334C17.5 | brown | - | - | 1.3 | 5.7E-06 | - | - | 1.2 | 2.7E-11 |
| ENSG00000224307.1 | RP11-344B5.2 | green | -3.1 | 3.9E-06 | - | - | - | - | -1.1 | 5.5E-08 |
| ENSG00000259380.5 | RP11-346D14.1 | magenta | 1.2 | 9.6E-03 | 1.8 | 4.8E-10 | 1.1 | 7.7E-14 | 1.7 | 1.4E-10 |
| ENSG00000260798.1 | RP11-354M1.2 | blue | 2.0 | 6.3E-03 | - | - | - | - | 1.4 | 1.4E-07 |
| ENSG00000241288.7 | RP11-379B18.5 | brown | - | - | 1.2 | 1.8E-05 | - | - | 1.1 | 1.9E-09 |
| ENSG00000279417.1 | RP11-382B18.1 | magenta | - | - | 1.5 | 5.3E-07 | - | - | 1.7 | 1.1E-10 |
| ENSG00000266538.1 | RP11-385D13.3 | brown | - | - | 1.6 | 4.1E-04 | 1.6 | 1.1E-05 | 1.4 | 2.4E-09 |
| ENSG00000261269.1 | RP11-389C8.2 | yellow | - | - | - | - | -1.4 | 3.4E-10 | -2.1 | 5.8E-13 |
| ENSG00000276434.1 | RP11-391H12.8 | lightgreen | 2.3 | 1.3E-04 | 1.1 | 2.6E-04 | - | - | - | - |
| ENSG00000269936.3 | RP11-394O4.5 | red | -4.8 | 3.7E-19 | 1.1 | 9.8E-03 | - | - | -1.3 | 3.3E-06 |
| ENSG00000261685.2 | RP11-401P9.4 | green | -2.6 | 8.1E-13 | - | - | -2.4 | 8.9E-48 | - | - |
| ENSG00000279283.1 | RP11-417L19.5 | brown | - | - | 1.9 | 1.5E-06 | - | - | 1.6 | 4.3E-18 |
| ENSG00000251432.6 | RP11-420A23.1 | tan | 1.5 | 3.7E-03 | 1.5 | 6.8E-05 | - | - | - | - |
| ENSG00000264895.1 | RP11-421E14.2 | brown | 2.1 | 3.5E-05 | 1.5 | 2.3E-04 | - | - | 2.1 | 2.0E-14 |
| ENSG00000255864.5 | RP11-444D3.1 | yellow | - | - | - | - | -1.4 | 2.0E-05 | -1.3 | 3.7E-04 |

| | | | | | | | | | | |
|---|---|---|---|---|---|---|---|---|---|---|
| ENSG00000224799.1 | RP11-445O16.3 | brown | 1.4 | 2.3E-03 | 1.4 | 6.1E-05 | - | - | - | - |
| ENSG00000260265.1 | RP11-44F21.5 | midnightblue | 1.6 | 8.2E-03 | -2.1 | 5.6E-14 | - | - | - | - |
| ENSG00000273142.1 | RP11-458F8.4 | brown | 1.1 | 6.0E-03 | 1.4 | 1.0E-05 | - | - | 1.3 | 1.6E-10 |
| ENSG00000263235.1 | RP11-461A8.4 | brown | 1.9 | 7.5E-05 | 1.5 | 1.7E-05 | - | - | 1.7 | 5.1E-14 |
| ENSG00000272141.1 | RP11-465B22.8 | blue | 2.6 | 1.1E-06 | - | - | 1.6 | 6.0E-09 | 2.0 | 1.9E-16 |
| ENSG00000234678.1 | RP11-465N4.4 | magenta | 1.8 | 4.6E-05 | 1.0 | 7.8E-04 | - | - | 1.3 | 4.5E-17 |
| ENSG00000273478.1 | RP11-465N4.5 | blue | 1.9 | 2.0E-05 | - | - | - | - | 1.2 | 2.1E-16 |
| ENSG00000205890.3 | RP11-473M20.5 | blue | 1.6 | 7.7E-04 | - | - | 1.6 | 3.7E-10 | 1.2 | 4.2E-06 |
| ENSG00000262370.5 | RP11-473M20.9 | green | -2.0 | 1.0E-04 | - | - | -1.4 | 5.8E-11 | - | - |
| ENSG00000260261.1 | RP11-480A16.1 | blue | 1.8 | 1.1E-07 | - | - | 1.6 | 1.7E-19 | - | - |
| ENSG00000279089.1 | RP11-481C4.2 | magenta | 1.6 | 1.4E-06 | 1.1 | 1.7E-05 | - | - | - | - |
| ENSG00000251379.1 | RP11-484O2.1 | brown | 1.4 | 3.6E-03 | 2.1 | 1.5E-11 | - | - | 1.5 | 2.6E-09 |
| ENSG00000273302.1 | RP11-493E12.2 | brown | - | - | 1.3 | 7.4E-06 | - | - | 1.2 | 1.0E-10 |
| ENSG00000273308.1 | RP11-496H1.2 | blue | - | - | - | - | 1.6 | 7.3E-09 | 1.4 | 8.9E-12 |
| ENSG00000254815.5 | RP11-496I9.1 | blue | 1.9 | 3.0E-04 | 1.3 | 1.0E-04 | - | - | 2.4 | 1.3E-13 |
| ENSG00000278876.1 | RP11-498C9.4 | brown | 1.9 | 1.6E-03 | 1.4 | 4.6E-04 | 1.6 | 2.8E-07 | 1.6 | 7.0E-12 |
| ENSG00000240291.1 | RP11-499P20.2 | magenta | - | - | 1.3 | 2.4E-05 | - | - | 1.2 | 9.0E-09 |
| ENSG00000274849.1 | RP11-49I11.4 | salmon | 2.4 | 1.2E-08 | - | - | - | - | 1.3 | 3.4E-08 |
| ENSG00000253972.5 | RP11-4K16.2 | turquoise | 2.2 | 1.9E-04 | - | - | 1.3 | 6.8E-05 | 1.5 | 9.7E-14 |
| ENSG00000272645.2 | RP11-504P24.8 | brown | 1.2 | 1.6E-05 | 1.1 | 4.1E-06 | - | - | 1.1 | 3.2E-11 |
| ENSG00000249007.1 | RP11-510N19.5 | magenta | 2.8 | 3.9E-08 | 1.3 | 3.5E-03 | 2.2 | 1.8E-05 | 2.4 | 1.2E-10 |
| ENSG00000272183.1 | RP11-523H20.3 | blue | 2.8 | 6.6E-06 | - | - | 2.0 | 1.2E-11 | 1.7 | 5.3E-12 |
| ENSG00000261295.1 | RP11-524D16__A.3 | yellow | - | - | - | - | 1.1 | 2.8E-07 | -1.8 | 4.8E-08 |
| ENSG00000260461.1 | RP11-541N10.3 | green | - | - | - | - | -1.1 | 1.5E-08 | -1.3 | 8.9E-11 |
| ENSG00000235652.7 | RP11-545I5.3 | brown | - | - | 1.5 | 3.6E-08 | - | - | 1.0 | 4.3E-06 |
| ENSG00000277566.1 | RP11-545P7.9 | grey60 | 1.7 | 9.1E-03 | - | - | - | - | 1.1 | 1.5E-07 |
| ENSG00000279706.1 | RP11-561O23.9 | magenta | 2.0 | 6.8E-04 | - | - | - | - | 1.3 | 4.6E-14 |
| ENSG00000275764.1 | RP11-582E3.6 | brown | - | - | 1.3 | 1.1E-07 | - | - | 1.2 | 2.7E-08 |
| ENSG00000260244.1 | RP11-588K22.2 | pink | -2.0 | 4.1E-04 | - | - | - | - | -1.6 | 7.8E-11 |
| ENSG00000279806.1 | RP11-58O9.2 | magenta | 2.7 | 5.0E-05 | -1.8 | 1.4E-03 | - | - | -1.4 | 1.6E-06 |
| ENSG00000248429.5 | RP11-597D13.9 | black | - | - | 1.2 | 2.1E-05 | 1.5 | 9.3E-06 | - | - |
| ENSG00000279662.1 | RP11-609N14.4 | brown | 1.5 | 9.7E-05 | - | - | -1.4 | 7.8E-09 | 1.4 | 1.2E-09 |
| ENSG00000273691.1 | RP11-617F23.2 | brown | 1.3 | 9.6E-03 | - | - | - | - | 1.9 | 1.5E-20 |
| ENSG00000279912.1 | RP11-624A21.1 | magenta | - | - | 1.4 | 1.1E-06 | - | - | 1.7 | 7.0E-11 |
| ENSG00000259345.5 | RP11-624L4.1 | brown | 1.3 | 1.3E-05 | - | - | - | - | 1.6 | 3.4E-13 |
| ENSG00000255191.1 | RP11-626H12.1 | black | - | - | - | - | -2.4 | 6.2E-11 | -2.1 | 6.6E-12 |
| ENSG00000275180.1 | RP11-631N16.4 | black | - | - | 1.5 | 6.4E-06 | 1.3 | 1.1E-05 | - | - |
| ENSG00000272556.1 | RP11-638I8.1 | brown | 1.3 | 2.4E-03 | - | - | - | - | 1.1 | 1.0E-08 |
| ENSG00000248787.1 | RP11-666A20.4 | brown | - | - | 1.1 | 5.7E-03 | - | - | 1.2 | 6.4E-08 |
| ENSG00000269924.1 | RP11-697N18.4 | blue | 1.8 | 5.8E-04 | - | - | - | - | 2.0 | 7.2E-17 |
| ENSG00000249614.1 | RP11-703G6.1 | brown | - | - | 1.5 | 1.2E-05 | 1.2 | 1.9E-15 | 1.8 | 3.6E-10 |
| ENSG00000279670.1 | RP11-70L8.5 | tan | - | - | 2.5 | 3.9E-12 | - | - | 1.6 | 3.0E-13 |
| ENSG00000254528.7 | RP11-728F11.4 | red | -3.6 | 3.9E-05 | - | - | -1.2 | 4.9E-04 | - | - |
| ENSG00000280339.1 | RP11-736K20.4 | yellow | - | - | - | - | -2.2 | 1.5E-23 | -2.3 | 7.2E-17 |
| ENSG00000255471.1 | RP11-736K20.5 | greenyellow | - | - | - | - | -2.0 | 1.1E-13 | -2.5 | 1.7E-16 |
| ENSG00000131484.4 | RP11-798G7.5 | brown | 2.1 | 6.2E-04 | - | - | - | - | 1.0 | 1.7E-05 |
| ENSG00000266918.1 | RP11-798G7.8 | blue | - | - | 1.3 | 1.1E-04 | 1.2 | 6.1E-10 | - | - |
| ENSG00000265490.1 | RP11-806L2.6 | magenta | 2.1 | 4.3E-04 | 1.5 | 3.2E-05 | - | - | - | - |
| ENSG00000276115.1 | RP11-817I4.2 | pink | 1.2 | 8.8E-03 | 2.4 | 9.2E-13 | - | - | - | - |
| ENSG00000279107.1 | RP11-823P9.4 | magenta | - | - | 1.9 | 8.4E-04 | - | - | 1.2 | 4.0E-09 |
| ENSG00000273247.5 | RP11-83A24.2 | brown | 1.1 | 9.1E-03 | 1.1 | 2.4E-06 | - | - | - | - |
| ENSG00000266717.1 | RP11-855A2.3 | brown | 2.2 | 1.6E-04 | - | - | - | - | 1.2 | 2.3E-08 |
| ENSG00000245156.1 | RP11-867G23.3 | brown | 1.6 | 1.0E-03 | 1.5 | 9.3E-06 | - | - | - | - |
| ENSG00000265218.1 | RP11-927P21.1 | brown | 1.2 | 1.8E-03 | 1.4 | 7.2E-05 | - | - | - | - |
| ENSG00000267026.5 | RP11-92C4.3 | turquoise | - | - | 2.1 | 1.1E-04 | 2.2 | 9.9E-06 | - | - |
| ENSG00000257303.1 | RP11-977G19.11 | magenta | 3.1 | 2.8E-07 | 1.3 | 3.0E-03 | - | - | - | - |
| ENSG00000215769.8 | RP13-104F24.2 | blue | 1.9 | 2.7E-07 | 1.4 | 1.1E-06 | - | - | - | - |
| ENSG00000279312.1 | RP3-331H24.7 | grey | -3.5 | 9.8E-16 | 1.1 | 3.0E-04 | -2.2 | 8.9E-13 | - | - |
| ENSG00000279159.1 | RP3-394A18.1 | grey | 1.3 | 1.2E-03 | 1.1 | 1.8E-04 | - | - | - | - |
| ENSG00000260418.1 | RP3-406A7.7 | turquoise | 2.2 | 4.2E-05 | - | - | - | - | 1.3 | 2.2E-08 |
| ENSG00000279453.1 | RP3-425C14.4 | red | -1.7 | 9.9E-05 | - | - | -1.3 | 4.2E-14 | -1.3 | 8.1E-19 |
| ENSG00000230910.2 | RP3-525N10.2 | red | -3.9 | 7.2E-09 | - | - | -2.0 | 2.0E-09 | - | - |
| ENSG00000235501.5 | RP4-639F20.1 | red | -2.5 | 1.0E-04 | - | - | -1.1 | 1.6E-05 | - | - |
| ENSG00000261135.1 | RP4-798A10.7 | brown | 1.9 | 6.9E-05 | 1.2 | 2.6E-04 | - | - | - | - |
| ENSG00000224093.5 | RP5-1033H22.2 | grey | 1.4 | 4.5E-03 | - | - | 1.1 | 5.8E-07 | - | - |
| ENSG00000273373.1 | RP5-1074L1.4 | grey | 1.4 | 1.7E-03 | 1.3 | 1.4E-04 | - | - | - | - |
| ENSG00000270605.1 | RP5-1092A3.4 | magenta | 1.4 | 1.6E-03 | 1.7 | 1.3E-10 | - | - | - | - |
| ENSG00000260793.2 | RP5-882C2.2 | brown | 1.7 | 2.2E-09 | 1.1 | 7.5E-04 | - | - | 1.5 | 3.3E-10 |
| ENSG00000244701.1 | RP5-894A10.2 | brown | 1.0 | 1.9E-04 | 1.5 | 5.2E-09 | - | - | 1.0 | 2.6E-08 |
| ENSG00000274536.4 | RP6-159A1.4 | green | -2.3 | 1.6E-04 | - | - | -1.7 | 2.7E-15 | - | - |
| ENSG00000225855.6 | RUSC1-AS1 | brown | 2.0 | 6.2E-07 | 1.3 | 3.7E-08 | - | - | 1.2 | 4.3E-12 |
| ENSG00000245275.7 | SAP30L-AS1 | blue | - | - | - | - | 1.7 | 7.0E-09 | 1.7 | 1.6E-24 |
| ENSG00000251322.7 | SHANK3 | yellow | -1.5 | 5.0E-04 | - | - | -2.3 | 3.7E-30 | -1.8 | 2.8E-11 |
| ENSG00000279078.1 | SND1-IT1 | brown | 1.7 | 8.6E-03 | 1.2 | 2.3E-03 | - | - | 1.3 | 1.8E-14 |
| ENSG00000242125.3 | SNHG3 | magenta | 1.3 | 7.8E-03 | 1.2 | 1.6E-11 | - | - | 1.1 | 2.9E-12 |
| ENSG00000281398.2 | SNHG4 | salmon | 2.2 | 2.0E-06 | - | - | 1.2 | 5.5E-07 | - | - |

| | | | | | | | | | | |
|---|---|---|---|---|---|---|---|---|---|---|
| ENSG00000227543.4 | SPAG5-AS1 | lightgreen | 1.3 | 6.1E-04 | - | - | - | - | 1.3 | 3.5E-13 |
| ENSG00000249700.8 | SRD5A3-AS1 | tan | 1.3 | 8.4E-03 | - | - | 1.0 | 1.2E-05 | - | - |
| ENSG00000204832.9 | ST8SIA6-AS1 | pink | - | - | 2.1 | 3.1E-06 | - | - | 1.5 | 2.5E-14 |
| ENSG00000227954.6 | TARID | blue | -3.5 | 2.5E-12 | - | - | -2.8 | 2.4E-16 | - | - |
| ENSG00000229950.1 | TFAP2A-AS1 | brown | 1.5 | 4.2E-03 | - | - | - | - | 1.7 | 9.0E-11 |
| ENSG00000223573.6 | TINCR | blue | - | - | -1.2 | 7.5E-06 | -2.6 | 1.4E-24 | - | - |
| ENSG00000175147.11 | TMEM51-AS1 | magenta | 2.2 | 4.6E-07 | - | - | 1.2 | 2.6E-05 | - | - |
| ENSG00000248275.1 | TRIM52-AS1 | turquoise | -1.0 | 8.5E-04 | - | - | - | - | -1.0 | 1.4E-11 |
| ENSG00000249348.1 | UGDH-AS1 | brown | - | - | 1.8 | 1.2E-11 | - | - | 1.3 | 1.5E-07 |
| ENSG00000259248.5 | USP3-AS1 | turquoise | 1.1 | 1.8E-03 | 1.2 | 1.1E-04 | - | - | - | - |
| ENSG00000232354.7 | VIPR1-AS1 | green | 1.4 | 2.5E-03 | 1.4 | 7.5E-06 | -1.4 | 3.8E-04 | - | - |
| ENSG00000261373.1 | VPS9D1-AS1 | blue | 1.7 | 2.7E-04 | 1.3 | 2.7E-05 | - | - | - | - |
| ENSG00000226245.1 | ZNF32-AS1 | turquoise | 2.0 | 8.8E-04 | 1.2 | 6.3E-03 | - | - | - | - |

## Supplementary Table S5: Functional onco-lncRNAs derived from PubMed

| gene id | gene name |
|---|---|
| ENSG00000241684.5 | ADAMTS9-AS2 |
| ENSG00000245750.7 | DRAIC |
| ENSG00000203499.10 | FAM83H-AS1 |
| ENSG00000268388.5 | FENDRR |
| ENSG00000272143.1 | FGF14-AS2 |
| ENSG00000214548.14 | MEG3 |
| ENSG00000238045.9 | PAGR1 |
| ENSG00000267107.6 | PCAT19 |
| ENSG00000228288.6 | PCAT6 |
| ENSG00000249859.7 | PVT1 |
| ENSG00000227954.6 | TARID |
| ENSG00000258498.6 | DIO3OS |
| ENSG00000206195.10 | DUXAP8 |
| ENSG00000249669.7 | MIR143HG |
| ENSG00000247095.2 | MIR210HG |
| ENSG00000186594.12 | MIR22HG |
| ENSG00000223749.7 | MIR503HG |
| ENSG00000260804.3 | PKI55 |
| ENSG00000251322.7 | SHANK3 |
| ENSG00000223573.6 | TINCR |

## Supplementary Table S6: log2FC (fold change) of onco-PCGs in each cancer type

| Gene id | Gene name | BLC | PRC | ADC | EBC |
|---|---|---|---|---|---|
| ENSG00000001617.11 | SEMA3F | 1.7 | - | -1.3 | - |
| ENSG00000002726.19 | AOC1 | - | -4.2 | 4.4 | - |
| ENSG00000003096.13 | KLHL13 | -2.1 | - | - | -2.9 |
| ENSG00000003137.8 | CYP26B1 | - | - | -1.2 | -3.0 |
| ENSG00000003249.13 | DBNDD1 | 1.4 | - | 1.3 | 1.3 |
| ENSG00000004399.12 | PLXND1 | -1.3 | - | - | -1.6 |
| ENSG00000004776.11 | HSPB6 | -8.4 | -1.2 | -2.1 | -4.2 |
| ENSG00000004799.7 | PDK4 | -4.4 | - | -2.5 | -3.3 |
| ENSG00000004838.13 | ZMYND10 | - | 1.5 | -1.1 | 1.9 |
| ENSG00000005001.9 | PRSS22 | - | - | 1.5 | 1.7 |
| ENSG00000005102.12 | MEOX1 | -4.0 | - | - | -2.7 |
| ENSG00000005187.11 | ACSM3 | - | - | 1.5 | -1.4 |
| ENSG00000005243.9 | COPZ2 | -3.0 | - | - | -1.4 |
| ENSG00000005249.12 | PRKAR2B | -3.2 | - | - | -3.5 |
| ENSG00000005801.16 | ZNF195 | 1.1 | 1.1 | - | - |
| ENSG00000005884.17 | ITGA3 | 1.5 | -1.2 | - | - |
| ENSG00000006016.10 | CRLF1 | - | - | 3.2 | -1.2 |
| ENSG00000006118.14 | TMEM132A | 2.5 | - | 1.2 | - |
| ENSG00000006210.6 | CX3CL1 | -1.9 | -1.3 | -1.7 | -2.7 |
| ENSG00000006468.13 | ETV1 | -1.9 | - | - | -1.5 |
| ENSG00000006555.10 | TTC22 | 1.7 | -1.6 | - | - |
| ENSG00000006625.17 | GGCT | - | - | 1.3 | 1.2 |
| ENSG00000007062.11 | PROM1 | - | -1.5 | 1.9 | - |
| ENSG00000007237.18 | GAS7 | -3.4 | - | - | -1.8 |
| ENSG00000007908.15 | SELE | -4.9 | - | -3.3 | - |
| ENSG00000008256.15 | CYTH3 | -1.4 | - | - | -1.7 |
| ENSG00000008300.14 | CELSR3 | 2.8 | - | - | 1.5 |
| ENSG00000008394.12 | MGST1 | - | - | 1.1 | -1.7 |
| ENSG00000008441.16 | NFIX | -2.9 | - | - | -2.2 |
| ENSG00000010319.6 | SEMA3G | -2.7 | - | -3.2 | -3.0 |
| ENSG00000010610.9 | CD4 | -1.4 | - | - | -1.3 |
| ENSG00000010810.17 | FYN | -2.2 | - | -1.2 | -1.8 |
| ENSG00000011465.16 | DCN | -5.7 | - | -1.1 | -2.4 |

| | | | | | |
|---|---|---|---|---|---|
| ENSG00000012223.12 | LTF | - | -2.2 | 2.2 | -3.1 |
| ENSG00000013297.10 | CLDN11 | -2.5 | - | - | -1.2 |
| ENSG00000013810.18 | TACC3 | 2.1 | - | - | 1.5 |
| ENSG00000015479.17 | MATR3 | 1.2 | 1.2 | - | - |
| ENSG00000018236.14 | CNTN1 | -5.6 | -1.0 | - | -1.9 |
| ENSG00000018280.16 | SLC11A1 | -2.0 | - | -1.6 | 1.1 |
| ENSG00000018408.14 | WWTR1 | -2.0 | - | - | -1.4 |
| ENSG00000018625.14 | ATP1A2 | -6.2 | -1.2 | -1.4 | -3.2 |
| ENSG00000019144.16 | PHLDB1 | -1.4 | - | - | -1.5 |
| ENSG00000019485.12 | PRDM11 | -1.2 | - | -1.2 | -1.5 |
| ENSG00000019549.8 | S-I2 | -1.5 | -1.2 | - | -1.3 |
| ENSG00000020181.17 | ADGRA2 | -3.8 | -1.2 | -1.1 | -2.5 |
| ENSG00000020577.13 | SAMD4A | -2.5 | - | - | -2.0 |
| ENSG00000020633.18 | RUNX3 | - | - | -1.3 | -1.5 |
| ENSG00000022267.16 | FHL1 | -5.5 | - | -3.1 | -4.3 |
| ENSG00000024422.11 | EHD2 | -2.2 | - | -1.4 | -2.6 |
| ENSG00000025039.14 | RRAGD | -1.6 | - | - | -2.7 |
| ENSG00000026025.13 | VIM | -3.4 | - | - | -1.8 |
| ENSG00000027075.13 | PRKCH | - | - | -1.2 | -1.6 |
| ENSG00000028137.16 | TNFRSF1B | -2.0 | - | -1.1 | -2.1 |
| ENSG00000029993.14 | HMGB3 | 1.4 | - | 2.9 | 1.2 |
| ENSG00000031081.10 | ARHGAP31 | -2.1 | - | -1.6 | -2.4 |
| ENSG00000033327.12 | GAB2 | -1.3 | - | -1.1 | - |
| ENSG00000034971.14 | MYOC | -7.9 | - | -4.3 | - |
| ENSG00000035403.16 | VCL | -1.5 | - | - | -1.6 |
| ENSG00000035862.12 | TIMP2 | -4.1 | - | - | -1.4 |
| ENSG00000036448.9 | MYOM2 | -3.6 | - | -1.8 | - |
| ENSG00000036672.15 | USP2 | -3.3 | -1.0 | - | - |
| ENSG00000037280.15 | FLT4 | - | - | -2.0 | -1.4 |
| ENSG00000039523.17 | FAM65A | -1.1 | - | -1.3 | - |
| ENSG00000041982.14 | TNC | -2.8 | - | - | -1.0 |
| ENSG00000042062.11 | FAM65C | -1.5 | - | -1.1 | - |
| ENSG00000046653.14 | GPM6B | -2.1 | - | -1.4 | -1.9 |
| ENSG00000046889.18 | PREX2 | -2.5 | - | -1.7 | -2.2 |
| ENSG00000047457.13 | CP | - | - | 4.0 | -2.2 |
| ENSG00000047617.14 | ANO2 | - | - | -2.5 | -1.6 |

| | | | | | |
|---|---|---|---|---|---|
| ENSG00000047648.21 | ARHGAP6 | - | - | -2.4 | -1.7 |
| ENSG00000048540.14 | LMO3 | -2.6 | - | - | -2.0 |
| ENSG00000048740.17 | CELF2 | -3.1 | - | -1.0 | -2.7 |
| ENSG00000049283.17 | EPN3 | - | - | 3.4 | 1.8 |
| ENSG00000049540.16 | ELN | -4.1 | - | -1.0 | - |
| ENSG00000050165.17 | DKK3 | -3.2 | -1.1 | -1.1 | -1.9 |
| ENSG00000050555.17 | LAMC3 | -2.6 | - | -3.4 | - |
| ENSG00000050628.20 | PTGER3 | -3.7 | - | - | -1.4 |
| ENSG00000050767.15 | COL23A1 | - | -1.1 | - | -1.5 |
| ENSG00000051180.16 | RAD51 | 1.3 | - | - | 1.0 |
| ENSG00000053747.15 | LAMA3 | - | - | -2.3 | -1.4 |
| ENSG00000053918.15 | KCNQ1 | - | -1.1 | - | -1.4 |
| ENSG00000055813.5 | CCDC85A | - | - | -3.1 | -3.8 |
| ENSG00000056998.18 | GYG2 | - | - | 2.6 | -4.5 |
| ENSG00000057704.10 | TMCC3 | - | - | -1.2 | -1.9 |
| ENSG00000058668.14 | ATP2B4 | -1.2 | - | - | -2.0 |
| ENSG00000058866.14 | DGKG | -2.9 | - | -1.2 | - |
| ENSG00000059804.15 | SLC2A3 | -3.5 | - | -1.7 | -1.2 |
| ENSG00000060718.18 | COL11A1 | - | - | 4.5 | 4.3 |
| ENSG00000061656.9 | SPAG4 | - | - | 1.7 | 1.3 |
| ENSG00000061918.12 | GUCY1B3 | -1.8 | - | - | -1.5 |
| ENSG00000062038.13 | CDH3 | 2.0 | -1.3 | 2.4 | - |
| ENSG00000062716.10 | VMP1 | - | - | 1.1 | 1.4 |
| ENSG00000063180.8 | CA11 | -1.9 | -1.1 | - | - |
| ENSG00000064042.17 | LIMCH1 | - | - | -1.5 | -1.6 |
| ENSG00000064205.10 | WISP2 | -5.6 | - | -1.9 | -1.3 |
| ENSG00000064300.8 | NGFR | - | -1.5 | - | -2.2 |
| ENSG00000064651.13 | SLC12A2 | - | - | 1.3 | -1.7 |
| ENSG00000064655.18 | EYA2 | - | - | 1.8 | -1.8 |
| ENSG00000064787.12 | BCAS1 | - | - | 1.9 | 1.6 |
| ENSG00000064989.12 | CALCRL | -1.6 | - | -2.1 | -1.4 |
| ENSG00000064999.13 | ANKS1A | - | - | -1.1 | -1.5 |
| ENSG00000065054.13 | SLC9A3R2 | -1.8 | - | -1.3 | - |
| ENSG00000065154.11 | OAT | -1.8 | -1.2 | - | - |
| ENSG00000065320.8 | NTN1 | -3.5 | -1.8 | - | - |
| ENSG00000065361.14 | ERBB3 | 2.0 | - | 1.1 | - |

| | | | | | |
|---|---|---|---|---|---|
| ENSG00000065534.18 | MYLK | -5.5 | - | -1.1 | -1.9 |
| ENSG00000065618.16 | COL17A1 | - | -2.6 | 3.1 | -2.4 |
| ENSG00000065833.8 | ME1 | -2.2 | - | - | -3.1 |
| ENSG00000066056.13 | TIE1 | -2.2 | - | -2.3 | -1.6 |
| ENSG00000066336.11 | SPI1 | -2.1 | - | -1.2 | - |
| ENSG00000066468.20 | FGFR2 | - | -1.4 | -1.3 | -1.2 |
| ENSG00000066629.16 | EML1 | -2.9 | - | -1.7 | - |
| ENSG00000066735.14 | KIF26A | - | - | -2.2 | -2.6 |
| ENSG00000067082.14 | KLF6 | -1.2 | - | -1.8 | -1.0 |
| ENSG00000067113.16 | PPAP2A | -1.4 | - | - | -2.0 |
| ENSG00000067840.12 | PDZD4 | -3.4 | - | -1.8 | - |
| ENSG00000068001.13 | HYAL2 | -1.0 | - | -2.2 | - |
| ENSG00000068024.16 | HDAC4 | -1.9 | - | - | -1.2 |
| ENSG00000068028.17 | RASSF1 | -1.3 | - | -1.1 | - |
| ENSG00000068078.17 | FGFR3 | 2.1 | - | - | 1.4 |
| ENSG00000068383.18 | INPP5A | -1.5 | - | -1.0 | -1.1 |
| ENSG00000068615.16 | REEP1 | -5.6 | - | -1.6 | 1.1 |
| ENSG00000068831.18 | RASGRP2 | -3.3 | - | -1.4 | -1.2 |
| ENSG00000069011.15 | PITX1 | - | -2.1 | 4.3 | 4.2 |
| ENSG00000069535.13 | MAOB | -4.6 | - | -1.1 | -1.3 |
| ENSG00000069702.10 | TGFBR3 | -1.6 | - | -2.5 | -2.9 |
| ENSG00000070193.4 | FGF10 | -6.9 | - | -1.5 | -3.2 |
| ENSG00000070404.9 | FSTL3 | -2.5 | - | -2.2 | -1.1 |
| ENSG00000070501.11 | POLB | 1.1 | - | - | 1.0 |
| ENSG00000070614.14 | NDST1 | - | - | -1.5 | -1.8 |
| ENSG00000070731.9 | ST6GAL-C2 | - | -1.5 | -1.5 | - |
| ENSG00000070756.13 | PABPC1 | 1.2 | - | 1.1 | - |
| ENSG00000070778.12 | PTPN21 | - | - | -2.0 | -1.7 |
| ENSG00000071205.11 | ARHGAP10 | -1.6 | - | - | -1.7 |
| ENSG00000071242.11 | RPS6KA2 | -1.7 | - | -1.1 | -1.5 |
| ENSG00000071246.10 | VASH1 | - | - | -1.0 | -1.1 |
| ENSG00000071282.11 | LMCD1 | -3.7 | - | -2.0 | -1.1 |
| ENSG00000071539.13 | TRIP13 | 2.6 | - | 1.1 | 1.1 |
| ENSG00000071967.11 | CYBRD1 | -3.8 | - | -1.4 | -1.7 |
| ENSG00000072080.10 | SPP2 | 1.7 | 1.6 | - | 1.0 |
| ENSG00000072163.18 | LIMS2 | -4.9 | - | -3.0 | -1.7 |

| | | | | | |
|---|---|---|---|---|---|
| ENSG00000072840.12 | EVC | -2.0 | - | - | -1.3 |
| ENSG00000072952.18 | MRVI1 | -3.7 | - | - | -1.1 |
| ENSG00000073282.12 | TP63 | - | -2.4 | 2.0 | -1.9 |
| ENSG00000073350.13 | LLGL2 | 1.4 | - | - | 1.5 |
| ENSG00000073605.18 | GSDMB | 2.1 | 1.3 | 1.4 | - |
| ENSG00000073712.13 | FERMT2 | -3.3 | - | -1.3 | -1.5 |
| ENSG00000073756.11 | PTGS2 | -1.8 | -1.3 | - | - |
| ENSG00000073849.14 | ST6GAL1 | -1.9 | - | - | -1.4 |
| ENSG00000074416.13 | MGLL | -2.0 | - | - | -2.5 |
| ENSG00000074660.15 | SCARF1 | -1.1 | - | - | -1.1 |
| ENSG00000075213.10 | SEMA3A | -2.3 | - | 1.0 | - |
| ENSG00000075218.18 | GTSE1 | 2.8 | - | - | 1.9 |
| ENSG00000075239.13 | ACAT1 | -2.1 | - | - | -1.0 |
| ENSG00000075426.11 | FOSL2 | - | - | -1.5 | -1.3 |
| ENSG00000075461.5 | CACNG4 | - | -1.6 | - | 1.9 |
| ENSG00000075702.16 | WDR62 | 2.7 | - | - | 1.2 |
| ENSG00000076067.11 | RBMS2 | -1.0 | - | -1.2 | - |
| ENSG00000076344.15 | RGS11 | -3.5 | 1.8 | -1.1 | 1.3 |
| ENSG00000076382.16 | SPAG5 | 2.1 | 1.4 | 1.0 | 2.0 |
| ENSG00000076554.15 | TPD52 | - | - | 1.2 | 1.1 |
| ENSG00000076555.15 | ACACB | -2.3 | - | -1.2 | -3.1 |
| ENSG00000076706.14 | MCAM | -2.8 | - | -1.5 | -2.8 |
| ENSG00000076944.14 | STXBP2 | 1.4 | - | - | 1.4 |
| ENSG00000077152.9 | UBE2T | - | - | 1.8 | 2.7 |
| ENSG00000077157.20 | PPP1R12B | -3.1 | - | - | -1.4 |
| ENSG00000077274.8 | CAPN6 | - | -1.7 | - | -3.8 |
| ENSG00000077420.15 | APBB1IP | -2.3 | - | - | -2.3 |
| ENSG00000077782.19 | FGFR1 | -4.7 | - | - | -1.3 |
| ENSG00000077942.17 | FBLN1 | -2.7 | - | -1.6 | -1.1 |
| ENSG00000077943.7 | ITGA8 | -3.6 | - | -1.9 | - |
| ENSG00000077984.5 | CST7 | -3.2 | - | -1.4 | - |
| ENSG00000078018.19 | MAP2 | - | 1.2 | -1.2 | -1.3 |
| ENSG00000078081.7 | LAMP3 | - | -1.7 | -2.1 | - |
| ENSG00000078098.13 | FAP | -2.8 | - | 2.0 | 1.3 |
| ENSG00000078401.6 | EDN1 | -1.1 | - | -2.4 | - |
| ENSG00000078596.10 | ITM2A | -4.9 | - | -1.7 | -2.1 |

| | | | | | |
|---|---|---|---|---|---|
| ENSG00000079308.16 | TNS1 | -4.6 | - | -1.7 | -3.9 |
| ENSG00000079385.21 | CEACAM1 | - | -1.1 | 1.9 | - |
| ENSG00000079435.9 | LIPE | - | -1.2 | - | -4.2 |
| ENSG00000079462.7 | PAFAH1B3 | 2.5 | - | 1.3 | 2.3 |
| ENSG00000079819.16 | EPB41L2 | -2.5 | - | -1.4 | -2.5 |
| ENSG00000080573.6 | COL5A3 | - | - | -1.2 | -1.8 |
| ENSG00000081041.8 | CXCL2 | - | - | -3.0 | -2.8 |
| ENSG00000081189.13 | MEF2C | -2.5 | - | - | -1.8 |
| ENSG00000081277.11 | PKP1 | - | -1.1 | - | -3.0 |
| ENSG00000081818.3 | PCDHB4 | -3.4 | - | -1.0 | -1.3 |
| ENSG00000081853.14 | PCDHGA2 | - | - | -1.3 | -1.8 |
| ENSG00000082196.20 | C1QTNF3 | - | 1.9 | 1.9 | 1.5 |
| ENSG00000082397.15 | EPB41L3 | -3.1 | - | -1.0 | -1.9 |
| ENSG00000083857.13 | FAT1 | - | - | 1.0 | -2.0 |
| ENSG00000084207.15 | GSTP1 | - | -1.7 | - | -1.2 |
| ENSG00000085063.14 | CD59 | -1.1 | - | - | -1.1 |
| ENSG00000085265.10 | FCN1 | -4.0 | - | -1.7 | - |
| ENSG00000085514.15 | PILRA | -3.2 | - | -1.2 | - |
| ENSG00000085552.16 | IGSF9 | 2.4 | - | 1.9 | 1.5 |
| ENSG00000085563.14 | ABCB1 | -2.1 | - | -2.1 | -1.8 |
| ENSG00000086289.11 | EPDR1 | -2.5 | - | - | -2.1 |
| ENSG00000087074.7 | PPP1R15A | -1.2 | - | -2.1 | - |
| ENSG00000087085.13 | ACHE | - | - | 2.0 | -1.1 |
| ENSG00000087116.13 | ADAMTS2 | -2.7 | - | - | -1.6 |
| ENSG00000087510.6 | TFAP2C | - | - | 1.5 | -1.5 |
| ENSG00000087586.17 | AURKA | 2.2 | - | 1.3 | 1.6 |
| ENSG00000087903.12 | RFX2 | - | - | -1.5 | -1.2 |
| ENSG00000088002.11 | SULT2B1 | - | -1.9 | -1.2 | - |
| ENSG00000088320.3 | REM1 | -3.2 | - | -2.0 | -1.8 |
| ENSG00000088325.15 | TPX2 | 3.0 | - | 1.8 | 2.3 |
| ENSG00000088836.12 | SLC4A11 | 2.1 | -1.1 | 2.6 | - |
| ENSG00000088882.7 | CPXM1 | -3.2 | -1.3 | - | -1.4 |
| ENSG00000088992.17 | TESC | - | - | 1.6 | -1.9 |
| ENSG00000089127.12 | OAS1 | 2.1 | - | 1.0 | - |
| ENSG00000089472.16 | HEPH | -3.7 | - | - | -1.8 |
| ENSG00000089486.16 | CDIP1 | - | -1.2 | - | -1.3 |

| | | | | | |
|---|---|---|---|---|---|
| ENSG00000089685.14 | BIRC5 | 1.7 | - | 1.2 | - |
| ENSG00000090006.17 | LTBP4 | -3.6 | - | -2.7 | -1.5 |
| ENSG00000090104.11 | RGS1 | -2.6 | - | - | 1.1 |
| ENSG00000090447.11 | TFAP4 | 1.4 | - | 1.1 | - |
| ENSG00000090530.9 | P3H2 | - | - | -1.6 | -2.2 |
| ENSG00000090776.5 | EFNB1 | - | -1.5 | -1.2 | -2.2 |
| ENSG00000090975.12 | PITPNM2 | -1.8 | - | -1.3 | -1.1 |
| ENSG00000091073.19 | DTX2 | 1.0 | - | 1.0 | - |
| ENSG00000091436.16 | pk | -3.0 | - | - | -1.1 |
| ENSG00000091490.10 | SEL1L3 | - | - | 1.1 | -1.2 |
| ENSG00000091513.14 | TF | - | -1.4 | - | -2.4 |
| ENSG00000091527.15 | CDV3 | -1.1 | - | - | -1.5 |
| ENSG00000091972.18 | CD200 | -2.1 | - | -1.2 | -1.5 |
| ENSG00000091986.15 | CCDC80 | -4.0 | - | - | -1.6 |
| ENSG00000092051.16 | JPH4 | - | -1.1 | -1.3 | - |
| ENSG00000092096.14 | SLC22A17 | -1.7 | -1.6 | - | - |
| ENSG00000092295.11 | TGM1 | - | -1.7 | -2.6 | - |
| ENSG00000092421.16 | SEMA6A | 1.8 | - | -2.9 | -1.0 |
| ENSG00000092621.11 | PHGDH | - | -1.2 | - | -1.6 |
| ENSG00000092758.15 | COL9A3 | -1.5 | - | 1.5 | - |
| ENSG00000092853.13 | CLSPN | 2.8 | - | - | 1.4 |
| ENSG00000092964.16 | DPYSL2 | -2.8 | - | -1.2 | -2.5 |
| ENSG00000092969.11 | TGFB2 | -2.1 | - | -1.1 | - |
| ENSG00000093009.9 | CDC45 | 2.4 | - | - | 2.0 |
| ENSG00000094755.16 | GABRP | - | -1.5 | - | -2.2 |
| ENSG00000094804.9 | CDC6 | 2.1 | - | 1.9 | - |
| ENSG00000094963.13 | FMO2 | - | - | -2.4 | -3.1 |
| ENSG00000095370.19 | SH2D3C | -1.8 | - | -2.5 | -1.3 |
| ENSG00000095383.19 | TBC1D2 | - | -1.3 | -1.2 | - |
| ENSG00000095585.16 | BLNK | - | 1.2 | 1.4 | 1.0 |
| ENSG00000095637.20 | SORBS1 | -4.5 | - | - | -3.6 |
| ENSG00000095713.13 | CRTAC1 | - | - | -1.5 | -2.1 |
| ENSG00000095794.19 | CREM | -3.0 | - | -1.4 | - |
| ENSG00000096060.14 | FKBP5 | -2.4 | - | - | -1.2 |
| ENSG00000096063.14 | SRPK1 | 1.3 | - | 1.2 | - |
| ENSG00000097007.17 | ABL1 | -1.9 | - | - | -1.3 |

| | | | | | |
|---|---|---|---|---|---|
| ENSG00000099204.18 | ABLIM1 | - | - | -1.2 | -2.1 |
| ENSG00000099250.17 | NRP1 | -1.6 | - | - | -2.0 |
| ENSG00000099260.10 | PALMD | - | - | -1.3 | -2.6 |
| ENSG00000099849.14 | RASSF7 | 1.4 | - | - | 1.8 |
| ENSG00000099860.8 | GADD45B | -3.5 | - | -2.2 | - |
| ENSG00000099864.17 | PALM | -2.3 | - | -1.2 | -2.2 |
| ENSG00000099953.9 | MMP11 | 1.3 | - | 4.4 | 3.4 |
| ENSG00000099994.10 | SUSD2 | -1.2 | - | -1.3 | - |
| ENSG00000099998.17 | GGT5 | -2.1 | - | 1.2 | - |
| ENSG00000100033.16 | PRODH | - | - | 1.1 | 1.9 |
| ENSG00000100034.13 | PPM1F | - | - | -1.7 | -1.0 |
| ENSG00000100060.17 | MFNG | -2.5 | - | -1.4 | -1.6 |
| ENSG00000100079.6 | LGALS2 | -2.5 | - | -1.2 | - |
| ENSG00000100154.14 | TTC28 | -1.5 | - | -1.2 | -1.8 |
| ENSG00000100162.14 | CENPM | - | 1.3 | - | 2.1 |
| ENSG00000100234.11 | TIMP3 | -3.0 | - | -1.4 | -2.5 |
| ENSG00000100243.20 | CYB5R3 | - | - | -1.0 | -1.4 |
| ENSG00000100290.2 | BIK | - | - | 1.2 | 1.3 |
| ENSG00000100307.12 | CBX7 | -1.7 | - | -1.2 | -1.6 |
| ENSG00000100321.14 | SYNGR1 | -1.3 | -1.1 | - | - |
| ENSG00000100422.13 | CERK | -2.1 | - | - | -1.4 |
| ENSG00000100429.17 | HDAC10 | 1.3 | 1.2 | - | 1.1 |
| ENSG00000100479.12 | POLE2 | 1.7 | - | 1.3 | 1.3 |
| ENSG00000100526.19 | CDKN3 | - | - | 1.8 | 1.9 |
| ENSG00000100599.15 | RIN3 | -1.3 | - | - | -1.5 |
| ENSG00000100605.16 | ITPK1 | -1.1 | - | - | -1.0 |
| ENSG00000100625.8 | SIX4 | 1.2 | - | 2.1 | - |
| ENSG00000100626.16 | GALNT16 | -2.7 | - | -1.4 | - |
| ENSG00000100941.8 | PNN | - | 1.0 | - | 1.0 |
| ENSG00000100994.11 | PYGB | -2.2 | - | - | -1.4 |
| ENSG00000101003.9 | GINS1 | 2.3 | - | 1.9 | 1.4 |
| ENSG00000101057.15 | MYBL2 | 2.5 | - | 1.8 | 2.7 |
| ENSG00000101096.19 | NFATC2 | -2.4 | - | - | -2.3 |
| ENSG00000101104.12 | PABPC1L | 1.6 | 1.3 | 1.3 | 1.5 |
| ENSG00000101115.12 | SALL4 | 2.1 | - | 3.3 | - |
| ENSG00000101187.15 | SLCO4A1 | - | - | -2.0 | -1.8 |

| | | | | | |
|---|---|---|---|---|---|
| ENSG00000101194.17 | SLC17A9 | - | - | 1.4 | 2.4 |
| ENSG00000101210.10 | EEF1A2 | - | - | 5.1 | 3.2 |
| ENSG00000101213.6 | PTK6 | 2.2 | - | 1.8 | 1.2 |
| ENSG00000101265.15 | RASSF2 | -1.6 | - | -1.3 | -1.4 |
| ENSG00000101311.15 | FERMT1 | 1.3 | - | 2.5 | - |
| ENSG00000101331.15 | CCM2L | -2.0 | - | -2.8 | -1.9 |
| ENSG00000101333.16 | PLCB4 | -2.5 | 1.1 | - | - |
| ENSG00000101335.9 | MYL9 | -5.8 | -1.0 | -1.6 | -1.4 |
| ENSG00000101336.12 | HCK | -1.9 | - | -1.1 | - |
| ENSG00000101347.8 | SAMHD1 | -2.1 | - | - | -1.3 |
| ENSG00000101400.5 | SNTA1 | -1.9 | - | - | -1.4 |
| ENSG00000101412.12 | E2F1 | 1.7 | - | - | 1.7 |
| ENSG00000101443.17 | WFDC2 | - | -1.8 | 2.5 | - |
| ENSG00000101445.9 | PPP1R16B | - | - | -1.4 | -3.2 |
| ENSG00000101470.9 | TNNC2 | -2.4 | - | 1.1 | - |
| ENSG00000101605.12 | MYOM1 | -3.7 | - | - | -2.9 |
| ENSG00000101665.8 | SMAD7 | -1.3 | - | -1.2 | -1.0 |
| ENSG00000101938.14 | CHRDL1 | -7.8 | -1.2 | -1.6 | -4.6 |
| ENSG00000101955.14 | SRPX | -6.0 | -1.3 | -1.9 | -3.2 |
| ENSG00000102024.17 | PLS3 | -1.1 | - | - | -1.6 |
| ENSG00000102181.19 | CD99L2 | -2.0 | - | - | -1.6 |
| ENSG00000102265.11 | TIMP1 | -1.6 | - | - | 1.2 |
| ENSG00000102359.5 | SRPX2 | - | 1.1 | 2.1 | - |
| ENSG00000102543.14 | CDADC1 | -1.4 | - | - | -1.2 |
| ENSG00000102547.18 | CAB39L | - | - | -1.2 | -1.7 |
| ENSG00000102554.13 | KLF5 | - | -1.3 | - | -1.9 |
| ENSG00000102755.10 | FLT1 | - | - | -1.1 | -1.7 |
| ENSG00000102760.12 | RGCC | -2.6 | - | -2.9 | -2.4 |
| ENSG00000102802.9 | MEDAG | -4.1 | - | -1.6 | -3.0 |
| ENSG00000102804.14 | TSC22D1 | -1.7 | - | -1.0 | -1.0 |
| ENSG00000102878.15 | HSF4 | - | 1.7 | - | 1.3 |
| ENSG00000102931.7 | ARL2BP | -2.0 | - | - | -1.3 |
| ENSG00000102935.11 | ZNF423 | -1.8 | - | - | -1.9 |
| ENSG00000103034.14 | NDRG4 | - | -1.2 | -2.7 | -1.1 |
| ENSG00000103175.10 | WFDC1 | -3.2 | - | -2.4 | - |
| ENSG00000103196.11 | CRISPLD2 | -4.5 | -1.3 | -1.7 | - |

| | | | | | |
|---|---|---|---|---|---|
| ENSG00000103241.6 | FOXF1 | -3.9 | - | -3.6 | - |
| ENSG00000103316.10 | CRYM | -2.3 | - | 3.3 | - |
| ENSG00000103335.19 | PIEZO1 | - | - | -1.2 | -1.1 |
| ENSG00000103460.16 | TOX3 | 2.4 | - | 3.7 | - |
| ENSG00000103490.13 | PYCARD | - | -1.2 | - | 1.4 |
| ENSG00000103534.16 | TMC5 | - | - | 2.2 | 1.2 |
| ENSG00000103550.13 | KNOP1 | 1.4 | - | - | 1.1 |
| ENSG00000103647.12 | CORO2B | -3.1 | - | -2.0 | -2.4 |
| ENSG00000103710.10 | RASL12 | -4.4 | - | -1.8 | -1.9 |
| ENSG00000103994.16 | ZNF106 | - | - | -1.1 | -1.5 |
| ENSG00000104140.6 | RHOV | - | -1.5 | 2.7 | - |
| ENSG00000104164.10 | BLOC1S6 | - | 1.2 | - | 1.1 |
| ENSG00000104219.12 | ZDHHC2 | -1.5 | - | - | -2.0 |
| ENSG00000104324.15 | CPQ | -2.2 | - | - | -1.3 |
| ENSG00000104332.11 | SFRP1 | -5.5 | - | - | -3.0 |
| ENSG00000104368.17 | PLAT | - | -1.0 | 1.6 | - |
| ENSG00000104375.15 | STK3 | - | - | 1.0 | 1.0 |
| ENSG00000104413.15 | ESRP1 | 1.5 | - | 1.3 | - |
| ENSG00000104419.14 | NDRG1 | - | -1.1 | - | -2.2 |
| ENSG00000104450.12 | SPAG1 | - | - | 1.6 | 1.1 |
| ENSG00000104490.17 | NCALD | -2.6 | - | - | -2.2 |
| ENSG00000104635.13 | SLC39A14 | -1.8 | - | - | -1.6 |
| ENSG00000104695.12 | PPP2CB | -1.3 | - | - | -1.2 |
| ENSG00000104728.15 | ARHGEF10 | -1.6 | - | -1.6 | - |
| ENSG00000104738.16 | MCM4 | 1.8 | - | 1.0 | - |
| ENSG00000104756.15 | KCTD9 | -1.4 | - | - | -1.4 |
| ENSG00000104889.4 | R-SEH2A | 1.3 | - | - | 1.1 |
| ENSG00000104967.6 | NOVA2 | - | - | -2.8 | -1.1 |
| ENSG00000105011.8 | ASF1B | 2.2 | - | 1.0 | 2.3 |
| ENSG00000105048.16 | TNNT1 | - | - | 1.7 | 3.2 |
| ENSG00000105131.7 | EPHX3 | 2.1 | - | 2.1 | - |
| ENSG00000105137.12 | SYDE1 | -2.3 | - | -1.3 | -1.7 |
| ENSG00000105173.13 | CCNE1 | 1.3 | - | 1.8 | - |
| ENSG00000105227.14 | PRX | - | - | -4.1 | -1.8 |
| ENSG00000105270.14 | CLIP3 | -3.9 | - | - | -1.1 |
| ENSG00000105374.9 | NKG7 | -2.1 | - | -2.0 | - |

| | | | | | |
|---|---|---|---|---|---|
| ENSG00000105427.9 | CNFN | - | - | 1.9 | 1.4 |
| ENSG00000105538.8 | RASIP1 | -2.4 | - | -3.1 | -2.2 |
| ENSG00000105559.11 | PLEKHA4 | -2.0 | - | - | -1.1 |
| ENSG00000105609.16 | LILRB5 | -2.8 | - | - | -1.1 |
| ENSG00000105655.18 | ISY-1 | - | -1.2 | - | 1.2 |
| ENSG00000105664.10 | COMP | - | - | 4.2 | 3.5 |
| ENSG00000105707.13 | HPN | - | 1.8 | 1.2 | 1.7 |
| ENSG00000105835.11 | -MPT | -1.8 | - | -1.4 | - |
| ENSG00000105894.11 | PTN | -3.9 | - | -1.9 | - |
| ENSG00000105926.15 | MPP6 | - | 1.1 | 1.1 | -1.4 |
| ENSG00000105971.14 | CAV2 | -1.9 | - | -2.7 | -2.9 |
| ENSG00000105974.11 | CAV1 | -3.9 | - | -3.6 | -3.4 |
| ENSG00000105976.14 | MET | - | -1.2 | 1.2 | -2.8 |
| ENSG00000106004.4 | HOXA5 | 2.1 | - | -1.9 | -2.1 |
| ENSG00000106006.6 | HOXA6 | - | - | -1.1 | -1.9 |
| ENSG00000106034.17 | CPED1 | -4.8 | - | -1.4 | -3.5 |
| ENSG00000106066.13 | CPVL | -4.1 | - | - | -1.6 |
| ENSG00000106078.17 | COBL | - | - | -1.0 | -1.6 |
| ENSG00000106089.11 | STX1A | 1.8 | - | 1.7 | 1.6 |
| ENSG00000106123.11 | EPHB6 | 1.6 | -1.2 | -1.4 | -1.9 |
| ENSG00000106258.13 | CYP3A5 | 1.4 | -1.7 | - | 1.7 |
| ENSG00000106327.12 | TFR2 | 3.0 | - | - | 1.7 |
| ENSG00000106462.10 | EZH2 | 2.1 | 1.2 | 1.1 | 1.6 |
| ENSG00000106538.9 | RARRES2 | -3.4 | - | - | -2.0 |
| ENSG00000106541.11 | AGR2 | - | - | 2.9 | 1.6 |
| ENSG00000106665.15 | CLIP2 | -1.1 | - | - | -1.4 |
| ENSG00000106772.17 | PRUNE2 | -7.1 | - | - | -2.1 |
| ENSG00000106809.10 | OGN | -6.5 | - | -2.0 | -2.1 |
| ENSG00000106819.11 | ASPN | -3.1 | 1.4 | - | 1.5 |
| ENSG00000106823.12 | ECM2 | -2.6 | - | -1.0 | -2.3 |
| ENSG00000106852.15 | LHX6 | - | - | -1.3 | -2.1 |
| ENSG00000106948.16 | AK- | -1.3 | - | - | -1.5 |
| ENSG00000106991.13 | ENG | - | - | -1.6 | -1.5 |
| ENSG00000107104.18 | KANK1 | -1.1 | - | - | -1.9 |
| ENSG00000107159.12 | CA9 | 3.0 | - | 3.5 | - |
| ENSG00000107186.16 | MPDZ | -1.5 | - | - | -1.6 |

| | | | | | |
|---|---|---|---|---|---|
| ENSG00000107263.18 | RAPGEF1 | -1.1 | - | - | -1.6 |
| ENSG00000107317.11 | PTGDS | -5.5 | -1.4 | -1.5 | -2.0 |
| ENSG00000107372.12 | ZFAND5 | -1.2 | - | - | -1.2 |
| ENSG00000107438.8 | PDLIM1 | - | -1.1 | -1.1 | -1.1 |
| ENSG00000107485.15 | GATA3 | 2.0 | -1.9 | - | 1.6 |
| ENSG00000107562.16 | CXCL12 | -5.1 | - | - | -1.9 |
| ENSG00000107719.8 | PALD1 | -1.8 | - | -1.9 | -1.1 |
| ENSG00000107736.19 | CDH23 | - | - | -1.3 | -2.2 |
| ENSG00000107738.19 | C10orf54 | -1.7 | - | -1.9 | -2.7 |
| ENSG00000107742.12 | SPOCK2 | - | - | -2.9 | -1.3 |
| ENSG00000107796.12 | ACTA2 | -5.2 | - | - | -1.3 |
| ENSG00000107821.14 | KAZALD1 | -1.8 | - | - | 1.2 |
| ENSG00000107890.16 | ANKRD26 | - | 1.3 | - | 1.4 |
| ENSG00000108176.14 | D-JC12 | - | - | 3.1 | 1.3 |
| ENSG00000108244.16 | KRT23 | - | -1.8 | - | -1.4 |
| ENSG00000108342.12 | CSF3 | -5.3 | - | -4.9 | - |
| ENSG00000108352.11 | RAPGEFL1 | 1.9 | -1.2 | - | - |
| ENSG00000108370.15 | RGS9 | -4.5 | - | -2.7 | - |
| ENSG00000108381.10 | ASPA | -2.3 | - | -2.0 | - |
| ENSG00000108387.14 | SEPT4 | -1.7 | - | -1.3 | - |
| ENSG00000108511.9 | HOXB6 | 2.6 | - | 1.3 | - |
| ENSG00000108515.17 | ENO3 | - | 1.4 | - | 1.5 |
| ENSG00000108551.4 | RASD1 | -2.5 | - | -1.5 | -1.6 |
| ENSG00000108602.17 | ALDH3A1 | - | -2.3 | - | -2.0 |
| ENSG00000108622.10 | ICAM2 | - | - | -1.4 | -1.4 |
| ENSG00000108691.9 | CCL2 | -5.1 | - | -2.3 | -1.9 |
| ENSG00000108821.13 | COL1A1 | -2.2 | - | 2.0 | - |
| ENSG00000108823.15 | SGCA | -5.5 | -1.0 | -2.1 | - |
| ENSG00000108840.15 | HDAC5 | -1.0 | - | - | -1.1 |
| ENSG00000108846.15 | ABCC3 | 2.1 | - | 2.4 | - |
| ENSG00000108848.15 | LUC7L3 | - | 1.0 | - | 1.3 |
| ENSG00000108924.13 | HLF | -4.8 | - | -1.4 | -2.8 |
| ENSG00000108932.11 | SLC16A6 | - | - | -1.3 | 1.5 |
| ENSG00000108947.4 | EFNB3 | - | -1.8 | - | -1.7 |
| ENSG00000108984.13 | MAP2K6 | - | 1.1 | - | -1.6 |
| ENSG00000109079.9 | TNFAIP1 | - | - | -1.0 | -1.0 |

| | | | | | |
|---|---|---|---|---|---|
| ENSG00000109099.13 | PMP22 | -5.0 | - | -1.2 | -1.0 |
| ENSG00000109107.13 | ALDOC | - | -1.4 | - | -2.4 |
| ENSG00000109321.10 | AREG | - | - | -2.0 | 1.3 |
| ENSG00000109472.13 | CPE | -3.4 | - | - | -1.6 |
| ENSG00000109501.13 | WFS1 | -1.0 | - | -1.6 | - |
| ENSG00000109610.5 | SOD3 | -5.5 | -1.3 | -1.2 | -3.1 |
| ENSG00000109654.14 | TRIM2 | - | - | 1.8 | -1.7 |
| ENSG00000109667.11 | SLC2A9 | - | -1.1 | - | -1.6 |
| ENSG00000109686.16 | SH3D19 | -1.2 | - | - | -2.5 |
| ENSG00000109771.15 | LRP2BP | - | 1.3 | -1.1 | - |
| ENSG00000109775.10 | UFSP2 | -1.4 | - | - | -1.0 |
| ENSG00000109846.7 | CRYAB | -5.2 | - | -2.1 | -3.2 |
| ENSG00000109881.16 | CCDC34 | 1.4 | - | 1.0 | - |
| ENSG00000109906.13 | ZBTB16 | -4.6 | - | -2.4 | -2.2 |
| ENSG00000110042.7 | DTX4 | 1.4 | - | - | -2.0 |
| ENSG00000110047.17 | EHD1 | -1.2 | - | -1.1 | - |
| ENSG00000110076.18 | NRXN2 | -4.6 | - | - | -1.2 |
| ENSG00000110079.16 | MS4A4A | -3.1 | - | - | -1.3 |
| ENSG00000110108.9 | TMEM109 | -1.3 | - | - | -1.0 |
| ENSG00000110328.5 | GALNT18 | -1.6 | - | -1.6 | -1.1 |
| ENSG00000110400.10 | PVRL1 | - | -1.3 | 1.3 | -1.5 |
| ENSG00000110422.11 | HIPK3 | -1.3 | - | - | -1.9 |
| ENSG00000110446.9 | SLC15A3 | -1.6 | - | - | -1.1 |
| ENSG00000110492.15 | MDK | 2.0 | - | 1.9 | - |
| ENSG00000110628.13 | SLC22A18 | 1.3 | - | 1.7 | 1.4 |
| ENSG00000110651.11 | CD81 | -1.6 | - | - | -1.2 |
| ENSG00000110799.13 | VWF | -1.8 | - | -2.3 | -3.6 |
| ENSG00000110811.19 | P3H3 | -2.7 | -1.3 | - | - |
| ENSG00000110848.8 | CD69 | -4.5 | - | -1.5 | - |
| ENSG00000110876.9 | SELPLG | -2.0 | - | -1.3 | - |
| ENSG00000110880.10 | CORO1C | -1.8 | - | - | -1.4 |
| ENSG00000110900.14 | TSPAN11 | -2.5 | - | - | -2.2 |
| ENSG00000111052.7 | LIN7A | - | - | -2.4 | -1.5 |
| ENSG00000111058.7 | ACSS3 | -2.2 | - | -1.4 | -2.2 |
| ENSG00000111077.17 | TNS2 | -2.8 | - | -1.6 | -1.3 |
| ENSG00000111145.7 | ELK3 | -1.2 | - | - | -1.5 |

| | | | | | |
|---|---|---|---|---|---|
| ENSG00000111199.10 | TRPV4 | - | -1.3 | 1.5 | -1.3 |
| ENSG00000111206.12 | FOXM1 | 2.4 | 1.2 | 1.5 | 1.6 |
| ENSG00000111215.11 | PRR4 | - | 1.5 | - | 1.2 |
| ENSG00000111247.14 | RAD51AP1 | 1.7 | - | 1.3 | 2.1 |
| ENSG00000111252.10 | SH2B3 | -1.2 | - | -1.0 | -1.2 |
| ENSG00000111269.2 | CREBL2 | -1.3 | - | - | -1.8 |
| ENSG00000111275.12 | ALDH2 | -1.8 | - | - | -1.7 |
| ENSG00000111404.6 | RERGL | -6.0 | - | - | -1.2 |
| ENSG00000111450.13 | STX2 | -1.9 | - | -1.0 | - |
| ENSG00000111452.12 | ADGRD1 | -7.0 | - | -1.9 | -2.3 |
| ENSG00000111665.11 | CDCA3 | 1.4 | 1.2 | 1.2 | 1.9 |
| ENSG00000111716.12 | LDHB | - | -1.1 | - | -2.2 |
| ENSG00000111727.11 | HCFC2 | -1.2 | - | - | -1.4 |
| ENSG00000111816.7 | FRK | - | - | 1.1 | 1.4 |
| ENSG00000111817.16 | DSE | -1.7 | - | - | -2.2 |
| ENSG00000111859.16 | NEDD9 | -1.6 | - | -1.8 | -1.4 |
| ENSG00000111897.6 | SERINC1 | -1.4 | - | - | -1.6 |
| ENSG00000111911.6 | HINT3 | -1.3 | - | - | -1.0 |
| ENSG00000111961.16 | SASH1 | - | - | -1.9 | -2.4 |
| ENSG00000112149.9 | CD83 | -2.0 | - | -1.4 | -1.1 |
| ENSG00000112182.14 | BACH2 | - | - | -1.0 | -1.5 |
| ENSG00000112210.11 | RAB23 | -3.2 | - | - | -1.3 |
| ENSG00000112214.10 | FHL5 | - | - | -2.3 | -2.5 |
| ENSG00000112312.9 | GMNN | - | -1.3 | 1.2 | - |
| ENSG00000112319.17 | EYA4 | -4.3 | - | -1.8 | - |
| ENSG00000112320.11 | SOBP | -3.0 | - | - | -2.2 |
| ENSG00000112419.14 | PHACTR2 | - | - | -1.2 | -1.5 |
| ENSG00000112425.13 | EPM2A | -2.4 | - | - | -1.5 |
| ENSG00000112531.16 | QKI | - | - | -1.0 | -2.3 |
| ENSG00000112562.18 | SMOC2 | -5.1 | - | - | -1.3 |
| ENSG00000112658.7 | SRF | -1.7 | - | -1.0 | -1.3 |
| ENSG00000112769.18 | LAMA4 | -2.3 | - | -1.2 | -2.6 |
| ENSG00000112773.15 | FAM46A | -1.6 | - | - | -1.1 |
| ENSG00000112782.15 | CLIC5 | - | - | -2.7 | -3.0 |
| ENSG00000112812.15 | PRSS16 | 1.5 | -1.5 | - | - |
| ENSG00000112902.11 | SEMA5A | - | - | -2.0 | -2.4 |

| | | | | | |
|---|---|---|---|---|---|
| ENSG00000112936.18 | C7 | -8.1 | - | -1.4 | -1.2 |
| ENSG00000112964.13 | GHR | -3.3 | - | -1.8 | -2.7 |
| ENSG00000112984.11 | KIF20A | 3.0 | - | 1.9 | 2.2 |
| ENSG00000113070.7 | HBEGF | -1.7 | - | -2.8 | -1.1 |
| ENSG00000113319.11 | RASGRF2 | -1.8 | - | - | -1.9 |
| ENSG00000113555.5 | PCDH12 | - | - | -2.2 | -1.7 |
| ENSG00000113580.14 | NR3C1 | -2.0 | - | - | -2.2 |
| ENSG00000113594.9 | LIFR | -2.7 | - | -1.7 | -3.0 |
| ENSG00000113615.12 | SEC24A | - | - | 1.0 | -1.0 |
| ENSG00000113657.12 | DPYSL3 | -3.2 | - | - | -1.4 |
| ENSG00000113721.13 | PDGFRB | -2.1 | - | - | -1.8 |
| ENSG00000113810.15 | SMC4 | 1.4 | - | - | 1.6 |
| ENSG00000114019.14 | AMOTL2 | -2.2 | - | -1.4 | -2.7 |
| ENSG00000114115.9 | RBP1 | -3.5 | - | - | 1.0 |
| ENSG00000114200.9 | BCHE | -3.3 | - | -2.7 | - |
| ENSG00000114346.13 | ECT2 | 1.7 | - | 1.3 | - |
| ENSG00000114378.16 | HYAL1 | -1.6 | - | -1.6 | -1.4 |
| ENSG00000114631.10 | PODXL2 | 1.7 | - | 2.3 | - |
| ENSG00000114654.7 | EFCC1 | -2.3 | - | -3.3 | - |
| ENSG00000114698.14 | PLSCR4 | -2.8 | - | -1.4 | -2.6 |
| ENSG00000114790.12 | ARHGEF26 | -1.6 | 1.5 | -2.4 | - |
| ENSG00000114853.13 | ZBTB47 | -1.7 | - | -1.0 | -1.7 |
| ENSG00000114857.17 | NKTR | - | 1.3 | - | 1.0 |
| ENSG00000115107.19 | STEAP3 | 1.6 | -1.0 | 1.6 | -1.0 |
| ENSG00000115226.9 | FNDC4 | - | -1.2 | - | -2.2 |
| ENSG00000115255.10 | REEP6 | - | - | 1.5 | 1.1 |
| ENSG00000115306.15 | SPTBN1 | -1.2 | - | -1.8 | -3.4 |
| ENSG00000115380.18 | EFEMP1 | -6.3 | - | -1.4 | -2.1 |
| ENSG00000115468.11 | EFHD1 | -2.7 | - | 1.3 | - |
| ENSG00000115594.11 | IL1R1 | -2.1 | - | - | -2.0 |
| ENSG00000115641.18 | FHL2 | - | -1.0 | 1.6 | - |
| ENSG00000115758.12 | ODC1 | -1.9 | - | - | -1.5 |
| ENSG00000115884.10 | SDC1 | - | -2.1 | 1.4 | - |
| ENSG00000115896.15 | PLCL1 | - | - | -1.1 | -1.7 |
| ENSG00000115935.16 | WIPF1 | -1.8 | - | - | -1.6 |
| ENSG00000115947.13 | ORC4 | 1.0 | 1.2 | - | - |

| | | | | | |
|---|---|---|---|---|---|
| ENSG00000115956.9 | PLEK | -2.2 | - | - | -1.3 |
| ENSG00000115963.13 | RND3 | -1.4 | - | -1.1 | -1.1 |
| ENSG00000116016.13 | EPAS1 | -1.3 | -1.0 | -3.0 | -3.1 |
| ENSG00000116031.8 | CD207 | - | -1.9 | 2.8 | - |
| ENSG00000116117.17 | PARD3B | -1.5 | - | - | -1.2 |
| ENSG00000116194.12 | ANGPTL1 | -5.2 | - | -2.5 | -2.8 |
| ENSG00000116678.18 | LEPR | - | - | -1.5 | -1.3 |
| ENSG00000116711.9 | PLA2G4A | - | - | 1.2 | -2.9 |
| ENSG00000116741.7 | RGS2 | -4.5 | - | -2.2 | -1.6 |
| ENSG00000116962.14 | NID1 | -2.0 | - | - | -2.5 |
| ENSG00000117020.16 | AKT3 | -2.7 | - | -1.2 | - |
| ENSG00000117069.14 | ST6GAL-C5 | -3.2 | - | -2.3 | - |
| ENSG00000117114.19 | ADGRL2 | - | - | -2.6 | -2.0 |
| ENSG00000117122.13 | MFAP2 | - | - | 1.2 | 2.2 |
| ENSG00000117245.12 | KIF17 | - | - | -2.1 | -1.1 |
| ENSG00000117298.14 | ECE1 | -1.1 | - | - | -1.2 |
| ENSG00000117308.14 | GALE | 1.2 | - | 1.4 | 1.7 |
| ENSG00000117318.8 | ID3 | - | -1.7 | -1.8 | - |
| ENSG00000117399.13 | CDC20 | 2.3 | - | 1.8 | 2.1 |
| ENSG00000117472.9 | TSPAN1 | - | - | 2.3 | 1.6 |
| ENSG00000117479.12 | SLC19A2 | -1.8 | - | -1.1 | - |
| ENSG00000117519.15 | CNN3 | -2.1 | - | - | -1.1 |
| ENSG00000117525.13 | F3 | -2.6 | - | -1.0 | - |
| ENSG00000117632.20 | STMN1 | 1.5 | - | - | 1.1 |
| ENSG00000117724.12 | CENPF | 2.9 | - | 1.8 | 2.9 |
| ENSG00000117868.15 | ESYT2 | -1.2 | - | - | -1.1 |
| ENSG00000118094.11 | TREH | - | - | -1.3 | -1.8 |
| ENSG00000118257.16 | NRP2 | -3.2 | - | - | -1.2 |
| ENSG00000118407.14 | FILIP1 | -4.7 | - | -1.4 | -1.0 |
| ENSG00000118420.16 | UBE3D | 1.1 | 1.3 | - | - |
| ENSG00000118473.21 | SGIP1 | - | 1.0 | - | 1.3 |
| ENSG00000118495.18 | PLAGL1 | -3.3 | - | -1.0 | -2.0 |
| ENSG00000118496.4 | FBXO30 | -1.2 | - | - | -1.1 |
| ENSG00000118503.14 | TNFAIP3 | -2.8 | - | -1.9 | - |
| ENSG00000118508.4 | RAB32 | -1.6 | - | - | -1.2 |
| ENSG00000118515.11 | SGK1 | - | - | -1.1 | -1.7 |

| | | | | | |
|---|---|---|---|---|---|
| ENSG00000118523.5 | CTGF | -5.0 | - | -1.0 | -1.3 |
| ENSG00000118526.6 | TCF21 | -4.0 | - | -2.6 | - |
| ENSG00000118596.11 | SLC16A7 | - | - | 1.4 | -1.0 |
| ENSG00000118777.10 | ABCG2 | -1.6 | - | -1.8 | - |
| ENSG00000118804.8 | FAM47E-STBD1 | -1.8 | - | - | -2.5 |
| ENSG00000118922.16 | KLF12 | -1.2 | - | - | -1.4 |
| ENSG00000118971.7 | CCND2 | -2.2 | - | - | -2.3 |
| ENSG00000119138.4 | KLF9 | -3.8 | - | -1.9 | -2.3 |
| ENSG00000119147.9 | C2orf40 | -5.4 | -1.5 | -1.6 | -3.3 |
| ENSG00000119280.16 | C1orf198 | - | - | -1.1 | -1.2 |
| ENSG00000119326.14 | CTN-L1 | -2.0 | - | -1.9 | -1.6 |
| ENSG00000119333.11 | WDR34 | 1.1 | - | - | 1.3 |
| ENSG00000119508.17 | NR4A3 | -4.5 | - | -3.0 | - |
| ENSG00000119535.17 | CSF3R | -2.3 | - | -1.2 | - |
| ENSG00000119699.7 | TGFB3 | -3.9 | -1.3 | - | - |
| ENSG00000119729.10 | RHOQ | -1.2 | - | - | -1.9 |
| ENSG00000119862.12 | LGALSL | - | - | -1.5 | -1.1 |
| ENSG00000119865.8 | CNRIP1 | -3.9 | - | -1.3 | -2.1 |
| ENSG00000119866.20 | BCL11A | 1.6 | -1.4 | - | -1.6 |
| ENSG00000119900.7 | OGFRL1 | -1.2 | - | - | -1.3 |
| ENSG00000119950.20 | MXI1 | -1.2 | - | - | -1.2 |
| ENSG00000119969.14 | HELLS | 1.9 | - | 1.2 | 1.2 |
| ENSG00000119986.6 | AVPI1 | - | -1.7 | -1.5 | -1.6 |
| ENSG00000120093.11 | HOXB3 | 2.3 | - | 1.3 | - |
| ENSG00000120129.5 | DUSP1 | -2.9 | - | -2.2 | -1.8 |
| ENSG00000120156.20 | TEK | - | - | -2.8 | -2.6 |
| ENSG00000120279.6 | MYCT1 | -3.0 | - | -2.1 | -2.3 |
| ENSG00000120318.15 | ARAP3 | - | - | -1.8 | -1.2 |
| ENSG00000120594.16 | PLXDC2 | -1.7 | - | - | -1.0 |
| ENSG00000120645.11 | IQSEC3 | - | - | -2.0 | -2.8 |
| ENSG00000120693.13 | SMAD9 | -1.4 | - | -1.2 | -1.1 |
| ENSG00000120738.7 | EGR1 | -2.7 | - | -2.1 | -2.2 |
| ENSG00000120833.13 | SOCS2 | -2.0 | - | -1.7 | -1.0 |
| ENSG00000120885.19 | CLU | -2.1 | -1.1 | - | -1.2 |
| ENSG00000120896.13 | SORBS3 | -2.7 | - | -1.2 | - |
| ENSG00000120910.14 | PPP3CC | -1.4 | - | - | -1.1 |

| | | | | | |
|---|---|---|---|---|---|
| ENSG00000120913.23 | PDLIM2 | -1.8 | - | -1.6 | - |
| ENSG00000121152.9 | NCAPH | 2.1 | - | 1.3 | - |
| ENSG00000121210.15 | KIAA0922 | -2.2 | - | - | -1.3 |
| ENSG00000121297.6 | TSHZ3 | -2.4 | - | - | -1.1 |
| ENSG00000121361.3 | KCNJ8 | -2.5 | - | -1.5 | -2.7 |
| ENSG00000121380.12 | BCL2L14 | 1.2 | - | 1.4 | - |
| ENSG00000121440.14 | PDZRN3 | -4.9 | - | -1.1 | -2.2 |
| ENSG00000121552.3 | CSTA | - | -1.7 | - | -1.7 |
| ENSG00000121671.11 | CRY2 | -1.5 | - | - | -1.7 |
| ENSG00000121691.4 | CAT | - | - | -1.5 | -3.0 |
| ENSG00000121716.18 | PILRB | 1.6 | 1.1 | - | 1.2 |
| ENSG00000121753.12 | ADGRB2 | - | - | 1.1 | 1.4 |
| ENSG00000121769.7 | FABP3 | -2.4 | - | 2.0 | - |
| ENSG00000121898.12 | CPXM2 | -5.6 | - | - | -2.1 |
| ENSG00000122026.10 | RPL21 | -1.4 | - | - | -1.0 |
| ENSG00000122035.6 | RASL11A | -2.1 | -1.1 | - | - |
| ENSG00000122176.10 | FMOD | -3.4 | - | - | -2.0 |
| ENSG00000122483.17 | CCDC18 | 1.2 | 1.2 | - | - |
| ENSG00000122484.8 | RPAP2 | 1.0 | 1.3 | - | 1.4 |
| ENSG00000122490.18 | PQLC1 | -1.1 | - | - | -1.2 |
| ENSG00000122591.11 | FAM126A | -1.2 | - | - | -1.2 |
| ENSG00000122592.7 | HOXA7 | - | -1.4 | -1.3 | -2.5 |
| ENSG00000122642.10 | FKBP9 | -1.4 | - | - | -1.4 |
| ENSG00000122679.8 | RAMP3 | -2.7 | - | -3.5 | - |
| ENSG00000122691.12 | TWIST1 | -2.4 | 1.3 | - | -1.4 |
| ENSG00000122694.15 | GLIPR2 | -3.6 | - | -1.7 | -1.8 |
| ENSG00000122707.11 | RECK | -3.6 | - | -1.4 | -1.9 |
| ENSG00000122756.14 | CNTFR | - | - | -2.6 | -3.7 |
| ENSG00000122862.4 | SRGN | -3.3 | - | -1.3 | -1.3 |
| ENSG00000122863.5 | CHST3 | -1.4 | - | - | -2.5 |
| ENSG00000122877.13 | EGR2 | -3.9 | - | -1.9 | -2.2 |
| ENSG00000122952.16 | ZWINT | - | - | 1.4 | 2.1 |
| ENSG00000122966.13 | CIT | 1.5 | - | 2.7 | 1.1 |
| ENSG00000123094.15 | RASSF8 | -2.2 | - | -1.0 | - |
| ENSG00000123095.5 | BHLHE41 | 1.4 | - | - | -1.5 |
| ENSG00000123096.11 | SSPN | -1.6 | - | - | -1.3 |

| | | | | | |
|---|---|---|---|---|---|
| ENSG00000123146.19 | ADGRE5 | -1.3 | - | -1.6 | -1.6 |
| ENSG00000123219.12 | CENPK | 2.3 | - | 1.3 | 1.1 |
| ENSG00000123243.14 | ITIH5 | -3.7 | -1.0 | -2.1 | -2.9 |
| ENSG00000123342.15 | MMP19 | -2.0 | - | -2.5 | -1.9 |
| ENSG00000123358.19 | NR4A1 | -3.9 | - | -2.8 | - |
| ENSG00000123473.15 | STIL | 2.0 | - | 1.9 | - |
| ENSG00000123485.11 | HJURP | 2.2 | - | 1.9 | - |
| ENSG00000123500.9 | COL10A1 | - | - | 5.1 | 5.0 |
| ENSG00000123685.8 | BATF3 | -2.2 | - | -1.3 | - |
| ENSG00000123689.5 | G0S2 | -3.2 | - | -1.6 | -3.4 |
| ENSG00000124067.16 | SLC12A4 | -1.5 | - | - | -1.3 |
| ENSG00000124104.18 | SNX21 | -1.3 | - | - | -1.1 |
| ENSG00000124107.5 | SLPI | - | -1.4 | - | -1.2 |
| ENSG00000124126.13 | PREX1 | -1.7 | - | -1.4 | - |
| ENSG00000124191.17 | TOX2 | -3.5 | - | -2.2 | -1.1 |
| ENSG00000124212.5 | PTGIS | -6.3 | - | -1.2 | -1.9 |
| ENSG00000124215.16 | CDH26 | 2.2 | - | 1.7 | - |
| ENSG00000124216.3 | S-I1 | -3.8 | -1.1 | -2.0 | -1.0 |
| ENSG00000124440.15 | HIF3A | -4.4 | - | - | -1.9 |
| ENSG00000124491.15 | F13A1 | -3.4 | - | - | -1.9 |
| ENSG00000124615.17 | MOCS1 | - | - | -1.1 | -2.5 |
| ENSG00000124664.10 | SPDEF | - | - | 3.6 | 1.2 |
| ENSG00000124762.13 | CDKN1A | - | - | -1.5 | -1.2 |
| ENSG00000124766.5 | SOX4 | 2.7 | - | 1.3 | - |
| ENSG00000124785.8 | NRN1 | -3.8 | - | -1.9 | -2.6 |
| ENSG00000124839.12 | RAB17 | 1.3 | 1.2 | - | 1.1 |
| ENSG00000124942.13 | AH-K | -1.6 | - | -1.4 | -2.4 |
| ENSG00000125148.6 | MT2A | -4.8 | - | -2.3 | - |
| ENSG00000125266.6 | EFNB2 | - | - | -1.4 | -1.7 |
| ENSG00000125398.5 | SOX9 | - | - | 1.0 | -1.2 |
| ENSG00000125538.11 | IL1B | -3.2 | - | -2.5 | - |
| ENSG00000125730.16 | C3 | -2.9 | - | - | -2.2 |
| ENSG00000125740.13 | FOSB | -2.6 | - | -3.2 | -2.3 |
| ENSG00000125810.9 | CD93 | -2.4 | - | -2.1 | -2.6 |
| ENSG00000125827.8 | TMX4 | -1.1 | - | - | -1.1 |
| ENSG00000125845.6 | BMP2 | - | - | -1.5 | -3.3 |

| | | | | | |
|---|---|---|---|---|---|
| ENSG00000125895.5 | TMEM74B | 2.2 | - | -2.8 | - |
| ENSG00000125968.8 | ID1 | - | -2.2 | -2.3 | -1.8 |
| ENSG00000126016.13 | AMOT | - | -1.4 | 1.1 | -2.0 |
| ENSG00000126218.11 | F10 | -6.1 | - | -2.0 | - |
| ENSG00000126351.12 | THRA | -1.6 | - | -1.0 | -1.8 |
| ENSG00000126458.3 | RRAS | -2.0 | - | -1.6 | - |
| ENSG00000126561.16 | STAT5A | -1.0 | - | - | -2.2 |
| ENSG00000126709.14 | IFI6 | - | - | 1.1 | 1.9 |
| ENSG00000126759.12 | CFP | -2.1 | - | -2.6 | - |
| ENSG00000126785.12 | RHOJ | -3.2 | - | -2.0 | -1.4 |
| ENSG00000126803.9 | HSPA2 | -1.8 | -1.0 | -1.0 | - |
| ENSG00000126895.13 | AVPR2 | - | - | -2.7 | -2.3 |
| ENSG00000126947.11 | ARMCX1 | -2.3 | -1.6 | - | - |
| ENSG00000127084.17 | FGD3 | - | - | -1.1 | 1.6 |
| ENSG00000127241.16 | MASP1 | -5.5 | - | -3.5 | - |
| ENSG00000127329.14 | PTPRB | - | - | -2.8 | -1.6 |
| ENSG00000127415.12 | IDUA | 1.2 | - | - | 1.3 |
| ENSG00000127418.14 | FGFRL1 | - | 1.3 | - | -1.1 |
| ENSG00000127528.5 | KLF2 | -4.4 | - | -2.5 | -1.4 |
| ENSG00000127564.16 | PKMYT1 | 1.9 | - | 1.6 | 3.1 |
| ENSG00000127920.5 | GNG11 | -3.6 | - | -2.4 | -2.2 |
| ENSG00000127951.5 | FGL2 | -4.2 | - | - | -2.0 |
| ENSG00000127954.12 | STEAP4 | -2.5 | - | - | -1.4 |
| ENSG00000127990.15 | SGCE | -3.4 | - | -1.5 | -1.7 |
| ENSG00000128016.5 | ZFP36 | -2.5 | - | -2.2 | - |
| ENSG00000128050.8 | PAICS | 1.1 | - | 1.1 | - |
| ENSG00000128059.8 | PPAT | 1.1 | - | 1.3 | - |
| ENSG00000128266.8 | G-Z | - | -1.0 | - | -2.4 |
| ENSG00000128268.11 | MGAT3 | - | - | -2.4 | -3.2 |
| ENSG00000128283.6 | CDC42EP1 | -1.3 | - | - | -1.1 |
| ENSG00000128284.19 | APOL3 | - | - | -1.3 | -1.2 |
| ENSG00000128298.16 | BAIAP2L2 | 1.7 | - | - | 1.2 |
| ENSG00000128342.4 | LIF | -1.9 | - | -1.3 | - |
| ENSG00000128346.10 | C22orf23 | - | - | -1.2 | -1.2 |
| ENSG00000128422.15 | KRT17 | - | -1.9 | - | -1.8 |
| ENSG00000128482.15 | RNF112 | -3.1 | - | -1.5 | - |

| | | | | | |
|---|---|---|---|---|---|
| ENSG00000128512.19 | DOCK4 | -1.5 | - | -1.4 | -1.4 |
| ENSG00000128567.16 | PODXL | - | - | -1.3 | -1.9 |
| ENSG00000128591.15 | FLNC | -8.3 | -1.2 | -1.5 | -2.4 |
| ENSG00000128602.9 | SMO | -2.0 | - | - | -1.3 |
| ENSG00000128606.12 | LRRC17 | -1.8 | - | 1.2 | - |
| ENSG00000128849.10 | CGNL1 | -3.1 | - | -1.7 | -1.0 |
| ENSG00000128917.6 | DLL4 | - | - | -1.9 | -1.3 |
| ENSG00000129009.12 | ISLR | -5.0 | - | - | -1.1 |
| ENSG00000129235.10 | TXNDC17 | 1.3 | - | 1.4 | - |
| ENSG00000129244.8 | ATP1B2 | -3.4 | - | -1.4 | - |
| ENSG00000129250.11 | KIF1C | -1.1 | - | -1.3 | -1.4 |
| ENSG00000129295.8 | LRRC6 | - | - | 1.2 | 1.0 |
| ENSG00000129467.13 | ADCY4 | -1.8 | - | -2.0 | -1.0 |
| ENSG00000129473.9 | BCL2L2 | -1.2 | - | - | -1.4 |
| ENSG00000129538.13 | R-SE1 | -2.8 | - | - | -1.7 |
| ENSG00000129596.4 | CDO1 | -6.7 | - | -2.8 | -3.6 |
| ENSG00000129657.14 | SEC14L1 | -1.2 | - | -1.1 | -1.5 |
| ENSG00000129680.15 | MAP7D3 | -1.4 | - | - | -1.4 |
| ENSG00000129757.12 | CDKN1C | -2.4 | - | -1.5 | -2.3 |
| ENSG00000129993.14 | CBFA2T3 | -1.6 | - | -1.6 | - |
| ENSG00000130052.13 | STARD8 | -2.0 | - | -2.3 | -1.6 |
| ENSG00000130176.7 | CNN1 | -8.6 | -1.1 | -1.6 | -2.1 |
| ENSG00000130193.7 | THEM6 | 1.2 | - | 1.3 | - |
| ENSG00000130203.9 | APOE | -2.3 | 1.2 | - | - |
| ENSG00000130222.10 | GADD45G | -3.6 | - | - | 2.2 |
| ENSG00000130224.14 | LRCH2 | -2.6 | - | -1.2 | - |
| ENSG00000130300.8 | PLVAP | -1.7 | - | - | -1.4 |
| ENSG00000130508.10 | PXDN | -1.9 | - | - | -1.3 |
| ENSG00000130513.6 | GDF15 | - | - | 2.5 | 2.2 |
| ENSG00000130522.5 | JUND | -2.0 | - | -1.8 | - |
| ENSG00000130558.18 | OLFM1 | -4.9 | - | -1.2 | - |
| ENSG00000130584.10 | ZBTB46 | -1.0 | - | - | -1.1 |
| ENSG00000130590.13 | SAMD10 | 1.6 | - | 1.4 | - |
| ENSG00000130635.15 | COL5A1 | -2.4 | - | 1.4 | - |
| ENSG00000130701.3 | RBBP8NL | 1.9 | - | - | 1.6 |
| ENSG00000130762.14 | ARHGEF16 | 1.5 | - | 1.4 | 1.3 |

| | | | | | |
|---|---|---|---|---|---|
| ENSG00000130775.15 | THEMIS2 | -2.4 | - | -1.1 | - |
| ENSG00000130830.14 | MPP1 | -2.9 | - | - | -1.1 |
| ENSG00000130844.16 | ZNF331 | -2.7 | - | -2.3 | - |
| ENSG00000130956.13 | HABP4 | -2.0 | - | - | -1.5 |
| ENSG00000130988.12 | RGN | -3.5 | - | - | -1.6 |
| ENSG00000131016.16 | AKAP12 | -3.3 | - | -2.1 | -2.6 |
| ENSG00000131018.22 | SYNE1 | -3.0 | - | - | -1.1 |
| ENSG00000131042.13 | LILRB2 | -2.2 | - | -1.9 | - |
| ENSG00000131055.4 | COX4I2 | - | - | -3.1 | -1.7 |
| ENSG00000131153.8 | GINS2 | 2.0 | - | - | 1.6 |
| ENSG00000131187.9 | F12 | 2.4 | - | 2.0 | 2.3 |
| ENSG00000131196.17 | NFATC1 | -2.2 | - | -1.6 | -1.9 |
| ENSG00000131370.15 | SH3BP5 | - | - | -1.8 | -1.3 |
| ENSG00000131378.13 | RFTN1 | -1.5 | - | - | -1.8 |
| ENSG00000131409.12 | LRRC4B | - | - | -2.2 | -1.5 |
| ENSG00000131435.12 | PDLIM4 | -1.9 | -1.0 | - | - |
| ENSG00000131459.12 | GFPT2 | -5.7 | - | -1.4 | -1.1 |
| ENSG00000131471.6 | AOC3 | -4.2 | - | -2.6 | -3.9 |
| ENSG00000131477.10 | RAMP2 | -2.2 | - | -3.0 | -1.1 |
| ENSG00000131591.17 | C1orf159 | 1.0 | - | - | 1.2 |
| ENSG00000131711.14 | MAP1B | -3.7 | - | - | -1.6 |
| ENSG00000131730.15 | CKMT2 | -2.3 | - | - | -2.0 |
| ENSG00000131746.12 | TNS4 | 1.8 | -2.1 | 2.1 | -1.3 |
| ENSG00000131747.14 | TOP2A | 3.3 | 1.7 | 2.4 | 2.9 |
| ENSG00000131771.13 | PPP1R1B | - | - | 1.5 | -1.3 |
| ENSG00000131773.13 | KHDRBS3 | -2.0 | - | - | -1.0 |
| ENSG00000131831.17 | RAI2 | -3.2 | - | -1.8 | - |
| ENSG00000131873.5 | CHSY1 | -1.5 | - | - | -1.1 |
| ENSG00000132170.19 | PPARG | - | - | -1.4 | -3.3 |
| ENSG00000132205.10 | EMILIN2 | -1.0 | - | - | -2.1 |
| ENSG00000132329.10 | RAMP1 | -4.9 | - | 1.7 | 1.5 |
| ENSG00000132376.19 | INPP5K | - | - | -1.1 | -1.2 |
| ENSG00000132386.10 | SERPINF1 | -3.5 | - | - | -1.5 |
| ENSG00000132470.13 | ITGB4 | 1.1 | -2.2 | 1.3 | - |
| ENSG00000132613.14 | MTSS1L | - | -1.0 | - | -1.1 |
| ENSG00000132622.10 | HSPA12B | -3.3 | - | -2.9 | -2.2 |

| | | | | | |
|---|---|---|---|---|---|
| ENSG00000132688.10 | NES | -2.4 | - | -2.4 | -1.5 |
| ENSG00000132698.13 | RAB25 | 1.8 | - | - | 1.1 |
| ENSG00000132718.8 | SYT11 | -3.1 | - | - | -1.1 |
| ENSG00000132746.14 | ALDH3B2 | - | 2.6 | 3.9 | - |
| ENSG00000132749.10 | MTL5 | 2.0 | - | - | 1.6 |
| ENSG00000132819.16 | RBM38 | -1.9 | -1.2 | - | - |
| ENSG00000132821.11 | VSTM2L | - | 1.5 | 1.9 | - |
| ENSG00000132840.9 | BHMT2 | -5.3 | - | - | -1.6 |
| ENSG00000132854.18 | KANK4 | - | - | -1.5 | -2.2 |
| ENSG00000132965.9 | ALOX5AP | -2.7 | - | - | -1.1 |
| ENSG00000132970.12 | WASF3 | -1.9 | - | -1.4 | -3.3 |
| ENSG00000133056.13 | PIK3C2B | 1.2 | - | - | -1.3 |
| ENSG00000133083.14 | DCLK1 | -4.0 | - | - | -1.4 |
| ENSG00000133104.12 | SPG20 | -1.8 | - | - | -1.6 |
| ENSG00000133121.20 | STARD13 | -2.3 | - | -1.7 | -1.5 |
| ENSG00000133138.19 | TBC1D8B | - | 1.5 | 1.0 | 1.3 |
| ENSG00000133142.17 | TCEAL4 | -1.6 | - | - | 1.2 |
| ENSG00000133169.5 | BEX1 | - | -1.7 | -2.6 | - |
| ENSG00000133195.11 | SLC39A11 | 1.2 | - | 1.5 | - |
| ENSG00000133392.16 | MYH11 | -7.7 | - | -1.2 | -1.8 |
| ENSG00000133401.15 | PDZD2 | - | - | -2.0 | -2.5 |
| ENSG00000133466.13 | C1QTNF6 | - | - | 1.2 | 1.2 |
| ENSG00000133561.15 | GIMAP6 | -1.4 | - | -1.8 | -2.9 |
| ENSG00000133574.9 | GIMAP4 | -2.2 | - | -1.1 | -1.5 |
| ENSG00000133687.15 | TMTC1 | -2.9 | - | -1.0 | -2.3 |
| ENSG00000133740.10 | E2F5 | 1.1 | - | 1.2 | - |
| ENSG00000133800.8 | LYVE1 | -5.7 | - | -3.2 | -4.0 |
| ENSG00000133874.1 | RNF122 | -2.5 | - | -2.2 | -1.0 |
| ENSG00000134030.13 | CTIF | - | - | -1.1 | -1.5 |
| ENSG00000134057.14 | CCNB1 | 2.0 | - | 1.8 | 1.5 |
| ENSG00000134072.10 | CAMK1 | -1.4 | - | - | -1.4 |
| ENSG00000134121.9 | CHL1 | - | - | 1.2 | -3.2 |
| ENSG00000134138.19 | MEIS2 | -1.9 | - | -1.2 | -1.2 |
| ENSG00000134198.9 | TSPAN2 | -2.2 | - | -1.1 | - |
| ENSG00000134201.10 | GSTM5 | -5.3 | - | -2.0 | -3.9 |
| ENSG00000134222.16 | PSRC1 | 1.2 | - | - | 1.7 |

| | | | | | |
|---|---|---|---|---|---|
| ENSG00000134245.17 | WNT2B | -2.1 | - | -1.3 | - |
| ENSG00000134259.3 | NGF | -3.6 | - | -1.2 | - |
| ENSG00000134317.17 | GRHL1 | 1.5 | - | 1.5 | - |
| ENSG00000134398.12 | ERN2 | 2.4 | - | 3.9 | - |
| ENSG00000134463.14 | ECHDC3 | - | -1.2 | - | -1.5 |
| ENSG00000134516.15 | DOCK2 | -2.0 | - | - | -1.5 |
| ENSG00000134531.9 | EMP1 | -3.4 | - | -1.9 | -3.1 |
| ENSG00000134532.15 | SOX5 | - | - | -1.0 | -2.3 |
| ENSG00000134569.9 | LRP4 | - | -1.4 | -1.4 | -1.6 |
| ENSG00000134668.12 | SPOCD1 | 2.4 | - | - | 2.0 |
| ENSG00000134690.10 | CDCA8 | 2.3 | - | 1.9 | 2.0 |
| ENSG00000134716.9 | CYP2J2 | - | 1.3 | 1.7 | - |
| ENSG00000134802.17 | SLC43A3 | -1.8 | - | - | -1.6 |
| ENSG00000134853.11 | PDGFRA | -3.3 | - | - | -1.9 |
| ENSG00000134917.9 | ADAMTS8 | -3.7 | - | -3.0 | - |
| ENSG00000134954.14 | ETS1 | - | - | -1.3 | -2.2 |
| ENSG00000135046.13 | ANXA1 | - | -1.0 | -1.2 | -2.5 |
| ENSG00000135047.14 | CTSL | -1.7 | - | - | -1.1 |
| ENSG00000135048.13 | TMEM2 | - | - | -1.0 | -1.3 |
| ENSG00000135049.15 | AGTPBP1 | - | - | -1.2 | -1.0 |
| ENSG00000135052.16 | GOLM1 | - | 1.2 | 2.3 | - |
| ENSG00000135063.17 | FAM189A2 | -2.5 | - | -2.0 | -1.4 |
| ENSG00000135111.14 | TBX3 | 1.3 | - | -2.9 | - |
| ENSG00000135119.14 | RNFT2 | 2.0 | - | - | 1.0 |
| ENSG00000135218.17 | CD36 | -3.4 | - | -2.6 | -3.8 |
| ENSG00000135318.11 | NT5E | - | - | 1.9 | -2.3 |
| ENSG00000135363.11 | LMO2 | - | - | -1.6 | -1.4 |
| ENSG00000135373.12 | EHF | - | - | 1.8 | -1.7 |
| ENSG00000135374.9 | ELF5 | - | - | 1.8 | -2.6 |
| ENSG00000135424.15 | ITGA7 | -4.0 | - | - | -3.2 |
| ENSG00000135447.16 | PPP1R1A | - | -1.0 | - | -3.6 |
| ENSG00000135451.12 | TROAP | 2.9 | - | 1.3 | 2.8 |
| ENSG00000135476.11 | ESPL1 | 2.7 | - | - | 1.7 |
| ENSG00000135540.11 | NHSL1 | 1.4 | - | -1.0 | -1.2 |
| ENSG00000135549.14 | PKIB | - | 1.1 | 1.9 | - |
| ENSG00000135604.9 | STX11 | -3.1 | - | -2.8 | -3.6 |

| | | | | | |
|---|---|---|---|---|---|
| ENSG00000135636.13 | DYSF | -2.2 | - | - | -1.7 |
| ENSG00000135678.11 | CPM | - | -1.1 | - | -1.9 |
| ENSG00000135722.8 | FBXL8 | 1.4 | - | 1.0 | - |
| ENSG00000135750.14 | KCNK1 | - | - | 1.0 | 1.5 |
| ENSG00000135835.10 | KIAA1614 | - | - | -1.1 | -2.1 |
| ENSG00000135842.16 | FAM129A | -4.0 | - | - | -1.4 |
| ENSG00000135862.5 | LAMC1 | -1.1 | - | - | -2.5 |
| ENSG00000135916.15 | ITM2C | - | -1.4 | - | -1.5 |
| ENSG00000135917.13 | SLC19A3 | - | - | -2.5 | -3.2 |
| ENSG00000135919.12 | SERPINE2 | -2.3 | - | 1.2 | - |
| ENSG00000135929.8 | CYP27A1 | -2.6 | -1.3 | -1.1 | - |
| ENSG00000135951.14 | TSGA10 | 1.2 | 1.1 | - | - |
| ENSG00000135976.16 | ANKRD36 | - | 1.5 | - | 1.7 |
| ENSG00000136114.15 | THSD1 | - | - | -2.4 | -1.3 |
| ENSG00000136141.14 | LRCH1 | -1.4 | - | - | -1.4 |
| ENSG00000136143.13 | SUCLA2 | -1.0 | - | - | -1.8 |
| ENSG00000136158.10 | SPRY2 | -1.7 | - | - | -3.0 |
| ENSG00000136160.14 | EDNRB | -2.9 | - | -3.2 | -3.4 |
| ENSG00000136167.13 | LCP1 | -1.6 | - | - | -1.3 |
| ENSG00000136193.16 | SCRN1 | -1.2 | -1.1 | - | -1.1 |
| ENSG00000136237.18 | RAPGEF5 | - | - | -1.3 | -1.4 |
| ENSG00000136244.11 | IL6 | -6.8 | - | -4.5 | - |
| ENSG00000136274.8 | -CAD | -3.0 | - | -1.2 | -2.3 |
| ENSG00000136425.12 | CIB2 | - | -1.7 | - | -2.0 |
| ENSG00000136457.9 | CHAD | - | - | 1.4 | 1.0 |
| ENSG00000136546.13 | SCN7A | -6.6 | - | -2.0 | - |
| ENSG00000136630.12 | HLX | -1.4 | - | -1.8 | - |
| ENSG00000136717.14 | BIN1 | -3.6 | - | - | -2.5 |
| ENSG00000136732.14 | GYPC | -4.5 | - | -1.7 | -2.5 |
| ENSG00000136802.11 | LRRC8A | - | - | -1.0 | -1.3 |
| ENSG00000136826.14 | KLF4 | -2.6 | - | -3.3 | -2.5 |
| ENSG00000136840.18 | ST6GAL-C4 | -1.3 | -1.2 | - | - |
| ENSG00000136842.13 | TMOD1 | -4.7 | - | -2.0 | -2.8 |
| ENSG00000136848.16 | DAB2IP | - | -1.0 | -1.3 | -1.8 |
| ENSG00000136854.17 | STXBP1 | -3.0 | - | - | -1.8 |
| ENSG00000136859.9 | ANGPTL2 | -3.4 | - | - | -2.9 |

| | | | | | |
|---|---|---|---|---|---|
| ENSG00000136883.12 | KIF12 | - | - | 1.4 | 1.5 |
| ENSG00000136960.12 | ENPP2 | -2.0 | - | -1.5 | -3.2 |
| ENSG00000136997.14 | MYC | -2.0 | - | -1.8 | -1.8 |
| ENSG00000136999.4 | NOV | - | - | -1.2 | -1.5 |
| ENSG00000137033.11 | IL33 | - | - | -1.4 | -2.2 |
| ENSG00000137054.15 | POLR1E | -1.1 | - | - | -1.3 |
| ENSG00000137070.17 | IL11RA | -1.4 | - | -1.1 | -1.2 |
| ENSG00000137076.18 | TLN1 | -2.0 | - | -1.0 | -2.0 |
| ENSG00000137077.7 | CCL21 | -6.0 | - | -1.4 | -1.5 |
| ENSG00000137094.14 | D-JB5 | -4.0 | -1.0 | - | - |
| ENSG00000137124.6 | ALDH1B1 | -2.9 | - | 1.0 | - |
| ENSG00000137193.13 | PIM1 | - | - | -1.2 | -2.1 |
| ENSG00000137203.10 | TFAP2A | 1.7 | - | 3.8 | 1.1 |
| ENSG00000137267.5 | TUBB2A | -2.3 | - | - | -1.5 |
| ENSG00000137331.11 | IER3 | -2.4 | -1.3 | - | 1.5 |
| ENSG00000137393.9 | RNF144B | - | - | -1.1 | -1.1 |
| ENSG00000137460.8 | FHDC1 | - | -1.3 | -1.0 | - |
| ENSG00000137486.16 | ARRB1 | -1.8 | - | -1.4 | -1.0 |
| ENSG00000137491.14 | SLCO2B1 | -2.2 | - | - | -1.4 |
| ENSG00000137507.11 | LRRC32 | -3.0 | - | -2.4 | -1.8 |
| ENSG00000137573.13 | SULF1 | -3.0 | - | 2.4 | - |
| ENSG00000137648.16 | TMPRSS4 | 2.0 | - | 4.6 | - |
| ENSG00000137672.12 | TRPC6 | - | - | -1.2 | -1.6 |
| ENSG00000137673.8 | MMP7 | - | - | 2.6 | -1.3 |
| ENSG00000137699.16 | TRIM29 | - | -2.6 | - | -1.9 |
| ENSG00000137726.15 | FXYD6 | -4.9 | - | -2.3 | -1.3 |
| ENSG00000137801.10 | THBS1 | -3.3 | - | -1.3 | -1.5 |
| ENSG00000137804.12 | NUSAP1 | 2.1 | 1.5 | 1.3 | 1.8 |
| ENSG00000137807.13 | KIF23 | 1.7 | - | - | 1.7 |
| ENSG00000137809.16 | ITGA11 | -2.8 | - | 2.9 | - |
| ENSG00000137834.14 | SMAD6 | - | - | -2.6 | -1.5 |
| ENSG00000137857.17 | DUOX1 | - | -2.2 | -2.0 | - |
| ENSG00000137868.18 | STRA6 | 2.1 | - | 3.2 | - |
| ENSG00000137872.15 | SEMA6D | - | - | -2.1 | -2.1 |
| ENSG00000137878.16 | GCOM1 | - | - | -2.8 | -2.4 |
| ENSG00000137941.16 | TTLL7 | -1.8 | - | -1.8 | - |

| | | | | | |
|---|---|---|---|---|---|
| ENSG00000138061.11 | CYP1B1 | -3.9 | - | 1.2 | -1.1 |
| ENSG00000138135.6 | CH25H | -4.9 | - | -1.6 | - |
| ENSG00000138172.10 | CALHM2 | -1.7 | - | -1.0 | - |
| ENSG00000138180.15 | CEP55 | 2.2 | - | 1.5 | - |
| ENSG00000138182.14 | KIF20B | 1.1 | - | - | 1.1 |
| ENSG00000138207.12 | RBP4 | -6.5 | - | -1.8 | -4.0 |
| ENSG00000138346.14 | D-2 | 1.4 | - | 1.3 | 1.3 |
| ENSG00000138356.13 | AOX1 | -7.6 | -2.0 | -1.5 | -2.9 |
| ENSG00000138411.10 | HECW2 | - | - | -1.7 | -1.3 |
| ENSG00000138413.13 | IDH1 | - | - | 1.0 | -1.3 |
| ENSG00000138639.17 | ARHGAP24 | -2.2 | - | - | -1.6 |
| ENSG00000138641.15 | HERC3 | -1.3 | - | - | -1.5 |
| ENSG00000138678.10 | AGPAT9 | -2.6 | - | -1.2 | -2.5 |
| ENSG00000138685.12 | FGF2 | -4.0 | - | -1.9 | -3.1 |
| ENSG00000138722.9 | MMRN1 | -4.3 | - | -1.8 | - |
| ENSG00000138735.15 | PDE5A | -2.8 | - | -1.7 | - |
| ENSG00000138772.12 | ANXA3 | - | - | -1.5 | -1.1 |
| ENSG00000138778.11 | CENPE | 2.2 | - | - | 3.0 |
| ENSG00000138821.12 | SLC39A8 | - | - | -2.1 | -1.1 |
| ENSG00000139112.10 | GABARAPL1 | -2.6 | - | -1.3 | -2.1 |
| ENSG00000139117.13 | CPNE8 | -1.4 | - | - | -1.5 |
| ENSG00000139180.10 | NDUFA9 | 1.4 | 1.0 | - | - |
| ENSG00000139263.11 | LRIG3 | - | - | 1.6 | -1.9 |
| ENSG00000139278.9 | GLIPR1 | -2.7 | - | - | -1.1 |
| ENSG00000139567.12 | ACVRL1 | -2.2 | - | -2.7 | -2.1 |
| ENSG00000139629.15 | GALNT6 | - | - | 1.5 | 1.3 |
| ENSG00000139865.16 | TTC6 | - | 1.2 | - | 1.5 |
| ENSG00000139946.9 | PELI2 | -2.1 | - | - | -2.1 |
| ENSG00000139970.16 | RTN1 | -1.8 | - | -1.5 | -1.8 |
| ENSG00000139974.15 | SLC38A6 | - | 1.1 | - | 1.2 |
| ENSG00000140022.9 | STON2 | 1.6 | - | - | -1.1 |
| ENSG00000140044.12 | JDP2 | -2.0 | - | -1.5 | - |
| ENSG00000140092.14 | FBLN5 | -4.6 | - | -1.5 | -2.0 |
| ENSG00000140105.17 | WARS | - | - | -1.3 | -1.0 |
| ENSG00000140254.12 | DUOXA1 | - | -2.6 | -1.7 | - |
| ENSG00000140263.13 | SORD | 1.5 | - | 1.3 | - |

| | | | | | |
|---|---|---|---|---|---|
| ENSG00000140285.9 | FGF7 | -4.8 | - | - | -1.7 |
| ENSG00000140287.10 | HDC | -4.5 | - | -1.2 | - |
| ENSG00000140398.13 | NEIL1 | - | 1.2 | - | 1.0 |
| ENSG00000140451.12 | PIF1 | 1.7 | - | - | 1.1 |
| ENSG00000140525.17 | FANCI | 1.8 | - | - | 1.1 |
| ENSG00000140538.16 | NTRK3 | -6.0 | - | -1.3 | - |
| ENSG00000140545.14 | MFGE8 | -2.6 | - | - | -1.9 |
| ENSG00000140682.18 | TGFB1I1 | -4.0 | - | -1.0 | -1.0 |
| ENSG00000140832.9 | MARVELD3 | 1.2 | - | 1.4 | - |
| ENSG00000140941.12 | MAP1LC3B | -1.2 | - | - | -1.1 |
| ENSG00000140945.15 | CDH13 | - | - | -1.8 | -1.8 |
| ENSG00000140968.10 | IRF8 | -2.8 | - | - | -2.1 |
| ENSG00000141338.13 | ABCA8 | -2.9 | - | -2.1 | -1.6 |
| ENSG00000141404.15 | G-L | -2.2 | - | - | -1.2 |
| ENSG00000141447.16 | OSBPL1A | -1.4 | - | - | -1.5 |
| ENSG00000141448.8 | GATA6 | -3.6 | - | -1.9 | -1.5 |
| ENSG00000141469.16 | SLC14A1 | - | -2.0 | -2.0 | - |
| ENSG00000141519.14 | CCDC40 | - | 1.0 | - | 1.1 |
| ENSG00000141527.16 | CARD14 | 1.4 | - | 1.6 | - |
| ENSG00000141570.10 | CBX8 | 1.1 | - | - | 1.4 |
| ENSG00000141576.14 | RNF157 | - | 1.5 | - | -1.4 |
| ENSG00000141579.6 | ZNF750 | - | -2.2 | - | -1.2 |
| ENSG00000141750.6 | STAC2 | - | -1.0 | - | -1.8 |
| ENSG00000141934.9 | PPAP2C | 1.2 | - | 2.6 | - |
| ENSG00000142192.20 | APP | -1.2 | - | - | -1.4 |
| ENSG00000142227.10 | EMP3 | -4.2 | - | -1.4 | - |
| ENSG00000142252.10 | GEMIN7 | 1.4 | - | - | 1.2 |
| ENSG00000142273.10 | CBLC | 1.8 | - | 2.9 | 1.4 |
| ENSG00000142583.17 | SLC2A5 | - | -2.9 | 1.3 | - |
| ENSG00000142627.12 | EPHA2 | - | -1.7 | - | -2.1 |
| ENSG00000142632.16 | ARHGEF19 | 2.0 | -1.3 | 1.5 | - |
| ENSG00000142675.17 | CNKSR1 | 1.9 | - | - | 1.1 |
| ENSG00000142731.10 | PLK4 | 2.0 | - | 1.1 | - |
| ENSG00000142748.12 | FCN3 | - | - | -5.2 | -1.8 |
| ENSG00000142871.15 | CYR61 | -5.0 | - | -1.4 | -1.5 |
| ENSG00000142910.15 | TI-GL1 | - | - | -1.2 | -2.0 |

| | | | | | |
|---|---|---|---|---|---|
| ENSG00000142945.12 | KIF2C | 2.5 | - | 1.8 | - |
| ENSG00000142973.12 | CYP4B1 | - | -2.7 | -2.2 | - |
| ENSG00000143110.11 | C1orf162 | -2.4 | - | -1.2 | - |
| ENSG00000143153.12 | ATP1B1 | 1.9 | -1.1 | 1.5 | - |
| ENSG00000143196.4 | DPT | -9.4 | - | -2.2 | -3.9 |
| ENSG00000143248.12 | RGS5 | -2.2 | - | - | -2.0 |
| ENSG00000143320.8 | CRABP2 | - | -2.6 | 3.6 | 1.9 |
| ENSG00000143344.15 | RGL1 | -2.2 | - | - | -2.4 |
| ENSG00000143369.14 | ECM1 | -1.8 | - | - | 2.1 |
| ENSG00000143382.13 | ADAMTSL4 | -3.3 | - | -1.6 | -1.8 |
| ENSG00000143384.12 | MCL1 | -1.5 | - | - | -1.1 |
| ENSG00000143479.15 | DYRK3 | -2.5 | - | - | -1.1 |
| ENSG00000143578.15 | CREB3L4 | - | - | 1.2 | 1.6 |
| ENSG00000143590.13 | EF-3 | 1.5 | - | 2.1 | 1.5 |
| ENSG00000143630.9 | HCN3 | 1.9 | - | - | 1.0 |
| ENSG00000143772.9 | ITPKB | -1.7 | - | -1.0 | -1.9 |
| ENSG00000143867.6 | OSR1 | - | - | -1.6 | -2.1 |
| ENSG00000143878.9 | RHOB | -2.0 | -1.1 | -1.6 | - |
| ENSG00000143995.19 | MEIS1 | -1.6 | - | -1.4 | - |
| ENSG00000144031.11 | ANKRD53 | -2.9 | - | -1.2 | -2.3 |
| ENSG00000144218.18 | AFF3 | -3.5 | - | -1.6 | - |
| ENSG00000144230.16 | GPR17 | - | - | -2.9 | -1.6 |
| ENSG00000144331.18 | ZNF385B | -5.2 | - | -1.1 | - |
| ENSG00000144476.5 | ACKR3 | -2.3 | - | -1.7 | -1.6 |
| ENSG00000144485.10 | HES6 | - | - | 1.1 | 1.4 |
| ENSG00000144642.20 | RBMS3 | -2.5 | - | -1.0 | -2.5 |
| ENSG00000144655.14 | CSRNP1 | -2.8 | - | -2.9 | -1.9 |
| ENSG00000144668.11 | ITGA9 | -2.9 | - | - | -2.2 |
| ENSG00000144681.10 | STAC | -3.8 | - | -1.3 | -2.3 |
| ENSG00000144712.11 | CAND2 | - | -1.0 | - | -1.1 |
| ENSG00000144724.18 | PTPRG | -1.0 | - | - | -2.1 |
| ENSG00000144749.13 | LRIG1 | -2.6 | - | - | -1.5 |
| ENSG00000144824.19 | PHLDB2 | - | - | -1.4 | -1.3 |
| ENSG00000144843.11 | ADPRH | -2.2 | - | -1.2 | -1.9 |
| ENSG00000144857.14 | BOC | -4.8 | - | - | -2.3 |
| ENSG00000144891.17 | AGTR1 | -2.6 | 1.1 | -2.6 | -1.2 |

| | | | | | |
|---|---|---|---|---|---|
| ENSG00000144908.13 | ALDH1L1 | - | - | 1.5 | -3.2 |
| ENSG00000144935.14 | TRPC1 | -2.3 | - | - | -1.1 |
| ENSG00000145147.19 | SLIT2 | -5.8 | - | -2.4 | -2.2 |
| ENSG00000145247.11 | OCIAD2 | 1.4 | - | 2.3 | - |
| ENSG00000145284.11 | SCD5 | -1.9 | - | -1.2 | - |
| ENSG00000145335.15 | SNCA | -2.5 | - | -1.3 | -1.6 |
| ENSG00000145349.16 | CAMK2D | -1.0 | - | - | -1.6 |
| ENSG00000145362.16 | ANK2 | -4.2 | - | -1.2 | -2.4 |
| ENSG00000145386.9 | CC-2 | 2.0 | 1.6 | 1.3 | - |
| ENSG00000145391.13 | SETD7 | -1.6 | - | - | -1.7 |
| ENSG00000145423.4 | SFRP2 | -8.0 | - | 2.1 | 1.1 |
| ENSG00000145431.10 | PDGFC | -2.6 | - | 1.0 | -1.9 |
| ENSG00000145623.12 | OSMR | -2.3 | - | - | -1.1 |
| ENSG00000145730.20 | PAM | -2.7 | - | - | -1.9 |
| ENSG00000145861.7 | C1QTNF2 | -4.1 | - | -2.5 | - |
| ENSG00000145911.5 | N4BP3 | - | - | -1.6 | 1.2 |
| ENSG00000145990.10 | GFOD1 | -1.6 | - | -2.1 | -1.0 |
| ENSG00000146072.6 | TNFRSF21 | - | -1.0 | 1.3 | -2.5 |
| ENSG00000146122.16 | DAAM2 | -2.6 | - | - | -2.4 |
| ENSG00000146278.10 | PNRC1 | -1.8 | - | -1.1 | -1.4 |
| ENSG00000146350.13 | TBC1D32 | 1.3 | - | - | 1.4 |
| ENSG00000146374.13 | RSPO3 | -6.3 | - | -1.2 | -1.7 |
| ENSG00000146535.13 | G-12 | -1.7 | - | - | -1.6 |
| ENSG00000146555.18 | SDK1 | - | 1.8 | - | -2.8 |
| ENSG00000146592.16 | CREB5 | -4.5 | - | -1.3 | -2.1 |
| ENSG00000146670.9 | CDCA5 | 1.6 | - | 1.5 | 1.5 |
| ENSG00000146674.14 | IGFBP3 | - | - | 1.5 | -1.1 |
| ENSG00000146859.6 | TMEM140 | -1.1 | - | - | -2.1 |
| ENSG00000146966.12 | DENND2A | -2.6 | - | -2.1 | -1.6 |
| ENSG00000147027.3 | TMEM47 | - | - | -1.3 | -2.4 |
| ENSG00000147065.16 | MSN | -2.0 | - | - | -1.7 |
| ENSG00000147100.9 | SLC16A2 | -2.4 | -1.1 | - | -1.5 |
| ENSG00000147113.16 | CXorf36 | -2.2 | - | -2.0 | -2.0 |
| ENSG00000147119.3 | CHST7 | -2.3 | - | - | -2.2 |
| ENSG00000147251.15 | DOCK11 | -2.5 | - | - | -3.5 |
| ENSG00000147257.13 | GPC3 | -2.1 | - | -2.7 | -3.9 |

| | | | | | |
|---|---|---|---|---|---|
| ENSG00000147408.14 | CSGAL-CT1 | -3.6 | - | - | -1.2 |
| ENSG00000147443.12 | DOK2 | -2.2 | - | -1.3 | -1.7 |
| ENSG00000147526.19 | TACC1 | -1.3 | - | -1.4 | -2.3 |
| ENSG00000147689.16 | FAM83A | 3.4 | - | 5.3 | - |
| ENSG00000147799.11 | ARHGAP39 | 1.6 | - | 1.2 | - |
| ENSG00000147804.9 | SLC39A4 | 1.3 | - | 1.4 | - |
| ENSG00000147862.14 | NFIB | -1.5 | - | - | -2.1 |
| ENSG00000147889.16 | CDKN2A | - | - | 1.4 | 1.1 |
| ENSG00000148053.15 | NTRK2 | -3.6 | -1.1 | - | -2.7 |
| ENSG00000148180.16 | GSN | -3.8 | - | - | -3.1 |
| ENSG00000148339.12 | SLC25A25 | -2.0 | - | -2.2 | -2.1 |
| ENSG00000148346.11 | LCN2 | 3.3 | -1.7 | 1.6 | - |
| ENSG00000148400.9 | NOTCH1 | - | -1.2 | -1.4 | -2.1 |
| ENSG00000148411.7 | -CC2 | -2.1 | - | -1.2 | -1.9 |
| ENSG00000148468.16 | FAM171A1 | -1.4 | - | -1.0 | -2.0 |
| ENSG00000148516.21 | ZEB1 | -3.1 | - | - | -1.3 |
| ENSG00000148541.12 | FAM13C | -2.4 | - | -1.5 | -2.0 |
| ENSG00000148735.14 | PLEKHS1 | - | 1.4 | 2.6 | -1.2 |
| ENSG00000148773.12 | MKI67 | 2.5 | - | 1.7 | 2.4 |
| ENSG00000148824.18 | MTG1 | 1.8 | 1.4 | - | 1.3 |
| ENSG00000148841.15 | ITPRIP | -2.2 | - | -1.9 | -1.5 |
| ENSG00000149090.11 | PAMR1 | -3.9 | - | - | -3.0 |
| ENSG00000149131.15 | SERPING1 | -3.3 | - | -1.4 | -1.5 |
| ENSG00000149218.4 | ENDOD1 | -1.8 | - | 1.0 | -1.5 |
| ENSG00000149256.14 | TENM4 | - | - | 1.9 | -1.9 |
| ENSG00000149260.14 | CAPN5 | - | - | 1.3 | -1.4 |
| ENSG00000149269.9 | PAK1 | - | - | 1.0 | 1.5 |
| ENSG00000149294.16 | NCAM1 | -6.6 | - | -1.2 | -3.2 |
| ENSG00000149380.11 | P4HA3 | - | - | 1.6 | 1.7 |
| ENSG00000149451.17 | ADAM33 | -6.5 | - | -2.2 | -1.6 |
| ENSG00000149554.12 | CHEK1 | 1.5 | - | 1.3 | - |
| ENSG00000149557.12 | FEZ1 | -1.7 | - | -1.8 | - |
| ENSG00000149564.11 | ESAM | - | - | -2.3 | -1.7 |
| ENSG00000149573.8 | MPZL2 | - | -1.3 | 1.5 | - |
| ENSG00000149591.16 | TAGLN | -6.3 | - | - | -1.2 |
| ENSG00000149596.6 | JPH2 | -4.8 | -1.2 | -1.5 | -2.9 |

| | | | | | |
|---|---|---|---|---|---|
| ENSG00000149599.15 | DUSP15 | - | -1.0 | - | 1.1 |
| ENSG00000149798.4 | CDC42EP2 | -1.5 | - | -2.3 | -1.9 |
| ENSG00000149823.7 | VPS51 | -1.2 | - | - | -1.3 |
| ENSG00000149922.10 | TBX6 | 1.8 | - | 1.9 | - |
| ENSG00000150048.10 | CLEC1A | -2.7 | - | -1.9 | - |
| ENSG00000150281.6 | CTF1 | - | -1.7 | - | -1.3 |
| ENSG00000150457.8 | LATS2 | -2.4 | - | -1.3 | -1.9 |
| ENSG00000150477.14 | KIAA1328 | - | 1.5 | - | 1.7 |
| ENSG00000150510.15 | FAM124A | - | -1.3 | -2.0 | -1.2 |
| ENSG00000150551.10 | LYPD1 | 3.3 | - | 2.5 | - |
| ENSG00000150594.6 | ADRA2A | -3.9 | - | - | -2.7 |
| ENSG00000150630.3 | VEGFC | -1.4 | - | -1.3 | -1.3 |
| ENSG00000150636.15 | CCDC102B | -1.5 | - | -1.4 | - |
| ENSG00000150764.13 | DIXDC1 | -3.8 | - | - | -1.7 |
| ENSG00000150907.6 | FOXO1 | - | - | -1.0 | -2.5 |
| ENSG00000150938.9 | CRIM1 | -1.0 | - | -1.4 | -2.8 |
| ENSG00000150995.17 | ITPR1 | -2.0 | - | - | -1.5 |
| ENSG00000151338.18 | MIPOL1 | - | 1.5 | - | 1.5 |
| ENSG00000151364.15 | KCTD14 | - | - | 1.3 | -1.7 |
| ENSG00000151388.10 | ADAMTS12 | - | - | 1.1 | -1.1 |
| ENSG00000151414.14 | NEK7 | -1.0 | - | - | -1.7 |
| ENSG00000151468.10 | CCDC3 | -2.0 | - | - | -3.3 |
| ENSG00000151491.12 | EPS8 | - | - | 1.2 | -2.2 |
| ENSG00000151623.14 | NR3C2 | -2.2 | - | - | -2.9 |
| ENSG00000151689.12 | INPP1 | - | - | -1.0 | -1.4 |
| ENSG00000151702.16 | FLI1 | -2.1 | - | -1.6 | -1.6 |
| ENSG00000151718.15 | WWC2 | - | - | -1.8 | -1.0 |
| ENSG00000151725.11 | CENPU | 2.1 | - | 1.4 | 2.4 |
| ENSG00000151835.13 | SACS | -2.8 | - | - | -2.0 |
| ENSG00000152049.6 | KCNE4 | -2.5 | - | - | 1.2 |
| ENSG00000152102.17 | FAM168B | -1.1 | - | - | -1.7 |
| ENSG00000152137.6 | HSPB8 | -4.1 | -1.2 | -2.0 | - |
| ENSG00000152217.16 | SETBP1 | -1.8 | - | -1.0 | - |
| ENSG00000152284.4 | TCF7L1 | -2.6 | - | -1.1 | -1.4 |
| ENSG00000152484.13 | USP12 | -1.4 | - | - | -1.2 |
| ENSG00000152518.6 | ZFP36L2 | - | -1.0 | -1.1 | -1.6 |

| | | | | | |
|---|---|---|---|---|---|
| ENSG00000152527.13 | PLEKHH2 | -1.7 | - | -1.6 | - |
| ENSG00000152580.8 | IGSF10 | -4.8 | - | -2.6 | -3.1 |
| ENSG00000152582.12 | SPEF2 | -1.9 | - | - | 1.1 |
| ENSG00000152583.12 | SPARCL1 | -4.2 | - | -1.8 | -1.6 |
| ENSG00000152601.17 | MBNL1 | -1.4 | - | - | -1.7 |
| ENSG00000152642.10 | GPD1L | - | -1.0 | - | -1.5 |
| ENSG00000152669.8 | CCNO | - | - | 2.9 | 1.9 |
| ENSG00000152784.15 | PRDM8 | -3.4 | -2.8 | - | - |
| ENSG00000152818.18 | UTRN | -1.7 | - | - | -1.9 |
| ENSG00000152926.14 | ZNF117 | 1.7 | - | 1.6 | - |
| ENSG00000152952.11 | PLOD2 | - | - | 1.3 | -1.7 |
| ENSG00000153071.14 | DAB2 | -1.9 | - | - | -1.7 |
| ENSG00000153162.8 | BMP6 | - | - | -2.2 | -3.5 |
| ENSG00000153179.11 | RASSF3 | -2.5 | - | - | -1.8 |
| ENSG00000153234.13 | NR4A2 | -2.5 | - | -2.9 | - |
| ENSG00000153246.11 | PLA2R1 | 1.4 | - | - | -1.2 |
| ENSG00000153291.15 | SLC25A27 | -1.9 | 1.4 | - | - |
| ENSG00000153446.15 | C16orf89 | -6.1 | -1.3 | 1.4 | -1.3 |
| ENSG00000153814.11 | JAZF1 | -3.0 | - | - | -1.6 |
| ENSG00000153823.18 | PID1 | -5.0 | - | -1.8 | -3.3 |
| ENSG00000153902.13 | LGI4 | -3.7 | - | -2.0 | -2.3 |
| ENSG00000153956.15 | CAC-2D1 | -3.3 | - | - | -2.3 |
| ENSG00000154027.18 | AK5 | - | 1.1 | - | -2.0 |
| ENSG00000154065.16 | ANKRD29 | -5.2 | - | -2.7 | -1.3 |
| ENSG00000154133.14 | ROBO4 | -1.8 | - | -3.4 | -2.4 |
| ENSG00000154175.16 | ABI3BP | -4.7 | - | -2.2 | -2.0 |
| ENSG00000154188.9 | ANGPT1 | - | - | -1.8 | -2.5 |
| ENSG00000154258.16 | ABCA9 | - | - | -1.6 | -1.1 |
| ENSG00000154262.12 | ABCA6 | - | 1.1 | -1.2 | -1.4 |
| ENSG00000154263.17 | ABCA10 | - | 1.1 | -1.4 | -1.4 |
| ENSG00000154330.12 | PGM5 | -7.2 | -1.1 | -1.7 | -2.6 |
| ENSG00000154359.12 | LONRF1 | -1.3 | - | -1.1 | -1.4 |
| ENSG00000154511.11 | FAM69A | -1.8 | - | - | -1.7 |
| ENSG00000154529.14 | CNT-P3B | - | - | -1.6 | -1.6 |
| ENSG00000154553.13 | PDLIM3 | -6.0 | - | -1.0 | -1.2 |
| ENSG00000154678.16 | PDE1C | -3.3 | - | -2.6 | - |

| | | | | | |
|---|---|---|---|---|---|
| ENSG00000154721.14 | JAM2 | -4.1 | - | -2.1 | -1.2 |
| ENSG00000154734.14 | ADAMTS1 | -4.3 | - | -2.4 | -2.8 |
| ENSG00000154783.10 | FGD5 | - | - | -2.1 | -1.9 |
| ENSG00000154813.9 | DPH3 | -1.0 | - | - | -1.4 |
| ENSG00000154822.15 | PLCL2 | -2.2 | - | - | -2.8 |
| ENSG00000154856.12 | APCDD1 | -1.5 | - | - | -2.3 |
| ENSG00000154920.14 | EME1 | - | - | 1.4 | 2.3 |
| ENSG00000155016.17 | CYP2U1 | -1.7 | - | - | -2.3 |
| ENSG00000155066.15 | PROM2 | - | -1.0 | 3.3 | - |
| ENSG00000155090.14 | KLF10 | -1.7 | -1.1 | -1.4 | -2.0 |
| ENSG00000155099.7 | TMEM55A | -1.8 | - | - | -1.7 |
| ENSG00000155254.12 | MARVELD1 | -1.7 | -1.0 | - | -1.5 |
| ENSG00000155324.9 | GRAMD3 | - | - | 1.5 | -1.4 |
| ENSG00000155380.11 | SLC16A1 | -1.8 | - | - | -2.0 |
| ENSG00000155659.14 | VSIG4 | -3.6 | - | -1.6 | -2.0 |
| ENSG00000155760.2 | FZD7 | -2.9 | - | - | -2.4 |
| ENSG00000155761.13 | SPAG17 | 2.8 | - | - | 1.9 |
| ENSG00000155846.16 | PPARGC1B | - | - | -1.0 | -1.5 |
| ENSG00000155849.15 | ELMO1 | -2.1 | - | -1.3 | - |
| ENSG00000155850.7 | SLC26A2 | - | - | -1.1 | -1.4 |
| ENSG00000155876.5 | RRAGA | -1.1 | - | - | -1.0 |
| ENSG00000155962.12 | CLIC2 | -1.9 | - | - | -1.6 |
| ENSG00000156076.9 | WIF1 | - | -1.7 | -3.2 | - |
| ENSG00000156218.12 | ADAMTSL3 | - | - | -1.8 | 1.7 |
| ENSG00000156298.12 | TSPAN7 | -3.4 | - | -2.5 | -1.8 |
| ENSG00000156802.12 | ATAD2 | - | - | 1.5 | 1.0 |
| ENSG00000156804.7 | FBXO32 | -2.3 | - | 2.3 | -1.4 |
| ENSG00000156876.9 | SASS6 | 1.1 | - | - | 1.2 |
| ENSG00000156970.12 | BUB1B | 2.8 | - | 1.7 | 1.7 |
| ENSG00000157150.4 | TIMP4 | - | -1.7 | - | -4.7 |
| ENSG00000157240.3 | FZD1 | -1.9 | -1.2 | - | -2.0 |
| ENSG00000157368.10 | IL34 | - | - | -1.5 | -1.8 |
| ENSG00000157388.13 | CAC-1D | - | 1.9 | -1.1 | 1.5 |
| ENSG00000157456.7 | CCNB2 | 1.6 | - | 1.5 | - |
| ENSG00000157510.13 | AFAP1L1 | - | - | -2.1 | -2.2 |
| ENSG00000157514.16 | TSC22D3 | -2.6 | -1.2 | - | - |

| | | | | | |
|---|---|---|---|---|---|
| ENSG00000157554.18 | ERG | -2.4 | 2.1 | -1.9 | -2.2 |
| ENSG00000157557.11 | ETS2 | - | -1.3 | -1.2 | -1.3 |
| ENSG00000157570.11 | TSPAN18 | - | - | -2.2 | -1.9 |
| ENSG00000157613.10 | CREB3L1 | -2.2 | - | - | 1.5 |
| ENSG00000157734.13 | SNX22 | - | 1.5 | -1.9 | - |
| ENSG00000157827.19 | FMNL2 | -1.0 | - | - | -2.0 |
| ENSG00000157927.16 | RADIL | - | - | -3.3 | -1.1 |
| ENSG00000157933.9 | SKI | -1.2 | - | - | -1.9 |
| ENSG00000158050.4 | DUSP2 | - | -1.1 | -2.2 | - |
| ENSG00000158089.14 | GALNT14 | 3.1 | -1.3 | 2.1 | - |
| ENSG00000158106.12 | RHPN1 | 2.4 | 1.1 | 2.1 | 2.6 |
| ENSG00000158125.9 | XDH | 2.7 | - | 4.7 | - |
| ENSG00000158186.12 | MRAS | -2.7 | - | -1.1 | -3.3 |
| ENSG00000158246.7 | FAM46B | -3.3 | -1.2 | -1.9 | - |
| ENSG00000158292.6 | GPR153 | - | - | -1.5 | -1.3 |
| ENSG00000158301.18 | GPRASP2 | -1.6 | - | - | -2.2 |
| ENSG00000158352.15 | SHROOM4 | - | - | -1.7 | -1.4 |
| ENSG00000158373.8 | HIST1H2BD | - | - | 2.3 | 2.0 |
| ENSG00000158458.19 | NRG2 | - | -1.4 | - | -3.1 |
| ENSG00000158517.13 | NCF1 | -2.7 | - | -1.3 | - |
| ENSG00000158555.14 | GDPD5 | -2.0 | - | - | -1.8 |
| ENSG00000158747.13 | NBL1 | -1.8 | -1.1 | - | - |
| ENSG00000158792.15 | SPATA2L | -1.1 | - | -1.1 | - |
| ENSG00000158887.15 | MPZ | -2.6 | -1.2 | - | -3.0 |
| ENSG00000158966.13 | CACHD1 | -1.1 | - | - | -2.3 |
| ENSG00000159079.18 | C21orf59 | 1.0 | - | - | 1.1 |
| ENSG00000159167.11 | STC1 | -2.7 | - | -1.1 | - |
| ENSG00000159176.13 | CSRP1 | -4.0 | - | -1.0 | -1.3 |
| ENSG00000159200.17 | RCAN1 | -3.1 | - | -1.5 | -1.7 |
| ENSG00000159214.12 | CCDC24 | 1.3 | - | - | 1.7 |
| ENSG00000159403.15 | C1R | -3.7 | - | - | -1.4 |
| ENSG00000159433.11 | STARD9 | -3.1 | - | -2.0 | - |
| ENSG00000159674.11 | SPON2 | -2.9 | 1.9 | - | - |
| ENSG00000159713.10 | TPPP3 | - | - | -1.3 | -1.2 |
| ENSG00000159840.15 | ZYX | -1.6 | - | -1.1 | - |
| ENSG00000159884.11 | CCDC107 | -2.8 | - | - | -1.0 |

| | | | | | |
|---|---|---|---|---|---|
| ENSG00000159915.12 | ZNF233 | - | 1.2 | - | 1.1 |
| ENSG00000160013.8 | PTGIR | -1.9 | - | -1.7 | - |
| ENSG00000160111.12 | CPAMD8 | - | -1.6 | - | -1.9 |
| ENSG00000160180.15 | TFF3 | - | 1.7 | 1.8 | 1.6 |
| ENSG00000160182.2 | TFF1 | - | -2.3 | - | 2.4 |
| ENSG00000160183.13 | TMPRSS3 | - | - | 1.3 | 1.2 |
| ENSG00000160233.7 | LRRC3 | - | - | -1.2 | -1.5 |
| ENSG00000160255.16 | ITGB2 | -2.1 | - | - | -1.0 |
| ENSG00000160284.14 | SPATC1L | - | -1.1 | - | 1.1 |
| ENSG00000160298.17 | C21orf58 | 1.8 | - | - | 1.7 |
| ENSG00000160307.9 | S100B | -3.4 | - | 1.8 | -3.8 |
| ENSG00000160352.15 | ZNF714 | 1.6 | - | - | 1.0 |
| ENSG00000160360.11 | GPSM1 | -1.1 | - | -1.6 | - |
| ENSG00000160408.14 | ST6GAL-C6 | -1.9 | - | - | -2.0 |
| ENSG00000160539.4 | PPAPDC3 | -4.2 | - | - | -2.1 |
| ENSG00000160593.17 | AMICA1 | -2.2 | - | - | -1.1 |
| ENSG00000160606.10 | TLCD1 | - | - | 1.3 | 1.5 |
| ENSG00000160712.12 | IL6R | -1.7 | - | - | -1.5 |
| ENSG00000160781.15 | PAQR6 | - | 2.5 | - | 2.6 |
| ENSG00000160789.19 | LM- | -1.2 | -1.1 | -1.2 | - |
| ENSG00000160801.13 | PTH1R | -3.6 | - | -2.4 | -2.3 |
| ENSG00000160867.14 | FGFR4 | - | - | -3.2 | 1.7 |
| ENSG00000160888.6 | IER2 | -1.5 | - | -1.1 | - |
| ENSG00000160949.16 | TONSL | 1.9 | - | 1.3 | 1.3 |
| ENSG00000160957.12 | RECQL4 | 1.8 | -1.5 | 1.7 | 2.3 |
| ENSG00000160972.9 | PPP1R16A | 1.0 | - | - | 1.4 |
| ENSG00000160973.7 | FOXH1 | 2.8 | - | 2.4 | 1.9 |
| ENSG00000161055.3 | SCGB3A1 | - | -2.3 | 4.1 | -1.5 |
| ENSG00000161281.10 | COX7A1 | -4.5 | - | -1.8 | -1.4 |
| ENSG00000161544.9 | CYGB | -2.5 | - | -1.2 | -2.2 |
| ENSG00000161638.10 | ITGA5 | -4.2 | - | -1.4 | - |
| ENSG00000161647.18 | MPP3 | - | - | -1.1 | 1.1 |
| ENSG00000161649.12 | CD300LG | - | - | -4.6 | -3.8 |
| ENSG00000161798.6 | AQP5 | - | - | 2.3 | -2.3 |
| ENSG00000161835.10 | GRASP | -3.9 | - | -2.9 | -1.1 |
| ENSG00000161888.11 | SPC24 | 2.1 | - | 1.2 | - |

| | | | | | |
|---|---|---|---|---|---|
| ENSG00000161940.10 | BCL6B | -2.3 | - | -2.4 | -2.0 |
| ENSG00000161958.10 | FGF11 | 2.7 | - | 2.3 | - |
| ENSG00000162062.14 | C16orf59 | 2.4 | - | 2.3 | 2.5 |
| ENSG00000162063.12 | CCNF | 1.6 | - | 1.2 | - |
| ENSG00000162069.14 | CCDC64B | 1.3 | - | 1.5 | 1.5 |
| ENSG00000162407.8 | PPAP2B | -3.2 | - | -1.5 | -3.0 |
| ENSG00000162433.14 | AK4 | - | - | 1.7 | -2.0 |
| ENSG00000162444.11 | RBP7 | -4.0 | - | - | -2.2 |
| ENSG00000162493.16 | PDPN | -3.1 | -1.4 | - | - |
| ENSG00000162512.15 | SDC3 | -1.5 | - | - | -1.5 |
| ENSG00000162520.14 | SYNC | -4.4 | - | -1.4 | -1.3 |
| ENSG00000162551.13 | ALPL | - | -1.2 | - | -2.4 |
| ENSG00000162599.15 | NFIA | -1.5 | - | - | -1.0 |
| ENSG00000162614.18 | NEXN | -4.0 | - | -1.2 | - |
| ENSG00000162616.8 | D-JB4 | -2.4 | - | -1.2 | -1.4 |
| ENSG00000162618.12 | ADGRL4 | -2.2 | - | - | -2.3 |
| ENSG00000162692.10 | VCAM1 | -3.2 | - | - | -1.9 |
| ENSG00000162706.12 | CADM3 | -9.0 | - | -1.2 | -1.8 |
| ENSG00000162733.16 | DDR2 | -4.4 | - | - | -3.3 |
| ENSG00000162747.9 | FCGR3B | -3.6 | - | -2.5 | - |
| ENSG00000162772.16 | ATF3 | -2.1 | - | -2.3 | - |
| ENSG00000162804.13 | SNED1 | -3.2 | - | - | -1.1 |
| ENSG00000162817.6 | C1orf115 | - | - | -1.9 | -1.6 |
| ENSG00000162836.11 | ACP6 | 1.6 | - | 1.1 | - |
| ENSG00000162849.15 | KIF26B | - | - | 3.2 | 1.4 |
| ENSG00000162878.12 | PKDCC | -5.8 | - | -1.5 | -2.8 |
| ENSG00000162882.14 | HAAO | -3.0 | -1.2 | - | - |
| ENSG00000162896.5 | PIGR | 1.9 | - | 2.1 | -4.0 |
| ENSG00000162998.4 | FRZB | -1.3 | - | - | -1.4 |
| ENSG00000163017.13 | ACTG2 | -7.6 | - | -1.5 | -2.4 |
| ENSG00000163069.12 | SGCB | -2.2 | - | -1.0 | -1.1 |
| ENSG00000163071.10 | SPATA18 | - | -1.2 | - | -1.3 |
| ENSG00000163083.5 | INHBB | -1.9 | - | - | -1.7 |
| ENSG00000163132.6 | MSX1 | -2.9 | - | -1.6 | -1.3 |
| ENSG00000163141.18 | BNIPL | - | -1.9 | - | 1.3 |
| ENSG00000163145.12 | C1QTNF7 | -4.7 | - | -1.7 | - |

| | | | | | |
|---|---|---|---|---|---|
| ENSG00000163171.7 | CDC42EP3 | -2.4 | - | - | -1.3 |
| ENSG00000163220.10 | S100A9 | - | -1.3 | - | -2.3 |
| ENSG00000163235.15 | TGFA | - | - | 1.3 | -2.0 |
| ENSG00000163239.12 | TDRD10 | - | - | -1.3 | -1.2 |
| ENSG00000163297.16 | ANTXR2 | -2.7 | - | - | -2.6 |
| ENSG00000163362.10 | C1orf106 | 1.7 | -2.0 | 2.0 | - |
| ENSG00000163378.13 | EOGT | - | - | -1.1 | -1.5 |
| ENSG00000163412.12 | EIF4E3 | -1.2 | - | - | -1.9 |
| ENSG00000163430.9 | FSTL1 | -2.7 | - | - | -2.2 |
| ENSG00000163431.12 | LMOD1 | -6.3 | - | -1.3 | -2.1 |
| ENSG00000163462.17 | TRIM46 | - | - | 1.3 | 1.3 |
| ENSG00000163463.11 | KRTCAP2 | 1.7 | 1.0 | - | - |
| ENSG00000163512.13 | AZI2 | -1.1 | - | - | -1.4 |
| ENSG00000163513.17 | TGFBR2 | -1.4 | - | -1.3 | -3.3 |
| ENSG00000163520.13 | FBLN2 | -5.7 | - | - | -2.4 |
| ENSG00000163531.15 | NFASC | -3.3 | - | -1.7 | -1.0 |
| ENSG00000163563.7 | MNDA | -3.0 | - | -1.2 | - |
| ENSG00000163637.11 | PRICKLE2 | -2.3 | - | -1.3 | -1.6 |
| ENSG00000163638.13 | ADAMTS9 | -2.7 | - | -1.7 | -2.1 |
| ENSG00000163659.12 | TIPARP | -1.9 | - | - | -1.4 |
| ENSG00000163686.13 | ABHD6 | - | - | -1.6 | -2.1 |
| ENSG00000163687.13 | D-SE1L3 | -4.0 | - | -2.5 | -2.1 |
| ENSG00000163710.7 | PCOLCE2 | -6.9 | - | -1.6 | -3.8 |
| ENSG00000163762.6 | TM4SF18 | -1.5 | - | - | -1.6 |
| ENSG00000163788.13 | SNRK | - | - | -1.4 | -1.3 |
| ENSG00000163794.6 | UCN | - | 1.7 | - | 1.8 |
| ENSG00000163814.7 | CDCP1 | 1.7 | - | 1.0 | - |
| ENSG00000163815.5 | CLEC3B | -6.3 | -1.3 | -3.7 | -3.1 |
| ENSG00000163820.14 | FYCO1 | -1.3 | - | - | -1.7 |
| ENSG00000163884.3 | KLF15 | - | - | -1.8 | -3.8 |
| ENSG00000163909.7 | HEYL | -1.9 | - | -1.4 | - |
| ENSG00000163993.6 | S100P | - | -2.0 | 2.5 | 2.6 |
| ENSG00000164023.14 | SGMS2 | -1.8 | - | - | -1.8 |
| ENSG00000164035.9 | EMCN | -3.2 | - | -2.4 | -1.5 |
| ENSG00000164038.14 | SLC9B2 | -2.3 | - | - | -1.1 |
| ENSG00000164056.10 | SPRY1 | -2.3 | - | - | -2.1 |

| | | | | | |
|---|---|---|---|---|---|
| ENSG00000164078.12 | MST1R | 2.7 | - | - | 1.4 |
| ENSG00000164086.9 | DUSP7 | - | - | -1.1 | -1.3 |
| ENSG00000164096.12 | C4orf3 | -1.3 | - | - | -1.5 |
| ENSG00000164111.14 | ANXA5 | -2.0 | - | - | -1.0 |
| ENSG00000164116.16 | GUCY1A3 | -2.0 | - | - | -1.9 |
| ENSG00000164125.15 | FAM198B | -1.8 | - | - | -1.7 |
| ENSG00000164221.12 | CCDC112 | - | 1.0 | - | 1.1 |
| ENSG00000164241.13 | C5orf63 | 1.3 | 1.0 | - | - |
| ENSG00000164294.13 | GPX8 | -1.7 | - | 1.1 | - |
| ENSG00000164330.16 | EBF1 | -3.1 | - | - | -3.0 |
| ENSG00000164442.9 | CITED2 | -1.4 | - | - | -1.6 |
| ENSG00000164530.13 | PI16 | -10.4 | - | -2.1 | -3.0 |
| ENSG00000164611.12 | PTTG1 | - | - | 1.1 | 2.6 |
| ENSG00000164619.8 | BMPER | - | - | -2.7 | -2.1 |
| ENSG00000164620.8 | RELL2 | 1.6 | - | - | 1.2 |
| ENSG00000164626.8 | KCNK5 | - | - | 2.2 | -1.1 |
| ENSG00000164647.8 | STEAP1 | - | - | 1.4 | -1.1 |
| ENSG00000164649.19 | CDCA7L | - | -1.2 | - | -1.2 |
| ENSG00000164659.14 | KIAA1324L | - | - | -1.7 | -1.4 |
| ENSG00000164687.10 | FABP5 | - | 2.2 | - | -1.9 |
| ENSG00000164691.16 | TAGAP | -2.3 | - | -1.1 | -1.2 |
| ENSG00000164736.5 | SOX17 | -4.7 | - | -3.2 | -2.6 |
| ENSG00000164741.14 | DLC1 | -2.3 | - | -2.0 | -2.7 |
| ENSG00000164764.10 | SBSPON | -3.3 | - | -2.2 | - |
| ENSG00000164849.8 | GPR146 | - | - | -2.2 | -2.9 |
| ENSG00000164850.14 | GPER1 | -3.0 | -2.1 | -3.0 | -1.6 |
| ENSG00000164855.15 | TMEM184A | - | - | 1.8 | 1.7 |
| ENSG00000164867.10 | NOS3 | -2.3 | - | -1.1 | -1.7 |
| ENSG00000164929.16 | BAALC | - | - | -2.8 | -2.9 |
| ENSG00000164932.12 | CTHRC1 | -2.6 | - | 3.2 | 1.7 |
| ENSG00000164946.19 | FREM1 | -1.6 | - | - | -3.2 |
| ENSG00000164949.7 | GEM | -4.6 | - | -1.3 | - |
| ENSG00000165030.3 | NFIL3 | -3.2 | - | -1.8 | -1.5 |
| ENSG00000165072.9 | MAMDC2 | -3.7 | - | -2.3 | -2.5 |
| ENSG00000165092.12 | ALDH1A1 | -3.5 | - | - | -2.8 |
| ENSG00000165124.17 | SVEP1 | -4.2 | - | -1.8 | -3.7 |

| | | | | | |
|---|---|---|---|---|---|
| ENSG00000165152.8 | TMEM246 | - | -2.3 | - | -2.4 |
| ENSG00000165168.7 | CYBB | -2.2 | - | - | -1.8 |
| ENSG00000165197.4 | FIGF | - | - | -3.0 | -4.9 |
| ENSG00000165215.6 | CLDN3 | - | - | 1.8 | 1.1 |
| ENSG00000165238.16 | WNK2 | - | - | 2.0 | -2.1 |
| ENSG00000165304.7 | MELK | 2.3 | - | 2.1 | - |
| ENSG00000165312.6 | OTUD1 | - | - | -1.6 | -1.7 |
| ENSG00000165410.14 | CFL2 | -4.1 | - | -1.2 | - |
| ENSG00000165424.6 | ZCCHC24 | -3.2 | - | - | -2.4 |
| ENSG00000165457.13 | FOLR2 | -3.1 | - | - | -2.0 |
| ENSG00000165474.5 | GJB2 | 2.4 | - | 3.1 | 3.1 |
| ENSG00000165507.8 | C10orf10 | -1.5 | - | -1.9 | -2.4 |
| ENSG00000165548.10 | TMEM63C | - | - | 3.9 | 1.3 |
| ENSG00000165633.12 | VSTM4 | -2.3 | - | -1.4 | - |
| ENSG00000165716.9 | FAM69B | -1.3 | - | -1.5 | - |
| ENSG00000165757.8 | KIAA1462 | -2.6 | - | -2.2 | -2.2 |
| ENSG00000165795.20 | NDRG2 | - | -1.3 | -1.4 | -2.3 |
| ENSG00000165804.15 | ZNF219 | - | -1.1 | -1.0 | -1.3 |
| ENSG00000165810.16 | BTNL9 | - | - | -3.8 | -3.0 |
| ENSG00000165868.12 | HSPA12A | -3.5 | - | - | -1.9 |
| ENSG00000165914.14 | TTC7B | -1.1 | - | -1.0 | - |
| ENSG00000165923.15 | AGBL2 | 1.6 | - | - | 1.2 |
| ENSG00000165996.13 | HACD1 | -2.4 | - | -2.1 | - |
| ENSG00000166025.17 | AMOTL1 | -2.3 | - | -1.3 | -2.6 |
| ENSG00000166073.8 | GPR176 | -2.1 | -1.3 | - | -1.8 |
| ENSG00000166086.12 | JAM3 | -3.9 | - | -1.3 | -1.5 |
| ENSG00000166106.3 | ADAMTS15 | - | - | -2.0 | -1.3 |
| ENSG00000166123.13 | GPT2 | 1.8 | - | 2.3 | -1.3 |
| ENSG00000166128.12 | RAB8B | -1.1 | - | - | -1.7 |
| ENSG00000166147.13 | FBN1 | -3.6 | - | - | -1.8 |
| ENSG00000166165.12 | CKB | -3.7 | - | -1.0 | - |
| ENSG00000166173.10 | LARP6 | -1.6 | - | -1.0 | -1.8 |
| ENSG00000166250.11 | CLMP | -3.8 | - | - | -3.0 |
| ENSG00000166265.11 | CYYR1 | -1.9 | - | -1.9 | -2.3 |
| ENSG00000166292.11 | TMEM100 | -4.4 | - | -4.8 | - |
| ENSG00000166311.9 | SMPD1 | -1.5 | - | - | -1.1 |

| | | | | | |
|---|---|---|---|---|---|
| ENSG00000166313.18 | APBB1 | -3.1 | - | -1.3 | -1.3 |
| ENSG00000166341.7 | DCHS1 | -3.2 | - | -1.5 | -1.7 |
| ENSG00000166377.19 | ATP9B | 1.0 | 1.3 | - | - |
| ENSG00000166402.8 | TUB | -2.9 | - | - | -1.7 |
| ENSG00000166432.14 | ZMAT1 | - | 1.3 | - | 1.0 |
| ENSG00000166482.11 | MFAP4 | -6.2 | - | -2.5 | -1.6 |
| ENSG00000166510.13 | CCDC68 | - | -1.1 | -1.1 | - |
| ENSG00000166523.7 | CLEC4E | -3.6 | - | -2.3 | - |
| ENSG00000166579.15 | NDEL1 | -1.2 | - | -1.1 | -1.1 |
| ENSG00000166592.11 | RRAD | -2.9 | - | - | -1.2 |
| ENSG00000166819.11 | PLIN1 | - | 1.5 | - | -4.4 |
| ENSG00000166825.13 | ANPEP | -2.4 | - | - | -1.7 |
| ENSG00000166828.2 | SCNN1G | 2.0 | -2.3 | - | - |
| ENSG00000166831.8 | RBPMS2 | -5.1 | - | -1.2 | -2.5 |
| ENSG00000166851.14 | PLK1 | 2.0 | - | 1.4 | 1.3 |
| ENSG00000166927.12 | MS4A7 | -1.8 | - | - | 1.4 |
| ENSG00000166963.12 | MAP1A | -4.0 | - | - | -1.3 |
| ENSG00000166974.12 | MAPRE2 | -2.2 | - | - | -1.4 |
| ENSG00000166979.12 | EVA1C | -2.0 | -1.0 | - | - |
| ENSG00000167074.14 | TEF | - | - | -1.1 | -2.4 |
| ENSG00000167100.14 | SAMD14 | -1.6 | - | -1.6 | - |
| ENSG00000167191.11 | GPRC5B | -1.4 | -1.5 | - | -2.7 |
| ENSG00000167371.16 | PRRT2 | -3.7 | - | - | 2.3 |
| ENSG00000167434.9 | CA4 | - | - | -5.2 | -5.1 |
| ENSG00000167513.8 | CDT1 | 1.9 | - | 1.2 | 1.1 |
| ENSG00000167535.7 | CACNB3 | 1.3 | - | - | 1.4 |
| ENSG00000167549.18 | CORO6 | -4.2 | - | -1.4 | 1.7 |
| ENSG00000167588.12 | GPD1 | - | - | -2.1 | -4.5 |
| ENSG00000167601.11 | AXL | -2.8 | - | - | -1.6 |
| ENSG00000167608.11 | TMC4 | 1.4 | - | 1.4 | - |
| ENSG00000167641.10 | PPP1R14A | -4.8 | - | -2.3 | -2.3 |
| ENSG00000167644.11 | C19orf33 | 2.4 | - | - | 2.3 |
| ENSG00000167702.11 | KIFC2 | 1.3 | 1.0 | - | 2.1 |
| ENSG00000167778.8 | SPRYD3 | -1.1 | - | - | -1.0 |
| ENSG00000167779.7 | IGFBP6 | -5.3 | - | -1.7 | -2.6 |
| ENSG00000167799.9 | NUDT8 | 1.7 | 2.0 | - | - |

| | | | | | |
|---|---|---|---|---|---|
| ENSG00000167874.6 | TMEM88 | -1.9 | - | -3.2 | - |
| ENSG00000167880.7 | EVPL | 1.4 | - | - | 1.6 |
| ENSG00000167900.11 | TK1 | 1.8 | - | 1.5 | 2.2 |
| ENSG00000167964.12 | RAB26 | 1.8 | 1.1 | 2.2 | 3.0 |
| ENSG00000167968.12 | D-SE1L2 | 1.5 | - | - | 2.4 |
| ENSG00000167971.15 | CASKIN1 | - | 1.1 | 3.2 | 1.2 |
| ENSG00000167972.13 | ABCA3 | -2.3 | - | -2.0 | - |
| ENSG00000167994.11 | RAB3IL1 | -3.5 | - | - | -1.0 |
| ENSG00000167995.15 | BEST1 | -2.9 | - | -1.2 | - |
| ENSG00000168032.8 | ENTPD3 | - | -1.3 | 1.2 | - |
| ENSG00000168060.14 | -ALADL1 | -2.7 | - | -1.1 | -1.3 |
| ENSG00000168077.13 | SCARA3 | -1.9 | - | - | -1.1 |
| ENSG00000168079.16 | SCARA5 | -5.8 | - | -2.5 | -5.0 |
| ENSG00000168140.4 | VASN | -3.4 | - | - | -1.2 |
| ENSG00000168309.16 | FAM107A | -6.1 | -1.1 | -4.1 | -3.0 |
| ENSG00000168329.13 | CX3CR1 | - | - | -1.6 | -1.8 |
| ENSG00000168350.7 | DEGS2 | - | - | 1.7 | 1.8 |
| ENSG00000168386.18 | FILIP1L | -4.2 | - | -1.1 | -1.3 |
| ENSG00000168447.10 | SCNN1B | - | -2.2 | - | -1.2 |
| ENSG00000168461.12 | RAB31 | -1.4 | - | - | 1.4 |
| ENSG00000168477.17 | TNXB | -6.0 | - | -2.9 | -2.9 |
| ENSG00000168490.13 | PHYHIP | -2.7 | - | - | -1.9 |
| ENSG00000168491.9 | CCDC110 | - | 1.5 | - | 1.1 |
| ENSG00000168497.4 | SDPR | -4.5 | - | -3.2 | -3.5 |
| ENSG00000168528.11 | SERINC2 | 1.5 | - | 1.8 | 1.1 |
| ENSG00000168542.12 | COL3A1 | -2.0 | - | 1.6 | - |
| ENSG00000168685.14 | IL7R | -2.5 | - | -1.6 | - |
| ENSG00000168734.13 | PKIG | -3.7 | - | -1.7 | - |
| ENSG00000168743.12 | NPNT | - | - | -2.2 | 1.3 |
| ENSG00000168874.12 | ATOH8 | - | -1.4 | -2.5 | -3.3 |
| ENSG00000168899.4 | VAMP5 | -1.6 | - | -1.3 | -1.0 |
| ENSG00000168907.13 | PLA2G4F | - | - | -2.2 | 1.1 |
| ENSG00000168952.15 | STXBP6 | -4.2 | - | -3.8 | - |
| ENSG00000168994.13 | PXDC1 | -1.8 | -1.1 | -1.5 | -1.6 |
| ENSG00000169047.5 | IRS1 | - | -1.3 | - | -2.1 |
| ENSG00000169071.14 | ROR2 | -4.9 | -1.5 | - | - |

| | | | | | |
|---|---|---|---|---|---|
| ENSG00000169116.11 | PARM1 | -2.0 | -1.3 | - | -2.8 |
| ENSG00000169122.11 | FAM110B | -3.4 | - | -1.1 | - |
| ENSG00000169129.14 | AFAP1L2 | - | -1.0 | - | -1.2 |
| ENSG00000169252.5 | ADRB2 | - | - | -2.3 | -2.9 |
| ENSG00000169255.13 | B3GALNT1 | 1.6 | - | -1.6 | - |
| ENSG00000169282.17 | KC-B1 | -2.7 | - | - | -2.3 |
| ENSG00000169291.9 | SHE | -2.7 | - | -1.4 | -1.2 |
| ENSG00000169302.14 | STK32A | 2.1 | - | 2.4 | - |
| ENSG00000169418.9 | NPR1 | -3.6 | - | -2.3 | -3.8 |
| ENSG00000169439.11 | SDC2 | -2.5 | - | - | -1.8 |
| ENSG00000169442.8 | CD52 | -2.6 | - | -1.3 | - |
| ENSG00000169504.14 | CLIC4 | -3.4 | - | - | -1.9 |
| ENSG00000169508.6 | GPR183 | -3.0 | - | -1.0 | - |
| ENSG00000169515.5 | CCDC8 | -3.5 | -1.1 | - | -1.6 |
| ENSG00000169554.16 | ZEB2 | -3.3 | - | - | -2.4 |
| ENSG00000169583.12 | CLIC3 | - | -2.2 | -2.6 | - |
| ENSG00000169679.14 | BUB1 | 2.3 | - | 1.8 | - |
| ENSG00000169715.14 | MT1E | -2.6 | - | -1.5 | - |
| ENSG00000169744.12 | LDB2 | -2.7 | - | -2.5 | -2.4 |
| ENSG00000169764.14 | UGP2 | -1.2 | - | - | -2.1 |
| ENSG00000169851.15 | PCDH7 | -2.2 | -1.3 | 1.3 | - |
| ENSG00000169855.19 | ROBO1 | - | -1.0 | - | -1.9 |
| ENSG00000169894.17 | MUC3A | 2.7 | - | 5.4 | - |
| ENSG00000169902.13 | TPST1 | -2.0 | - | - | -1.3 |
| ENSG00000169926.9 | KLF13 | - | - | -1.4 | -2.0 |
| ENSG00000170049.9 | KC-B3 | - | 1.0 | - | 1.0 |
| ENSG00000170145.4 | SIK2 | - | - | -1.0 | -2.5 |
| ENSG00000170153.10 | RNF150 | -3.0 | - | - | -3.1 |
| ENSG00000170190.15 | SLC16A5 | - | -2.0 | -1.1 | - |
| ENSG00000170214.3 | ADRA1B | - | - | -1.6 | -1.7 |
| ENSG00000170222.11 | ADPRM | -1.0 | - | - | -1.0 |
| ENSG00000170271.10 | FAXDC2 | -2.1 | - | -1.3 | -1.7 |
| ENSG00000170275.14 | CRTAP | -1.9 | - | - | -1.8 |
| ENSG00000170312.15 | CDK1 | 1.8 | - | 1.4 | 2.1 |
| ENSG00000170323.8 | FABP4 | - | - | -2.7 | -4.0 |
| ENSG00000170345.9 | FOS | -1.8 | - | -1.8 | - |

| | | | | | |
|---|---|---|---|---|---|
| ENSG00000170369.3 | CST2 | - | - | 2.4 | 4.0 |
| ENSG00000170373.8 | CST1 | 3.4 | -1.6 | 3.5 | - |
| ENSG00000170417.14 | TMEM182 | 1.2 | 1.8 | - | 1.1 |
| ENSG00000170421.11 | KRT8 | - | - | 1.0 | 1.7 |
| ENSG00000170458.13 | CD14 | -2.6 | - | - | -1.4 |
| ENSG00000170464.9 | D-JC18 | -1.7 | - | -1.1 | - |
| ENSG00000170469.10 | SPATA24 | - | - | 1.2 | 1.6 |
| ENSG00000170476.15 | MZB1 | - | - | 1.8 | 1.5 |
| ENSG00000170525.18 | PFKFB3 | - | - | -1.0 | -3.2 |
| ENSG00000170571.11 | EMB | - | - | 1.2 | -1.0 |
| ENSG00000170891.10 | CYTL1 | -5.1 | -1.3 | - | - |
| ENSG00000170903.10 | MSANTD4 | - | - | 1.2 | -1.1 |
| ENSG00000170962.12 | PDGFD | -2.9 | - | - | -2.1 |
| ENSG00000170989.8 | S1PR1 | -3.0 | - | -2.9 | -2.9 |
| ENSG00000171051.8 | FPR1 | -3.7 | - | -2.1 | - |
| ENSG00000171056.7 | SOX7 | -2.1 | -1.9 | -3.6 | -1.6 |
| ENSG00000171115.3 | GIMAP8 | - | - | -2.2 | -2.4 |
| ENSG00000171121.16 | KCNMB3 | - | 1.0 | 1.4 | 1.3 |
| ENSG00000171124.12 | FUT3 | 2.8 | - | 2.0 | - |
| ENSG00000171132.13 | PRKCE | - | - | -2.0 | -1.0 |
| ENSG00000171163.15 | ZNF692 | 1.3 | 1.1 | - | 1.8 |
| ENSG00000171223.5 | JUNB | -1.8 | - | -2.2 | - |
| ENSG00000171303.6 | KCNK3 | - | - | -3.7 | -1.1 |
| ENSG00000171345.13 | KRT19 | - | -2.0 | - | 1.3 |
| ENSG00000171346.13 | KRT15 | - | -2.4 | 3.5 | -1.6 |
| ENSG00000171368.11 | TPPP | -2.1 | - | -1.8 | -2.2 |
| ENSG00000171388.11 | APLN | - | - | -2.7 | -1.2 |
| ENSG00000171522.5 | PTGER4 | - | - | -1.7 | -2.7 |
| ENSG00000171533.11 | MAP6 | -2.3 | - | - | -1.1 |
| ENSG00000171596.6 | NMUR1 | - | - | -2.9 | -1.7 |
| ENSG00000171608.15 | PIK3CD | -1.5 | - | - | -1.2 |
| ENSG00000171621.13 | SPSB1 | -1.6 | -1.0 | - | - |
| ENSG00000171631.14 | P2RY6 | - | - | 2.9 | 1.1 |
| ENSG00000171659.13 | GPR34 | -2.2 | - | - | -2.1 |
| ENSG00000171766.15 | GATM | -2.0 | - | 1.3 | - |
| ENSG00000171777.15 | RASGRP4 | -2.5 | - | -1.7 | - |

| | | | | | |
|---|---|---|---|---|---|
| ENSG00000171791.11 | BCL2 | -1.8 | - | - | -1.1 |
| ENSG00000171812.10 | COL8A2 | -3.8 | - | 1.4 | - |
| ENSG00000171848.13 | RRM2 | 2.2 | - | 1.6 | - |
| ENSG00000171860.4 | C3AR1 | -2.3 | - | - | -1.0 |
| ENSG00000171867.16 | PRNP | -1.7 | - | - | -2.4 |
| ENSG00000171992.12 | SYNPO | -2.2 | - | -1.2 | -2.8 |
| ENSG00000172005.10 | MAL | - | -1.0 | -2.0 | - |
| ENSG00000172037.13 | LAMB2 | -1.8 | - | -1.1 | -1.1 |
| ENSG00000172059.10 | KLF11 | -1.7 | - | -1.0 | -2.8 |
| ENSG00000172137.18 | CALB2 | - | - | -1.7 | -3.6 |
| ENSG00000172159.15 | FRMD3 | - | - | -2.3 | -2.5 |
| ENSG00000172201.10 | ID4 | -1.2 | -1.6 | -2.0 | -1.9 |
| ENSG00000172216.5 | CEBPB | -1.7 | -1.2 | -1.4 | -1.1 |
| ENSG00000172236.16 | TPSAB1 | -5.3 | - | - | 1.1 |
| ENSG00000172260.13 | NEGR1 | -4.7 | - | -1.1 | - |
| ENSG00000172346.14 | CSDC2 | -6.1 | - | - | 1.4 |
| ENSG00000172348.14 | RCAN2 | -4.4 | - | - | -1.7 |
| ENSG00000172349.16 | IL16 | -3.0 | - | - | -1.4 |
| ENSG00000172403.10 | SYNPO2 | -5.7 | - | -1.1 | -2.4 |
| ENSG00000172458.4 | IL17D | -2.2 | - | -1.5 | - |
| ENSG00000172594.12 | SMPDL3A | -2.0 | - | - | -1.1 |
| ENSG00000172602.9 | RND1 | - | - | -1.7 | 1.4 |
| ENSG00000172638.12 | EFEMP2 | -3.0 | - | - | -1.0 |
| ENSG00000172724.11 | CCL19 | -3.8 | - | - | 1.4 |
| ENSG00000172889.15 | EGFL7 | -2.0 | - | -2.7 | -1.1 |
| ENSG00000172932.14 | ANKRD13D | 1.1 | - | - | 1.3 |
| ENSG00000172935.8 | MRGPRF | -5.5 | -1.1 | -1.6 | -2.3 |
| ENSG00000172985.10 | SH3RF3 | -2.5 | - | -1.0 | -1.7 |
| ENSG00000172987.12 | HPSE2 | -8.2 | - | -1.1 | -2.9 |
| ENSG00000173137.11 | ADCK5 | - | - | 1.2 | 1.4 |
| ENSG00000173175.14 | ADCY5 | -4.8 | - | - | -2.2 |
| ENSG00000173198.5 | CYSLTR1 | 1.2 | 1.5 | - | - |
| ENSG00000173210.19 | ABLIM3 | - | - | -2.0 | -2.4 |
| ENSG00000173212.4 | MAB21L3 | - | 1.2 | - | 1.8 |
| ENSG00000173267.13 | SNCG | - | -1.4 | - | -1.7 |
| ENSG00000173269.13 | MMRN2 | - | - | -2.1 | -2.5 |

| | | | | | |
|---|---|---|---|---|---|
| ENSG00000173276.13 | ZBTB21 | -1.8 | - | - | -1.9 |
| ENSG00000173369.15 | C1QB | -2.5 | - | -1.1 | - |
| ENSG00000173376.13 | NDNF | -4.2 | -1.1 | -1.4 | - |
| ENSG00000173391.8 | OLR1 | - | - | -1.5 | 1.4 |
| ENSG00000173457.10 | PPP1R14B | - | - | 1.3 | 1.4 |
| ENSG00000173482.16 | PTPRM | - | - | -1.8 | -2.1 |
| ENSG00000173511.9 | VEGFB | -1.7 | - | - | -1.4 |
| ENSG00000173530.5 | TNFRSF10D | -1.4 | - | -1.5 | -2.1 |
| ENSG00000173546.7 | CSPG4 | -2.3 | - | -1.9 | -2.5 |
| ENSG00000173599.13 | PC | - | - | 1.8 | -2.8 |
| ENSG00000173641.17 | HSPB7 | -7.1 | - | -1.3 | -4.5 |
| ENSG00000173706.12 | HEG1 | -2.1 | - | -1.8 | -1.8 |
| ENSG00000173715.15 | C11orf80 | - | - | 1.4 | 1.0 |
| ENSG00000173918.14 | C1QTNF1 | -2.7 | - | - | -2.0 |
| ENSG00000174004.5 | NRROS | -2.5 | - | - | -1.6 |
| ENSG00000174059.16 | CD34 | -2.9 | - | -1.9 | -1.7 |
| ENSG00000174080.10 | CTSF | -2.3 | - | - | -1.1 |
| ENSG00000174099.10 | MSRB3 | -4.8 | - | -1.3 | -1.3 |
| ENSG00000174125.7 | TLR1 | -2.0 | - | - | -1.2 |
| ENSG00000174175.16 | SELP | -3.8 | - | -1.8 | -2.2 |
| ENSG00000174282.11 | ZBTB4 | -1.2 | - | - | -2.0 |
| ENSG00000174306.21 | ZHX3 | -1.6 | - | - | -1.1 |
| ENSG00000174501.14 | ANKRD36C | - | 1.5 | - | 1.1 |
| ENSG00000174516.14 | PELI3 | -1.4 | - | - | -1.0 |
| ENSG00000174567.7 | GOLT1A | 1.6 | - | - | 1.6 |
| ENSG00000174600.13 | CMKLR1 | -2.4 | - | - | -1.6 |
| ENSG00000174640.12 | SLCO2A1 | -1.7 | - | -2.3 | - |
| ENSG00000174684.6 | B4GAT1 | -1.6 | - | -1.1 | - |
| ENSG00000174788.9 | PCP2 | 1.5 | - | - | 2.3 |
| ENSG00000174804.3 | FZD4 | -1.3 | - | -1.8 | -2.6 |
| ENSG00000174807.3 | CD248 | -3.2 | - | - | -2.4 |
| ENSG00000174944.8 | P2RY14 | -3.9 | - | -1.8 | - |
| ENSG00000174945.13 | AMZ1 | - | 1.6 | - | 1.8 |
| ENSG00000175040.5 | CHST2 | - | -1.6 | - | -2.9 |
| ENSG00000175063.16 | UBE2C | 2.3 | 1.2 | 2.0 | 3.4 |
| ENSG00000175084.11 | DES | -9.8 | -1.0 | -2.6 | -1.7 |

| | | | | | |
|---|---|---|---|---|---|
| ENSG00000175164.13 | ABO | 1.6 | -1.7 | - | - |
| ENSG00000175183.9 | CSRP2 | -1.3 | -1.1 | - | - |
| ENSG00000175279.21 | APITD1 | 1.8 | - | - | 1.0 |
| ENSG00000175395.15 | ZNF25 | -1.5 | - | - | -1.3 |
| ENSG00000175445.14 | LPL | - | - | -1.9 | -4.8 |
| ENSG00000175455.14 | CCDC14 | 1.0 | 1.1 | - | - |
| ENSG00000175592.8 | FOSL1 | - | -1.2 | -3.1 | - |
| ENSG00000175600.15 | SUGCT | -2.1 | - | 2.3 | - |
| ENSG00000175602.3 | CCDC85B | -2.0 | - | -1.3 | - |
| ENSG00000175643.8 | RMI2 | 1.6 | - | 1.9 | 1.5 |
| ENSG00000175745.11 | NR2F1 | -3.2 | - | -1.2 | - |
| ENSG00000175764.14 | TTLL11 | -1.2 | - | - | -1.5 |
| ENSG00000175785.12 | PRIMA1 | -4.1 | -1.7 | - | -2.0 |
| ENSG00000175832.12 | ETV4 | 3.0 | -1.0 | 3.1 | - |
| ENSG00000175899.14 | A2M | -3.0 | - | -1.6 | -2.2 |
| ENSG00000176014.12 | TUBB6 | -3.8 | - | -2.0 | -2.0 |
| ENSG00000176124.11 | DLEU1 | 1.9 | - | 1.1 | - |
| ENSG00000176208.8 | ATAD5 | 1.6 | - | - | 1.1 |
| ENSG00000176387.6 | HSD11B2 | - | -1.9 | 1.4 | 1.3 |
| ENSG00000176435.6 | CLEC14A | -1.8 | - | -2.7 | -1.7 |
| ENSG00000176438.12 | SYNE3 | - | - | -1.4 | -2.7 |
| ENSG00000176532.3 | PRR15 | - | -1.5 | - | 1.8 |
| ENSG00000176533.12 | GNG7 | -3.2 | - | - | -1.4 |
| ENSG00000176597.11 | B3GNT5 | - | - | -1.1 | -1.5 |
| ENSG00000176842.14 | IRX5 | - | -1.2 | - | 1.1 |
| ENSG00000176845.12 | METRNL | -1.8 | - | -1.3 | - |
| ENSG00000176903.4 | PNMA1 | -2.8 | -1.1 | - | -1.3 |
| ENSG00000176920.11 | FUT2 | - | -1.6 | 3.4 | - |
| ENSG00000176971.3 | FIBIN | -4.8 | - | -2.0 | - |
| ENSG00000177054.13 | ZDHHC13 | 1.1 | - | 1.3 | - |
| ENSG00000177098.8 | SCN4B | - | - | -3.0 | -3.1 |
| ENSG00000177202.2 | SPACA4 | - | - | 1.3 | 2.4 |
| ENSG00000177283.5 | FZD8 | - | - | -1.1 | -1.5 |
| ENSG00000177374.12 | HIC1 | -3.6 | - | -1.6 | -1.6 |
| ENSG00000177432.6 | -P1L5 | -1.9 | - | -1.1 | -1.2 |
| ENSG00000177464.4 | GPR4 | - | - | -2.0 | -1.6 |

| | | | | | |
|---|---|---|---|---|---|
| ENSG00000177469.12 | PTRF | -4.3 | -1.1 | -2.0 | -2.5 |
| ENSG00000177606.6 | JUN | -2.5 | - | -1.0 | - |
| ENSG00000177694.14 | -ALADL2 | - | 1.3 | 1.4 | - |
| ENSG00000177706.8 | FAM20C | -3.2 | - | - | -1.2 |
| ENSG00000177990.11 | DPY19L2 | -2.4 | 1.4 | - | - |
| ENSG00000178031.15 | ADAMTSL1 | -4.7 | - | - | -1.6 |
| ENSG00000178175.11 | ZNF366 | - | - | -1.5 | -1.4 |
| ENSG00000178222.12 | RNF212 | -2.6 | - | 1.8 | - |
| ENSG00000178401.14 | D-JC22 | - | - | 2.9 | 1.1 |
| ENSG00000178573.6 | MAF | -2.2 | - | - | -2.1 |
| ENSG00000178695.5 | KCTD12 | -2.5 | - | - | -2.8 |
| ENSG00000178726.6 | THBD | -2.2 | - | -2.8 | -2.0 |
| ENSG00000178773.14 | CPNE7 | - | 2.9 | 2.3 | 3.0 |
| ENSG00000178826.10 | TMEM139 | - | -1.8 | -1.9 | - |
| ENSG00000178852.15 | EFCAB13 | - | 1.3 | 1.2 | - |
| ENSG00000178878.12 | APOLD1 | -3.2 | - | -2.3 | -2.7 |
| ENSG00000178935.5 | ZNF552 | 1.6 | - | 1.0 | 1.8 |
| ENSG00000178999.12 | AURKB | 2.4 | - | 1.3 | 2.4 |
| ENSG00000179023.8 | KLHDC7A | 1.9 | - | 2.1 | - |
| ENSG00000179094.13 | PER1 | -2.9 | - | -1.6 | -1.6 |
| ENSG00000179144.4 | GIMAP7 | -2.3 | - | -1.5 | -1.3 |
| ENSG00000179314.13 | WSCD1 | - | - | -1.7 | -1.8 |
| ENSG00000179388.8 | EGR3 | -4.2 | - | -2.3 | -2.0 |
| ENSG00000179528.15 | LBX2 | 1.8 | - | 1.4 | 1.2 |
| ENSG00000179604.9 | CDC42EP4 | - | -1.1 | - | -2.3 |
| ENSG00000179776.17 | CDH5 | -2.0 | - | -3.0 | -2.4 |
| ENSG00000179820.15 | MYADM | -3.8 | - | -2.2 | -1.3 |
| ENSG00000179855.5 | GIPC3 | - | - | -1.9 | -1.5 |
| ENSG00000179862.6 | CITED4 | - | -1.4 | -1.2 | 1.0 |
| ENSG00000179921.14 | GPBAR1 | -2.8 | - | -2.1 | -2.9 |
| ENSG00000179979.8 | CRIPAK | - | 1.2 | - | 1.1 |
| ENSG00000180155.18 | LYNX1 | -2.2 | -1.1 | - | -1.6 |
| ENSG00000180198.15 | RCC1 | 1.3 | - | 1.5 | - |
| ENSG00000180346.3 | TIGD2 | - | - | 1.1 | -1.3 |
| ENSG00000180354.15 | MTURN | -1.8 | - | -1.3 | -2.9 |
| ENSG00000180447.6 | GAS1 | -5.4 | -2.1 | - | -2.0 |

| | | | | | |
|---|---|---|---|---|---|
| ENSG00000180543.4 | TSPYL5 | -1.5 | - | 1.0 | - |
| ENSG00000180644.6 | PRF1 | - | - | -2.3 | -2.1 |
| ENSG00000180879.13 | SSR4 | - | - | 1.3 | 1.0 |
| ENSG00000180884.9 | ZNF792 | 1.1 | - | - | -1.4 |
| ENSG00000180921.6 | FAM83H | 1.8 | - | 1.2 | - |
| ENSG00000180953.11 | ST20 | - | 1.1 | - | 1.3 |
| ENSG00000181085.14 | MAPK15 | 3.0 | - | - | 2.7 |
| ENSG00000181191.11 | PJA1 | - | -1.0 | - | -2.0 |
| ENSG00000181218.5 | HIST3H2A | 1.4 | 1.2 | - | 2.1 |
| ENSG00000181392.14 | SYNE4 | - | - | 1.1 | 1.2 |
| ENSG00000181444.12 | ZNF467 | -1.5 | - | - | 1.5 |
| ENSG00000181449.3 | SOX2 | - | -1.3 | 1.9 | - |
| ENSG00000181458.10 | TMEM45A | - | 1.5 | - | 1.1 |
| ENSG00000181649.5 | PHLDA2 | - | -1.2 | 1.0 | - |
| ENSG00000181754.6 | AMIGO1 | - | - | -1.3 | -1.7 |
| ENSG00000181804.14 | SLC9A9 | -2.6 | - | - | -2.6 |
| ENSG00000181826.9 | RELL1 | - | - | -1.0 | -1.7 |
| ENSG00000181856.14 | SLC2A4 | -5.2 | - | - | -2.0 |
| ENSG00000182013.17 | PNMAL1 | - | -1.1 | 2.2 | - |
| ENSG00000182247.9 | UBE2E2 | -1.6 | - | - | -1.5 |
| ENSG00000182253.14 | SYNM | -7.5 | - | -2.0 | -3.1 |
| ENSG00000182272.11 | B4GALNT4 | - | - | 2.2 | 1.7 |
| ENSG00000182325.10 | FBXL6 | 1.1 | - | - | 1.5 |
| ENSG00000182326.14 | C1S | -4.2 | - | - | -1.5 |
| ENSG00000182472.8 | CAPN12 | 1.8 | - | 1.5 | - |
| ENSG00000182492.15 | BGN | -1.7 | - | - | 2.1 |
| ENSG00000182511.11 | FES | -1.0 | - | - | -1.2 |
| ENSG00000182534.13 | MXRA7 | -3.0 | - | - | -1.3 |
| ENSG00000182575.7 | NXPH3 | -5.5 | - | -2.5 | - |
| ENSG00000182578.13 | CSF1R | -2.3 | - | - | -1.5 |
| ENSG00000182636.5 | NDN | -2.2 | - | - | -2.5 |
| ENSG00000182667.14 | NTM | -1.9 | - | - | -1.0 |
| ENSG00000182685.7 | BRICD5 | - | 1.1 | - | 1.4 |
| ENSG00000182771.17 | GRID1 | - | - | -1.5 | -2.0 |
| ENSG00000182782.7 | HCAR2 | - | - | -1.8 | -3.0 |
| ENSG00000182916.7 | TCEAL7 | -5.5 | - | -1.2 | -1.8 |

| | | | | | |
|---|---|---|---|---|---|
| ENSG00000183010.16 | PYCR1 | - | - | 2.5 | 1.4 |
| ENSG00000183036.10 | PCP4 | -8.4 | - | 3.6 | - |
| ENSG00000183048.11 | SLC25A10 | 2.0 | - | 1.5 | - |
| ENSG00000183087.14 | GAS6 | -4.1 | - | -1.4 | - |
| ENSG00000183111.11 | ARHGEF37 | -1.9 | - | - | -1.5 |
| ENSG00000183248.11 | PRR36 | 2.1 | 1.1 | 1.6 | 1.9 |
| ENSG00000183340.6 | JRKL | - | - | 1.0 | -1.4 |
| ENSG00000183421.11 | RIPK4 | - | -1.2 | - | -1.7 |
| ENSG00000183578.5 | TNFAIP8L3 | -3.8 | - | -1.6 | - |
| ENSG00000183580.9 | FBXL7 | -3.5 | - | -1.3 | -1.1 |
| ENSG00000183688.4 | FAM101B | -1.5 | - | -1.6 | -1.1 |
| ENSG00000183722.7 | LHFP | -3.1 | - | -1.9 | -2.2 |
| ENSG00000183741.11 | CBX6 | -1.3 | - | - | -1.1 |
| ENSG00000183773.15 | AIFM3 | 1.7 | - | - | 1.6 |
| ENSG00000183798.4 | EMILIN3 | -2.7 | -1.3 | - | -4.4 |
| ENSG00000183801.7 | OLFML1 | -3.5 | - | -1.5 | -1.3 |
| ENSG00000183807.7 | FAM162B | -2.0 | - | -2.9 | - |
| ENSG00000183853.17 | KIRREL | -2.0 | - | - | -2.4 |
| ENSG00000183856.10 | IQGAP3 | 3.7 | - | 2.0 | 3.0 |
| ENSG00000183963.18 | SMTN | -3.7 | - | -1.0 | - |
| ENSG00000184005.10 | ST6GAL-C3 | -2.6 | - | -2.0 | - |
| ENSG00000184014.7 | DENND5A | -1.5 | - | - | -1.7 |
| ENSG00000184113.9 | CLDN5 | -4.2 | -1.5 | -3.9 | -2.5 |
| ENSG00000184160.7 | ADRA2C | -2.5 | -1.6 | -1.4 | - |
| ENSG00000184232.8 | OAF | -1.8 | - | - | -1.5 |
| ENSG00000184347.14 | SLIT3 | -2.1 | - | -1.8 | -3.1 |
| ENSG00000184349.12 | EF-5 | - | -1.0 | 1.8 | -1.3 |
| ENSG00000184371.13 | CSF1 | -2.2 | - | - | -1.6 |
| ENSG00000184445.11 | KNTC1 | 1.3 | - | 1.3 | - |
| ENSG00000184481.16 | FOXO4 | -1.0 | - | - | -1.5 |
| ENSG00000184497.12 | TMEM255B | -1.9 | - | -1.5 | - |
| ENSG00000184500.14 | PROS1 | -3.1 | - | -1.3 | -2.4 |
| ENSG00000184557.4 | SOCS3 | -3.0 | -1.1 | -2.2 | -2.2 |
| ENSG00000184588.17 | PDE4B | -3.6 | - | -1.4 | -1.6 |
| ENSG00000184602.5 | SNN | -1.1 | - | - | -2.5 |
| ENSG00000184785.5 | SMIM10 | -2.6 | - | - | -1.2 |

| | | | | | |
|---|---|---|---|---|---|
| ENSG00000184905.8 | TCEAL2 | -5.0 | -1.7 | -2.4 | - |
| ENSG00000184925.11 | LCN12 | - | 1.1 | 1.1 | - |
| ENSG00000185010.13 | F8 | -2.0 | - | -1.1 | -2.4 |
| ENSG00000185022.11 | MAFF | -1.9 | - | -2.7 | -1.3 |
| ENSG00000185052.11 | SLC24A3 | -1.8 | - | - | -1.2 |
| ENSG00000185101.12 | ANO9 | 2.0 | - | - | 1.5 |
| ENSG00000185112.5 | FAM43A | -3.5 | -1.4 | -2.1 | -2.2 |
| ENSG00000185274.11 | WBSCR17 | -7.0 | - | - | -2.4 |
| ENSG00000185298.12 | CCDC137 | 1.2 | - | - | 1.4 |
| ENSG00000185339.8 | TCN2 | -1.5 | - | - | -2.2 |
| ENSG00000185340.15 | GAS2L1 | - | - | -1.5 | -1.1 |
| ENSG00000185386.14 | MAPK11 | -1.7 | - | -1.2 | - |
| ENSG00000185432.11 | METTL7A | - | - | -1.1 | -1.5 |
| ENSG00000185482.7 | STAC3 | - | 1.2 | - | 1.6 |
| ENSG00000185483.11 | ROR1 | -2.7 | - | - | -1.9 |
| ENSG00000185499.16 | MUC1 | - | - | 1.1 | 2.0 |
| ENSG00000185522.8 | LMNTD2 | 1.3 | - | - | 1.8 |
| ENSG00000185565.11 | LSAMP | -2.2 | -1.9 | - | - |
| ENSG00000185567.6 | AH-K2 | -2.9 | -1.0 | 1.5 | - |
| ENSG00000185585.19 | OLFML2A | -1.7 | -1.4 | -1.7 | -2.2 |
| ENSG00000185614.4 | FAM212A | - | -1.9 | -1.6 | - |
| ENSG00000185842.14 | D-H14 | 1.8 | 1.1 | 1.2 | 1.1 |
| ENSG00000185885.15 | IFITM1 | -2.5 | -1.2 | - | 1.0 |
| ENSG00000185920.15 | PTCH1 | -1.5 | - | -1.3 | -2.1 |
| ENSG00000185950.8 | IRS2 | - | - | -1.3 | -2.1 |
| ENSG00000185989.10 | RASA3 | -1.8 | - | - | -1.7 |
| ENSG00000186081.11 | KRT5 | - | -2.5 | 2.2 | -2.1 |
| ENSG00000186185.13 | KIF18B | 3.1 | - | 1.6 | 1.2 |
| ENSG00000186340.14 | THBS2 | -2.3 | - | 2.4 | - |
| ENSG00000186350.9 | RXRA | - | - | -1.1 | -1.1 |
| ENSG00000186377.7 | CYP4X1 | 1.1 | -1.1 | 1.3 | - |
| ENSG00000186469.8 | GNG2 | -2.8 | - | -1.1 | -3.1 |
| ENSG00000186522.14 | SEPT10 | - | - | -1.0 | -1.6 |
| ENSG00000186567.12 | CEACAM19 | - | 1.1 | -1.1 | - |
| ENSG00000186642.15 | PDE2A | -4.1 | - | -1.9 | -3.4 |
| ENSG00000186832.8 | KRT16 | 2.3 | - | - | -1.3 |

| | | | | | |
|---|---|---|---|---|---|
| ENSG00000186847.5 | KRT14 | - | -2.2 | - | -1.8 |
| ENSG00000186994.11 | KANK3 | -2.9 | - | -3.3 | - |
| ENSG00000187017.14 | ESPN | - | - | 2.3 | 2.1 |
| ENSG00000187193.8 | MT1X | -2.5 | - | -2.3 | -1.7 |
| ENSG00000187210.12 | GCNT1 | - | 1.2 | 1.8 | - |
| ENSG00000187260.15 | WDR86 | - | - | 1.2 | -1.4 |
| ENSG00000187479.5 | C11orf96 | -4.8 | - | -2.6 | -1.7 |
| ENSG00000187513.8 | GJA4 | -2.0 | - | -2.1 | -1.5 |
| ENSG00000187583.10 | PLEKHN1 | - | - | 2.3 | 1.7 |
| ENSG00000187634.10 | SAMD11 | -2.8 | - | - | 2.6 |
| ENSG00000187642.9 | PERM1 | 1.8 | - | 2.4 | 2.5 |
| ENSG00000187688.14 | TRPV2 | -1.8 | - | -1.5 | -1.6 |
| ENSG00000187720.14 | THSD4 | -2.1 | -1.5 | -1.3 | - |
| ENSG00000187741.14 | FANCA | 2.1 | 1.0 | - | 1.4 |
| ENSG00000187758.7 | ADH1A | 2.3 | - | -2.7 | - |
| ENSG00000187800.13 | PEAR1 | -1.6 | - | -2.5 | -2.3 |
| ENSG00000187824.8 | TMEM220 | -2.1 | - | - | -1.4 |
| ENSG00000187837.3 | HIST1H1C | - | - | 1.2 | 1.3 |
| ENSG00000187951.10 | ARHGAP11B | 1.9 | - | - | 1.3 |
| ENSG00000187955.11 | COL14A1 | -4.8 | - | - | -2.0 |
| ENSG00000188042.7 | ARL4C | -1.7 | - | - | -1.2 |
| ENSG00000188070.9 | C11orf95 | -1.1 | - | - | -1.7 |
| ENSG00000188215.9 | DCUN1D3 | -1.3 | - | - | -1.2 |
| ENSG00000188257.10 | PLA2G2A | -3.7 | - | -1.5 | -2.2 |
| ENSG00000188322.4 | SBK1 | 2.6 | - | 2.2 | 1.4 |
| ENSG00000188536.12 | HBA2 | -4.1 | - | -3.4 | - |
| ENSG00000188641.12 | DPYD | -1.9 | - | - | -2.0 |
| ENSG00000188643.10 | S100A16 | - | -1.7 | - | 1.1 |
| ENSG00000188677.14 | PARVB | -2.2 | - | -1.2 | - |
| ENSG00000188738.13 | FSIP2 | 1.0 | 1.7 | - | - |
| ENSG00000188783.5 | PRELP | -6.1 | - | -1.6 | -2.3 |
| ENSG00000188818.12 | ZDHHC11 | - | 1.7 | - | 1.2 |
| ENSG00000188878.16 | FBF1 | 1.1 | - | - | 1.7 |
| ENSG00000188921.13 | HACD4 | -1.4 | - | -1.1 | - |
| ENSG00000189057.10 | FAM111B | 1.9 | - | 2.0 | - |
| ENSG00000189058.8 | APOD | -4.1 | - | - | -2.6 |

| | | | | | |
|---|---|---|---|---|---|
| ENSG00000189067.12 | LITAF | -1.0 | - | - | -1.0 |
| ENSG00000189129.13 | PLAC9 | -3.4 | - | -2.8 | -2.6 |
| ENSG00000189143.9 | CLDN4 | - | -1.1 | 1.3 | - |
| ENSG00000189159.15 | HN1 | 1.4 | - | - | 1.9 |
| ENSG00000189184.11 | PCDH18 | -2.5 | - | - | -2.6 |
| ENSG00000189320.8 | FAM180A | - | - | -1.2 | -1.3 |
| ENSG00000189334.8 | S100A14 | - | -2.8 | 1.1 | 2.8 |
| ENSG00000189367.14 | KIAA0408 | -2.8 | - | - | -2.8 |
| ENSG00000189376.11 | C8orf76 | 1.3 | - | 1.1 | - |
| ENSG00000189377.8 | CXCL17 | - | -2.1 | 1.5 | - |
| ENSG00000189409.12 | MMP23B | -4.4 | - | -1.1 | - |
| ENSG00000196118.11 | C16orf93 | - | - | 1.2 | 1.9 |
| ENSG00000196154.11 | S100A4 | - | - | -1.6 | -1.0 |
| ENSG00000196182.10 | STK40 | -1.2 | - | - | -1.3 |
| ENSG00000196188.10 | CTSE | 2.9 | - | 1.3 | - |
| ENSG00000196196.2 | HRCT1 | -2.5 | - | -1.5 | - |
| ENSG00000196263.7 | ZNF471 | - | 1.1 | - | 1.1 |
| ENSG00000196296.13 | ATP2A1 | - | 1.2 | - | 1.4 |
| ENSG00000196329.10 | GIMAP5 | -2.1 | - | -1.4 | -1.8 |
| ENSG00000196418.12 | ZNF124 | 1.8 | 1.3 | - | - |
| ENSG00000196428.12 | TSC22D2 | -1.1 | - | - | -1.1 |
| ENSG00000196507.10 | TCEAL3 | -2.0 | - | - | 1.2 |
| ENSG00000196526.10 | AFAP1 | -1.1 | - | - | -1.1 |
| ENSG00000196549.10 | MME | - | - | -1.9 | -3.8 |
| ENSG00000196557.10 | CAC-1H | -3.0 | - | -1.1 | 1.6 |
| ENSG00000196569.11 | LAMA2 | -3.4 | - | - | -2.2 |
| ENSG00000196586.13 | MYO6 | 1.2 | 1.5 | - | - |
| ENSG00000196611.4 | MMP1 | 4.8 | - | 3.4 | - |
| ENSG00000196616.12 | ADH1B | -5.6 | - | -2.5 | -4.1 |
| ENSG00000196628.13 | TCF4 | -1.6 | - | - | -1.2 |
| ENSG00000196814.14 | MVB12B | - | - | -1.5 | -1.0 |
| ENSG00000196834.11 | POTEI | - | 1.6 | - | 2.9 |
| ENSG00000196843.15 | ARID5A | -3.1 | - | -1.8 | -1.2 |
| ENSG00000196878.12 | LAMB3 | - | -2.5 | - | -2.6 |
| ENSG00000196912.12 | ANKRD36B | - | 1.4 | - | 1.6 |
| ENSG00000196924.14 | FL- | -4.6 | -1.1 | - | - |

| | | | | | |
|---|---|---|---|---|---|
| ENSG00000196972.7 | SMIM10L2B | - | -1.1 | - | -2.1 |
| ENSG00000197019.4 | SERTAD1 | -1.8 | - | -1.8 | - |
| ENSG00000197043.13 | ANXA6 | -4.0 | -1.0 | - | -1.1 |
| ENSG00000197124.11 | ZNF682 | 1.2 | 1.1 | - | - |
| ENSG00000197162.9 | ZNF785 | 1.5 | - | 1.1 | - |
| ENSG00000197165.10 | SULT1A2 | 2.4 | - | - | 1.3 |
| ENSG00000197245.4 | FAM110D | -3.8 | -1.4 | -2.4 | -2.3 |
| ENSG00000197256.10 | KANK2 | -3.0 | - | -1.7 | -1.4 |
| ENSG00000197321.14 | SVIL | -2.5 | - | - | -1.4 |
| ENSG00000197381.15 | ADARB1 | -3.2 | - | -1.9 | -1.2 |
| ENSG00000197405.7 | C5AR1 | -2.8 | - | -2.0 | - |
| ENSG00000197461.13 | PDGFA | -1.1 | - | - | -1.3 |
| ENSG00000197467.13 | COL13A1 | -3.1 | - | -1.2 | - |
| ENSG00000197471.11 | SPN | -1.5 | - | -1.5 | - |
| ENSG00000197576.13 | HOXA4 | - | - | -2.1 | -1.7 |
| ENSG00000197614.10 | MFAP5 | -5.8 | - | -1.8 | -1.7 |
| ENSG00000197629.5 | MPEG1 | -2.8 | - | - | -2.1 |
| ENSG00000197635.9 | DPP4 | -2.2 | - | 1.9 | -2.3 |
| ENSG00000197702.11 | PARVA | -1.7 | - | - | -1.6 |
| ENSG00000197757.7 | HOXC6 | - | 3.2 | - | 1.4 |
| ENSG00000197766.7 | CFD | -6.7 | - | -2.4 | -3.3 |
| ENSG00000197774.12 | EME2 | 1.4 | 1.5 | - | 1.9 |
| ENSG00000197852.9 | FAM212B | - | - | -2.1 | -1.6 |
| ENSG00000197872.11 | FAM49A | -2.2 | - | -1.2 | -2.9 |
| ENSG00000197879.14 | MYO1C | - | - | -1.2 | -1.7 |
| ENSG00000197930.12 | ERO1L | 1.3 | - | 1.7 | - |
| ENSG00000198053.11 | SIRPA | -1.8 | - | - | -2.4 |
| ENSG00000198075.9 | SULT1C4 | -2.5 | - | -1.7 | - |
| ENSG00000198087.7 | CD2AP | 1.3 | - | 1.0 | - |
| ENSG00000198121.13 | LPAR1 | -1.8 | - | - | -1.7 |
| ENSG00000198125.12 | MB | - | - | 3.5 | 1.2 |
| ENSG00000198203.9 | SULT1C2 | 1.7 | - | 2.0 | - |
| ENSG00000198298.12 | ZNF485 | 1.0 | - | 1.2 | - |
| ENSG00000198300.12 | PEG3 | -4.1 | - | - | -1.7 |
| ENSG00000198353.7 | HOXC4 | - | 2.9 | - | 1.6 |
| ENSG00000198355.4 | PIM3 | -1.2 | - | -1.1 | - |

| | | | | | |
|---|---|---|---|---|---|
| ENSG00000198431.15 | TXNRD1 | -2.2 | - | - | -1.2 |
| ENSG00000198513.11 | ATL1 | -1.4 | - | - | -1.3 |
| ENSG00000198517.9 | MAFK | -1.5 | - | -1.4 | - |
| ENSG00000198554.11 | WDHD1 | 1.7 | - | 1.2 | - |
| ENSG00000198598.6 | MMP17 | - | - | 1.0 | 1.0 |
| ENSG00000198624.12 | CCDC69 | -2.9 | - | -1.1 | -3.1 |
| ENSG00000198682.12 | PAPSS2 | - | - | -1.1 | -1.2 |
| ENSG00000198719.8 | DLL1 | -1.9 | - | -1.5 | -1.0 |
| ENSG00000198732.10 | SMOC1 | - | -1.0 | - | -1.6 |
| ENSG00000198743.5 | SLC5A3 | - | - | 1.7 | -1.7 |
| ENSG00000198771.10 | RCSD1 | -2.5 | - | -1.0 | -1.3 |
| ENSG00000198795.10 | ZNF521 | -3.6 | - | - | -1.9 |
| ENSG00000198807.12 | PAX9 | - | - | 2.4 | 1.3 |
| ENSG00000198826.10 | ARHGAP11A | 1.6 | - | 1.1 | - |
| ENSG00000198832.10 | SELM | -3.9 | -1.3 | - | - |
| ENSG00000198844.10 | ARHGEF15 | -2.1 | - | -2.5 | -2.1 |
| ENSG00000198848.12 | CES1 | -7.3 | - | -2.1 | -3.6 |
| ENSG00000198853.11 | RUSC2 | -3.0 | - | -1.2 | -1.1 |
| ENSG00000198873.11 | GRK5 | -2.7 | - | -2.6 | -2.2 |
| ENSG00000198901.13 | PRC1 | 1.5 | - | - | 1.4 |
| ENSG00000198909.7 | MAP3K3 | -1.4 | - | -1.0 | -1.2 |
| ENSG00000198932.12 | GPRASP1 | -3.0 | - | - | -1.4 |
| ENSG00000198947.14 | DMD | -3.4 | - | - | -2.2 |
| ENSG00000198959.11 | TGM2 | -2.7 | - | - | -1.2 |
| ENSG00000198961.9 | PJA2 | -1.7 | - | - | -1.8 |
| ENSG00000203697.11 | CAPN8 | - | - | 2.0 | 1.4 |
| ENSG00000203727.3 | SAMD5 | - | - | -1.5 | -1.6 |
| ENSG00000203772.7 | SPRN | 1.5 | 1.3 | - | - |
| ENSG00000203780.10 | FANK1 | 1.1 | - | - | 1.3 |
| ENSG00000203883.6 | SOX18 | -2.8 | - | -2.0 | -1.6 |
| ENSG00000203896.9 | LIME1 | 1.9 | 1.3 | - | 1.5 |
| ENSG00000204060.5 | FOXO6 | - | - | 2.1 | 1.3 |
| ENSG00000204131.8 | NHSL2 | -1.9 | - | - | -1.4 |
| ENSG00000204161.13 | C10orf128 | -3.0 | - | - | -1.6 |
| ENSG00000204262.11 | COL5A2 | -1.7 | - | 1.6 | - |
| ENSG00000204291.10 | COL15A1 | -2.9 | - | - | -2.5 |

| | | | | | |
|---|---|---|---|---|---|
| ENSG00000204301.6 | NOTCH4 | - | - | -2.5 | -1.6 |
| ENSG00000204381.11 | LAYN | -2.2 | -1.0 | - | -1.1 |
| ENSG00000204385.10 | SLC44A4 | - | - | 1.5 | 2.0 |
| ENSG00000204410.14 | MSH5 | 1.5 | 1.7 | 1.0 | - |
| ENSG00000204514.9 | ZNF814 | 1.5 | 1.1 | - | 1.2 |
| ENSG00000204839.8 | MROH6 | 1.5 | - | 2.9 | 1.5 |
| ENSG00000205090.8 | TMEM240 | -2.8 | - | -1.0 | - |
| ENSG00000205213.13 | LGR4 | - | - | 2.3 | -2.4 |
| ENSG00000205221.12 | VIT | -8.7 | - | - | -2.6 |
| ENSG00000205362.10 | MT1A | -5.3 | - | -3.8 | - |
| ENSG00000205363.5 | C15orf59 | -2.1 | -1.5 | -1.3 | 1.1 |
| ENSG00000205364.3 | MT1M | -4.6 | - | -3.5 | -1.7 |
| ENSG00000205502.3 | C2CD4B | -3.1 | - | -1.9 | - |
| ENSG00000205517.12 | RGL3 | 1.5 | 1.0 | - | 1.1 |
| ENSG00000205560.12 | CPT1B | 3.4 | 1.8 | - | 1.4 |
| ENSG00000205795.4 | CYS1 | -5.2 | - | -1.6 | -1.1 |
| ENSG00000205869.2 | KRTAP5-1 | - | - | 1.7 | 2.7 |
| ENSG00000205978.5 | NYNRIN | - | -1.4 | - | -1.7 |
| ENSG00000206075.13 | SERPINB5 | - | -2.5 | - | -1.3 |
| ENSG00000206172.8 | HBA1 | -3.9 | - | -3.4 | - |
| ENSG00000206530.8 | CFAP44 | - | 1.2 | - | 1.5 |
| ENSG00000206535.7 | LNP1 | -2.5 | - | -1.3 | - |
| ENSG00000206538.7 | VGLL3 | - | - | -1.4 | -2.9 |
| ENSG00000211445.11 | GPX3 | -5.1 | -1.5 | -2.2 | -3.9 |
| ENSG00000211448.11 | DIO2 | - | - | 2.9 | -1.3 |
| ENSG00000213088.9 | ACKR1 | -5.6 | - | -2.0 | -1.8 |
| ENSG00000213145.9 | CRIP1 | - | - | -1.6 | 1.1 |
| ENSG00000213199.7 | ASIC3 | - | 1.9 | - | 2.2 |
| ENSG00000213203.2 | GIMAP1 | -2.0 | - | -1.6 | -1.3 |
| ENSG00000213462.4 | ERV3-1 | 1.5 | - | 1.9 | - |
| ENSG00000213626.11 | LBH | -1.7 | - | -1.1 | -1.4 |
| ENSG00000213639.9 | PPP1CB | -1.0 | - | - | -1.0 |
| ENSG00000213865.7 | C8orf44 | 1.4 | - | 1.0 | - |
| ENSG00000213901.10 | SLC23A3 | 1.9 | 1.5 | - | - |
| ENSG00000213928.8 | IRF9 | 1.1 | 1.0 | - | - |
| ENSG00000213937.3 | CLDN9 | - | - | 2.0 | 2.8 |

| | | | | | |
|---|---|---|---|---|---|
| ENSG00000213949.8 | ITGA1 | -1.8 | - | - | -1.5 |
| ENSG00000214050.7 | FBXO16 | - | - | 2.0 | 1.4 |
| ENSG00000214140.10 | PRCD | -1.6 | - | - | -1.5 |
| ENSG00000214274.9 | ANG | -2.3 | -1.1 | - | - |
| ENSG00000214575.9 | CPEB1 | -5.0 | - | - | -1.1 |
| ENSG00000214944.9 | ARHGEF28 | 1.3 | - | - | -1.2 |
| ENSG00000215915.9 | ATAD3C | - | -1.2 | - | 1.6 |
| ENSG00000216937.11 | CCDC7 | - | 1.3 | - | 1.2 |
| ENSG00000219438.8 | FAM19A5 | -2.6 | -1.1 | - | - |
| ENSG00000219626.8 | FAM228B | - | 1.4 | - | 1.6 |
| ENSG00000220201.7 | ZGLP1 | - | 1.1 | - | 1.7 |
| ENSG00000221869.4 | CEBPD | -3.0 | -1.3 | -1.5 | - |
| ENSG00000221963.5 | APOL6 | 1.1 | - | - | -1.6 |
| ENSG00000221968.8 | FADS3 | -1.8 | - | -1.1 | -1.5 |
| ENSG00000223572.9 | CKMT1A | 2.2 | - | - | 1.3 |
| ENSG00000224420.3 | ADM5 | - | - | -1.7 | 1.6 |
| ENSG00000228594.3 | C1orf233 | 1.9 | 1.5 | - | - |
| ENSG00000228716.6 | DHFR | 1.6 | - | 1.1 | - |
| ENSG00000228727.8 | SAPCD1 | - | 1.8 | 1.4 | 1.4 |
| ENSG00000233608.3 | TWIST2 | -5.7 | - | -2.0 | -2.4 |
| ENSG00000235098.8 | ANKRD65 | - | -1.3 | - | -1.2 |
| ENSG00000235173.6 | HGH1 | 1.4 | - | 1.1 | - |
| ENSG00000236609.3 | ZNF853 | -1.8 | -1.1 | - | -1.5 |
| ENSG00000236699.8 | ARHGEF38 | - | 1.7 | 1.5 | - |
| ENSG00000237440.8 | ZNF737 | 1.6 | 1.1 | - | - |
| ENSG00000237649.7 | KIFC1 | 2.1 | - | - | 1.6 |
| ENSG00000239264.8 | TXNDC5 | - | - | 1.1 | -1.3 |
| ENSG00000239672.7 | NME1 | 1.4 | - | 1.1 | 2.2 |
| ENSG00000239697.10 | TNFSF12 | -3.3 | - | -1.0 | -1.0 |
| ENSG00000240184.6 | PCDHGC3 | -1.6 | - | -1.5 | -2.1 |
| ENSG00000240583.10 | AQP1 | -3.2 | - | -2.0 | -2.4 |
| ENSG00000241343.9 | RPL36A | - | 1.1 | - | 1.2 |
| ENSG00000241370.5 | RPP21 | - | 1.0 | - | 1.3 |
| ENSG00000241399.6 | CD302 | -1.5 | - | - | -1.3 |
| ENSG00000241484.9 | ARHGAP8 | 2.4 | 1.7 | - | - |
| ENSG00000241489.7 | IDS | - | 1.1 | - | 1.1 |

| | | | | | |
|---|---|---|---|---|---|
| ENSG00000241839.9 | PLEKHO2 | -2.2 | - | -1.1 | -1.8 |
| ENSG00000241978.9 | AKAP2 | -3.1 | - | -1.8 | -2.6 |
| ENSG00000242265.5 | PEG10 | -2.3 | - | -1.2 | - |
| ENSG00000242612.6 | DECR2 | 1.1 | - | 1.3 | 1.3 |
| ENSG00000242732.4 | RGAG4 | -1.7 | - | - | -1.4 |
| ENSG00000243244.5 | STON1 | -2.2 | - | - | -1.9 |
| ENSG00000243364.7 | EF-4 | 1.3 | - | 1.7 | - |
| ENSG00000243449.6 | C4orf48 | 2.8 | - | 1.1 | - |
| ENSG00000243678.11 | NME1-NME2 | 1.7 | - | - | 1.1 |
| ENSG00000243989.7 | ACY1 | 2.0 | - | - | 1.2 |
| ENSG00000244405.7 | ETV5 | - | -1.0 | - | -2.6 |
| ENSG00000244509.3 | APOBEC3C | - | -1.7 | - | -1.1 |
| ENSG00000244617.2 | ASPRV1 | - | - | -1.6 | -1.3 |
| ENSG00000244734.3 | HBB | -2.9 | - | -3.0 | -4.0 |
| ENSG00000248485.1 | PCP4L1 | - | -2.8 | 1.5 | - |
| ENSG00000249242.7 | TMEM150C | -1.9 | - | 1.3 | - |
| ENSG00000249437.7 | -IP | - | 1.5 | - | 1.1 |
| ENSG00000249709.7 | ZNF564 | 1.2 | 1.0 | - | - |
| ENSG00000249751.3 | ECSCR | -2.1 | - | -2.0 | - |
| ENSG00000250067.11 | YJEFN3 | - | 1.6 | - | 1.6 |
| ENSG00000250588.6 | IQCJ-SCHIP1 | -2.5 | -1.1 | -1.6 | -2.0 |
| ENSG00000250722.5 | SEPP1 | -2.6 | - | - | -2.3 |
| ENSG00000253250.2 | C8orf88 | -3.4 | - | -1.4 | - |
| ENSG00000253276.2 | CCDC71L | -1.7 | - | - | -1.9 |
| ENSG00000253304.1 | TMEM200B | -4.7 | - | - | -1.9 |
| ENSG00000254122.2 | PCDHGB7 | - | - | -1.6 | -2.7 |
| ENSG00000254726.2 | MEX3A | 3.7 | - | 2.2 | 1.3 |
| ENSG00000254996.5 | [illegible]NKHD1-EIF4EBP | 1.0 | 1.2 | - | - |
| ENSG00000256043.2 | CTSO | -1.1 | - | - | -1.2 |
| ENSG00000256235.1 | SMIM3 | -2.3 | - | - | -2.2 |
| ENSG00000257594.3 | GALNT4 | - | - | 1.2 | -1.1 |
| ENSG00000258472.8 | RP11-192H23.4 | 1.8 | 1.1 | - | - |
| ENSG00000258947.6 | TUBB3 | 2.5 | - | 1.4 | 2.1 |
| ENSG00000259207.7 | ITGB3 | -2.4 | - | - | -1.9 |
| ENSG00000261052.5 | SULT1A3 | 1.8 | 1.2 | - | 1.6 |
| ENSG00000261371.5 | PECAM1 | -2.4 | - | -2.0 | -2.1 |

| | | | | | |
|---|---|---|---|---|---|
| ENSG00000262655.3 | SPON1 | -3.9 | - | - | -2.0 |
| ENSG00000263155.5 | MYZAP | -1.8 | - | -3.5 | -3.1 |
| ENSG00000265107.2 | GJA5 | -2.3 | - | -1.2 | -1.3 |
| ENSG00000266094.6 | RASSF5 | - | -1.1 | - | -1.2 |
| ENSG00000266524.2 | GDF10 | - | - | -3.4 | -3.4 |
| ENSG00000266964.5 | FXYD1 | -4.3 | - | -2.7 | -2.3 |
| ENSG00000267206.5 | LCN6 | -5.2 | - | -1.9 | - |
| ENSG00000267795.5 | SMIM22 | - | - | 2.4 | 2.3 |
| ENSG00000269190.5 | FBXO17 | -3.2 | -1.1 | - | -1.2 |
| ENSG00000272636.3 | DOC2B | - | - | -1.5 | -1.8 |
| ENSG00000273045.5 | C2orf15 | 1.3 | - | - | 1.0 |
| ENSG00000275302.1 | CCL4 | -2.6 | - | -1.5 | - |
| ENSG00000275395.4 | FCGBP | - | - | 2.7 | -2.8 |
| ENSG00000275832.4 | ARHGAP23 | - | -1.3 | -1.3 | -1.8 |
| ENSG00000276043.4 | UHRF1 | 2.4 | - | 1.5 | 2.0 |
| ENSG00000276600.4 | RAB7B | - | -1.2 | - | -2.0 |
| ENSG00000277363.4 | SRCIN1 | 2.1 | - | 2.0 | - |
| ENSG00000277494.1 | GPIHBP1 | -4.6 | - | -3.8 | -3.6 |
| ENSG00000280308.1 | FKSG61 | 1.3 | 1.1 | - | - |
| ENSG00000280789.1 | PAGR1 | -2.3 | - | - | 1.4 |
| ENSG00000280987.2 | MATR3 | 1.6 | 1.2 | - | - |
| ENSG00000282608.1 | ADORA3 | -2.3 | - | 1.2 | - |

## Supplementary Table S7: Oncogenes reported by COSMIC

| Gene id | Gene name |
|---|---|
| ENSG00000006468.13 | ETV1 |
| ENSG00000007237.18 | GAS7 |
| ENSG00000018408.14 | WWTR1 |
| ENSG00000037280.15 | FLT4 |
| ENSG00000046889.18 | PREX2 |
| ENSG00000049540.16 | ELN |
| ENSG00000065361.14 | ERBB3 |
| ENSG00000066468.20 | FGFR2 |
| ENSG00000067082.14 | KLF6 |
| ENSG00000068078.17 | FGFR3 |
| ENSG00000070404.9 | FSTL3 |
| ENSG00000073282.12 | TP63 |
| ENSG00000077782.19 | FGFR1 |
| ENSG00000083857.13 | FAT1 |
| ENSG00000097007.17 | ABL1 |
| ENSG00000101096.19 | NFATC2 |
| ENSG00000101213.6 | PTK6 |
| ENSG00000104419.14 | NDRG1 |
| ENSG00000105173.13 | CCNE1 |
| ENSG00000105976.14 | MET |
| ENSG00000106462.10 | EZH2 |
| ENSG00000107485.15 | GATA3 |
| ENSG00000108821.13 | COL1A1 |
| ENSG00000108924.13 | HLF |
| ENSG00000109906.13 | ZBTB16 |
| ENSG00000111252.10 | SH2B3 |
| ENSG00000111275.12 | ALDH2 |
| ENSG00000112531.16 | QKI |
| ENSG00000113594.9 | LIFR |
| ENSG00000113721.13 | PDGFRB |
| ENSG00000116016.13 | EPAS1 |
| ENSG00000118503.14 | TNFAIP3 |
| ENSG00000118971.7 | CCND2 |
| ENSG00000119508.17 | NR4A3 |
| ENSG00000119535.17 | CSF3R |
| ENSG00000119866.20 | BCL11A |
| ENSG00000123473.15 | STIL |
| ENSG00000127329.14 | PTPRB |
| ENSG00000128602.9 | SMO |
| ENSG00000129993.14 | CBFA2T3 |
| ENSG00000130844.16 | ZNF331 |
| ENSG00000132170.19 | PPARG |
| ENSG00000133392.16 | MYH11 |
| ENSG00000134853.11 | PDGFRA |
| ENSG00000135111.14 | TBX3 |
| ENSG00000135363.11 | LMO2 |
| ENSG00000136167.13 | LCP1 |
| ENSG00000136826.14 | KLF4 |
| ENSG00000136997.14 | MYC |
| ENSG00000137193.13 | PIM1 |
| ENSG00000138413.13 | IDH1 |
| ENSG00000139263.11 | LRIG3 |
| ENSG00000140538.16 | NTRK3 |
| ENSG00000142273.10 | CBLC |
| ENSG00000144218.18 | AFF3 |
| ENSG00000144476.5 | ACKR3 |
| ENSG00000146374.13 | RSPO3 |
| ENSG00000147065.16 | MSN |
| ENSG00000147257.13 | GPC3 |
| ENSG00000147862.14 | NFIB |
| ENSG00000148400.9 | NOTCH1 |
| ENSG00000150907.6 | FOXO1 |

| | |
|---|---|
| ENSG00000151702.16 | FLI1 |
| ENSG00000152217.16 | SETBP1 |
| ENSG00000153814.11 | JAZF1 |
| ENSG00000156076.9 | WIF1 |
| ENSG00000156970.12 | BUB1B |
| ENSG00000157388.13 | CAC-1D |
| ENSG00000157554.18 | ERG |
| ENSG00000157613.10 | CREB3L1 |
| ENSG00000160789.19 | LM- |
| ENSG00000160867.14 | FGFR4 |
| ENSG00000160957.12 | RECQL4 |
| ENSG00000162733.16 | DDR2 |
| ENSG00000163513.17 | TGFBR2 |
| ENSG00000164330.16 | EBF1 |
| ENSG00000168685.14 | IL7R |
| ENSG00000171791.11 | BCL2 |
| ENSG00000175643.8 | RMI2 |
| ENSG00000175832.12 | ETV4 |
| ENSG00000177606.6 | JUN |
| ENSG00000178573.6 | MAF |
| ENSG00000179094.13 | PER1 |
| ENSG00000180644.6 | PRF1 |
| ENSG00000181449.3 | SOX2 |
| ENSG00000183722.7 | LHFP |
| ENSG00000184481.16 | FOXO4 |
| ENSG00000185499.16 | MUC1 |
| ENSG00000185920.15 | PTCH1 |
| ENSG00000187741.14 | FANCA |
| ENSG00000198795.10 | ZNF521 |
| ENSG00000244405.7 | ETV5 |

**Supplementary Table S8: Significantly enriched BP terms (P ≤ 0.05) of the 12 modules**

| Module | Term ID | Term name | PValue |
|---|---|---|---|
| brown | GO:0045449 | regulation of transcription | 6.72E-13 |
| brown | GO:0006396 | RNA processing | 6.93E-11 |
| brown | GO:0006259 | DNA metabolic process | 1.08E-10 |
| brown | GO:0006974 | response to DNA damage stimulus | 1.58E-09 |
| brown | GO:0051276 | chromosome organization | 2.09E-09 |
| brown | GO:0008380 | RNA splicing | 4.69E-09 |
| brown | GO:0006281 | DNA repair | 1.67E-08 |
| brown | GO:0016071 | mRNA metabolic process | 4.04E-08 |
| brown | GO:0006397 | mRNA processing | 1.01E-07 |
| brown | GO:0006325 | chromatin organization | 5.96E-07 |
| brown | GO:0016568 | chromatin modification | 9.10E-07 |
| brown | GO:0006260 | DNA replication | 1.50E-05 |
| brown | GO:0006355 | regulation of transcription, DNA-dependent | 1.97E-05 |
| brown | GO:0051252 | regulation of RNA metabolic process | 2.96E-05 |
| brown | GO:0033554 | cellular response to stress | 4.00E-05 |
| brown | GO:0006338 | chromatin remodeling | 4.99E-05 |
| brown | GO:0007049 | cell cycle | 7.51E-05 |
| brown | GO:0006333 | chromatin assembly or disassembly | 1.16E-04 |
| brown | GO:0007017 | microtubule-based process | 1.34E-04 |
| brown | GO:0006310 | DNA recombination | 2.04E-04 |
| brown | GO:0022402 | cell cycle process | 6.66E-04 |
| brown | GO:0000279 | M phase | 1.05E-03 |
| brown | GO:0016584 | nucleosome positioning | 1.06E-03 |
| brown | GO:0044265 | cellular macromolecule catabolic process | 1.20E-03 |
| brown | GO:0006304 | DNA modification | 1.49E-03 |
| brown | GO:0060271 | cilium morphogenesis | 1.49E-03 |
| brown | GO:0022403 | cell cycle phase | 1.56E-03 |
| brown | GO:0006261 | DNA-dependent DNA replication | 1.67E-03 |
| brown | GO:0006305 | DNA alkylation | 1.97E-03 |
| brown | GO:0006306 | DNA methylation | 1.97E-03 |
| brown | GO:0031023 | microtubule organizing center organization | 2.04E-03 |

| | | | |
|---|---|---|---|
| brown | GO:0040029 | regulation of gene expression, epigenetic | 2.84E-03 |
| brown | GO:0009057 | macromolecule catabolic process | 2.95E-03 |
| brown | GO:0010629 | negative regulation of gene expression | 4.25E-03 |
| brown | GO:0010605 | negative regulation of macromolecule metabolic process | 4.33E-03 |
| brown | GO:0000280 | nuclear division | 5.30E-03 |
| brown | GO:0007067 | mitosis | 5.30E-03 |
| brown | GO:0016458 | gene silencing | 5.63E-03 |
| brown | GO:0006323 | DNA packaging | 5.67E-03 |
| brown | GO:0000087 | M phase of mitotic cell cycle | 6.46E-03 |
| brown | GO:0000377 | RNA splicing, via transesterification reactions with bulged adenosine as nucleophile | 6.52E-03 |
| brown | GO:0000375 | RNA splicing, via transesterification reactions | 6.52E-03 |
| brown | GO:0000398 | nuclear mRNA splicing, via spliceosome | 6.52E-03 |
| brown | GO:0051297 | centrosome organization | 7.25E-03 |
| brown | GO:0048285 | organelle fission | 7.97E-03 |
| brown | GO:0048489 | synaptic vesicle transport | 8.27E-03 |
| brown | GO:0031047 | gene silencing by RNA | 8.27E-03 |
| brown | GO:0019941 | modification-dependent protein catabolic process | 9.11E-03 |
| brown | GO:0043632 | modification-dependent macromolecule catabolic process | 9.11E-03 |
| brown | GO:0070647 | protein modification by small protein conjugation or removal | 9.36E-03 |
| brown | GO:0051603 | proteolysis involved in cellular protein catabolic process | 1.05E-02 |
| brown | GO:0044257 | cellular protein catabolic process | 1.10E-02 |
| brown | GO:0051301 | cell division | 1.11E-02 |
| brown | GO:0000226 | microtubule cytoskeleton organization | 1.18E-02 |
| brown | GO:0006298 | mismatch repair | 1.47E-02 |
| brown | GO:0030031 | cell projection assembly | 1.49E-02 |
| brown | GO:0045934 | negative regulation of nucleobase, nucleoside, nucleotide and nucleic acid metabolic process | 1.60E-02 |
| brown | GO:0030163 | protein catabolic process | 1.69E-02 |
| brown | GO:0016567 | protein ubiquitination | 1.71E-02 |
| brown | GO:0000278 | mitotic cell cycle | 1.78E-02 |
| brown | GO:0051172 | negative regulation of nitrogen compound metabolic process | 1.92E-02 |
| brown | GO:0007099 | centriole replication | 2.03E-02 |
| brown | GO:0043087 | regulation of GTPase activity | 2.11E-02 |
| brown | GO:0042384 | cilium assembly | 2.18E-02 |
| brown | GO:0034621 | cellular macromolecular complex subunit organization | 2.20E-02 |

| | | | |
|---|---|---|---|
| brown | GO:0006400 | tRNA modification | 2.22E-02 |
| brown | GO:0016569 | covalent chromatin modification | 2.45E-02 |
| brown | GO:0051253 | negative regulation of RNA metabolic process | 2.52E-02 |
| brown | GO:0006511 | ubiquitin-dependent protein catabolic process | 2.67E-02 |
| brown | GO:0034661 | ncRNA catabolic process | 2.76E-02 |
| brown | GO:0043044 | ATP-dependent chromatin remodeling | 2.76E-02 |
| brown | GO:0032392 | DNA geometric change | 3.06E-02 |
| brown | GO:0032508 | DNA duplex unwinding | 3.06E-02 |
| brown | GO:0032446 | protein modification by small protein conjugation | 3.24E-02 |
| brown | GO:0016481 | negative regulation of transcription | 3.45E-02 |
| brown | GO:0010558 | negative regulation of macromolecule biosynthetic process | 3.52E-02 |
| brown | GO:0007098 | centrosome cycle | 3.53E-02 |
| brown | GO:0006351 | transcription, DNA-dependent | 3.59E-02 |
| brown | GO:0009451 | RNA modification | 3.73E-02 |
| brown | GO:0045892 | negative regulation of transcription, DNA-dependent | 3.74E-02 |
| brown | GO:0006399 | tRNA metabolic process | 3.96E-02 |
| brown | GO:0032774 | RNA biosynthetic process | 4.03E-02 |
| brown | GO:0051298 | centrosome duplication | 4.50E-02 |
| brown | GO:0007020 | microtubule nucleation | 4.50E-02 |
| brown | GO:0031327 | negative regulation of cellular biosynthetic process | 4.57E-02 |
| brown | GO:0016570 | histone modification | 4.73E-02 |
| blue | GO:0006350 | transcription | 1.40E-07 |
| blue | GO:0046578 | regulation of Ras protein signal transduction | 6.53E-06 |
| blue | GO:0051056 | regulation of small GTPase mediated signal transduction | 2.23E-05 |
| blue | GO:0006355 | regulation of transcription, DNA-dependent | 1.85E-04 |
| blue | GO:0046777 | protein amino acid autophosphorylation | 6.19E-04 |
| blue | GO:0006468 | protein amino acid phosphorylation | 1.64E-03 |
| blue | GO:0007242 | intracellular signaling cascade | 4.17E-03 |
| blue | GO:0006325 | chromatin organization | 4.26E-03 |
| blue | GO:0007265 | Ras protein signal transduction | 5.33E-03 |
| blue | GO:0033554 | cellular response to stress | 5.66E-03 |
| blue | GO:0010627 | regulation of protein kinase cascade | 6.90E-03 |
| blue | GO:0008104 | protein localization | 8.54E-03 |
| blue | GO:0045184 | establishment of protein localization | 9.05E-03 |

| | | | |
|---|---|---|---|
| blue | GO:0006886 | intracellular protein transport | 9.97E-03 |
| blue | GO:0031098 | stress-activated protein kinase signaling pathway | 1.03E-02 |
| blue | GO:0019637 | organophosphate metabolic process | 1.05E-02 |
| blue | GO:0032312 | regulation of ARF GTPase activity | 1.06E-02 |
| blue | GO:0016310 | phosphorylation | 1.06E-02 |
| blue | GO:0006913 | nucleocytoplasmic transport | 1.07E-02 |
| blue | GO:0015031 | protein transport | 1.07E-02 |
| blue | GO:0006644 | phospholipid metabolic process | 1.12E-02 |
| blue | GO:0006289 | nucleotide-excision repair | 1.20E-02 |
| blue | GO:0051169 | nuclear transport | 1.21E-02 |
| blue | GO:0045814 | negative regulation of gene expression, epigenetic | 1.23E-02 |
| blue | GO:0006605 | protein targeting | 1.28E-02 |
| blue | GO:0006338 | chromatin remodeling | 1.35E-02 |
| blue | GO:0016458 | gene silencing | 1.35E-02 |
| blue | GO:0006670 | sphingosine metabolic process | 1.35E-02 |
| blue | GO:0042048 | olfactory behavior | 1.35E-02 |
| blue | GO:0006914 | autophagy | 1.37E-02 |
| blue | GO:0016571 | histone methylation | 1.50E-02 |
| blue | GO:0006974 | response to DNA damage stimulus | 1.55E-02 |
| blue | GO:0034613 | cellular protein localization | 1.62E-02 |
| blue | GO:0016192 | vesicle-mediated transport | 1.76E-02 |
| blue | GO:0035023 | regulation of Rho protein signal transduction | 1.78E-02 |
| blue | GO:0006915 | apoptosis | 1.78E-02 |
| blue | GO:0070727 | cellular macromolecule localization | 1.81E-02 |
| blue | GO:0007254 | JNK cascade | 1.86E-02 |
| blue | GO:0007635 | chemosensory behavior | 1.99E-02 |
| blue | GO:0051726 | regulation of cell cycle | 2.00E-02 |
| blue | GO:0008654 | phospholipid biosynthetic process | 2.23E-02 |
| blue | GO:0012501 | programmed cell death | 2.34E-02 |
| blue | GO:0051338 | regulation of transferase activity | 2.39E-02 |
| blue | GO:0051329 | interphase of mitotic cell cycle | 2.40E-02 |
| blue | GO:0050772 | positive regulation of axonogenesis | 2.55E-02 |
| blue | GO:0032318 | regulation of Ras GTPase activity | 2.58E-02 |
| blue | GO:0008219 | cell death | 2.67E-02 |

| | | | |
|---|---|---|---|
| blue | GO:0034504 | protein localization in nucleus | 2.72E-02 |
| blue | GO:0051325 | interphase | 2.96E-02 |
| blue | GO:0016265 | death | 3.07E-02 |
| blue | GO:0002275 | myeloid cell activation during immune response | 3.13E-02 |
| blue | GO:0006650 | glycerophospholipid metabolic process | 3.16E-02 |
| blue | GO:0006399 | tRNA metabolic process | 3.16E-02 |
| blue | GO:0043122 | regulation of I-kappaB kinase/NF-kappaB cascade | 3.17E-02 |
| blue | GO:0045892 | negative regulation of transcription, DNA-dependent | 3.21E-02 |
| blue | GO:0006396 | RNA processing | 3.25E-02 |
| blue | GO:0010558 | negative regulation of macromolecule biosynthetic process | 3.25E-02 |
| blue | GO:0046328 | regulation of JNK cascade | 3.29E-02 |
| blue | GO:0006606 | protein import into nucleus | 3.31E-02 |
| blue | GO:0043549 | regulation of kinase activity | 3.33E-02 |
| blue | GO:0045859 | regulation of protein kinase activity | 3.36E-02 |
| blue | GO:0009890 | negative regulation of biosynthetic process | 3.39E-02 |
| blue | GO:0031327 | negative regulation of cellular biosynthetic process | 3.44E-02 |
| blue | GO:0000082 | G1/S transition of mitotic cell cycle | 3.65E-02 |
| blue | GO:0006383 | transcription from RNA polymerase III promoter | 3.75E-02 |
| blue | GO:0035176 | social behavior | 3.75E-02 |
| blue | GO:0051170 | nuclear import | 3.83E-02 |
| blue | GO:0046907 | intracellular transport | 3.98E-02 |
| blue | GO:0042384 | cilium assembly | 3.98E-02 |
| blue | GO:0006796 | phosphate metabolic process | 3.98E-02 |
| blue | GO:0006793 | phosphorus metabolic process | 3.98E-02 |
| blue | GO:0051253 | negative regulation of RNA metabolic process | 3.98E-02 |
| blue | GO:0050770 | regulation of axonogenesis | 4.00E-02 |
| blue | GO:0006346 | methylation-dependent chromatin silencing | 4.11E-02 |
| blue | GO:0046474 | glycerophospholipid biosynthetic process | 4.23E-02 |
| blue | GO:0042158 | lipoprotein biosynthetic process | 4.38E-02 |
| blue | GO:0006259 | DNA metabolic process | 4.40E-02 |
| blue | GO:0070302 | regulation of stress-activated protein kinase signaling pathway | 4.59E-02 |
| blue | GO:0010975 | regulation of neuron projection development | 4.96E-02 |
| magenta | GO:0048704 | embryonic skeletal system morphogenesis | 6.27E-07 |
| magenta | GO:0051252 | regulation of RNA metabolic process | 1.25E-06 |

| | | | |
|---|---|---|---|
| magenta | GO:0006355 | regulation of transcription, DNA-dependent | 2.14E-06 |
| magenta | GO:0048706 | embryonic skeletal system development | 4.93E-06 |
| magenta | GO:0048562 | embryonic organ morphogenesis | 2.22E-05 |
| magenta | GO:0048705 | skeletal system morphogenesis | 5.77E-05 |
| magenta | GO:0048568 | embryonic organ development | 1.37E-04 |
| magenta | GO:0009952 | anterior/posterior pattern formation | 2.34E-04 |
| magenta | GO:0045449 | regulation of transcription | 3.01E-04 |
| magenta | GO:0048598 | embryonic morphogenesis | 3.75E-04 |
| magenta | GO:0043009 | chordate embryonic development | 6.75E-04 |
| magenta | GO:0009792 | embryonic development ending in birth or egg hatching | 7.24E-04 |
| magenta | GO:0006350 | transcription | 9.90E-04 |
| magenta | GO:0003002 | regionalization | 1.77E-03 |
| magenta | GO:0010604 | positive regulation of macromolecule metabolic process | 3.38E-03 |
| magenta | GO:0060429 | epithelium development | 3.91E-03 |
| magenta | GO:0010628 | positive regulation of gene expression | 5.08E-03 |
| magenta | GO:0001501 | skeletal system development | 7.15E-03 |
| magenta | GO:0000122 | negative regulation of transcription from RNA polymerase II promoter | 9.10E-03 |
| magenta | GO:0007389 | pattern specification process | 9.27E-03 |
| magenta | GO:0045893 | positive regulation of transcription, DNA-dependent | 9.35E-03 |
| magenta | GO:0051254 | positive regulation of RNA metabolic process | 9.88E-03 |
| magenta | GO:0045941 | positive regulation of transcription | 1.09E-02 |
| magenta | GO:0045935 | positive regulation of nucleobase, nucleoside, nucleotide and nucleic acid metabolic process | 2.15E-02 |
| magenta | GO:0050821 | protein stabilization | 2.50E-02 |
| magenta | GO:0006357 | regulation of transcription from RNA polymerase II promoter | 2.62E-02 |
| magenta | GO:0051173 | positive regulation of nitrogen compound metabolic process | 2.63E-02 |
| magenta | GO:0010557 | positive regulation of macromolecule biosynthetic process | 2.91E-02 |
| magenta | GO:0043632 | modification-dependent macromolecule catabolic process | 2.99E-02 |
| magenta | GO:0019941 | modification-dependent protein catabolic process | 2.99E-02 |
| magenta | GO:0034644 | cellular response to UV | 3.47E-02 |
| magenta | GO:0045892 | negative regulation of transcription, DNA-dependent | 3.77E-02 |
| magenta | GO:0051603 | proteolysis involved in cellular protein catabolic process | 3.86E-02 |
| magenta | GO:0031328 | positive regulation of cellular biosynthetic process | 3.87E-02 |
| magenta | GO:0044257 | cellular protein catabolic process | 3.98E-02 |
| magenta | GO:0051253 | negative regulation of RNA metabolic process | 4.07E-02 |

| | | | |
|---|---|---|---|
| magenta | GO:0009891 | positive regulation of biosynthetic process | 4.23E-02 |
| magenta | GO:0045944 | positive regulation of transcription from RNA polymerase II promoter | 4.54E-02 |
| magenta | GO:0030163 | protein catabolic process | 4.74E-02 |
| red | GO:0003012 | muscle system process | 9.46E-16 |
| red | GO:0007517 | muscle organ development | 5.57E-10 |
| red | GO:0007010 | cytoskeleton organization | 9.16E-07 |
| red | GO:0060537 | muscle tissue development | 3.34E-06 |
| red | GO:0034329 | cell junction assembly | 3.43E-05 |
| red | GO:0030036 | actin cytoskeleton organization | 4.51E-05 |
| red | GO:0031032 | actomyosin structure organization | 5.90E-05 |
| red | GO:0006941 | striated muscle contraction | 6.71E-05 |
| red | GO:0042692 | muscle cell differentiation | 9.95E-05 |
| red | GO:0007507 | heart development | 1.18E-04 |
| red | GO:0006939 | smooth muscle contraction | 1.34E-04 |
| red | GO:0055002 | striated muscle cell development | 1.35E-04 |
| red | GO:0030705 | cytoskeleton-dependent intracellular transport | 1.35E-04 |
| red | GO:0008016 | regulation of heart contraction | 1.72E-04 |
| red | GO:0010927 | cellular component assembly involved in morphogenesis | 2.05E-04 |
| red | GO:0055001 | muscle cell development | 2.05E-04 |
| red | GO:0034330 | cell junction organization | 2.26E-04 |
| red | GO:0030239 | myofibril assembly | 2.60E-04 |
| red | GO:0006940 | regulation of smooth muscle contraction | 2.66E-04 |
| red | GO:0007044 | cell-substrate junction assembly | 3.75E-04 |
| red | GO:0030048 | actin filament-based movement | 3.75E-04 |
| red | GO:0051146 | striated muscle cell differentiation | 3.95E-04 |
| red | GO:0007155 | cell adhesion | 4.51E-04 |
| red | GO:0022610 | biological adhesion | 4.60E-04 |
| red | GO:0008015 | blood circulation | 5.86E-04 |
| red | GO:0003013 | circulatory system process | 5.86E-04 |
| red | GO:0050880 | regulation of blood vessel size | 1.26E-03 |
| red | GO:0035150 | regulation of tube size | 1.26E-03 |
| red | GO:0003018 | vascular process in circulatory system | 1.90E-03 |
| red | GO:0042310 | vasoconstriction | 2.05E-03 |
| red | GO:0007160 | cell-matrix adhesion | 2.42E-03 |

| | | | |
|---|---|---|---|
| red | GO:0031589 | cell-substrate adhesion | 3.91E-03 |
| red | GO:0030030 | cell projection organization | 4.40E-03 |
| red | GO:0007015 | actin filament organization | 4.88E-03 |
| red | GO:0051493 | regulation of cytoskeleton organization | 4.91E-03 |
| red | GO:0006633 | fatty acid biosynthetic process | 7.21E-03 |
| red | GO:0014829 | vascular smooth muscle contraction | 7.82E-03 |
| red | GO:0030049 | muscle filament sliding | 7.82E-03 |
| red | GO:0033275 | actin-myosin filament sliding | 7.82E-03 |
| red | GO:0070252 | actin-mediated cell contraction | 7.82E-03 |
| red | GO:0032989 | cellular component morphogenesis | 8.18E-03 |
| red | GO:0051592 | response to calcium ion | 9.88E-03 |
| red | GO:0046456 | icosanoid biosynthetic process | 1.16E-02 |
| red | GO:0010035 | response to inorganic substance | 1.37E-02 |
| red | GO:0006928 | cell motion | 1.45E-02 |
| red | GO:0006636 | unsaturated fatty acid biosynthetic process | 1.49E-02 |
| red | GO:0051271 | negative regulation of cell motion | 1.57E-02 |
| red | GO:0005977 | glycogen metabolic process | 1.61E-02 |
| red | GO:0031175 | neuron projection development | 1.71E-02 |
| red | GO:0006073 | cellular glucan metabolic process | 1.74E-02 |
| red | GO:0044042 | glucan metabolic process | 1.74E-02 |
| red | GO:0033043 | regulation of organelle organization | 1.86E-02 |
| red | GO:0008217 | regulation of blood pressure | 1.87E-02 |
| red | GO:0051924 | regulation of calcium ion transport | 2.13E-02 |
| red | GO:0007229 | integrin-mediated signaling pathway | 2.23E-02 |
| red | GO:0043462 | regulation of ATPase activity | 2.43E-02 |
| red | GO:0006112 | energy reserve metabolic process | 2.78E-02 |
| red | GO:0045987 | positive regulation of smooth muscle contraction | 3.04E-02 |
| red | GO:0051495 | positive regulation of cytoskeleton organization | 3.13E-02 |
| red | GO:0060048 | cardiac muscle contraction | 3.36E-02 |
| red | GO:0006690 | icosanoid metabolic process | 3.50E-02 |
| red | GO:0010959 | regulation of metal ion transport | 3.56E-02 |
| red | GO:0048666 | neuron development | 3.60E-02 |
| red | GO:0010638 | positive regulation of organelle organization | 3.84E-02 |
| red | GO:0007268 | synaptic transmission | 3.99E-02 |

| | | | |
|---|---|---|---|
| red | GO:0045933 | positive regulation of muscle contraction | 4.04E-02 |
| red | GO:0033559 | unsaturated fatty acid metabolic process | 4.30E-02 |
| red | GO:0043244 | regulation of protein complex disassembly | 4.30E-02 |
| red | GO:0019226 | transmission of nerve impulse | 4.30E-02 |
| red | GO:0007264 | small GTPase mediated signal transduction | 4.51E-02 |
| red | GO:0007412 | axon target recognition | 4.52E-02 |
| red | GO:0003056 | regulation of vascular smooth muscle contraction | 4.52E-02 |
| red | GO:0048812 | neuron projection morphogenesis | 4.55E-02 |
| red | GO:0044264 | cellular polysaccharide metabolic process | 4.73E-02 |
| red | GO:0003015 | heart process | 4.78E-02 |
| red | GO:0060047 | heart contraction | 4.78E-02 |
| red | GO:0010038 | response to metal ion | 4.81E-02 |
| red | GO:0042493 | response to drug | 4.85E-02 |
| yellow | GO:0009725 | response to hormone stimulus | 2.46E-11 |
| yellow | GO:0009719 | response to endogenous stimulus | 7.84E-11 |
| yellow | GO:0032868 | response to insulin stimulus | 9.37E-09 |
| yellow | GO:0010033 | response to organic substance | 3.24E-08 |
| yellow | GO:0001568 | blood vessel development | 1.14E-06 |
| yellow | GO:0001944 | vasculature development | 1.66E-06 |
| yellow | GO:0001525 | angiogenesis | 8.86E-06 |
| yellow | GO:0007169 | transmembrane receptor protein tyrosine kinase signaling pathway | 1.91E-05 |
| yellow | GO:0048514 | blood vessel morphogenesis | 3.39E-05 |
| yellow | GO:0007242 | intracellular signaling cascade | 5.31E-05 |
| yellow | GO:0045785 | positive regulation of cell adhesion | 6.90E-05 |
| yellow | GO:0030334 | regulation of cell migration | 1.59E-04 |
| yellow | GO:0040012 | regulation of locomotion | 1.59E-04 |
| yellow | GO:0051270 | regulation of cell motion | 1.68E-04 |
| yellow | GO:0009755 | hormone-mediated signaling | 1.85E-04 |
| yellow | GO:0008286 | insulin receptor signaling pathway | 1.89E-04 |
| yellow | GO:0008360 | regulation of cell shape | 2.36E-04 |
| yellow | GO:0030802 | regulation of cyclic nucleotide biosynthetic process | 2.37E-04 |
| yellow | GO:0030808 | regulation of nucleotide biosynthetic process | 2.37E-04 |
| yellow | GO:0030799 | regulation of cyclic nucleotide metabolic process | 2.96E-04 |
| yellow | GO:0019933 | cAMP-mediated signaling | 3.56E-04 |

| | | | |
|---|---|---|---|
| yellow | GO:0006140 | regulation of nucleotide metabolic process | 3.65E-04 |
| yellow | GO:0006101 | citrate metabolic process | 4.05E-04 |
| yellow | GO:0034754 | cellular hormone metabolic process | 4.11E-04 |
| yellow | GO:0007243 | protein kinase cascade | 4.11E-04 |
| yellow | GO:0010811 | positive regulation of cell-substrate adhesion | 4.13E-04 |
| yellow | GO:0031279 | regulation of cyclase activity | 4.84E-04 |
| yellow | GO:0051339 | regulation of lyase activity | 5.61E-04 |
| yellow | GO:0010810 | regulation of cell-substrate adhesion | 6.39E-04 |
| yellow | GO:0031667 | response to nutrient levels | 7.05E-04 |
| yellow | GO:0042127 | regulation of cell proliferation | 7.26E-04 |
| yellow | GO:0006631 | fatty acid metabolic process | 7.37E-04 |
| yellow | GO:0006006 | glucose metabolic process | 8.85E-04 |
| yellow | GO:0033500 | carbohydrate homeostasis | 9.00E-04 |
| yellow | GO:0042593 | glucose homeostasis | 9.00E-04 |
| yellow | GO:0019432 | triglyceride biosynthetic process | 9.38E-04 |
| yellow | GO:0019216 | regulation of lipid metabolic process | 1.18E-03 |
| yellow | GO:0010906 | regulation of glucose metabolic process | 1.20E-03 |
| yellow | GO:0008203 | cholesterol metabolic process | 1.32E-03 |
| yellow | GO:0008610 | lipid biosynthetic process | 1.47E-03 |
| yellow | GO:0051350 | negative regulation of lyase activity | 1.66E-03 |
| yellow | GO:0031280 | negative regulation of cyclase activity | 1.66E-03 |
| yellow | GO:0007194 | negative regulation of adenylate cyclase activity | 1.66E-03 |
| yellow | GO:0045761 | regulation of adenylate cyclase activity | 1.74E-03 |
| yellow | GO:0010675 | regulation of cellular carbohydrate metabolic process | 1.75E-03 |
| yellow | GO:0019318 | hexose metabolic process | 1.78E-03 |
| yellow | GO:0009991 | response to extracellular stimulus | 1.93E-03 |
| yellow | GO:0008285 | negative regulation of cell proliferation | 1.96E-03 |
| yellow | GO:0006109 | regulation of carbohydrate metabolic process | 1.97E-03 |
| yellow | GO:0040017 | positive regulation of locomotion | 1.99E-03 |
| yellow | GO:0031589 | cell-substrate adhesion | 1.99E-03 |
| yellow | GO:0051272 | positive regulation of cell motion | 1.99E-03 |
| yellow | GO:0005996 | monosaccharide metabolic process | 2.09E-03 |
| yellow | GO:0046463 | acylglycerol biosynthetic process | 2.33E-03 |
| yellow | GO:0046460 | neutral lipid biosynthetic process | 2.33E-03 |

| | | | |
|---|---|---|---|
| yellow | GO:0051056 | regulation of small GTPase mediated signal transduction | 2.34E-03 |
| yellow | GO:0030817 | regulation of cAMP biosynthetic process | 2.40E-03 |
| yellow | GO:0016125 | sterol metabolic process | 2.40E-03 |
| yellow | GO:0030036 | actin cytoskeleton organization | 2.44E-03 |
| yellow | GO:0030814 | regulation of cAMP metabolic process | 2.72E-03 |
| yellow | GO:0008643 | carbohydrate transport | 2.83E-03 |
| yellow | GO:0030198 | extracellular matrix organization | 2.88E-03 |
| yellow | GO:0046504 | glycerol ether biosynthetic process | 2.98E-03 |
| yellow | GO:0042445 | hormone metabolic process | 3.25E-03 |
| yellow | GO:0019935 | cyclic-nucleotide-mediated signaling | 3.30E-03 |
| yellow | GO:0007188 | G-protein signaling, coupled to cAMP nucleotide second messenger | 3.57E-03 |
| yellow | GO:0022610 | biological adhesion | 3.60E-03 |
| yellow | GO:0007155 | cell adhesion | 3.63E-03 |
| yellow | GO:0046578 | regulation of Ras protein signal transduction | 3.73E-03 |
| yellow | GO:0030029 | actin filament-based process | 4.24E-03 |
| yellow | GO:0030335 | positive regulation of cell migration | 4.61E-03 |
| yellow | GO:0007160 | cell-matrix adhesion | 4.61E-03 |
| yellow | GO:0043467 | regulation of generation of precursor metabolites and energy | 4.85E-03 |
| yellow | GO:0044093 | positive regulation of molecular function | 5.10E-03 |
| yellow | GO:0006573 | valine metabolic process | 5.13E-03 |
| yellow | GO:0019530 | taurine metabolic process | 5.13E-03 |
| yellow | GO:0007584 | response to nutrient | 5.37E-03 |
| yellow | GO:0006639 | acylglycerol metabolic process | 5.40E-03 |
| yellow | GO:0006638 | neutral lipid metabolic process | 5.88E-03 |
| yellow | GO:0045860 | positive regulation of protein kinase activity | 6.01E-03 |
| yellow | GO:0050678 | regulation of epithelial cell proliferation | 6.03E-03 |
| yellow | GO:0006662 | glycerol ether metabolic process | 6.40E-03 |
| yellow | GO:0007010 | cytoskeleton organization | 6.48E-03 |
| yellow | GO:0034199 | activation of protein kinase A activity | 6.62E-03 |
| yellow | GO:0006468 | protein amino acid phosphorylation | 6.75E-03 |
| yellow | GO:0018904 | organic ether metabolic process | 7.53E-03 |
| yellow | GO:0008202 | steroid metabolic process | 7.68E-03 |
| yellow | GO:0019915 | lipid storage | 7.80E-03 |
| yellow | GO:0042551 | neuron maturation | 7.80E-03 |

| | | | |
|---|---|---|---|
| yellow | GO:0006766 | vitamin metabolic process | 7.85E-03 |
| yellow | GO:0033674 | positive regulation of kinase activity | 7.90E-03 |
| yellow | GO:0008284 | positive regulation of cell proliferation | 8.07E-03 |
| yellow | GO:0010907 | positive regulation of glucose metabolic process | 9.11E-03 |
| yellow | GO:0043085 | positive regulation of catalytic activity | 9.88E-03 |
| yellow | GO:0060173 | limb development | 1.01E-02 |
| yellow | GO:0048736 | appendage development | 1.01E-02 |
| yellow | GO:0030336 | negative regulation of cell migration | 1.02E-02 |
| yellow | GO:0006700 | C21-steroid hormone biosynthetic process | 1.04E-02 |
| yellow | GO:0007517 | muscle organ development | 1.05E-02 |
| yellow | GO:0051347 | positive regulation of transferase activity | 1.05E-02 |
| yellow | GO:0045913 | positive regulation of carbohydrate metabolic process | 1.05E-02 |
| yellow | GO:0010676 | positive regulation of cellular carbohydrate metabolic process | 1.05E-02 |
| yellow | GO:0032535 | regulation of cellular component size | 1.09E-02 |
| yellow | GO:0009187 | cyclic nucleotide metabolic process | 1.13E-02 |
| yellow | GO:0022604 | regulation of cell morphogenesis | 1.14E-02 |
| yellow | GO:0001569 | patterning of blood vessels | 1.21E-02 |
| yellow | GO:0046058 | cAMP metabolic process | 1.21E-02 |
| yellow | GO:0043255 | regulation of carbohydrate biosynthetic process | 1.21E-02 |
| yellow | GO:0060326 | cell chemotaxis | 1.24E-02 |
| yellow | GO:0016477 | cell migration | 1.26E-02 |
| yellow | GO:0060341 | regulation of cellular localization | 1.34E-02 |
| yellow | GO:0016049 | cell growth | 1.35E-02 |
| yellow | GO:0040013 | negative regulation of locomotion | 1.35E-02 |
| yellow | GO:0040014 | regulation of multicellular organism growth | 1.35E-02 |
| yellow | GO:0044057 | regulation of system process | 1.35E-02 |
| yellow | GO:0007193 | inhibition of adenylate cyclase activity by G-protein signaling | 1.35E-02 |
| yellow | GO:0050796 | regulation of insulin secretion | 1.35E-02 |
| yellow | GO:0040008 | regulation of growth | 1.36E-02 |
| yellow | GO:0043062 | extracellular structure organization | 1.39E-02 |
| yellow | GO:0007568 | aging | 1.41E-02 |
| yellow | GO:0048545 | response to steroid hormone stimulus | 1.44E-02 |
| yellow | GO:0006091 | generation of precursor metabolites and energy | 1.49E-02 |
| yellow | GO:0051271 | negative regulation of cell motion | 1.53E-02 |

| | | | |
|---|---|---|---|
| yellow | GO:0001523 | retinoid metabolic process | 1.56E-02 |
| yellow | GO:0016101 | diterpenoid metabolic process | 1.56E-02 |
| yellow | GO:0031328 | positive regulation of cellular biosynthetic process | 1.72E-02 |
| yellow | GO:0006641 | triglyceride metabolic process | 1.73E-02 |
| yellow | GO:0048754 | branching morphogenesis of a tube | 1.73E-02 |
| yellow | GO:0010884 | positive regulation of lipid storage | 1.74E-02 |
| yellow | GO:0046580 | negative regulation of Ras protein signal transduction | 1.74E-02 |
| yellow | GO:0051058 | negative regulation of small GTPase mediated signal transduction | 1.74E-02 |
| yellow | GO:0007187 | G-protein signaling, coupled to cyclic nucleotide second messenger | 1.77E-02 |
| yellow | GO:0010557 | positive regulation of macromolecule biosynthetic process | 1.81E-02 |
| yellow | GO:0015980 | energy derivation by oxidation of organic compounds | 1.91E-02 |
| yellow | GO:0045935 | positive regulation of nucleobase, nucleoside, nucleotide and nucleic acid metabolic process | 1.94E-02 |
| yellow | GO:0006721 | terpenoid metabolic process | 1.95E-02 |
| yellow | GO:0009190 | cyclic nucleotide biosynthetic process | 1.95E-02 |
| yellow | GO:0040018 | positive regulation of multicellular organism growth | 1.95E-02 |
| yellow | GO:0009891 | positive regulation of biosynthetic process | 1.97E-02 |
| yellow | GO:0051046 | regulation of secretion | 1.98E-02 |
| yellow | GO:0046890 | regulation of lipid biosynthetic process | 2.01E-02 |
| yellow | GO:0006107 | oxaloacetate metabolic process | 2.14E-02 |
| yellow | GO:0002791 | regulation of peptide secretion | 2.16E-02 |
| yellow | GO:0018108 | peptidyl-tyrosine phosphorylation | 2.16E-02 |
| yellow | GO:0008361 | regulation of cell size | 2.26E-02 |
| yellow | GO:0014070 | response to organic cyclic substance | 2.27E-02 |
| yellow | GO:0042325 | regulation of phosphorylation | 2.44E-02 |
| yellow | GO:0010817 | regulation of hormone levels | 2.46E-02 |
| yellow | GO:0018212 | peptidyl-tyrosine modification | 2.49E-02 |
| yellow | GO:0051173 | positive regulation of nitrogen compound metabolic process | 2.71E-02 |
| yellow | GO:0040007 | growth | 2.74E-02 |
| yellow | GO:0042592 | homeostatic process | 2.74E-02 |
| yellow | GO:0051674 | localization of cell | 2.75E-02 |
| yellow | GO:0048870 | cell motility | 2.75E-02 |
| yellow | GO:0035107 | appendage morphogenesis | 2.77E-02 |
| yellow | GO:0035023 | regulation of Rho protein signal transduction | 2.77E-02 |
| yellow | GO:0035108 | limb morphogenesis | 2.77E-02 |

| | | | |
|---|---|---|---|
| yellow | GO:0051240 | positive regulation of multicellular organismal process | 2.80E-02 |
| yellow | GO:0016053 | organic acid biosynthetic process | 2.82E-02 |
| yellow | GO:0046394 | carboxylic acid biosynthetic process | 2.82E-02 |
| yellow | GO:0000165 | MAPKKK cascade | 2.82E-02 |
| yellow | GO:0016202 | regulation of striated muscle tissue development | 2.84E-02 |
| yellow | GO:0001763 | morphogenesis of a branching structure | 2.86E-02 |
| yellow | GO:0030856 | regulation of epithelial cell differentiation | 2.90E-02 |
| yellow | GO:0010628 | positive regulation of gene expression | 3.00E-02 |
| yellow | GO:0048469 | cell maturation | 3.01E-02 |
| yellow | GO:0021700 | developmental maturation | 3.01E-02 |
| yellow | GO:0048634 | regulation of muscle development | 3.03E-02 |
| yellow | GO:0048878 | chemical homeostasis | 3.03E-02 |
| yellow | GO:0008207 | C21-steroid hormone metabolic process | 3.04E-02 |
| yellow | GO:0045859 | regulation of protein kinase activity | 3.09E-02 |
| yellow | GO:0032101 | regulation of response to external stimulus | 3.21E-02 |
| yellow | GO:0016310 | phosphorylation | 3.24E-02 |
| yellow | GO:0042330 | taxis | 3.32E-02 |
| yellow | GO:0006935 | chemotaxis | 3.32E-02 |
| yellow | GO:0035295 | tube development | 3.32E-02 |
| yellow | GO:0051174 | regulation of phosphorus metabolic process | 3.42E-02 |
| yellow | GO:0019220 | regulation of phosphate metabolic process | 3.42E-02 |
| yellow | GO:0042446 | hormone biosynthetic process | 3.45E-02 |
| yellow | GO:0042060 | wound healing | 3.48E-02 |
| yellow | GO:0051384 | response to glucocorticoid stimulus | 3.48E-02 |
| yellow | GO:0010595 | positive regulation of endothelial cell migration | 3.54E-02 |
| yellow | GO:0007265 | Ras protein signal transduction | 3.55E-02 |
| yellow | GO:0019748 | secondary metabolic process | 3.65E-02 |
| yellow | GO:0031327 | negative regulation of cellular biosynthetic process | 3.79E-02 |
| yellow | GO:0051051 | negative regulation of transport | 3.80E-02 |
| yellow | GO:0016051 | carbohydrate biosynthetic process | 3.84E-02 |
| yellow | GO:0043549 | regulation of kinase activity | 3.94E-02 |
| yellow | GO:0045941 | positive regulation of transcription | 3.98E-02 |
| yellow | GO:0046324 | regulation of glucose import | 4.05E-02 |
| yellow | GO:0032526 | response to retinoic acid | 4.05E-02 |

| | | | |
|---|---|---|---|
| yellow | GO:0006775 | fat-soluble vitamin metabolic process | 4.05E-02 |
| yellow | GO:0006171 | cAMP biosynthetic process | 4.07E-02 |
| yellow | GO:0035265 | organ growth | 4.07E-02 |
| yellow | GO:0042542 | response to hydrogen peroxide | 4.07E-02 |
| yellow | GO:0048771 | tissue remodeling | 4.07E-02 |
| yellow | GO:0003012 | muscle system process | 4.23E-02 |
| yellow | GO:0055114 | oxidation reduction | 4.24E-02 |
| yellow | GO:0010604 | positive regulation of macromolecule metabolic process | 4.25E-02 |
| yellow | GO:0010565 | regulation of cellular ketone metabolic process | 4.30E-02 |
| yellow | GO:0031281 | positive regulation of cyclase activity | 4.30E-02 |
| yellow | GO:0010827 | regulation of glucose transport | 4.36E-02 |
| yellow | GO:0007166 | cell surface receptor linked signal transduction | 4.50E-02 |
| yellow | GO:0009890 | negative regulation of biosynthetic process | 4.56E-02 |
| yellow | GO:0022617 | extracellular matrix disassembly | 4.59E-02 |
| yellow | GO:0032881 | regulation of polysaccharide metabolic process | 4.62E-02 |
| yellow | GO:0009060 | aerobic respiration | 4.69E-02 |
| yellow | GO:0048871 | multicellular organismal homeostasis | 4.76E-02 |
| yellow | GO:0031960 | response to corticosteroid stimulus | 4.76E-02 |
| yellow | GO:0006694 | steroid biosynthetic process | 4.76E-02 |
| yellow | GO:0051349 | positive regulation of lyase activity | 4.79E-02 |
| yellow | GO:0019932 | second-messenger-mediated signaling | 4.84E-02 |
| yellow | GO:0032147 | activation of protein kinase activity | 4.96E-02 |
| cyan | GO:0006959 | humoral immune response | 4.78E-06 |
| cyan | GO:0032101 | regulation of response to external stimulus | 2.76E-05 |
| cyan | GO:0009611 | response to wounding | 3.17E-05 |
| cyan | GO:0051384 | response to glucocorticoid stimulus | 7.05E-05 |
| cyan | GO:0006954 | inflammatory response | 7.44E-05 |
| cyan | GO:0031960 | response to corticosteroid stimulus | 1.06E-04 |
| cyan | GO:0030323 | respiratory tube development | 2.51E-04 |
| cyan | GO:0060541 | respiratory system development | 3.27E-04 |
| cyan | GO:0035295 | tube development | 1.37E-03 |
| cyan | GO:0006956 | complement activation | 1.61E-03 |
| cyan | GO:0002541 | activation of plasma proteins involved in acute inflammatory response | 1.72E-03 |
| cyan | GO:0002253 | activation of immune response | 1.82E-03 |

| | | | |
|---|---|---|---|
| cyan | GO:0048584 | positive regulation of response to stimulus | 1.95E-03 |
| cyan | GO:0002684 | positive regulation of immune system process | 2.04E-03 |
| cyan | GO:0002526 | acute inflammatory response | 2.12E-03 |
| cyan | GO:0030198 | extracellular matrix organization | 2.64E-03 |
| cyan | GO:0001944 | vasculature development | 2.66E-03 |
| cyan | GO:0006957 | complement activation, alternative pathway | 3.04E-03 |
| cyan | GO:0009612 | response to mechanical stimulus | 3.67E-03 |
| cyan | GO:0050900 | leukocyte migration | 3.86E-03 |
| cyan | GO:0009725 | response to hormone stimulus | 4.16E-03 |
| cyan | GO:0048545 | response to steroid hormone stimulus | 4.23E-03 |
| cyan | GO:0016477 | cell migration | 4.24E-03 |
| cyan | GO:0006955 | immune response | 4.61E-03 |
| cyan | GO:0009314 | response to radiation | 5.02E-03 |
| cyan | GO:0007155 | cell adhesion | 5.10E-03 |
| cyan | GO:0022610 | biological adhesion | 5.15E-03 |
| cyan | GO:0002673 | regulation of acute inflammatory response | 5.95E-03 |
| cyan | GO:0010033 | response to organic substance | 6.25E-03 |
| cyan | GO:0002252 | immune effector process | 6.50E-03 |
| cyan | GO:0048286 | lung alveolus development | 6.52E-03 |
| cyan | GO:0006952 | defense response | 6.76E-03 |
| cyan | GO:0051674 | localization of cell | 7.06E-03 |
| cyan | GO:0048870 | cell motility | 7.06E-03 |
| cyan | GO:0009719 | response to endogenous stimulus | 7.07E-03 |
| cyan | GO:0050778 | positive regulation of immune response | 8.55E-03 |
| cyan | GO:0045768 | positive regulation of anti-apoptosis | 1.19E-02 |
| cyan | GO:0002237 | response to molecule of bacterial origin | 1.21E-02 |
| cyan | GO:0043062 | extracellular structure organization | 1.27E-02 |
| cyan | GO:0030334 | regulation of cell migration | 1.44E-02 |
| cyan | GO:0006928 | cell motion | 1.60E-02 |
| cyan | GO:0009628 | response to abiotic stimulus | 1.62E-02 |
| cyan | GO:0031348 | negative regulation of defense response | 1.69E-02 |
| cyan | GO:0045767 | regulation of anti-apoptosis | 1.87E-02 |
| cyan | GO:0050817 | coagulation | 1.90E-02 |
| cyan | GO:0007596 | blood coagulation | 1.90E-02 |

| | | | |
|---|---|---|---|
| cyan | GO:0042060 | wound healing | 2.15E-02 |
| cyan | GO:0040012 | regulation of locomotion | 2.19E-02 |
| cyan | GO:0010572 | positive regulation of platelet activation | 2.20E-02 |
| cyan | GO:0002548 | monocyte chemotaxis | 2.20E-02 |
| cyan | GO:0007599 | hemostasis | 2.21E-02 |
| cyan | GO:0051270 | regulation of cell motion | 2.22E-02 |
| cyan | GO:0001558 | regulation of cell growth | 2.26E-02 |
| cyan | GO:0051591 | response to cAMP | 2.26E-02 |
| cyan | GO:0050867 | positive regulation of cell activation | 2.37E-02 |
| cyan | GO:0008284 | positive regulation of cell proliferation | 2.71E-02 |
| cyan | GO:0042127 | regulation of cell proliferation | 2.94E-02 |
| cyan | GO:0042493 | response to drug | 3.19E-02 |
| cyan | GO:0001501 | skeletal system development | 3.19E-02 |
| cyan | GO:0035239 | tube morphogenesis | 3.35E-02 |
| cyan | GO:0022604 | regulation of cell morphogenesis | 3.62E-02 |
| cyan | GO:0060425 | lung morphogenesis | 3.82E-02 |
| cyan | GO:0045597 | positive regulation of cell differentiation | 3.83E-02 |
| cyan | GO:0009416 | response to light stimulus | 4.12E-02 |
| cyan | GO:0045087 | innate immune response | 4.12E-02 |
| cyan | GO:0050769 | positive regulation of neurogenesis | 4.24E-02 |
| cyan | GO:0050878 | regulation of body fluid levels | 4.35E-02 |
| cyan | GO:0070482 | response to oxygen levels | 4.35E-02 |
| cyan | GO:0055093 | response to hyperoxia | 4.35E-02 |
| cyan | GO:0010543 | regulation of platelet activation | 4.35E-02 |
| cyan | GO:0001568 | blood vessel development | 4.70E-02 |
| cyan | GO:0045893 | positive regulation of transcription, DNA-dependent | 4.87E-02 |
| cyan | GO:0032103 | positive regulation of response to external stimulus | 4.90E-02 |
| tan | GO:0007049 | cell cycle | 6.19E-36 |
| tan | GO:0022403 | cell cycle phase | 5.04E-35 |
| tan | GO:0000279 | M phase | 5.86E-30 |
| tan | GO:0022402 | cell cycle process | 6.32E-30 |
| tan | GO:0000278 | mitotic cell cycle | 2.54E-28 |
| tan | GO:0051301 | cell division | 8.76E-26 |
| tan | GO:0007067 | mitosis | 1.63E-23 |

| | | | |
|---|---|---|---|
| tan | GO:0000280 | nuclear division | 1.63E-23 |
| tan | GO:0000087 | M phase of mitotic cell cycle | 2.52E-23 |
| tan | GO:0048285 | organelle fission | 4.30E-23 |
| tan | GO:0007059 | chromosome segregation | 1.77E-18 |
| tan | GO:0006259 | DNA metabolic process | 3.94E-16 |
| tan | GO:0006260 | DNA replication | 2.65E-15 |
| tan | GO:0006974 | response to DNA damage stimulus | 1.50E-10 |
| tan | GO:0051726 | regulation of cell cycle | 2.36E-10 |
| tan | GO:0000075 | cell cycle checkpoint | 2.66E-10 |
| tan | GO:0006281 | DNA repair | 2.75E-09 |
| tan | GO:0010564 | regulation of cell cycle process | 4.16E-08 |
| tan | GO:0051276 | chromosome organization | 5.56E-08 |
| tan | GO:0033554 | cellular response to stress | 7.87E-08 |
| tan | GO:0051640 | organelle localization | 1.16E-07 |
| tan | GO:0051329 | interphase of mitotic cell cycle | 2.79E-07 |
| tan | GO:0051325 | interphase | 3.48E-07 |
| tan | GO:0007051 | spindle organization | 3.69E-07 |
| tan | GO:0007346 | regulation of mitotic cell cycle | 4.95E-07 |
| tan | GO:0007017 | microtubule-based process | 5.59E-07 |
| tan | GO:0006261 | DNA-dependent DNA replication | 1.72E-06 |
| tan | GO:0051303 | establishment of chromosome localization | 1.97E-06 |
| tan | GO:0050000 | chromosome localization | 1.97E-06 |
| tan | GO:0000070 | mitotic sister chromatid segregation | 2.98E-06 |
| tan | GO:0000819 | sister chromatid segregation | 3.43E-06 |
| tan | GO:0000226 | microtubule cytoskeleton organization | 4.17E-06 |
| tan | GO:0051656 | establishment of organelle localization | 4.81E-06 |
| tan | GO:0000085 | G2 phase of mitotic cell cycle | 8.52E-06 |
| tan | GO:0051319 | G2 phase | 8.52E-06 |
| tan | GO:0051327 | M phase of meiotic cell cycle | 3.65E-05 |
| tan | GO:0007126 | meiosis | 3.65E-05 |
| tan | GO:0051321 | meiotic cell cycle | 4.09E-05 |
| tan | GO:0007052 | mitotic spindle organization | 1.07E-04 |
| tan | GO:0007093 | mitotic cell cycle checkpoint | 1.56E-04 |
| tan | GO:0051383 | kinetochore organization | 2.38E-04 |

| | | | |
|---|---|---|---|
| tan | GO:0031570 | DNA integrity checkpoint | 3.27E-04 |
| tan | GO:0034508 | centromere complex assembly | 5.89E-04 |
| tan | GO:0040001 | establishment of mitotic spindle localization | 1.09E-03 |
| tan | GO:0051293 | establishment of spindle localization | 1.74E-03 |
| tan | GO:0051653 | spindle localization | 1.74E-03 |
| tan | GO:0051310 | metaphase plate congression | 2.12E-03 |
| tan | GO:0000910 | cytokinesis | 2.22E-03 |
| tan | GO:0048015 | phosphoinositide-mediated signaling | 2.37E-03 |
| tan | GO:0007096 | regulation of exit from mitosis | 2.53E-03 |
| tan | GO:0031577 | spindle checkpoint | 2.53E-03 |
| tan | GO:0000077 | DNA damage checkpoint | 3.49E-03 |
| tan | GO:0006310 | DNA recombination | 4.47E-03 |
| tan | GO:0006297 | nucleotide-excision repair, DNA gap filling | 5.11E-03 |
| tan | GO:0007088 | regulation of mitosis | 5.39E-03 |
| tan | GO:0051783 | regulation of nuclear division | 5.39E-03 |
| tan | GO:0045787 | positive regulation of cell cycle | 5.67E-03 |
| tan | GO:0008283 | cell proliferation | 6.45E-03 |
| tan | GO:0007010 | cytoskeleton organization | 6.45E-03 |
| tan | GO:0010212 | response to ionizing radiation | 6.54E-03 |
| tan | GO:0006275 | regulation of DNA replication | 7.16E-03 |
| tan | GO:0030071 | regulation of mitotic metaphase/anaphase transition | 8.50E-03 |
| tan | GO:0051785 | positive regulation of nuclear division | 1.09E-02 |
| tan | GO:0045840 | positive regulation of mitosis | 1.09E-02 |
| tan | GO:0033043 | regulation of organelle organization | 1.24E-02 |
| tan | GO:0000022 | mitotic spindle elongation | 1.27E-02 |
| tan | GO:0007079 | mitotic chromosome movement towards spindle pole | 1.27E-02 |
| tan | GO:0051231 | spindle elongation | 1.27E-02 |
| tan | GO:0051782 | negative regulation of cell division | 1.27E-02 |
| tan | GO:0008156 | negative regulation of DNA replication | 1.36E-02 |
| tan | GO:0042770 | DNA damage response, signal transduction | 1.43E-02 |
| tan | GO:0010638 | positive regulation of organelle organization | 1.58E-02 |
| tan | GO:0045842 | positive regulation of mitotic metaphase/anaphase transition | 1.90E-02 |
| tan | GO:0034501 | protein localization to kinetochore | 1.90E-02 |
| tan | GO:0048478 | replication fork protection | 1.90E-02 |

| | | | |
|---|---|---|---|
| tan | GO:0051382 | kinetochore assembly | 1.90E-02 |
| tan | GO:0051305 | chromosome movement towards spindle pole | 1.90E-02 |
| tan | GO:0031536 | positive regulation of exit from mitosis | 2.52E-02 |
| tan | GO:0051053 | negative regulation of DNA metabolic process | 2.54E-02 |
| tan | GO:0007127 | meiosis I | 2.92E-02 |
| tan | GO:0016568 | chromatin modification | 3.03E-02 |
| tan | GO:0002520 | immune system development | 3.12E-02 |
| tan | GO:0006271 | DNA strand elongation during DNA replication | 3.14E-02 |
| tan | GO:0045005 | maintenance of fidelity during DNA-dependent DNA replication | 3.14E-02 |
| tan | GO:0051052 | regulation of DNA metabolic process | 3.60E-02 |
| tan | GO:0051651 | maintenance of location in cell | 3.73E-02 |
| tan | GO:0000132 | establishment of mitotic spindle orientation | 3.75E-02 |
| tan | GO:0051294 | establishment of spindle orientation | 3.75E-02 |
| tan | GO:0022616 | DNA strand elongation | 3.75E-02 |
| tan | GO:0032886 | regulation of microtubule-based process | 3.87E-02 |
| tan | GO:0031400 | negative regulation of protein modification process | 4.01E-02 |
| tan | GO:0042254 | ribosome biogenesis | 4.27E-02 |
| tan | GO:0010605 | negative regulation of macromolecule metabolic process | 4.31E-02 |
| tan | GO:0031503 | protein complex localization | 4.37E-02 |
| tan | GO:0032297 | negative regulation of DNA replication initiation | 4.37E-02 |
| tan | GO:0000076 | DNA replication checkpoint | 4.37E-02 |
| tan | GO:0000079 | regulation of cyclin-dependent protein kinase activity | 4.62E-02 |
| tan | GO:0006289 | nucleotide-excision repair | 4.77E-02 |
| tan | GO:0045132 | meiotic chromosome segregation | 4.98E-02 |
| greenyellow | GO:0016044 | membrane organization | 5.34E-08 |
| greenyellow | GO:0001568 | blood vessel development | 1.31E-05 |
| greenyellow | GO:0001944 | vasculature development | 1.65E-05 |
| greenyellow | GO:0030334 | regulation of cell migration | 2.18E-05 |
| greenyellow | GO:0040012 | regulation of locomotion | 5.94E-05 |
| greenyellow | GO:0051270 | regulation of cell motion | 6.19E-05 |
| greenyellow | GO:0048514 | blood vessel morphogenesis | 1.23E-04 |
| greenyellow | GO:0016337 | cell-cell adhesion | 3.52E-03 |
| greenyellow | GO:0007167 | enzyme linked receptor protein signaling pathway | 3.79E-03 |
| greenyellow | GO:0045621 | positive regulation of lymphocyte differentiation | 3.79E-03 |

| | | | |
|---|---|---|---|
| greenyellow | GO:0046634 | regulation of alpha-beta T cell activation | 4.11E-03 |
| greenyellow | GO:0043372 | positive regulation of CD4-positive, alpha beta T cell differentiation | 4.21E-03 |
| greenyellow | GO:0014706 | striated muscle tissue development | 4.43E-03 |
| greenyellow | GO:0045597 | positive regulation of cell differentiation | 4.70E-03 |
| salmon | GO:0006974 | response to DNA damage stimulus | 6.94E-06 |
| salmon | GO:0033554 | cellular response to stress | 6.86E-05 |
| salmon | GO:0007049 | cell cycle | 3.50E-04 |
| salmon | GO:0006281 | DNA repair | 1.06E-03 |
| salmon | GO:0006259 | DNA metabolic process | 2.05E-03 |
| salmon | GO:0022403 | cell cycle phase | 2.20E-03 |
| salmon | GO:0000279 | M phase | 2.45E-03 |
| salmon | GO:0007059 | chromosome segregation | 1.06E-02 |
| salmon | GO:0022402 | cell cycle process | 1.37E-02 |
| salmon | GO:0051327 | M phase of meiotic cell cycle | 1.77E-02 |
| salmon | GO:0007126 | meiosis | 1.77E-02 |
| salmon | GO:0051321 | meiotic cell cycle | 1.87E-02 |
| salmon | GO:0051276 | chromosome organization | 1.90E-02 |
| salmon | GO:0006350 | transcription | 2.13E-02 |
| salmon | GO:0051320 | S phase | 2.78E-02 |
| salmon | GO:0000084 | S phase of mitotic cell cycle | 2.78E-02 |
| salmon | GO:0008089 | anterograde axon cargo transport | 3.32E-02 |
| salmon | GO:0033043 | regulation of organelle organization | 3.37E-02 |
| lightgreen | GO:0006355 | regulation of transcription, DNA-dependent | 9.20E-05 |
| lightgreen | GO:0051252 | regulation of RNA metabolic process | 1.13E-04 |
| lightgreen | GO:0006350 | transcription | 4.31E-04 |
| lightgreen | GO:0045449 | regulation of transcription | 2.71E-03 |
| lightgreen | GO:0000279 | M phase | 1.98E-02 |
| lightgreen | GO:0022403 | cell cycle phase | 3.58E-02 |
| green | GO:0006955 | immune response | 2.04E-09 |
| green | GO:0006952 | defense response | 1.90E-08 |
| green | GO:0009611 | response to wounding | 4.09E-07 |
| green | GO:0006968 | cellular defense response | 8.93E-06 |
| green | GO:0045087 | innate immune response | 2.53E-05 |
| green | GO:0007166 | cell surface receptor linked signal transduction | 9.99E-05 |

| | | | |
|---|---|---|---|
| green | GO:0001568 | blood vessel development | 3.15E-04 |
| green | GO:0001944 | vasculature development | 3.99E-04 |
| green | GO:0042060 | wound healing | 4.59E-04 |
| green | GO:0006954 | inflammatory response | 4.93E-04 |
| green | GO:0002252 | immune effector process | 5.00E-04 |
| green | GO:0010648 | negative regulation of cell communication | 1.20E-03 |
| green | GO:0002449 | lymphocyte mediated immunity | 1.25E-03 |
| green | GO:0045730 | respiratory burst | 1.25E-03 |
| green | GO:0048878 | chemical homeostasis | 1.33E-03 |
| green | GO:0009968 | negative regulation of signal transduction | 1.52E-03 |
| green | GO:0002460 | adaptive immune response based on somatic recombination of immune receptors built from | 2.05E-03 |
| green | GO:0002250 | adaptive immune response | 2.05E-03 |
| green | GO:0006959 | humoral immune response | 2.34E-03 |
| green | GO:0019724 | B cell mediated immunity | 2.66E-03 |
| green | GO:0002684 | positive regulation of immune system process | 2.72E-03 |
| green | GO:0006916 | anti-apoptosis | 2.93E-03 |
| green | GO:0048514 | blood vessel morphogenesis | 3.50E-03 |
| green | GO:0002443 | leukocyte mediated immunity | 3.58E-03 |
| green | GO:0051605 | protein maturation by peptide bond cleavage | 3.58E-03 |
| green | GO:0007507 | heart development | 4.00E-03 |
| green | GO:0010324 | membrane invagination | 4.70E-03 |
| green | GO:0006897 | endocytosis | 4.70E-03 |
| green | GO:0007242 | intracellular signaling cascade | 5.02E-03 |
| green | GO:0051604 | protein maturation | 5.04E-03 |
| green | GO:0007202 | activation of phospholipase C activity | 5.09E-03 |
| green | GO:0010863 | positive regulation of phospholipase C activity | 5.09E-03 |
| green | GO:0007218 | neuropeptide signaling pathway | 5.26E-03 |
| green | GO:0030514 | negative regulation of BMP signaling pathway | 5.27E-03 |
| green | GO:0019835 | cytolysis | 5.27E-03 |
| green | GO:0006956 | complement activation | 5.66E-03 |
| green | GO:0055082 | cellular chemical homeostasis | 5.99E-03 |
| green | GO:0008219 | cell death | 6.14E-03 |
| green | GO:0002541 | activation of plasma proteins involved in acute inflammatory response | 6.16E-03 |
| green | GO:0006935 | chemotaxis | 6.37E-03 |

| | | | |
|---|---|---|---|
| green | GO:0042330 | taxis | 6.37E-03 |
| green | GO:0010518 | positive regulation of phospholipase activity | 6.55E-03 |
| green | GO:0016265 | death | 6.64E-03 |
| green | GO:0022604 | regulation of cell morphogenesis | 7.38E-03 |
| green | GO:0010517 | regulation of phospholipase activity | 7.39E-03 |
| green | GO:0048584 | positive regulation of response to stimulus | 7.65E-03 |
| green | GO:0006928 | cell motion | 7.84E-03 |
| green | GO:0016055 | Wnt receptor signaling pathway | 8.00E-03 |
| green | GO:0043066 | negative regulation of apoptosis | 8.16E-03 |
| green | GO:0016477 | cell migration | 8.17E-03 |
| green | GO:0043069 | negative regulation of programmed cell death | 9.08E-03 |
| green | GO:0006909 | phagocytosis | 9.09E-03 |
| green | GO:0060193 | positive regulation of lipase activity | 9.27E-03 |
| green | GO:0060548 | negative regulation of cell death | 9.39E-03 |
| green | GO:0018149 | peptide cross-linking | 9.68E-03 |
| green | GO:0034405 | response to fluid shear stress | 9.74E-03 |
| green | GO:0007204 | elevation of cytosolic calcium ion concentration | 1.16E-02 |
| green | GO:0050778 | positive regulation of immune response | 1.25E-02 |
| green | GO:0006873 | cellular ion homeostasis | 1.25E-02 |
| green | GO:0016485 | protein processing | 1.26E-02 |
| green | GO:0001889 | liver development | 1.28E-02 |
| green | GO:0001817 | regulation of cytokine production | 1.29E-02 |
| green | GO:0006958 | complement activation, classical pathway | 1.31E-02 |
| green | GO:0016064 | immunoglobulin mediated immune response | 1.36E-02 |
| green | GO:0008360 | regulation of cell shape | 1.36E-02 |
| green | GO:0051249 | regulation of lymphocyte activation | 1.39E-02 |
| green | GO:0032663 | regulation of interleukin-2 production | 1.44E-02 |
| green | GO:0030510 | regulation of BMP signaling pathway | 1.44E-02 |
| green | GO:0030178 | negative regulation of Wnt receptor signaling pathway | 1.44E-02 |
| green | GO:0002822 | regulation of adaptive immune response based on somatic recombination of immune receptors built from | 1.45E-02 |
| green | GO:0022409 | positive regulation of cell-cell adhesion | 1.45E-02 |
| green | GO:0019725 | cellular homeostasis | 1.46E-02 |
| green | GO:0002819 | regulation of adaptive immune response | 1.54E-02 |
| green | GO:0048661 | positive regulation of smooth muscle cell proliferation | 1.57E-02 |

| | | | |
|---|---|---|---|
| green | GO:0002455 | humoral immune response mediated by circulating immunoglobulin | 1.57E-02 |
| green | GO:0051480 | cytosolic calcium ion homeostasis | 1.60E-02 |
| green | GO:0007264 | small GTPase mediated signal transduction | 1.63E-02 |
| green | GO:0050900 | leukocyte migration | 1.64E-02 |
| green | GO:0060191 | regulation of lipase activity | 1.68E-02 |
| green | GO:0051674 | localization of cell | 1.70E-02 |
| green | GO:0048870 | cell motility | 1.70E-02 |
| green | GO:0012501 | programmed cell death | 1.80E-02 |
| green | GO:0008283 | cell proliferation | 1.83E-02 |
| green | GO:0042592 | homeostatic process | 1.88E-02 |
| green | GO:0045785 | positive regulation of cell adhesion | 1.94E-02 |
| green | GO:0032101 | regulation of response to external stimulus | 1.98E-02 |
| green | GO:0006875 | cellular metal ion homeostasis | 1.98E-02 |
| green | GO:0031329 | regulation of cellular catabolic process | 2.05E-02 |
| green | GO:0050905 | neuromuscular process | 2.17E-02 |
| green | GO:0050885 | neuromuscular process controlling balance | 2.18E-02 |
| green | GO:0015698 | inorganic anion transport | 2.18E-02 |
| green | GO:0010551 | regulation of specific transcription from RNA polymerase II promoter | 2.27E-02 |
| green | GO:0048565 | gut development | 2.35E-02 |
| green | GO:0001707 | mesoderm formation | 2.35E-02 |
| green | GO:0002694 | regulation of leukocyte activation | 2.44E-02 |
| green | GO:0050801 | ion homeostasis | 2.44E-02 |
| green | GO:0055065 | metal ion homeostasis | 2.51E-02 |
| green | GO:0030595 | leukocyte chemotaxis | 2.52E-02 |
| green | GO:0006022 | aminoglycan metabolic process | 2.53E-02 |
| green | GO:0002526 | acute inflammatory response | 2.67E-02 |
| green | GO:0048332 | mesoderm morphogenesis | 2.70E-02 |
| green | GO:0030324 | lung development | 2.77E-02 |
| green | GO:0032583 | regulation of gene-specific transcription | 2.79E-02 |
| green | GO:0060326 | cell chemotaxis | 2.89E-02 |
| green | GO:0007405 | neuroblast proliferation | 3.00E-02 |
| green | GO:0006915 | apoptosis | 3.03E-02 |
| green | GO:0001704 | formation of primary germ layer | 3.09E-02 |
| green | GO:0050817 | coagulation | 3.10E-02 |

| | | | |
|---|---|---|---|
| green | GO:0030323 | respiratory tube development | 3.10E-02 |
| green | GO:0007596 | blood coagulation | 3.10E-02 |
| green | GO:0050865 | regulation of cell activation | 3.12E-02 |
| green | GO:0016044 | membrane organization | 3.17E-02 |
| green | GO:0001701 | in utero embryonic development | 3.21E-02 |
| green | GO:0010553 | negative regulation of specific transcription from RNA polymerase II promoter | 3.29E-02 |
| green | GO:0048729 | tissue morphogenesis | 3.58E-02 |
| green | GO:0035295 | tube development | 3.60E-02 |
| green | GO:0051130 | positive regulation of cellular component organization | 3.65E-02 |
| green | GO:0021695 | cerebellar cortex development | 3.74E-02 |
| green | GO:0007599 | hemostasis | 3.82E-02 |
| green | GO:0060541 | respiratory system development | 3.82E-02 |
| green | GO:0045076 | regulation of interleukin-2 biosynthetic process | 4.13E-02 |
| green | GO:0005976 | polysaccharide metabolic process | 4.22E-02 |
| green | GO:0032496 | response to lipopolysaccharide | 4.32E-02 |
| green | GO:0030111 | regulation of Wnt receptor signaling pathway | 4.41E-02 |
| green | GO:0048660 | regulation of smooth muscle cell proliferation | 4.41E-02 |
| green | GO:0009617 | response to bacterium | 4.88E-02 |
| green | GO:0032582 | negative regulation of gene-specific transcription | 4.90E-02 |
| black | GO:0045449 | regulation of transcription | 1.03E-03 |
| black | GO:0051056 | regulation of small GTPase mediated signal transduction | 3.48E-03 |
| black | GO:0007243 | protein kinase cascade | 5.21E-03 |
| black | GO:0007167 | enzyme linked receptor protein signaling pathway | 8.51E-03 |
| black | GO:0046578 | regulation of Ras protein signal transduction | 1.36E-02 |
| black | GO:0007242 | intracellular signaling cascade | 1.57E-02 |
| black | GO:0007169 | transmembrane receptor protein tyrosine kinase signaling pathway | 1.89E-02 |
| black | GO:0032870 | cellular response to hormone stimulus | 2.24E-02 |
| black | GO:0000165 | MAPKKK cascade | 2.39E-02 |
| black | GO:0009967 | positive regulation of signal transduction | 2.65E-02 |
| black | GO:0051252 | regulation of RNA metabolic process | 2.66E-02 |
| black | GO:0006468 | protein amino acid phosphorylation | 3.07E-02 |
| black | GO:0035108 | limb morphogenesis | 3.17E-02 |
| black | GO:0035107 | appendage morphogenesis | 3.17E-02 |
| black | GO:0048598 | embryonic morphogenesis | 3.25E-02 |

| | | | |
|---|---|---|---|
| black | GO:0006355 | regulation of transcription, DNA-dependent | 3.30E-02 |
| black | GO:0009725 | response to hormone stimulus | 3.37E-02 |
| black | GO:0007050 | cell cycle arrest | 3.58E-02 |
| black | GO:0060173 | limb development | 3.58E-02 |
| black | GO:0048736 | appendage development | 3.58E-02 |
| black | GO:0007346 | regulation of mitotic cell cycle | 3.68E-02 |
| black | GO:0045941 | positive regulation of transcription | 4.01E-02 |
| black | GO:0010647 | positive regulation of cell communication | 4.58E-02 |
| black | GO:0043009 | chordate embryonic development | 4.72E-02 |
| black | GO:0016568 | chromatin modification | 4.82E-02 |
| black | GO:0010628 | positive regulation of gene expression | 4.84E-02 |
| black | GO:0009792 | embryonic development ending in birth or egg hatching | 4.93E-02 |
